# Pregnancy is linked to faster epigenetic aging in young women

**DOI:** 10.1073/pnas.2317290121

**Published:** 2024-04-08

**Authors:** Calen P. Ryan, Nanette R. Lee, Delia B. Carba, Julie L. MacIsaac, David T. S. Lin, Parmida Atashzay, Daniel W. Belsky, Michael S. Kobor, Christopher W. Kuzawa

**Affiliations:** ^a^Robert N. Butler Columbia Aging Center, Mailman School of Public Health, Columbia University, New York, NY 10032; ^b^USC-Office of Population Studies Foundation, University of San Carlos, Talamban, Cebu City 6000, Philippines; ^c^BC Children’s Hospital Research Institute, University of British Columbia, Vancouver, BC V5Z 4H4, Canada; ^d^Department of Epidemiology, Columbia University Mailman School of Public Health, Columbia University, New York, NY 10032; ^e^Child and Brain Development Program, Canadian Institute for Advanced Research, Toronto ON M5G 1M1, Canada; ^f^Department of Medical Genetics, Faculty of Medicine, University of British Columbia, Vancouver, BC V6T 2A1, Canada; ^g^Centre for Molecular Medicine and Therapeutics, Vancouver, BC V5Z 4H4, Canada; ^h^Department of Anthropology, Northwestern University, Evanston, IL 60208

**Keywords:** costs of reproduction, biological aging, pregnancy, fatherhood

## Abstract

A central prediction of evolutionary theory is that energy invested into reproduction comes at the expense of somatic maintenance and repair, accelerating biological aging. Supporting this prediction are findings that high fertility among women predicts shorter lifespan and poorer health later in life. However, biological aging is thought to begin before age-related health declines, limiting the applicability of morbidity and mortality for studying the aging process earlier in life. Here, we examine the relationship between reproductive history and biological aging in a sample of young (20 to 22yo) men and women from the Cebu Longitudinal Health and Nutrition Survey, located in the Philippines (n = 1,735). We quantify biological aging using six measures, collectively known as epigenetic clocks, reflecting various facets of cellular aging, health, and mortality risk. In a subset of women, we test whether longitudinal changes in gravidity between young and early–middle adulthood (25 to 31yo) are associated with changes in epigenetic aging during that time. Cross-sectionally, gravidity was associated with all six measures of accelerated epigenetic aging in women (n = 825). Furthermore, longitudinal increases in gravidity were linked to accelerated epigenetic aging in two epigenetic clocks (n = 331). In contrast, the number of pregnancies a man reported fathering was not associated with epigenetic aging among same-aged cohort men (n = 910). These effects were robust to socioecological, environmental, and immunological factors, consistent with the hypothesis that pregnancy accelerates biological aging and that these effects can be detected in young women in a high-fertility context.

## Significance

Energy invested into reproduction is thought to come at the expense of bodily maintenance. Consistent with this hypothesis, women with higher fertility tend to live shorter, less healthy lives. To test whether costs of reproduction are present prior to age-related health declines, we examined the effect of reproduction on six epigenetic clock measures of biological aging in 1,735 young adults in the Philippines. Cross-sectionally, pregnancy number was associated with faster aging across all six epigenetic clocks. Longitudinally, change in pregnancy number was associated with acceleration in two epigenetic clocks. The number of pregnancies fathered was not associated with aging among same-aged cohort men. Our findings support the hypothesis that pregnancy carries a cost that is detectable even in young women.

A central evolutionary theory of aging posits that reproduction will occur at the expense of maintenance and repair, leading to accelerated biological decline (1, 2). This idea has been supported across plant and animal taxa (3–5), including in humans. Based upon analyses of historical records spanning the 8th to 19th centuries, women in the British Aristocracy who had more progeny also sustained shorter postreproductive lifespans (6). Similar findings have been reported for frontier women settlers of early Québec and the South-West United States (ref. 7, but see ref. 8). While improvements in nutrition and medical care have likely attenuated some of the costs of reproduction in women (9), high parity is still associated with elevated morbidity (10–12) and all-cause mortality (ref. 13–16, but see ref. 17), even in industrialized, economically developed contexts.

Although past work is generally consistent with the premise that reproduction carries costs that accelerate biological aging in women, there are challenges to quantifying these costs using measures of health and mortality later in life. First, age-related morbidity and mortality only reach appreciable prevalence at more advanced chronological ages, limiting their utility in young populations (18, 19). In humans and other long-lived species, being able to quantify the costs of reproduction among the young confers both logistical and methodological advantages. Reducing the latency between the exposure and outcome minimizes the cumulative confounding effects of social and environmental factors that contribute to both reproductive decisions and pace of aging (20, 21). A second limitation when using morbidity and mortality to study aging is that these measures are often unidirectional, making them unsuitable for the study of individual changes in biological aging over time. Longitudinal studies of aging are crucial because individuals with the greatest capacity to acquire or allocate resources may be able to invest more into both fecundity and somatic maintenance, demonstrating both higher fertility and slower aging (22, 23).

A single gold standard for quantifying biological age across the lifespan is still lacking. However, new methods based on DNA methylation (DNAm) have recently been developed that show great promise to help fill this gap. These DNAm-based measures of aging—commonly known as “epigenetic clocks”—have been shown to accurately predict chronological age, mortality risk, and physiological decline (24, 25). Importantly, epigenetic clocks can be used to predict age acceleration before it becomes clinically apparent, providing an early indicator of biological aging in young adults. The predictive power of epigenetic clocks has brought them to the forefront of aging research (26) and points to their utility for quantifying tradeoffs between reproduction and aging (25).

Here, we use six epigenetic clocks to test for tradeoffs between reproduction and biological aging in a sample of 1,735 young adults in the Philippines. Participants come from the Cebu Longitudinal Health and Nutrition Survey, a long-running and well-characterized study of a birth cohort born in metropolitan Cebu, Philippines in 1983 (27, 28). Detailed, prospectively collected reproductive records and measures of the social and physical environment across life allow us to account for individual differences in access to resources or healthcare that may independently affect the pace of aging. Blood samples taken in 2005 (n = 825 women; n = 910 men) and again in 2009 to 2014 (n = 331 women) allow us to measure biological aging both cross-sectionally and longitudinally. This longitudinal approach minimizes the potential for residual confounding by time-invariant social and environmental exposures and provides a stronger test of causality for a tradeoff between reproduction and biological aging (29). We also test whether the number of pregnancies fathered was associated with biological aging in 910 same-aged men, allowing us to further isolate the effect of direct reproductive investment in the form of pregnancy on biological aging.

## Materials and Methods

### Population and Study Context.

Data are derived from the Cebu Longitudinal Health and Nutrition Survey (CLHNS), a birth cohort study started in 1983 to 84 in Metropolitan Cebu, Philippines, and are available for download at https://dataverse.unc.edu/dataverse/cebu. Blank versions of relevant surveys are also provided in *SI Appendix*. The current study focuses on surveys conducted in 2005 (baseline) and 2009 to 2014 (follow-up). Both men and women in this study came from the original birth cohort, and so were born in the same geographical areas (barangays or neighborhoods) at the same time (during enrollment in 1983 to 84), but were not necessarily affiliated in any other way. Descriptive statistics of both baseline and follow-up samples are provided in Table 1. Surveys were administered in participants’ homes by a trained interviewer, and included questions about mental and physical health, behavior, sociodemographic context. A subset of questions focused on reproductive history, including number of known pregnancies, their duration, and outcomes. For men, the number of pregnancies fathered was self-reported. For women, pregnancy status in 2005 was reported at the time of sampling, and through back-calculation from subsequent surveys based on parturition within 9 mo of the original interview (all past pregnancies and their outcomes are recorded as part of the ongoing tracking process). All past pregnancies reported by participants were counted regardless of outcome, including miscarriages, stillbirths, and live births. Three women reported having had twins, which counted as a single pregnancy. Household income, parental education, and assets were used to create a composite score of socioeconomic status, described in more detail in ref. 30 and below. Follow-up samples focused on a subset of participants enrolled in additional surveys tracking new pregnancies between 2009 and 2014. Informed consent was obtained from all participants, and data collection was conducted with approval and in accordance with the Institutional Review Boards of the University of North Carolina at Chapel Hill, the Office of Population Studies Foundation, and Northwestern University.

**Table 1.** Summary of sociodemographic characteristics, reproductive histories, and DNAm measures of aging for women (n = 825) and men (n = 910) used in cross-sectional analyses of reproductive history and epigenetic age and for a subsample of women at follow-up used in the longitudinal analyses (n = 331)

Characteristic	Baseline sample (Women) N = 825^*^	Baseline sample (Men) N = 910^*^	Follow-up sample (Women) N = 331^*^
Age	21.67 (0.36)	21.66 (0.34)	27.85 (1.53)
Pregnancy status at sampling			
Not pregnant	725 (87.9%)	*n/a*	0 (0%)
Pregnant	100 (12.1%)	*n/a*	331 (100%)
Pregnancy number	0.61 (0.93)	0.32 (0.70)	2.11 (1.62)
Horvath	−0.70 (3.9)	0.60 (3.8)	0.00 (3.6)
Hannum	−0.30 (3.4)	0.30 (3.5)	0.09 (2.63)
PhenoAge	0.30 (4.3)	−0.40 (3.9)	0.10 (4.4)
GrimAge	−0.80 (2.35)	0.67 (2.5)	−0.02 (2.03)
DunedinPACE	1.11 (0.12)	1.06 (0.1)	1.28 (0.10)
DNAmTL	0.04 (0.14)	0.04 (0.14)	0.00 (0.10)
Socioeconomic score	0.00 (1.56)	0.00 (1.60)	0.02 (1.42)
Current smoker (Y)	23 (2.8%)	386 (42.4%)	15 (4.5%)

^*^Mean (SD); n (%)

Clock values reported in Table 1 are residualized on chronological age and so are centered on zero (except DunedinPACE, which represents estimated pace of aging in units of years). Data come from the CLHNS, a longitudinal study of health and development based in Metropolitan Cebu, Philippines, and described in more detail in the *Materials and Methods* and elsewhere (27, 28).

### Socioeconomic Status Measure Construction.

A composite score of socioeconomic status was measured as a combination of income, education, and assets. Participants reported their annual income from all sources, including in-kind services, and the sale of livestock or other products by household members during the prior year, which were summed to determine total household income. Incomes were log-transformed. Maternal education (in years) was also reported. Participants also reported on ten assets (coded as 1 for possessing the asset in question, or 0 if not possessing the asset) that were selected to capture population-relevant aspects of social class, including electricity, refrigerators, air conditioners, color televisions, cable tv, tape recorders, electric fans, jeepneys, cars, trucks, and owning their residence. In addition, house construction type (i.e., light, mixed, permanent structure) was coded as 1, 2, or 3, respectively. Thus, asset scores ranged from 0 to 13. A principal components analysis was run on log income and assets, along with maternal education, at sample collection. The first dimension explained 70% of the variation in our composite SES score, and individual scores for the top component of variation were used as our measure of SES.

### Urbanicity Score Construction.

Urbanicity is associated with a range of health determinants in the Philippines (31). We used the urbanicity score developed by Dahly and Adair in this population to model this environmental effect (32). In brief, this scale was constructed using community-level data and validated with metrics of health in the CLHNS (32).

### Genetic Variation Measure Construction.

Principal components (PCs) of genome-wide genetic variation were included to control for potential population genetic structure. The derivation of these principal components has been described previously (33, 34).

### DNA Methylation and Epigenetic Clocks.

Blood samples for DNA methylation were collected concurrent with in-home interviews. Baseline blood samples were collected in EDTA-coated vacutainer tubes from overnight fasted subjects. Follow-up blood samples were collected using capillary whole blood collected on filter paper. DNA was extracted using a standard protocol; 750 ng of genomic DNA was treated with sodium bisulfite (Zyme EZDNA, Zymo Research, Irvine, CA) and 160 ng of converted DNA was applied to the Illumina Infinium MethylationEPIC BeadChip under standard conditions (Illumina Inc., San Diego, CA). Technicians were blind to information regarding participant characteristics, and samples were randomly assigned to plate, chip, and row. Background subtraction and color correction were performed using Illumina Genome Studio with default parameters. Data were then exported into R for further analysis. Quality control for baseline was performed as part of a larger sample to confirm participant sex and replicate status. This was followed by quantile normalization on all probes including SNP-associated and XY multiple binding probes. To maximize the number of sites available for the epigenetic age calculator, probes with detection *P*-values above 0.01 were called NA for poor-performing samples only and were otherwise retained (25). The same quality control steps were followed for follow-up samples. Epigenetic age for Horvath, Hannum, PhenoAge, GrimAge, and DNAmTL clocks were calculated using the original online calculator (http://labs.genetics.ucla.edu/horvath/dnamage/–new users are directed to https://dnamage.clockfoundation.org/). Principal component-based epigenetic clocks were calculated using the methods and code described in ref. 35. Background-corrected DNAm beta values (which represents the ratio of methylated beads to methylated and unmethylated beads) were processed further using the calculator’s internal normalization algorithms. The DunedinPACE clock was generated using the DunedinPACE calculator, available at https://github.com/danbelsky/DunedinPACE. Clock values reported in Table 1 are residualized on chronological age and so are centered on zero (except DunedinPACE, which represents estimated pace of aging in units of years). Equivalent months of epigenetic aging was determined by multiplying the SD of each epigenetic aging measure in the sample by the standardized effect size for that measure. For aging measures with units in years, this value was multiplied by 12 to provide equivalent months of epigenetic aging.

### Statistical Methods.

We first examined cross-sectional DNAm measures of aging for women at baseline who had never been pregnant compared to those who had. We next examined the relationship between cross-sectional DNAm measures of aging and gravidity as a continuous variable. In both cases, epigenetic age was the outcome of interest, with age, gravidity, pregnancy status at the time of blood sampling (i.e., whether or not a woman reported being pregnant at the time of the blood sample or was retrospectively deemed to be pregnant based on subsequent follow-up surveys), composite score of socioeconomic status, an urbanicity score (32), smoking status, and the top-10 principal components of genetic variation as covariates. Because the epigenetic clocks that we examined all aim to quantify potentially overlapping dimensions of biological aging, we did not consider tests of each clock to be independent tests and did not correct for multiple comparisons. This approach was prespecified in our preregistered analysis plan (https://osf.io/mqb37).

Exploratory data analysis and quality checks revealed potential outliers with values greater than three SD from the mean for several epigenetic clocks (*SI Appendix*, Fig. S1). In accordance with our preregistered analysis plan (https://osf.io/mqb37), we first fit ordinary least squares models including these values and examined diagnostics plots. These extreme observations were often high-leverage with large Cook’s distances, and had disproportionate influence on model estimates. The mean number of pregnancies reported were similar for those with epigenetic age estimates >3 SD from the mean compared to those with epigenetic age estimates =< 3 SD from the mean (mean pregnancies 0.57, SD 0.85 vs. mean pregnancies 0.52, SD 0.87, respectively). However, to moderate the influence of any single data point on the final estimates, and again in accordance with our preregistration protocol, we fit all models using a robust regression method defined by Yohai (36). We base our conclusions on the outcomes of the robust regression models. However, we also provide estimates from the ordinary least squares regression (including outliers) in *SI Appendix*, Tables S1–S20.

Next, we modeled longitudinal effects of reproduction by examining whether changes in DNAm measures of aging were associated with changes in gravidity. Here, change in epigenetic age was the outcome of interest, change in gravidity was the predictor of interest, and change in age, baseline pregnancy status at the time of blood sampling, composite score of socioeconomic status, urbanicity score, smoking status, and the top-10 principal components of genetic variation were included as covariates. Epigenetic clock estimates are based on DNAm derived from blood leukocytes. Because DNAm plays a key role in cellular identity, immune cell composition at the time of sampling may partly confound estimates of epigenetic age. To address this, we ran sensitivity analyses on all models that included bioinformatically estimated proportions of CD4T, CD8T, natural killer (NK), B cell, monocytes, and granulocytes (37). Cross-sectional analyses at baseline included baseline measures of immune cell counts, while longitudinal models included changes in cell counts between baseline and follow-up. Cross-sectional models using the same covariates (with the exclusion of current pregnancy status), including sensitivity models incorporating bioinformatically estimated cell composition, were run in same-aged cohort men. In keeping with our preregistration plan, all models were also fit using principal component-based versions of Horvath, Hannum, PhenoAge, GrimAge, and DNAmTL clocks (35). Data for the cross-sectional analysis of women is provided in *SI Appendix*, Data File 1. Data for the longitudinal analysis of women is provided in *SI Appendix*, Data File 2. Data for cross-sectional analysis of men is provided in *SI Appendix*, Data File 3.

## Results

The CLHNS is a prospective study of a single-year birth cohort started in 1983 with the enrollment of 3,327 pregnant women and their offspring in Metropolitan Cebu in the Philippines. Our sample consisted of 825 female and 910 male young adult participants who are members of the cohort of births from those pregnancies (see refs. 27 and 28).

At baseline in 2005, the women were aged 21.7, SD 0.36 y (range 20.8–22.5 y). Of 825 women, 314 had a history of at least one pregnancy at baseline, with 140 women having two or more pregnancies at baseline. Among women who had been pregnant, pregnancy number ranged from 1 to 5 (mean 1.61, SD 0.82). Similarly, at baseline, men were aged 21.7, SD 0.34 y (range 20.9 to 22.5 y). Of the 910 men in the study, 210 reported having a history of fathering at least one pregnancy at baseline. Among men who had fathered a pregnancy, the number of pregnancies fathered ranged from 1 to 6 (mean 1.41, SD 0.80).

In a subset of 331 female participants who became pregnant at least once during a longitudinal follow-up conducted from 2009 to 2014 (Table 1), we updated reproductive histories and collected additional blood samples for follow-up DNAm analysis. DNAm was collected for each woman’s last pregnancy during the follow-up period, which ranged in time from 3.5 to 9.0 y after baseline measurement. Excluding the current pregnancy, women reported having been pregnant between 0 and 7 times during follow-up (mean 1.31, SD 1.11).

### Cross-Sectional Analysis of Reproductive Effort and Biological Aging.

We first tested whether women who had been pregnant by early adulthood appeared biologically older than women who had not been pregnant. Following our preregistered analysis plan (https://osf.io/mqb37), we fit robust models as defined by Yohai (36) with pregnancy (ever vs. never pregnant) as the exposure and six epigenetic clocks as the outcomes of interest. Epigenetic clocks included the Horvath and Hannum first-generation clocks trained on chronological age; the PhenoAge and GrimAge second-generation clocks trained on mortality; the DunedinPACE pace of aging measure trained on the change in 19 indicators of organ-system integrity across two decades; and DNAmTL, a DNAm surrogate measure of leukocyte telomere length. As covariates, we included a composite measure of socioeconomic status that included all sources of household income, education, and family assets reflecting population-relevant aspects of social class; a measure of urbanicity of the participants’ primary residence; pregnancy status at the time of the blood sample; smoking status at the time of blood sample; and the top ten principal components of genome-wide genetic variation.

For all DNAm measures of aging, women with a history of at least one pregnancy appeared biologically older than women who had never been pregnant (Table 2). The effect sizes ranged from 0.14 SD for the GrimAge clock to 0.28 for DunedinPACE (Table 2). In contrast with other epigenetic clocks, pregnancy was associated with shorter DNAmTL, consistent with more advanced biological age. The effect of having been pregnant was equivalent to between 4.0 and 14.2 mo of accelerated aging for first- and second-generation clocks, an accelerated pace of aging of 3.3% per year according to DunedinPACE, and a shortening of 0.03 kilobases according to DNAmTL. Results for both robust models and ordinary least squares models including high-leverage data points are provided in *SI Appendix*, Table S1.

**Table 2.** Relationship between pregnancy (ever pregnant vs. never pregnant) and cross-sectional epigenetic age for six epigenetic clock measures of biological aging in 825 young women in the Philippines

	Horvath		PhenoAge		DunedinPACE	
Predictors	Estimates	*P*	Estimates	*P*	Estimates	*P*
Ever pregnant (yes)	0.26	**<0.001**	0.27	**<0.001**	0.28	**<0.001**
	(0.12–0.39)		(0.12–0.41)		(0.14–0.42)	
Observations	825		825		825	
R^2^/R^2^ adjusted	0.054/0.036		0.156/0.139		0.196/0.180	

	Hannum		GrimAge		DNAmTL	
Predictors	Estimates	*P*	Estimates	*P*	Estimates	*P*
Ever pregnant (yes)	0.17	**0.023**	0.14	0.062	–0.19	**0.012**
	(0.02–0.32)		(−0.01–0.29)		(−0.34– −0.04)	
Observations	825		825		825	
R^2^/R^2^ adjusted	0.107/0.090		0.168/0.152		0.106/0.089	

Estimates and 95% CI are in SD, equivalent to Cohen’s d, and *P*-values below alpha of 0.05 are bolded.

**Fig. 1.**
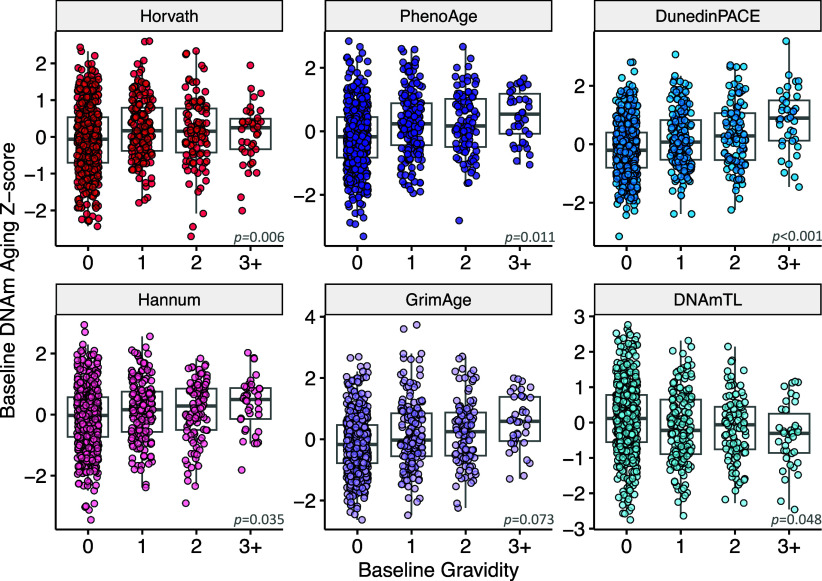
This figure shows the relationship between the number of pregnancies and cross-sectional measures of first-generation (Horvath and Hannum) and second-generation (PhenoAge and GrimAge) epigenetic clocks, DunedinPACE pace of aging, and a DNAm surrogate measure for leukocyte telomere length (DNAmTL). Higher values for all clocks correspond to accelerated biological aging, except for DNAmTL, where lower values correspond to shorter telomere length and accelerated aging. Four high-leverage data points with DNAm Aging values >3 SD from the mean are excluded from the figure but were included in all analyses using robust regression (n = 825). *P*-values are inset. Effect sizes and *P*-values are provided in Table 3.

Next, we tested whether women who experienced a greater number of pregnancies appeared biologically older than women with fewer or no pregnancies. Following our preregistered analysis plan (https://osf.io/mqb37), we fit robust models as defined by Yohai (36) with gravidity (number of pregnancies) as the exposure of interest, and the same six DNAm epigenetic clocks as the outcomes of interest. Socioeconomic status, urbanicity, pregnancy status, genetic variation, and smoking status were again included as covariates. For all DNAm measures of aging, women with a history of more pregnancies appeared biologically older than women with fewer pregnancies (Table 3). The per-pregnancy effect sizes ranged from 0.07 SD for the GrimAge clock to 0.16 SD for DunedinPACE. These effects are equivalent to 2.0 and 5.2 mo per pregnancy for first- and second-generation clocks, an accelerated pace of aging of 1.9% per year per pregnancy according to DunedinPACE, and shortening of 0.011 kilobases per pregnancy according to DNAmTL. Results for both robust models and ordinary least squares models are provided in *SI Appendix*, Table S3. Gradients in biological aging by number of pregnancies at study baseline are shown in Fig. 1.

**Table 3.** Relationship between pregnancy (number of times pregnant) and cross-sectional epigenetic age for six epigenetic clock measures of biological aging in 825 young women in the Philippines

	Horvath		PhenoAge		DunedinPACE	
Predictors	Estimates	*P*	Estimates	*P*	Estimates	*P*
Gravidity	0.10	**0.006**	0.10	**0.011**	0.16	**<0.001**
	(0.03–0.17)		(0.02–0.18)		(0.09–0.24)	
Observations	825		825		825	
R^2^/R^2^ adjusted	0.046/0.027		0.149/0.132		0.199/0.183	

	Hannum		GrimAge		DNAmTL	
Predictors	Estimates	*P*	Estimates	*P*	Estimates	*P*
Gravidity	0.08	**0.035**	0.07	0.073	−0.08	**0.048**
	(0.01–0.15)		(−0.01–0.15)		(−0.16– −0.00)	
Observations	825		825		825	
R^2^/R^2^ adjusted	0.106/0.088		0.168/0.151		0.103/0.086	

Estimates and 95% CI are in SD, equivalent to Cohen’s d, and *P*-values below alpha of 0.05 are bolded.

DNAm in blood can be affected by leukocyte composition at the time of sampling (38), which may be reflected in DNAm measures of aging (39, 40). To test the robustness of our findings to differences in leukocyte composition between women, we refit the above models with the addition of estimates of CD4T, CD8T, natural killer cells, B cells, monocytes, and granulocytes (37). Similar to models that did not include cell count, women with a history of at least one pregnancy appeared epigenetically older than women with no pregnancies, and women with a greater number of pregnancies appeared epigenetically older than women with fewer pregnancies. Standardized coefficients for the effect of gravidity on DNAm aging increased with the inclusion of cell count estimates for all clocks except Horvath and DNAmTL, which decreased modestly. Results for both robust models and ordinary least squares models controlling for cell counts are provided in *SI Appendix*, Tables S2 and S4. Cross-sectional models were also run using principal component-based versions of all clocks and yielded similar results (*SI Appendix,* Tables S11–S14).

### Longitudinal Changes in Reproductive Effort and Epigenetic Aging.

**Fig. 2.**
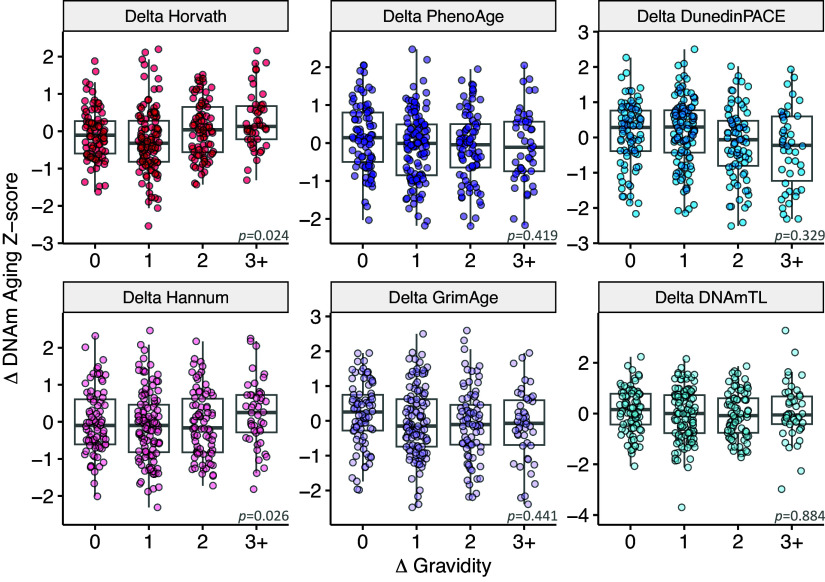
This figure shows the longitudinal association between the change in pregnancy number and change in biological aging measured using first-generation (Horvath and Hannum) and second-generation (PhenoAge and GrimAge) epigenetic clocks, DunedinPACE pace of aging, and a DNAm surrogate measure for leukocyte telomere length (DNAmTL). Higher values for all clocks correspond to accelerated biological aging, except for DNAmTL, where lower values correspond to shorter telomere length and accelerated aging. All women were pregnant at the second time point, but current pregnancies were not included in change scores. Four high-leverage data points with DNAm Aging values >3 SD from the mean are excluded from the figure but were included in all analyses using robust regression (n = 331). *P*-values are *Inset*. Effect sizes and *P* values are provided in Table 4.

We next asked whether the pace of biological aging that women experienced during the follow-up period was related to the number of pregnancies a woman had experienced during that period. By comparing women at follow-up to themselves at baseline, this longitudinal approach minimizes potential confounding by time-invariant, between-individual variation in health, access to resources, and other social and environmental exposures (29). Women who had more pregnancies between baseline and follow-up showed greater changes in both Horvath and Hannum first-generation clocks, but no significant changes according to the other DNAm measures of aging (Table 4 and Fig. 2). Horvath and Hannum effect sizes were similar to those of our cross-sectional analysis. Horvath epigenetic age increased 0.06 SD—equivalent to 2.9 mo—for each additional pregnancy (95% CI b = 0.01 to 0.11, *P* = 0.024). Hannum’s epigenetic clock increased 0.06 SD—equivalent to 2.4 mo—for each additional pregnancy (95% CI b = 0.01 to 0.11, *P* = 0.026). Results for both robust models and ordinary least squares models are provided in *SI Appendix*, Table S5. Sensitivity analyses that included changes in immune cell composition yielded similar results, with standardized coefficients showing almost no change with the inclusion of cell count estimates. Results for both robust models and ordinary least squares models controlling for cell counts are provided in *SI Appendix*, Table S6. Longitudinal models were also run using principal component-based versions of all clocks and yielded similar results (*SI Appendix*, Tables S15 and S16).

**Table 4.** Relationship between longitudinal changes in pregnancy number and longitudinal changes in six epigenetic clock measures of biological aging in 331 young women in the Philippines

	ΔHorvath		ΔPhenoAge		ΔDunedinPACE	
Predictors	Estimates	*P*	Estimates	*P*	Estimates	*P*
ΔGravidity	0.06	**0.024**	−0.03	0.419	−0.03	0.329
	(0.01, 0.11)		(−0.12, 0.05)		(−0.10, 0.03)	
Observations	331				331	
R^2^/R^2^ adjusted	0.485/0.457				0.438/0.408	

	ΔHannum		ΔGrimAge	*P*	ΔDNAmTL	
Predictors	Estimates	*P*	Estimates	*P*	Estimates	*P*
ΔGravidity	0.06	**0.026**	−0.03	0.441	−0.01	
	(0.01, 0.11)		(−0.12, 0.05)		(−0.07, 0.06)	
Observations	331				331	
R^2^/R^2^ adjusted	0.711/0.695				0.522/0.496	

Estimates and 95% CI are in SD, equivalent to Cohen’s d, and *P*-values below alpha of 0.05 are bolded.

### Cross-Sectional Analysis of Reproductive Effort and Biological Aging in Men.

In our final analysis, we tested whether the number of pregnancies cohort males fathered was associated with accelerated biological aging (n = 910). The men in this sample were born in the same geographical areas at the same time, allowing us to further disentangle potential sociocultural confounders of reproductive activity from the direct energetic investment of pregnancy and breastfeeding in the CLHNS. Using the same cross-sectional models described for the women in this cohort (minus the covariate for current pregnancy status), we found no evidence that men who reported fathering at least one pregnancy were biologically older than men who did not report fathering any pregnancies (*SI Appendix*, Table S7). In fact, men who fathered at least one pregnancy looked biologically younger according to Levine’s PhenoAge clock than men who did not father any pregnancies, but this effect was not statistically significant in sensitivity analyses that included immune cell composition (*SI Appendix*, Table S8). When examining number of pregnancies as a continuous variable, we found no evidence that men who reported fathering a greater number of pregnancies were epigenetically older than men who fathered fewer pregnancies (*SI Appendix*, Table S9). Sensitivity analyses that included immune cell composition yielded similar results, with standardized coefficients showing little change with the inclusion of cell count estimates (*SI Appendix*, Table S10). Cross-sectional models for men were also run using principal component-based versions of all clocks and yielded similar results (*SI Appendix*, Tables S17–S20).

## Discussion

Using six epigenetic clocks shown to predict mortality risk, physiological dysregulation, and biological decline, we provide evidence that women with a history of at least one pregnancy exhibited faster biological aging compared to those without a history of pregnancy. We also show that women who had been pregnant more often by early adulthood exhibited faster biological aging. These relationships were robust to potential social, environmental, and genetic confounding in the form of socioeconomic status and measures of urbanicity, smoking, and genetic variation, and were not attenuated after adjustment for differences in estimated cell composition. In a longitudinal analysis aimed to minimize the potential for confounding by unmeasured time-invariant social and environmental factors, women who experienced a greater number of pregnancies between baseline and follow-up exhibited faster rates of biological aging, but only as measured by the Horvath and Hannum clocks. In contrast, there was no evidence that the number of pregnancies fathered by men predicted epigenetic aging. Taken together, these findings provide evidence that pregnancy accelerates biological aging in a healthy, young adult population.

Our findings are largely consistent with our preregistered hypotheses based on evolutionary theory, and are in broad agreement with previous cross-sectional work documenting relationships between reproductive effort and DNAm measures of biological age. In a pilot sample from this population, we previously reported that gravidity was associated with accelerated aging using Horvath’s clock as well as leukocyte telomere length, another measure of molecular aging (30). Similarly, Kresovich and colleagues found evidence that parity was associated with faster Horvath, Hannum, and PhenoAge clocks in a large sample of American women participating in the Sister Study (41). Notably, the standardized effect sizes reported by Kresovich et al. for each additional pregnancy for both Horvath and Hannum clocks are comparable to those reported here (Horvath b = 0.10 vs. 0.10; Hannum b = 0.12 vs. 0.08). These findings contrast with a recent cross-sectional study among young Finnish women, where no relationship between nationally registered births and four measures of epigenetic aging was reported (42). To the extent that individual differences in access to resources and factors like healthcare might obscure tradeoffs at the population level, social and economic differences between countries may partly explain these divergent findings. The women in our study come mostly from low or middle-income households, have limited state-level social support, and limited access to high-quality healthcare (27). Furthermore, our study is characterized by comparatively high fertility and early age at reproductive debut. Support for costs of reproduction on biological aging in this population in the Philippines is consistent with the expectation that tradeoffs between reproduction and somatic maintenance will be greatest when resources are limited and reproductive effort is high (29).

Each additional pregnancy in our cross-sectional analysis was associated with an estimated effect of 4.0 to 14.2 mo of accelerated biological aging, while each additional pregnancy in our longitudinal analysis was associated with an estimated effect of 2.4 and 2.8 mo of accelerated biological aging. The higher estimated costs of gravidity for our cross-sectional analysis, when women were younger, are consistent with literature linking early maternal age at first birth with poorer long-term health outcomes later in life (43, 44). Using the conservative estimated effect sizes from our longitudinal analysis and hazard ratios for all-cause mortality from a large meta-analysis of first-generation clocks (45), we estimate this effect to be equivalent to an increase in all-cause mortality risk of 0.5 to 0.8% per pregnancy. Such modest effects could partly explain mixed support for costs of reproduction in studies that employ measures with less well-established links to long-term health and mortality risk (46). While relatively small, the potential long-term impact of small deviations in aging trajectories during early adulthood are unknown. Furthermore, such effects may be cumulative over multiple pregnancies, which typically exceed live births.

Although we control for a range of potential social, environmental, genetic, and immunological confounders in our cross-sectional analyses, estimates of the effect of gravidity on epigenetic clocks may be sensitive to residual confounding by variation not captured using our metrics. Nevertheless, none of the epigenetic clocks we examined were associated with the number of pregnancies fathered by young men in this same cohort. This suggests that it is the direct investment into gestation and breastfeeding rather than the socioeconomic factors tied to early life fertility (or sexual activity alone) that drives accelerated epigenetic aging in our study. Furthermore, longitudinal approaches that model the predictors of change in the outcome over time minimize the impact of factors that vary across individuals but are stable within individuals over time, such as birth weight, early life growth and development, family socioeconomic stratum, or parental education (47). Longitudinally, we found that women who had more pregnancies between baseline and follow-up aged more quickly according to both Horvath and Hannum clocks. This study connects longitudinal changes in pregnancy number to longitudinal changes in epigenetic age, thus providing a stronger basis for causal inference. However, the fact that this finding was restricted to Horvath and Hannum clocks—and not present for any of the other clocks that were associated with pregnancy number in our cross-sectional analyses—points to a potentially more complex relationship between reproduction and epigenetic aging in this population.

It is unclear why the effect of gravidity on longitudinal changes in epigenetic age was present for Horvath and Hannum clocks, but not the other epigenetic clocks that we examined. One potential factor is the underlying construction of the various clocks, which differ in the predictive targets and data used in their development. Both Horvath and Hannum were trained using machine-learning algorithms to predict chronological age. In contrast, PhenoAge, GrimAge, and DunedinPACE were trained using measures of blood chemistry, physiology, and organ-system integrity (48–50). The metabolic, immunological, and inflammatory profiles prevalent in high-income, Western settings, where these clocks were trained, often differ from those observed in the CLHNS and other non-Western contexts (51, 52). More importantly, all of our follow-up samples were taken from pregnant women, whose metabolic, physiological, and immunological profiles—as well as methylomes—are quite different from the largely nonpregnant population used in the training datasets (53–55). While it is unclear to what extent the physiological, metabolic, and immunological changes that accompany pregnancy are qualitatively distinct from variation in these states among nonpregnant people (55, 56), clocks built around these measures may be particularly sensitive to reproductive status. Because they are trained on chronological age, which is precisely measured and has similar meaning in all contexts, Horvath and Hannum clocks may be more robust to differences in biomarkers that vary between populations and with reproductive status. Nevertheless, confounding by reproductive status at our second time point would not explain the apparent dose-dependent effect of gravidity on Horvath and Hannum clocks—effects that were unaltered by our adjustment for immune cell composition. Additional work examining the impact of socioecological context (e.g., through exposures to infectious disease) and reproductive status on epigenetic clocks is needed (57).

Our findings should be interpreted in the context of several limitations. First, both our cross-sectional and longitudinal analyses focused on relatively young participants over a short timeframe. However, if the costs of reproduction are cumulative, becoming most evident at older ages and higher parity, our analysis may not capture the full impact of pregnancy on epigenetic age. Indeed, our work in a large representative sample of American women suggests that the effect of reproductive effort on some measures of biological aging may not be fully apparent until later in life (19). If this is the case in the Philippines, our focus on young women could underestimate the effect of gravidity on epigenetic aging. Second, we restricted our measure of reproductive effort to gravidity. While many of the metabolic, physiological, and immunological changes associated with pregnancy overlap with those that accompany aging (55, 56), breastfeeding and parental care are also thought to contribute to the long-term costs of reproduction. More work characterizing these other forms of reproductive investment—which in the case of breastfeeding requires detailed records of the frequency, duration, and intensity of breastfeeding for each child—and estimating their impact on biological aging is needed (22, 58). Finally, we are not yet able to link these DNAm measures of biological age to morbidity and mortality in later life in this population. While such links are now well-established among older individuals in the USA and Europe, the connection between faster biological aging and morbidity and mortality in young people, and in individuals living in non-Western contexts—where physiological and molecular aging follows different trajectories (e.g., ref. 59)—awaits future validation work in social and ecological contexts more similar to Cebu.

## Conclusions

This large-scale, preregistered study examined costs of reproduction in both young men and women in a high fertility context using state-of-the-art measures of biological aging. Our analyses controlled for a range of social, environmental, genetic, and immunological confounders. We find evidence supporting an effect of gravidity on epigenetic age, consistent with theorized tradeoffs between reproduction and aging, and supported by epidemiological findings that high reproductive effort may increase the risk for a range of diseases and early mortality. These findings suggest that gravidity accelerates biological aging, especially when carried out early in women’s reproductive careers, and that these effects may be detectable starting at a relatively young age.

## Supplementary Material

Appendix 01 (PDF)

Dataset S01 (CSV)

Dataset S02 (CSV)

Dataset S03 (CSV)

## Data Availability

Anonymized data are available in the *SI Appendix*, Data Files 1–3 and from the UNC Dataverse at https://dataverse.unc.edu/dataverse/cebu (60–62).

## References

[1] G. C. Williams, Natural Selection, the Costs of Reproduction, and a Refinement of Lack’s Principle. Am. Nat. **100**, 687–690 (1966).

[2] T. B. L. Kirkwood, Evolution of ageing. Nature. **270**, 301–304 (1977). 593350 10.1038/270301a0

[3] C. Dijkstra , Brood Size Manipulations in the Kestrel (Falco tinnunculus): Effects on Offspring and Parent Survival. J. Anim. Ecol. **59**, 269–285 (1990).

[4] J. R. Obeso, The costs of reproduction in plants. New Phytol. **155**, 321–348 (2002). 33873312 10.1046/j.1469-8137.2002.00477.x

[5] J. R. Speakman, The physiological costs of reproduction in small mammals. Philos. Trans. R. Soc. B Biol. Sci. **363**, 375–398 (2008). 10.1098/rstb.2007.2145 PMC2606756 17686735

[6] R. G. J. Westendorp, T. B. L. Kirkwood, Human longevity at the cost of reproductive success. Nature **396**, 743–746 (1998). 9874369 10.1038/25519

[7] A. Gagnon , Is there a trade-off between fertility and longevity? A comparative study of women from three large historical databases accounting for mortality selection. Am. J. Hum. Biol. **21**, 533–540 (2009). 19298004 10.1002/ajhb.20893 PMC6121733

[8] L. S. Hurt, C. Ronsmans, S. L. Thomas, The effect of number of births on women’s mortality: Systematic review of the evidence for women who have completed their childbearing. Popul. Stud. **60**, 55–71 (2006). 10.1080/00324720500436011 16464775

[9] E. Bolund, V. Lummaa, K. R. Smith, H. A. Hanson, A. A. Maklakov, Reduced costs of reproduction in females mediate a shift from a male-biased to a female-biased lifespan in humans. Sci. Rep. **6**, 24672 (2016). 27087670 10.1038/srep24672 PMC4834564

[10] H.-B. Guan, Q.-J. Wu, T.-T. Gong, Parity and Kidney Cancer Risk: Evidence from Epidemiologic Studies. Cancer Epidemiol. Prev. Biomark. **22**, 2345–2353 (2013). 10.1158/1055-9965.EPI-13-0759-T 24108791

[11] W. Li, W. Ruan, Z. Lu, D. Wang, Parity and risk of maternal cardiovascular disease: A dose–response meta-analysis of cohort studies. Eur. J. Prev. Cardiol. **26**, 592–602 (2019). 30567461 10.1177/2047487318818265

[12] H. Lv, H. Wu, J. Yin, J. Qian, J. Ge, Parity and Cardiovascular Disease Mortality: a Dose-Response Meta-Analysis of Cohort Studies. Sci. Rep. **5**, 13411 (2015). 26299306 10.1038/srep13411 PMC4547137

[13] E. Grundy, Women’s fertility and mortality in late mid life: A comparison of three contemporary populations. Am. J. Hum. Biol. **21**, 541–547 (2009). 19418527 10.1002/ajhb.20953

[14] E. Grundy, C. Tomassini, Fertility history and health in later life: a record linkage study in England and Wales. Soc. Sci. Med. **61**, 217–228 (2005). 15847974 10.1016/j.socscimed.2004.11.046

[15] A. Tamakoshi , Number of children and all-cause mortality risk: results from the Japan Collaborative Cohort Study. Eur. J. Public Health. **21**, 732–737 (2011). 21113028 10.1093/eurpub/ckq175

[16] Y. Zeng , Parity and All-cause Mortality in Women and Men: A Dose-Response Meta-Analysis of Cohort Studies. Sci. Rep. **6**, 19351 (2016). 26758416 10.1038/srep19351 PMC4725925

[17] E. Grundy, Ø. Kravdal, Reproductive History and Mortality in Late Middle Age among Norwegian Men and Women. Am. J. Epidemiol. **167**, 271–279 (2008). 18000019 10.1093/aje/kwm295

[18] D. W. Belsky , Quantification of biological aging in young adults. Proc. Natl. Acad. Sci. U.S.A. **112**, E4104–E4110 (2015). 26150497 10.1073/pnas.1506264112 PMC4522793

[19] T. N. Shirazi, W. J. Hastings, A. Y. Rosinger, C. P. Ryan, Parity predicts biological age acceleration in post-menopausal, but not pre-menopausal, women. Sci. Rep. **10**, 20522 (2020). 33239686 10.1038/s41598-020-77082-2 PMC7689483

[20] S.-L. Lai, N.-P. Tey, S.-T. Ng, Socio-economic Status and Fertility: A Study of Selected ASEAN Countries. Malays. J. Econ. Stud. **54**, 119–140 (2017).

[21] S. Schrempft , Associations between life course socioeconomic conditions and the Pace of Aging. J. Gerontol. Ser. A **77**, glab383 (2021). 10.1093/gerona/glab383 34951641

[22] E. Bolund, The challenge of measuring trade-offs in human life history research. Evol. Hum. Behav. **41**, S1090513820301185 (2020).

[23] A. J. van Noordwijk, G. de Jong, Acquisition and Allocation of Resources: Their Influence on Variation in Life History Tactics. Am. Nat. **128**, 137–142 (1986).

[24] S. Horvath, K. Raj, DNA methylation-based biomarkers and the epigenetic clock theory of ageing. Nat. Rev. Genet. **19**, 371–384 (2018). 29643443 10.1038/s41576-018-0004-3

[25] C. P. Ryan, “Epigenetic clocks”: Theory and applications in human biology. Am. J. Hum. Biol. **33**, 1–18 (2020). 10.1002/ajhb.23488 32845048

[26] L. Ferrucci , Measuring biological aging in humans: A quest. Aging Cell. **19**, e13080 (2020). 31833194 10.1111/acel.13080 PMC6996955

[27] L. S. Adair , Cohort Profile: The Cebu Longitudinal Health and Nutrition Survey. Int. J. Epidemiol. **40**, 619–625 (2011). 20507864 10.1093/ije/dyq085 PMC3147061

[28] C. W. Kuzawa , Evolutionary life history theory as an organising framework for cohort studies: insights from the Cebu Longitudinal Health and Nutrition Survey. Ann. Hum. Biol. **47**, 94–105 (2020). 32429766 10.1080/03014460.2020.1742787

[29] G. Jasienska, Costs of reproduction and ageing in the human female. *Philos*. Trans. R. Soc. B Biol. Sci. **375**, 20190615 (2020). 10.1098/rstb.2019.0615 PMC7540952 32951546

[30] C. P. Ryan , Reproduction predicts shorter telomeres and epigenetic age acceleration among young adult women. Sci. Rep. **8**, 11100 (2018). 30038336 10.1038/s41598-018-29486-4 PMC6056536

[31] K. J. G. Cheng, A. S. Rivera, R. T. D. P. Miguel, H. Y. Lam, A cross-sectional study on the determinants of health-related quality of life in the Philippines using the EQ-5D-5L. Qual. Life Res. **30**, 2137–2147 (2021), 10.1007/s11136-021-02799-0. 33677770

[32] D. L. Dahly, L. S. Adair, Quantifying the urban environment: a scale measure of urbanicity outperforms the urban-rural dichotomy. Soc. Sci. Med. **64**, 1407–1419 (2007). 17196724 10.1016/j.socscimed.2006.11.019 PMC2001275

[33] D. C. Croteau-Chonka , Genome-wide association study of anthropometric traits and evidence of interactions with age and study year in Filipino women. Obesity. **19**, 1019–1027 (2011). 20966902 10.1038/oby.2010.256 PMC3046220

[34] D. C. Croteau-Chonka , Population-specific coding variant underlies genome-wide association with adiponectin level. Hum. Mol. Genet. **21**, 463–471 (2012). 22010046 10.1093/hmg/ddr480 PMC3276282

[35] A. T. Higgins-Chen , A computational solution for bolstering reliability of epigenetic clocks: implications for clinical trials and longitudinal tracking. Nat. Aging **2**, 644–661 (2022). 36277076 10.1038/s43587-022-00248-2 PMC9586209

[36] V. J. Yohai, High Breakdown-Point and High Efficiency Robust Estimates for Regression. Ann. Stat. **15**, 642–656 (1987).

[37] E. A. Houseman , DNA methylation arrays as surrogate measures of cell mixture distribution. BMC Bioinformatics. **13**, 1–16 (2012). 22568884 10.1186/1471-2105-13-86 PMC3532182

[38] M. J. Ziller , Charting a dynamic DNA methylation landscape of the human genome. Nature. **500**, 477–481 (2013). 23925113 10.1038/nature12433 PMC3821869

[39] S. Komaki , Evaluation of short-term epigenetic age fluctuation. Clin. Epigenetics. **14**, 76 (2022). 35681206 10.1186/s13148-022-01293-9 PMC9185970

[40] Q. Zhang , Improved precision of epigenetic clock estimates across tissues and its implication for biological ageing. Genome Med. **11**, 54 (2019). 31443728 10.1186/s13073-019-0667-1 PMC6708158

[41] J. K. Kresovich , Reproduction, DNA methylation and biological age. Hum. Reprod. **34**, 1965–1973 (2019). 31600381 10.1093/humrep/dez149 PMC7209774

[42] E. W. Harville , Reproductive history and blood cell DNA methylation later in life: the Young Finns Study. Clin. Epigenetics. **13**, 227 (2021). 34930449 10.1186/s13148-021-01215-1 PMC8690999

[43] E. Grundy, E. Foverskov, Age at First Birth and Later Life Health in Western and Eastern Europe. Popul. Dev. Rev. **42**, 245–269 (2016).

[44] K. E. Peck , Adolescent childbirth and mobility disability among women ages 15–49: an analysis of population health surveys from 14 low-income and middle-income countries. BMJ Open **13**, e072535 (2023). 10.1136/bmjopen-2023-072535 PMC10360427 37474178

[45] B. H. Chen , DNA methylation-based measures of biological age: meta-analysis predicting time to death. Aging **8**, 1844 (2016). 27690265 10.18632/aging.101020 PMC5076441

[46] M. Gurven , Health costs of reproduction are minimal despite high fertility, mortality and subsistence lifestyle. Sci. Rep. **6**, 30056 (2016). 27436412 10.1038/srep30056 PMC4951795

[47] J. D. Singer, J. B. Willett, J. B. Willett, Applied Longitudinal Data Analysis: Modeling Change and Event Occurrence (Oxford University Press, New York, NY, 2003).

[48] D. W. Belsky , DunedinPACE, a DNA methylation biomarker of the pace of aging. eLife **11**, e73420 (2022). 35029144 10.7554/eLife.73420 PMC8853656

[49] M. E. Levine , An epigenetic biomarker of aging for lifespan and healthspan. Aging **10**, 19 (2018). 29676998 10.18632/aging.101414 PMC5940111

[50] A. T. Lu , DNA methylation GrimAge strongly predicts lifespan and healthspan. Aging **11**, 303–327 (2019). 30669119 10.18632/aging.101684 PMC6366976

[51] T. W. McDade, J. Rutherford, L. Adair, C. W. Kuzawa, Early origins of inflammation: microbial exposures in infancy predict lower levels of C-reactive protein in adulthood. Proc. R. Soc. B Biol. Sci. **277**, 1129–1137 (2010). 10.1098/rspb.2009.1795 PMC2842762 20007176

[52] T. W. McDade, J. N. Rutherford, L. Adair, C. Kuzawa, Population differences in associations between C-reactive protein concentration and adiposity: comparison of young adults in the Philippines and the United States. Am. J. Clin. Nutr. **89**, 1237–1245 (2009). 19225115 10.3945/ajcn.2008.27080 PMC2667466

[53] O. Gruzieva , DNA Methylation Trajectories During Pregnancy. Epigenetics Insights **12**, 251686571986709 (2019). 10.1177/2516865719867090 PMC6696836 31453433

[54] E. M. Miller, Changes in serum immunity during pregnancy. Am. J. Hum. Biol. **21**, 401–403 (2009). 19189417 10.1002/ajhb.20882

[55] C. P. Ryan , Immune cell type and DNA methylation vary with reproductive status in women: possible pathways for costs of reproduction. Evol. Med. Public Health. **10**, 47–58 (2022). 35169479 10.1093/emph/eoac003 PMC8841013

[56] A. Giller, M. Andrawus, D. Gutman, G. Atzmon, Pregnancy as a model for aging. Ageing Res. Rev. **62**, 101093 (2020). 32502628 10.1016/j.arr.2020.101093

[57] J. R. Poganik , Biological age is increased by stress and restored upon recovery. Cell Metab. **35**, 807–820.e5 (2023). 37086720 10.1016/j.cmet.2023.03.015 PMC11055493

[58] G. Jasienska, Reproduction and lifespan: Trade-offs, overall energy budgets, intergenerational costs, and costs neglected by research. Am. J. Hum. Biol. **21**, 524–532 (2009). 19367577 10.1002/ajhb.20931

[59] S. Horvath , An epigenetic clock analysis of race/ethnicity, sex, and coronary heart disease. Genome Biol. **17**, 171 (2016), 10.1186/s13059-016-1030-0. 27511193 PMC4980791

[60] Cebu Longitudinal Health and Nutrition Survey, Cebu child follow-up survey, 2005. UNC Dataverse. https://dataverse.unc.edu/dataset.xhtml?persistentId=hdl:1902.29/11701. Deposited 5 June 2014.

[61] Cebu Longitudinal Health and Nutrition Survey. Cebu mother baseline survey, 1983-1986. UNC Dataverse. https://dataverse.unc.edu/dataset.xhtml?persistentId=hdl:1902.29/11680.Deposited 5 June 2014.

[62] Cebu Longitudinal Health and Nutrition Survey. Cebu female index children follow-up survey, 2009. https://dataverse.unc.edu/dataset.xhtml?persistentId=hdl:1902.29/11706. Deposited 5 June 2014.

