# Supplementary material for: Pregnancy is linked to faster epigenetic aging in young women
Source: Proc Natl Acad Sci U S A. 2024 Apr 8;121(16):e2317290121. doi: 10.1073/pnas.2317290121 (PMC11032455; doi:10.1073/pnas.2317290121)
Supplement: Supplementary file 1 — Appendix 01 (PDF) [file pnas.2317290121.sapp.pdf]

PNAS

Supporting Information for

Pregnancy is linked to faster epigenetic aging in young women

Authors:
Calen P. Ryan[1*], Nanette R. Lee[2], Delia B. Carba[2], Julie L. MacIsaac, David S. Lin, Parmida Atashzay, Daniel W. Belsky, Michael S. Kobor, Christopher W. Kuzawa

*Correspondence to: Calen Patrick Ryan, Robert N. Butler Columbia Aging Center, Mailman School of Public Health, Columbia University, New York, NY, 10032, USA. Tel: (212)-305-5195. Email: cpr2139@cumc.columbia.edu

This PDF file includes:

Supplementary Materials

p1: Title Page
p2: Figure S1
pp3-13: Tables S1 to S10, Original Epigenetic Clocks
pp14-22: Tables S11 to 20, Principal Components-based Epigenetic Clocks
pp23-79: Cebu Mother Baseline Survey, 1983-1986 (CLHNSbaselineQRE.pdf)
pp80-194: Cebu Child Follow-up Survey, 2005 (CHILD05.pdf)
pp195-241: Cebu Child Pregnancy Tracking Survey, Pregnancy, 2009-2014 (pregnancyqre.pdf)
pp242-254: Cebu Child Pregnancy Tracking Survey, Birth outcomes, 2009-2014 (birthqre.pdf)

Supplementary Materials

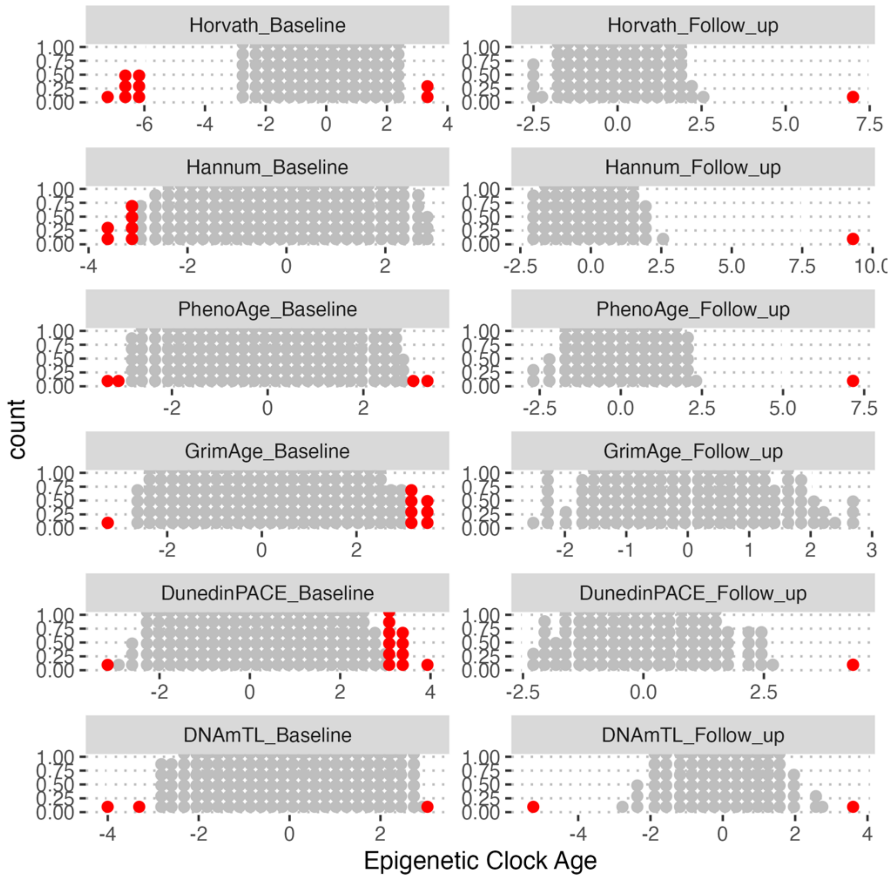

Figure S1. This figure shows scaled distributions of epigenetic clock values for young (20-22y) CLHNS participants included in the analysis (mean = 0; sd = 1). Baseline clock values for Horvath, Hannum, PhenoAge, GrimAge, DunedinPACE and DNAmTL are shown on the left-hand side for both women (n=825) and men (n=910). Follow-up values for the same clocks for women (n=331) are shown on the right-hand side. Observations with values greater than 3 standard deviations from the mean are highlighted in red. Rather than discard these extreme observations, and in accordance with our pre-registration protocol, our analyses focused on the outcomes of the robust regression method defined by Yohai (*35*).

Table S1. Comparison two statistical approaches to modelling the relationship between ever/never gravidity and epigenetic age in a cross-sectional sample of young women in the Philippines. Exploratory data analysis revealed four outliers (>3 SD from mean) in Horvath epigenetic age. Following a preregistered analysis plan, results are shown for ordinary least squares (M1) including outliers and using the robust regression method defined by Yohai (1987) (M2). All models include age, gravidity (ever vs. never), pregnancy status at blood draw, smoking status, an urbanicity score, a composite score for socioeconomic status, and the top-10 genetic PC scores.

	**M1: OLS (n = 825)**		**M2: Robust (n = 825)**	
	Estimates (95% CI)	*p*	*Estimates (95% CI)*	*p*
Horvath	0.26 (0.11 – 0.41)	**0.001**	0.26 (0.12 – 0.39)	**<0.001**
Hannum	0.17 (0.03 – 0.31)	**0.021**	0.17 (0.02 – 0.32)	**0.023**
PhenoAge	0.28 (0.14 – 0.42)	**<0.001**	0.27 (0.12 – 0.41)	**<0.001**
GrimAge	0.16 (0.03 – 0.30)	**0.019**	0.14 (-0.01 – 0.29)	0.062
DunedinPACE	0.29 (0.15 – 0.43)	**<0.001**	0.28 (0.14 – 0.42)	**<0.001**
DNAmTL	-0.20 (-0.34 – -0.05)	**0.007**	-0.19 (-0.34 – -0.04)	**0.012**

Table S2. Comparison two statistical approaches to modelling the relationship between ever/never gravidity and epigenetic age in a cross-sectional sample of young women in the Philippines, controlling for cell counts (CD4T, CD8T, NK, B cell, Granulocyte, and Monocyte). Prior analysis, exploratory data analysis revealed four outliers (>3 SD from mean) in Horvath epigenetic age. Following a preregistered analysis plan, results are shown for ordinary least squares including (M1) and using the robust regression method defined by Yohai (1987) (M2). All models include age, gravidity, pregnancy status at blood draw, smoking status, an urbanicity score, a composite score for socioeconomic status, and the top-10 genetic PC scores. Effects of having been pregnant are equivalent to between 6.2 and 13.6 months of accelerated aging for first- and second-generation clocks, an accelerated pace of aging of 3% per year according to DunedinPACE, and a shortening of 0.017 kilobases according to DNAmTL.

	**M1: OLS (n = 825)**		**M2: Robust (n = 825)**	
	Estimates (95% CI)	*p*	*Estimates (95% CI)*	*p*
Horvath	0.24<br>(0.09 – 0.39)	**0.002**	0.24<br>(0.10 – 0.37)	**0.001**
Hannum	0.17<br>(0.04 – 0.29)	**0.008**	0.19<br>(0.07 – 0.31)	**0.003**
PhenoAge	0.30<br>(0.18 – 0.43)	**<0.001**	0.28<br>(0.15 – 0.40)	**<0.001**
GrimAge	0.24<br>(0.12 – 0.36)	**<0.001**	0.23<br>(0.10 – 0.36)	**<0.001**
DunedinPACE	0.31<br>(0.19 – 0.44)	**<0.001**	0.31<br>(0.18 – 0.43)	**<0.001**
DNAmTL	-0.12<br>(-0.24 – 0.01)	0.062	-0.11<br>(-0.23 – 0.02)	0.106

Table S3. Comparison of two statistical approaches to modelling the relationship between gravidity and epigenetic age in a cross-sectional sample of young women in the Philippines. Prior analysis, exploratory data analysis revealed four outliers (>3 SD from mean) in Horvath epigenetic age. Following a preregistered analysis plan, results are shown for ordinary least squares including (M1) and using the robust regression method defined by Yohai (1987) (M2). All models include age, gravidity, pregnancy status at blood draw, smoking status, an urbanicity score, a composite score for socioeconomic status, and the top-10 genetic PC scores.

	**M1: OLS (n = 825)**		**M2: Robust (n = 825)**	
	Estimates (95% CI)	*p*	*Estimates (95% CI)*	*p*
Horvath	0.12<br>(0.04 – 0.19)	**0.004**	0.10<br>(0.03 – 0.17)	**0.006**
Hannum	0.07<br>(-0.00 – 0.15)	0.064	0.08<br>(0.01 – 0.15)	**0.035**
PhenoAge	0.11<br>(0.03 – 0.18)	**0.004**	0.10<br>(0.02 – 0.18)	**0.011**
GrimAge	0.08<br>(0.00 – 0.15)	**0.044**	0.07<br>(-0.01 – 0.15)	0.073
DunedinPACE	0.17<br>(0.10 – 0.24)	**<0.001**	0.16<br>(0.09 – 0.24)	**<0.001**
DNAmTL	-0.09<br>(-0.16 – -0.01)	**0.025**	-0.08<br>(-0.16 – -0.00)	**0.048**

Table S4. Comparison of two statistical approaches to modelling the relationship between gravidity and epigenetic age in a cross-sectional sample of young women in the Philippines, controlling for cell counts (CD4T, CD8T, NK, B cell, Granulocyte, and Monocyte). Prior analysis, exploratory data analysis revealed four outliers (>3 SD from mean) in Horvath epigenetic age. Following a preregistered analysis plan, results are shown for ordinary least squares including (M1) and using the robust regression method defined by Yohai (1987) (M2). All models include age, gravidity, pregnancy status at blood draw, smoking status, an urbanicity score, a composite score for socioeconomic status, and the top-10 genetic PC scores. The effect sizes were equivalent to between 2.8 and 5.2 months per pregnancy for first- and second-generation clocks, an accelerated pace of aging of 2% per year per pregnancy according to DunedinPACE, and shortening of 0.008 kilobases per pregnancy according to DNAmTL.

	**M1: OLS (n = 825)**		**M2: Robust (n = 825)**	
	Estimates (95% CI)	*p*	*Estimates (95% CI)*	*p*
Horvath	0.10 (0.03 – 0.18)	**0.009**	0.09 (0.01 – 0.16)	**0.020**
Hannum	0.09 (0.02 – 0.15)	**0.009**	0.10 (0.04 – 0.16)	**0.002**
PhenoAge	0.12 (0.05 – 0.19)	**<0.001**	0.12 (0.05 – 0.18)	**<0.001**
GrimAge	0.12 (0.05 – 0.18)	**<0.001**	0.12 (0.05 – 0.19)	**0.001**
DunedinPACE	0.18 (0.12 – 0.25)	**<0.001**	0.18 (0.11 – 0.24)	**<0.001**
DNAmTL	-0.06 (-0.12 – 0.01)	0.086	-0.05 (-0.11 – 0.02)	0.168

Table S5. Comparison of two statistical approaches to modelling the relationship between changes in gravidity and changes in epigenetic age in young women in the Philippines. Prior analysis, exploratory data analysis revealed four outliers (>3 SD from mean) in Horvath epigenetic age. Following a preregistered analysis plan, results are shown for ordinary least squares including (M1) and using the robust regression method defined by Yohai (1987) (M2). All models include delta age, delta pregnancy, and pregnancy status at blood draw, smoking status, an urbanicity score, and a composite score for socioeconomic status at baseline, and the top-10 genetic PC scores.

	**M1: OLS (N = 331)**		**M2: Robust (N = 331)**	
	Estimates (95% CI)	*p*	*Estimates (95% CI)*	*p*
∆Horvath	0.03 (-0.04 – 0.11)	0.383	0.06 (0.01 – 0.11)	**0.024**
∆Hannum	0.04 (-0.03 – 0.11)	0.280	0.06 (0.01 – 0.11)	**0.026**
∆PhenoAge	-0.06 (-0.14 – 0.02)	0.155	-0.03 (-0.12 – 0.05)	0.419
∆GrimAge	-0.04 (-0.11 – 0.04)	0.348	-0.03 (-0.12 – 0.05)	0.441
∆DunedinPACE	-0.03 (-0.11 – 0.04)	0.369	-0.03 (-0.10 – 0.03)	0.329
∆DNAmTL	0.02 (-0.05 – 0.10)	0.560	0.01 (-0.07 – 0.06)	0.884

Table S6. Comparison of two statistical approaches to modelling the relationship between changes in gravidity and changes in epigenetic age in young women in the Philippines, controlling for cell counts (CD4T, CD8T, NK, B cell, Granulocyte, and Monocyte). Prior analysis, exploratory data analysis revealed four outliers (>3 SD from mean) in Horvath epigenetic age. Following a preregistered analysis plan, results are shown for ordinary least squares including (M1) and using the robust regression method defined by Yohai (1987) (M2). All models include delta age, delta pregnancy, and pregnancy status at blood draw, smoking status, an urbanicity score, and composite score for socioeconomic status at baseline, and the top-10 genetic PC scores. Horvath epigenetic age increased 0.06 standard deviations – equivalent to 2.9 months – for each additional pregnancy. Hannum's epigenetic clock increased 0.06 standard deviations – equivalent to 2.4 months – for each additional pregnancy.

	**M1: OLS (N = 331)**		**M2: Robust (N = 331)**	
	Estimates (95% CI)	*p*	*Estimates (95% CI)*	*p*
ΔHorvath	0.02<br>(-0.06 – 0.09)	0.624	0.06<br>(-0.00 – 0.11)	**0.050**
ΔHannum	0.03<br>(-0.03 – 0.10)	0.348	0.06<br>(0.01 – 0.12)	**0.014**
ΔPhenoAge	-0.06<br>(-0.14 – 0.02)	0.149	-0.02<br>(-0.10 – 0.06)	0.610
ΔGrimAge	-0.03<br>(-0.10 – 0.04)	0.460	-0.02<br>(-0.10 – 0.06)	0.646
ΔDunedinPACE	-0.05<br>(-0.11 – 0.02)	0.186	-0.05<br>(-0.11 – 0.01)	0.092
ΔDNAmTL	0.03<br>(-0.04 – 0.11)	0.336	0.00<br>(-0.07 – 0.08)	0.952

Table S7. Comparison of two statistical approaches to modelling the relationship between whether or not a man reported fathering at least one pregnancy and epigenetic age in a cohort of young men in the Philippines. Prior analysis, exploratory data analysis revealed six outliers (>3 SD from mean) in Horvath epigenetic age. Following a preregistered analysis plan, results are shown for ordinary least squares including (M1) and using the robust regression method defined by Yohai (1987) (M2). All models include age, number of pregnancies fathered, smoking status in 2005, an urbanicity score, a composite score for socioeconomic status, and the top-10 genetic PC scores. Men who reported fathering at least one pregnancy had PhenoAge estimates 0.18 standard deviations below men who had not fathered any pregnancies, equivalent to 8.6 months of biological aging in this population.

	**M1: OLS (N = 910)**		**M2: Robust (N = 910)**	
	Estimates (95% CI)	*p*	*Estimates (95% CI)*	*p*
Horvath	-0.09 (-0.24 – 0.07)	0.263	-0.07 (-0.22 – 0.08)	0.353
Hannum	-0.07 (-0.22 – 0.08)	0.349	-0.08 (-0.24 – 0.07)	0.305
PhenoAge	-0.13 (-0.29 – 0.02)	0.087	-0.17 (-0.33 – -0.02)	**0.026**
GrimAge	-0.06 (-0.21 – 0.09)	0.427	-0.03 (-0.18 – 0.12)	0.663
DunedinPACE	-0.10 (-0.26 – 0.06)	0.216	-0.08 (-0.24 – 0.08)	0.340
DNAmTL	-0.00 (-0.16 – 0.15)	0.963	0.05 (-0.12 – 0.21)	0.587

Table S8. Comparison of two statistical approaches to modelling the relationship between whether or not a man reported fathering at least one pregnancy and epigenetic age in a cohort of young men in the Philippines, controlling for cell counts (CD4T, CD8T, NK, B cell, Granulocyte, and Monocyte). Prior analysis, exploratory data analysis revealed six outliers (>3 SD from mean) in Horvath epigenetic age. Following a preregistered analysis plan, results are shown for ordinary least squares including (M1) and using the robust regression method defined by Yohai (1987) (M2). All models include age, number of pregnancies fathered, smoking status in 2005, an urbanicity score, a composite score for socioeconomic status, and the top-10 genetic PC scores. Controlling for cell counts, reporting being responsible for at least one pregnancy was not associated with any DNAm measures of aging.

	**M1: OLS (N = 910)**		**M2: Robust (N = 910)**	
	Estimates (95% CI)	*p*	*Estimates (95% CI)*	*p*
Horvath	-0.08 (-0.23 – 0.07)	0.312	-0.08 (-0.21 – 0.06)	0.287
Hannum	-0.02 (-0.14 – 0.11)	0.797	-0.02 (-0.14 – 0.11)	0.807
PhenoAge	-0.09 (-0.23 – 0.05)	0.201	-0.12 (-0.26 – 0.02)	0.104
GrimAge	0.03 (-0.10 – 0.16)	0.672	0.06 (-0.07 – 0.19)	0.347
DunedinPACE	-0.08 (-0.23 – 0.06)	0.271	-0.07 (-0.22 – 0.08)	0.349
DNAmTL	0.03 (-0.09 – 0.15)	0.654	0.04 (-0.08 – 0.17)	0.500

Table S9. Comparison of two statistical approaches to modelling the relationship between the number of pregnancies a man fathered and epigenetic age in a cohort of young men in the Philippines. Prior analysis, exploratory data analysis revealed six outliers (>3 SD from mean) in Horvath epigenetic age. Following a preregistered analysis plan, results are shown for ordinary least squares including (M1) and using the robust regression method defined by Yohai (1987) (M2). All models include age, number of pregnancies fathered, smoking status in 2005, an urbanicity score, a composite score for socioeconomic status, and the top-10 genetic PC scores. The number of pregnancies a man fathered was not associated with any DNAm measures of aging.

	**M1: OLS (N = 910)**		**M2: Robust (N = 910)**	
	Estimates (95% CI)	*p*	*Estimates (95% CI)*	*p*
Horvath	-0.02 (-0.12 – 0.07)	0.611	-0.01 (-0.09 – 0.07)	0.822
Hannum	-0.01 (-0.10 – 0.08)	0.794	-0.02 (-0.10 – 0.07)	0.732
PhenoAge	-0.04 (-0.13 – 0.05)	0.404	-0.06 (-0.17 – 0.05)	0.277
GrimAge	-0.01 (-0.10 – 0.08)	0.801	0.00 (-0.07 – 0.08)	0.967
DunedinPACE	-0.03 (-0.13 – 0.06)	0.504	-0.02 (-0.12 – 0.09)	0.750
DNAmTL	-0.02 (-0.11 – 0.07)	0.729	0.01 (-0.07 – 0.08)	0.863

Table S10. Comparison of two statistical approaches to modelling the relationship between the number of pregnancies a man fathered and epigenetic age in a cohort of young men in the Philippines, controlling for cell counts (CD4T, CD8T, NK, B cell, Granulocyte, and Monocyte). Prior analysis, exploratory data analysis revealed six outliers (>3 SD from mean) in Horvath epigenetic age. Following a preregistered analysis plan, results are shown for ordinary least squares including (M1) and using the robust regression method defined by Yohai (1987) (M2). All models include age, number of pregnancies fathered, smoking status in 2005, an urbanicity score, a composite score for socioeconomic status, and the top-10 genetic PC scores. Controlling for cell counts, the number of pregnancies a man fathered was not associated with any DNAm measures of aging.

	**M1: OLS (N = 910)**		**M2: Robust (N = 910)**	
	Estimates (95% CI)	*p*	*Estimates (95% CI)*	*p*
Horvath	-0.02 (-0.11 – 0.07)	0.673	-0.01 (-0.09 – 0.07)	0.786
Hannum	-0.01 (-0.08 – 0.07)	0.888	-0.01 (-0.07 – 0.05)	0.806
PhenoAge	-0.03 (-0.11 – 0.05)	0.443	-0.05 (-0.14 – 0.04)	0.315
GrimAge	0.02 (-0.06 – 0.10)	0.618	0.03 (-0.04 – 0.10)	0.367
DunedinPACE	-0.03 (-0.12 – 0.06)	0.513	-0.01 (-0.10 – 0.08)	0.825
DNAmTL	0.01 (-0.06 – 0.08)	0.783	0.02 (-0.04 – 0.08)	0.523

Table S11. Comparison two statistical approaches to modelling the relationship between ever/never gravidity and principal component (PC)-based epigenetic age (Higgins-Chen et al. 2022) in a cross-sectional sample of young women in the Philippines. Exploratory data analysis revealed four outliers (>3 SD from mean) in Horvath epigenetic age. Following a preregistered analysis plan, results are shown for ordinary least squares (M1) including outliers and using the robust regression method defined by Yohai (1987) (M2). All models include age, gravidity (ever vs. never), pregnancy status at blood draw, smoking status, an urbanicity score, a composite score for socioeconomic status, and the top-10 genetic PC scores.

	**M1: OLS (n = 825)**		**M2: Robust (n = 825)**	
	Estimates (95% CI)	*p*	*Estimates (95% CI)*	*p*
PCHorvath†	0.27<br>(0.13 – 0.42)	**<0.001**	0.28<br>(0.13 – 0.43)	**<0.001**
PCHannum†	0.21<br>(0.07 – 0.36)	**0.004**	0.22<br>(0.08 – 0.37)	**0.003**
PCPhenoAge†	0.23<br>(0.09 – 0.37)	**0.001**	0.21<br>(0.07 – 0.34)	**0.003**
PCGrimAge†	0.11<br>(-0.03 – 0.24)	0.119	0.10<br>(-0.04 – 0.24)	0.145
DunedinPACE†	0.29<br>(0.15 – 0.43)	**<0.001**	0.28<br>(0.14 – 0.42)	**<0.001**
PCDNAmTL†	-0.25<br>(-0.40 – -0.11)	**0.001**	-0.26<br>(-0.40 – -0.11)	**0.001**

†All clocks are calculated using the principal component-based methods described by Higgins-Chen et al. 2022, except DunedinPACE for which there is no PC-based version.

Table S12. Comparison two statistical approaches to modelling the relationship between ever/never gravidity and principal component (PC)-based epigenetic age (Higgins-Chen et al. 2022) in a cross-sectional sample of young women in the Philippines, controlling for cell counts (CD4T, CD8T, NK, B cell, Granulocyte, and Monocyte). Prior analysis, exploratory data analysis revealed four outliers (>3 SD from mean) in Horvath epigenetic age. Following a preregistered analysis plan, results are shown for ordinary least squares including (M1) and using the robust regression method defined by Yohai (1987) (M2). All models include age, gravidity, pregnancy status at blood draw, smoking status, an urbanicity score, a composite score for socioeconomic status, and the top-10 genetic PC scores.

	**M1: OLS (n = 825)**		**M2: Robust (n = 825)**	
	Estimates (95% CI)	*p*	*Estimates (95% CI)*	*p*
PCHorvath†	0.19 (0.06 – 0.33)	**0.006**	0.20 (0.06 – 0.33)	**0.005**
PCHannum†	0.14 (0.03 – 0.26)	**0.016**	0.14 (0.02 – 0.27)	**0.024**
PCPhenoAge†	0.21 (0.12 – 0.31)	**<0.001**	0.21 (0.11 – 0.31)	**<0.001**
PCGrimAge†	0.16 (0.07 – 0.26)	**0.001**	0.16 (0.07 – 0.26)	**0.001**
DunedinPACE†	0.31 (0.19 – 0.44)	**<0.001**	0.31 (0.18 – 0.43)	**<0.001**
PCDNAmTL†	-0.14 (-0.25 – -0.02)	**0.018**	-0.13 (-0.24 – -0.01)	**0.031**

†All clocks are calculated using the principal component-based methods described by Higgins-Chen et al. 2022, except DunedinPACE for which there is no PC-based version.

Table S13. Comparison of two statistical approaches to modelling the relationship between gravidity and principal component (PC)-based epigenetic age (Higgins-Chen et al. 2022) in a cross-sectional sample of young women in the Philippines. Prior analysis, exploratory data analysis revealed four outliers (>3 SD from mean) in Horvath epigenetic age. Following a preregistered analysis plan, results are shown for ordinary least squares including (M1) and using the robust regression method defined by Yohai (1987) (M2). All models include age, gravidity, pregnancy status at blood draw, smoking status, an urbanicity score, a composite score for socioeconomic status, and the top-10 genetic PC scores.

	**M1: OLS (n = 825)**		**M2: Robust (n = 825)**	
	Estimates (95% CI)	*p*	*Estimates (95% CI)*	*p*
PCHorvath†	0.11 (0.03 – 0.19)	**0.005**	0.11 (0.04 – 0.19)	**0.004**
PCHannum†	0.10 (0.02 – 0.17)	**0.015**	0.09 (0.02 – 0.17)	**0.012**
PCPhenoAge†	0.11 (0.03 – 0.18)	**0.005**	0.10 (0.02 – 0.18)	**0.011**
PCGrimAge†	0.07 (-0.01 – 0.14)	0.073	0.07 (-0.00 – 0.15)	0.063
DunedinPACE†	0.17 (0.10 – 0.24)	**<0.001**	0.16 (0.09 – 0.24)	**<0.001**
PCDNAmTL†	-0.11 (-0.19 – -0.03)	**0.004**	-0.11 (-0.19 – -0.04)	**0.003**

†All clocks are calculated using the principal component-based methods described by Higgins-Chen et al. 2022, except DunedinPACE for which there is no PC-based version.

Table S14. Comparison of two statistical approaches to modelling the relationship between gravidity and principal component (PC)-based epigenetic age (Higgins-Chen et al. 2022) in a cross-sectional sample of young women in the Philippines, controlling for cell counts (CD4T, CD8T, NK, B cell, Granulocyte, and Monocyte). Prior analysis, exploratory data analysis revealed four outliers (>3 SD from mean) in Horvath epigenetic age. Following a preregistered analysis plan, results are shown for ordinary least squares including (M1) and using the robust regression method defined by Yohai (1987) (M2). All models include age, gravidity, pregnancy status at blood draw, smoking status, an urbanicity score, a composite score for socioeconomic status, and the top-10 genetic PC scores.

	**M1: OLS (n = 825)**		**M2: Robust (n = 825)**	
	Estimates (95% CI)	*p*	*Estimates (95% CI)*	*p*
PCHorvath†	0.07 (-0.00 – 0.14)	0.051	0.08 (0.00 – 0.15)	**0.038**
PCHannum†	0.07 (0.01 – 0.13)	**0.032**	0.06 (0.00 – 0.13)	**0.044**
PCPhenoAge†	0.10 (0.05 – 0.16)	**<0.001**	0.11 (0.05 – 0.16)	**<0.001**
PCGrimAge†	0.10 (0.05 – 0.15)	**<0.001**	0.10 (0.05 – 0.15)	**<0.001**
DunedinPACE†	0.18 (0.12 – 0.25)	**<0.001**	0.18 (0.11 – 0.24)	**<0.001**
PCDNAmTL†	-0.06 (-0.12 – 0.00)	0.059	-0.06 (-0.12 – 0.01)	0.076

†All clocks are calculated using the principal component-based methods described by Higgins-Chen et al. 2022, except DunedinPACE for which there is no PC-based version.

Table S15. Comparison of two statistical approaches to modelling the relationship between changes in gravidity and changes in principal component (PC)-based epigenetic age (Higgins-Chen et al. 2022) in young women in the Philippines. Prior analysis, exploratory data analysis revealed four outliers (>3 SD from mean) in Horvath epigenetic age. Following a preregistered analysis plan, results are shown for ordinary least squares including (M1) and using the robust regression method defined by Yohai (1987) (M2). All models include delta age, delta pregnancy, and pregnancy status at blood draw, smoking status, an urbanicity score, and a composite score for socioeconomic status at baseline, and the top-10 genetic PC scores.

	**M1: OLS (N = 331)**		**M2: Robust (N = 331)**	
	Estimates (95% CI)	*p*	*Estimates (95% CI)*	*p*
ΔPCHorvath†	0.04 (-0.05 – 0.12)	0.398	0.06 (-0.01 – 0.13)	0.101
ΔPCHannum†	0.03 (-0.05 – 0.11)	0.467	0.06 (-0.01 – 0.12)	0.079
ΔPCPhenoAge†	0.02 (-0.05 – 0.10)	0.549	0.04 (-0.03 – 0.11)	0.306
ΔPCGrimAge†	-0.04 (-0.11 – 0.02)	0.161	-0.04 (-0.10 – 0.02)	0.233
ΔDunedinPACE†	-0.03 (-0.11 – 0.04)	0.369	-0.03 (-0.10 – 0.03)	0.329
ΔPCDNAmTL†	0.02 (-0.06 – 0.10)	0.628	-0.01 (-0.08 – 0.05)	0.660

†All clocks are calculated using the principal component-based methods described by Higgins-Chen et al. 2022, except DunedinPACE for which there is no PC-based version.

Table S16. Comparison of two statistical approaches to modelling the relationship between changes in gravidity and changes in principal component (PC)-based epigenetic age (Higgins-Chen et al. 2022) in young women in the Philippines, controlling for cell counts (CD4T, CD8T, NK, B cell, Granulocyte, and Monocyte). Prior analysis, exploratory data analysis revealed four outliers (>3 SD from mean) in Horvath epigenetic age. Following a preregistered analysis plan, results are shown for ordinary least squares including (M1) and using the robust regression method defined by Yohai (1987) (M2). All models include delta age, delta pregnancy, and pregnancy status at blood draw, smoking status, an urbanicity score, and composite score for socioeconomic status at baseline, and the top-10 genetic PC scores. Horvath epigenetic age increased 0.06 standard deviations – equivalent to 2.9 months – for each additional pregnancy. Hannum's epigenetic clock increased 0.06 standard deviations – equivalent to 2.4 months – for each additional pregnancy.

	**M1: OLS (N = 331)**		**M2: Robust (N = 331)**	
	Estimates (95% CI)	*p*	*Estimates (95% CI)*	*p*
∆PCHorvath†	0.01 (-0.07 – 0.09)	0.768	0.05 (-0.01 – 0.11)	0.121
∆PCHannum†	0.01 (-0.07 – 0.08)	0.839	0.05 (-0.01 – 0.10)	0.080
∆PCPhenoAge†	0.01 (-0.05 – 0.07)	0.774	0.03 (-0.02 – 0.08)	0.229
∆PCGrimAge†	-0.03 (-0.08 – 0.01)	0.112	-0.01 (-0.04 – 0.02)	0.493
∆DunedinPACE†	-0.05 (-0.11 – 0.02)	0.186	-0.05 (-0.11 – 0.01)	0.092
∆PCDNAmTL†	0.05 (-0.01 – 0.12)	0.099	0.03 (-0.03 – 0.08)	0.339

†All clocks are calculated using the principal component-based methods described by Higgins-Chen et al. 2022, except DunedinPACE for which there is no PC-based version.

Table S17. Comparison of two statistical approaches to modelling the relationship between whether or not a man reported fathering at least one pregnancy and principal component (PC)-based epigenetic age (Higgins-Chen et al. 2022) in a cohort of young men in the Philippines. Prior analysis, exploratory data analysis revealed six outliers (>3 SD from mean) in Horvath epigenetic age. Following a preregistered analysis plan, results are shown for ordinary least squares including (M1) and using the robust regression method defined by Yohai (1987) (M2). All models include age, number of pregnancies fathered, smoking status in 2005, an urbanicity score, a composite score for socioeconomic status, and the top-10 genetic PC scores.

	**M1: OLS (N = 910)**		**M2: Robust (N = 910)**	
	Estimates (95% CI)	*p*	*Estimates (95% CI)*	*p*
PCHorvath†	-0.07 (-0.22 – 0.09)	0.384	-0.08 (-0.24 – 0.07)	0.293
PCHannum†	-0.07 (-0.23 – 0.08)	0.340	-0.09 (-0.24 – 0.07)	0.275
PCPhenoAge†	-0.04 (-0.19 – 0.11)	0.617	-0.04 (-0.20 – 0.11)	0.594
PCGrimAge†	-0.24 (-0.39 – -0.10)	**0.001**	-0.22 (-0.36 – -0.07)	**0.003**
DunedinPACE†	-0.10 (-0.26 – 0.06)	0.216	-0.08 (-0.24 – 0.08)	0.340
PCDNAmTL†	0.03 (-0.12 – 0.18)	0.719	0.06 (-0.10 – 0.22)	0.486

†All clocks are calculated using the principal component-based methods described by Higgins-Chen et al. 2022, except DunedinPACE for which there is no PC-based version.

Table S18. Comparison of two statistical approaches to modelling the relationship between whether or not a man reported fathering at least one pregnancy and principal component (PC)-based epigenetic age (Higgins-Chen et al. 2022) in a cohort of young men in the Philippines, controlling for cell counts (CD4T, CD8T, NK, B cell, Granulocyte, and Monocyte). Prior analysis, exploratory data analysis revealed six outliers (>3 SD from mean) in Horvath epigenetic age. Following a preregistered analysis plan, results are shown for ordinary least squares including (M1) and using the robust regression method defined by Yohai (1987) (M2). All models include age, number of pregnancies fathered, smoking status in 2005, an urbanicity score, a composite score for socioeconomic status, and the top-10 genetic PC scores. Controlling for cell counts, reporting being responsible for at least one pregnancy was not associated with any DNAm measures of aging.

	**M1: OLS (N = 910)**		**M2: Robust (N = 910)**	
	Estimates (95% CI)	*p*	*Estimates (95% CI)*	*p*
PCHorvath†	-0.08 (-0.22 – 0.06)	0.252	-0.09 (-0.23 – 0.06)	0.234
PCHannum†	-0.08 (-0.20 – 0.05)	0.254	-0.08 (-0.21 – 0.05)	0.244
PCPhenoAge†	0.03 (-0.08 – 0.14)	0.592	0.04 (-0.07 – 0.16)	0.473
PCGrimAge†	-0.15 (-0.26 – -0.04)	**0.009**	-0.12 (-0.23 – -0.01)	**0.037**
DunedinPACE†	-0.08 (-0.23 – 0.06)	0.271	-0.07 (-0.22 – 0.08)	0.349
PCDNAmTL†	0.09 (-0.02 – 0.21)	0.113	0.08 (-0.04 – 0.20)	0.198

†All clocks are calculated using the principal component-based methods described by Higgins-Chen et al. 2022, except DunedinPACE for which there is no PC-based version.

Table S19. Comparison of two statistical approaches to modelling the relationship between the number of pregnancies a man fathered and principal component (PC)-based epigenetic age (Higgins-Chen et al. 2022) in a cohort of young men in the Philippines. Prior analysis, exploratory data analysis revealed six outliers (>3 SD from mean) in Horvath epigenetic age. Following a preregistered analysis plan, results are shown for ordinary least squares including (M1) and using the robust regression method defined by Yohai (1987) (M2). All models include age, number of pregnancies fathered, smoking status in 2005, an urbanicity score, a composite score for socioeconomic status, and the top-10 genetic PC scores. The number of pregnancies a man fathered was not associated with any DNAm measures of aging.

	**M1: OLS (N = 910)**		**M2: Robust (N = 910)**	
	Estimates (95% CI)	*p*	*Estimates (95% CI)*	*p*
PCHorvath†	-0.01 (-0.11 – 0.08)	0.771	-0.02 (-0.11 – 0.06)	0.566
PCHannum†	-0.00 (-0.10 – 0.09)	0.919	-0.01 (-0.10 – 0.08)	0.801
PCPhenoAge†	0.01 (-0.08 – 0.10)	0.794	0.02 (-0.07 – 0.12)	0.623
PCGrimAge†	-0.08 (-0.17 – 0.01)	0.066	-0.06 (-0.14 – 0.02)	0.121
DunedinPACE†	-0.04 (-0.13 – 0.06)	0.460	-0.02 (-0.13 – 0.08)	0.698
PCDNAmTL†	0.01 (-0.08 – 0.10)	0.879	0.02 (-0.07 – 0.11)	0.645

†All clocks are calculated using the principal component-based methods described by Higgins-Chen et al. 2022, except DunedinPACE for which there is no PC-based version.

Table S20. Comparison of two statistical approaches to modelling the relationship between the number of pregnancies a man fathered and principal component (PC)-based epigenetic age (Higgins-Chen et al. 2022) in a cohort of young men in the Philippines, controlling for cell counts (CD4T, CD8T, NK, B cell, Granulocyte, and Monocyte). Prior analysis, exploratory data analysis revealed six outliers (>3 SD from mean) in Horvath epigenetic age. Following a preregistered analysis plan, results are shown for ordinary least squares including (M1) and using the robust regression method defined by Yohai (1987) (M2). All models include age, number of pregnancies fathered, smoking status in 2005, an urbanicity score, a composite score for socioeconomic status, and the top-10 genetic PC scores. Controlling for cell counts, the number of pregnancies a man fathered was not associated with any DNAm measures of aging.

	**M1: OLS (N = 910)**		**M2: Robust (N = 910)**	
	Estimates (95% CI)	*p*	*Estimates (95% CI)*	*p*
PCHorvath†	-0.03 (-0.11 – 0.06)	0.512	-0.04 (-0.11 – 0.03)	0.291
PCHannum†	-0.02 (-0.10 – 0.06)	0.623	-0.03 (-0.09 – 0.04)	0.443
PCPhenoAge†	0.02 (-0.04 – 0.09)	0.489	0.03 (-0.04 – 0.10)	0.406
PCGrimAge†	-0.05 (-0.12 – 0.02)	0.132	-0.04 (-0.10 – 0.03)	0.255
DunedinPACE†	-0.03 (-0.12 – 0.05)	0.445	-0.02 (-0.11 – 0.08)	0.738
PCDNAmTL†	0.05 (-0.02 – 0.11)	0.187	0.05 (-0.01 – 0.11)	0.117

†All clocks are calculated using the principal component-based methods described by Higgins-Chen et al. 2022, except DunedinPACE for which there is no PC-based version.

BASELINE SURVEY

NCP - CPC - OPS

INFANT FEEDING PROJECT

IDENTIFICATION

ID1 Type of Survey [2]

ID2 Barangay: ______________________ [|]
(SEE BARANGAY CODE LIST)

ID3 Stratum: 1 - Urban
2 - Rural [ ]

ID4 Number of Household in Barangay with Pregnant/Nursing Mother: [|]

ID5 Number of Pregnant Mothers in Household: [ ]

ID6 Exact Address of Mother: ______________________

____

ID7 Number of Visit to Household: [| 1]

ID8 Name of Interviewer: ______________________ [|]

ID9 Date of Interview Completion: [|] MONTH [|] DAY [|] YEAR

CALL RECORD

CALL NO.	DATE	TIME		RESULTS (Use codes below)	APPOINTMENT MADE	
		Started	Finished		Date	Time
1						
2						
3						
4						
5						

CODE FOR RESULTS: 1 - Interview completed
2 - Interview partly completed, appointment made for continuation
3 - Appointment made for interview later
4 - Refusal, no interview obtained
5 - No respondent at home
6 - Others (SPECIFY): ______________________

ID10 Number of Sessions Required to Complete Interview: [ ]

B. HOUSEHOLD QUESTIONNAIRE

QUESTIONS ARE TO BE ANSWERED PREFERABLY BY THE WIFE OF THE HOUSEHOLD HEAD, NEVER BY A CHILD, SERVANT, BOARDER OR VISITOR.

B1 How many persons live in this household with you at present?

[ ][ ] NUMBER OF PERSONS

B2 How many of the people usually living here with you in this household are temporarily absent? IF NONE, CODE 00.

[ ][ ] NUMBER OF PERSONS ABSENT

FOR ALL PERSONS LIVING IN THE HOUSEHOLD, INCLUDING THOSE TEMPORARILY ABSENT, ASK QUESTIONS B3 THRU B9. RECORD RESPONSES IN TABLE 1. BEGIN WITH THE HEAD OF THE HOUSEHOLD, HIS SPOUSE, HIS/HER UNMARRIED CHILDREN, MARRIED CHILDREN, HELPERS, ETC.

B3 What is his(her) full name? ENTER NAME IN TABLE 1, COL. B3.

B4 How is he(she) related to the head of the household?

THE RELATIONSHIP MUST ALWAYS BE STATED FROM THE POINT OF VIEW OF THE HOUSEHOLD HEAD.

00 - household head	11 - aunt	22 - aunt-in-law
01 - spouse	12 - cousin	23 - cousin-in-law
02 - father	13 - nephew	24 - nephew-in-law
03 - mother	14 - niece	25 - niece-in-law
04 - son	15 - father-in-law	26 - other relative
05 - daughter	16 - mother-in-law	27 - yaya
06 - brother	17 - son-in-law	28 - other servant
07 - sister	18 - daughter-in-law	29 - other non-relative
08 - grandson	19 - brother-in-law	30 - pregnant woman herself
09 - granddaughter	20 - sister-in-law	
10 - uncle	21 - uncle-in-law	-8 - NR

B5 How is he(she) related to the pregnant woman in the household?

IN CASE OF TWO PREGNANT WOMEN IN THE HOUSEHOLD, RELATE PERSON TO EACH PREGNANT WOMAN. IDENTIFY EACH PREGNANT WOMAN BY HER LINE NUMBER, e.g., BROTHER OF 3, COUSIN OF 7. USE SAME CODES AS IN B4.

B6 IF NOT OBVIOUS TO INTERVIEWER, ASK

Is this person male or female?

1 - Male   2 - Female

B7 When was he(she) born?
In what month? CODE JANUARY AS 01, FEBRUARY AS 02, ETC.
In what year? CODE LAST TWO DIGITS OF YEAR

B8 Can you tell me how old he(she) was on his(her) last birthday

B9 Has he(she) resided in this household for the last six (6) months?

INFANTS BORN INTO, AND PERSONS WHO MARRIED INTO, THE HOUSEHOL ARE RESIDENTS (REGARDLESS OF DATE THEY JOINED HOUSEHOLD).

Yes ENTER CODE 1 IN COL. B9. GO TO B10.
No } CONTINUE
NR }

Does he(she) have a residence any place else?

Yes ENTER CODE 2 IN COL. B9.
No ENTER CODE 1 IN COL. B9.
NR ENTER CODE -8 IN COL. B9.

A RESIDENT IS DEFINED AS A PERSON WHO HAS LIVED IN THE HOUSEHOLD FOR THE LAST SIX MONTHS OR ONE WHO HAS LIVED IN THE HOUSEHOLD FOR LESS THAN SIX MONTHS BUT HAS NO OTHER PLACE OF USUAL RESIDENCE.

FOR ALL PERSONS LIVING IN THE HOUSEHOLD AGED SIX YEARS OLD OR OVER, ASK QUESTION B10. FOR PERSONS UNDER 6, CODE -9.

B10 What is the highest grade he(she) has completed?

00 - no grade completed	12 - one year of college
01 - one year elementary	13 - two years of college
02 - two years elementary	14 - three years of college
03 - three years elementary	15 - four years of college
04 - four years elementary	16 - five years of college
05 - five years elementary	17 - some post graduate training
06 - six years elementary	
07 - seven years elementary	18 - MA degree
08 - one year of high school	19 - more than MA degree(un[illegible] towards Ph.D., Ph.D.
09 - two years of high school	
10 - three years of high school	-8 - NR
11 - four years of high school	-9 - NA (below 6 years of a[illegible]

B11 INTERVIEWER: CODE HOUSEHOLD TYPE AFTER INTERVIEW!

B6 IF NOT OBVIOUS TO INTERVIEWER, ASK: Babaye ba kini siya o lalaki?

1 - Male 2 - Female

Table 1. Household Roster

LINE NO.	NAME	RELATION TO HOUSEHOLD HEAD		RELATION TO ~~PREGNANT~~ WOMAN				SEX	DATE OF BIRTH		AGE	RESI-DENT	HIGHEST [illegible]DE COMPLE[illegible]
				WOMAN 1		WOMAN 2							
		Description	Code	Description	Code	Description	Code		Month	Year			
	(B3)	(B4)		(B5)				(B6)	(B7)		(B8)	(B9)	(B10

B11 - Household Type: [ ]

ASK QUESTIONS B12 THRU B17 ONLY OF THE PREGNANT/NURSING MOTHER AND HER SPOUSE. ENTER CODES OF REPLIES IN APPROPRIATE COLUMNS OF TABLE 2.

BEFORE ASKING QUESTIONS, COPY LINE NO., NAME AND HIGHEST GRADE COMPLETED (CODES ONLY) OF THE PREGNANT/NURSING MOTHER AND HER SPOUSE FROM TABLE 1 INTO TABLE 2.

IF HIGHEST GRADE COMPLETED IS AT LEAST ONE YEAR OF HIGH SCHOOL (CODE 08), ASK B12; OTHERWISE GO TO B13.

B12 Did the schooling include any vocational course?

1 - Yes
0 - No
-8 - NR

B13 Did he(she) have any vocational training outside of formal schooling? (E.G., SPECIAL SEMINARS, APPRENTICESHIPS, GOVERNMENT TRAINING PROGRAMS, PRIVATE VOCATIONAL COURSES, LIKE SEWING, COSMETOLOGY, ETC.)

1 - Yes   0 - No   -8 - NR

IF ANSWER TO EITHER B12 OR B13 IS "YES", GO TO B14.
IF ANSWER TO BOTH B12 AND B13 IS "NO", CODE -9 IN B14.
IF ANSWER TO B12 AND B13 ARE A COMBINATION OF "NO" AND "NR", CODE -8 IN B14.

B14 How many months (or years) of total vocational training did he(she) have?

IF PERSON TOOK MORE THAN ONE VOCATIONAL COURSE, SUM ALL DURATIONS IN MONTHS. E.G., ONE HALF MONTH IS CODED AS .5

B15 Was he(she) born in this barangay?

1 - Yes   CODE -9 IN B16 AND B17, THEN GO TO NEXT BLOCK
0 - No
-8 - NR   } CONTINUE

B16 How many years has he(she) lived in this barangay?

USE TWO-DIGIT CODE. IF NR, CODE -8.

B17 What was his(her) last place of residence immediately before he(she) came to this barangay?

01 - Other barangay in urban Metro Cebu
02 - Other barangay in rural Metro Cebu
03 - City in Cebu Province (e.g., Toledo, Danao)
04 - Poblacion in Cebu Province
05 - Rural barangay in Cebu Province
(03–05: OUTSIDE METRO CEBU)
06 - Metro Manila
07 - Other city in other province
08 - Poblacion in other province
09 - Rural barangay in other province
-8 - NR/DK
-9 - NA

Table 2. Vocational and Migration Information

LINE NO.	NAME	HIGHEST GRADE COMPLETED (CODE ONLY)	VOCATIONAL TRAINING In School	VOCATIONAL TRAINING Out of School	TRAINING DURATION	BORN IN BARANGAY?	YEARS LIVED IN BARANGAY	PREVIOUS RESIDENCE
			(B12)	(B13)	(B14)	(B15)	(B16)	(B17)

C. ENVIRONMENTAL INFORMATION

C1 (OBSERVE!) Of what kind of material is the house constructed?

1 - Light - refers to house made of nipa or similar material
2 - Mixed - refers to house made of cement and/or wood, but with nipa or similar material either for walls or roofing
3 - Strong - refers to house made exclusively of cement and/or wood with galvanized iron roofing

---

C2 What is your usual main source of drinking water?

IF HH HAS MORE THAN ONE, ASK FOR MOST OFTEN USED SOURCE

1 - Piped, in house
2 - Pump, in house
3 - Pump, in yard
4 - Rainwater
} CODE -9 IN AND C4, GO TO C5

5 - Pump or piped, not in house or yard, for free
6 - Open well
7 - Spring, river, lake
8 - Purchased (regardless of source)
9 - Other (SPECIFY): ______
} CONTINUE

-8 - NR/DK — CODE -8 IN C3 & C4

C3 Where is this water source located?

______

GIVE EXACT LOCATION AND/OR WELL NUMBER, IF ANY. DO NOT CODE!

C4 How long does it take (in walking minutes) to get to this water source from your house?

-9 - NA (water source is located in house or yard)

C5 What kind of drinking-water container do you use for storing your drinking water?

1 - Container kept in refrigerator
2 - Water tank (closed)
3 - Earthen Jar, with faucet
4 - Earthen Jar, without faucet
5 - Drum/Banyera (open)
6 - Can (tin)
7 - Plastic container with faucet
8 - Plastic container without faucet
9 - Bottle
10 - Other (SPECIFY): ______
11 - Combination (SPECIFY CODES): ______
-8 - NR
-9 - NA (does not store drinking water)

C6 Is the drinking-water container with cover or without cover? (OBSERVE IF POSSIBLE)

1 - with cover
2 - without cover
3 - some with, some without cover

C7 What kind of container do you usually use for fetching or buying drinking water?

1 - taro (kerosene can)
2 - gallonan (plastic container), either round or square
3 - metal pail
4 - plastic pail
5 - drum
6 - other (SPECIFY, including approximate capacity): ______

-8 - NR
-9 - NA (does not fetch or buy water) GO TO C11

☐

C8 How many of these containers do you fetch or buy per day (DRINKING WATER ONLY!)?

☐☐

C9 What kind of container do you usually use for fetching or buying other (non-drinking) water?

USE SAME CODES AS IN C7

☐

C10 How many of these containers do you fetch or buy per day (NON-DRINKING WATER ONLY!)?

☐☐

C11 What kind of toilet facilities do you have?

1 - Flush, inside house
2 - Flush, outside house
3 - Water sealed, inside house
4 - Water sealed, outside house, private
5 - Water sealed, outside house, public
6 - Antipolo
7 - Open pit
8 - None, go to open field, river, etc.)
9 - Other (SPECIFY): ______
-8 - NR

☐

C12 (OBSERVE!) What is the general condition of the area around the house with respect to excreta removal? Is the house smelling?

1 - Heavy defecation in area
2 - Some defecation in area
3 - Very little excreta visible
4 - No excreta visible
-8 - NR

☐

C13 What is your main method of garbage disposal?

1 - Collected by a garbage collector
2 - Burning
3 - Composting (decaying fertilizer)
4 - Dumping around or near house
5 - Dumping in specified place far from house
6 - Other (SPECIFY): ______
7 - Combination (SPECIFY) CODES): ______
-8 - NR/DK

☐

C14 (OBSERVE!) Is the area in the house where the food is kept

1 - Very clean
2 - Not so clean
3 - Filthy
-8 - NR

C15 What type of lighting do you use most of the time?

1 - Electric
2 - Kerosene
3 - Oil
4 - LPG (Gasul)
5 - Candle
6 - Other (SPECIFY): ____________
-8 - NR

C16 What fuel do you usually use for cooking?

1 - Electricity
2 - Kerosene
3 - LPG (Gasul)
4 - Wood
5 - Charcoal
6 - Other (SPECIFY): ____________
7 - Combination (LIST CODES) ____________
-8 - NR

C17 What brand (kind) of soap do most members of your household usually use for <u>bathing</u>?

01 - Perla, blue
02 - Perla, white
03 - Superwheel, white
04 - Superwheel, blue
05 - Wheel, blue
06 - Wheel, white
07 - Mr. Clean, blue
08 - Mr. Clean, white
09 - Ajax, blue
10 - Ajax, white
11 - Tide (bar), blue
12 - Tide (bar), white
13 - Argo bar
14 - Powdered soap (e.g., Tide, Breeze, Drive)
15 - Home-made soap
16 - Toilet soap (e.g. Lux, Camay, Palmolive, etc.)
17 - Medicinal soap
18 - Liquid bathing soap
19 - Tops
20 - Champion
21 - Other (SPECIFY) ____________
-8 - NR

C18 How much (IN PESOS) does your household usually spend for bathing soap in one week?

USE THREE-DIGIT CODE. CODE CENTAVOS, e.g., 150 FOR ₱ 1.50

CODE NR = -8

C19 Is this soap also used for washing of clothes?

1 - Yes
0 - No
-8 - NR

C20 Do you keep reading materials in your house, either bought or borrowed?

1 - Yes
0 - No } GO TO C22
-8 - NR

[ ]

C21 Who in your household reads these materials?

CODE ALL PERSONS/GROUPS MENTIONED

1 - Yes
0 - No
-8 - DK
-9 - NA (No reading materials in household)

Pregnant woman	[ ]
Spouse of pregnant woman	[ ]
Children	[ ]
Relatives/Grandparents	[ ]
______ Other (SPECIFY)	[ ]

C22 What language is usually spoken in your house? [ ]

C23 What language was usually spoken in the house of your parents when you grew up? [ ]

C24 What language was usually spoken in the house of your husband when he grew up? [ ]

CODES FOR C22, C23, C24

1 - Cebuano
2 - Tagalog/Pilipino
3 - Ilongo
4 - Waray
5 - English
6 - Chinese
7 - Spanish
8 - Other (SPECIFY): ______
-8 - NR
-9 - NA (No husband present)

ASK OF PREGNANT/NURSING MOTHER ONLY!!

C25 What is your religion? [ ]

C26 What is your husband's religion? [ ]

CODES FOR C25, C26

1 - Catholic
2 - Iglesia ni Cristo
3 - Seventh Day Adventist
4 - Mormon
5 - Other Protestant
6 - Muslim
7 - Buddhist
8 - Other Non-christian (SPECIFY): ______
-8 - NR/DK
-9 - NA (No husband present)

C27 How far (in meters or kilometers) is your house from the nearest road which is being used by public or private vehicles?

0 - house is by the roadside
-8 - DK } GO TO C30
-9 - NA (no road on island) } GO TO C30

CODE IN METERS. 1 KILOMETER = 1000 METERS [ ][ ][ ].[ ]

C28 How long (in minutes) does it take to walk from your house to this road?

-8 - DK
-9 - NA (house is by the roadside) [ ][ ][ ]

C29 What types of vehicles ordinarily pass along this road?

1 - Yes
0 - No
-8 - DK
-9 - NA (no road on island)

Tartanillas [ ]
Trucks [ ]
Buses/minibuses [ ]
Jeepneys [ ]
Passenger cars, e.g., taxis, PUs [ ]
Motorized tricycles [ ]
Pedal-operated tricycles [ ]
Private cars [ ]
______________ Other (SPECIFY): [ ]

C30 Do you know of any milk for the infant besides breastmilk?

1 - Yes CONTINUE
0 - No } GO TO C38
-8 - DK/NR } GO TO C38
[ ]

C31 What kind or brand of infant food do you know?

INTERVIEWER: MARK ALL TYPES MOTHER MENTIONS WITH "1"

1 - Yes
0 - No
-8 - DK
-9 - NA

Fresh milk [ ]
Other milk, canned or powdered [ ]
S-26 [ ]
SMA [ ]
Bonna [ ]
Enfamil [ ]
Similac [ ]
Sobee/Isomil [ ]
Enfalac [ ]
Al 110 [ ]
Nestogen [ ]
Lactogen [ ]
Pelargon [ ]
Alacta [ ]
Others (SPECIFY): [ ]
____

ENTER ANSWERS TO QUESTIONS C32 THRU C37 IN TABLE 2B BELOW.
IF ANSWER IS "DON'T KNOW", CODE -8.

C32 How far (in walking minutes) is it from your house to the nearest store that sells infant formula like Enfamil, Similac or others?

ENTER RESPONSE IN MINUTES IN COL. C32 OF TABLE 2B.

-9 - NA (too far for walking)

C33 How much does it cost when you take transportation to go to that store?

USE THREE-DIGIT CODE. CODE CENTAVOS, e.g., 150 FOR ₱ 1.50.
ENTER CODE IN COL. C33 OF TABLE 2B.

-9 - NA (too close for transportation)

C34 How far (in walking minutes) is it from your house to the nearest store that sells canned or powdered milk?

ENTER RESPONSE IN COL. C34 OF TABLE 2B.

C35 How much does it cost when you take transportation to go to that store?

USE THREE-DIGIT CODE. CODE CENTAVOS, e.g., 150 FOR ₱ 1.50.
ENTER CODE IN COL. C35 OF TABLE 2B.

-9 - NA (too close for transportation)

C36 How far (in walking minutes) is it from your house to the nearest store that sells infant food like Cerelac, Oatmeal or others?

ENTER RESPONSE IN COL. C36 OF TABLE 2B.

C37 How much does it cost when you take transportation to go to that store?

USE THREE-DIGIT CODE. CODE CENTAVOS, e.g., 150 FOR ₱ 1.50.
ENTER CODE IN COL. C37 OF TABLE 2B.

-9 - NA (too close for transportation)

Table 2B. Store Selling Infant Food

TYPE OF FOOD	WALKING MINUTES TO STORE SELLING (C32/34/36)	TRANSPORTATION COSTS TO NEAREST STORE SELLING (C33/35/37)
Infant Formula		
Milk (canned or powdered)		
Other Infant Food		

C38 Where do you usually shop for food?

LIST STORE OR MARKET: ______________________

C39 Does this store sell?

1 - Yes
0 - No
-8 - NR/DK

Infant formula ☐

Milk (canned or powdered) ☐

Other infant food ☐

D. HOUSEHOLD ASSETS

I WILL NAME NOW A NUMBER OF HOUSEHOLD ASSETS. WOULD YOU BE SO KIND AND TELL ME WHETHER YOUR HOUSEHOLD OWNS THEM OR NOT. IN CASE YOUR HOUSEHOLD DOES OWN THEM, BE SO GOOD AND GIVE ME AN ESTIMATE OF THEIR CURRENT VALUE.

D1 Do you or your household own this house in which you are living?

1 - Yes
0 - No
-8 - NR

D2 Do you or your household own any other residential houses?

1 - Yes
0 - No
-8 - NR

IF ANSWER TO BOTH D1 AND D2 IS "NO" (CODE 0), ENTER -9 IN D3 AND CONTINUE WITH D4. IF ANSWER TO EITHER D1 OR D2 IS "DK" (CODE -8), ENTER -8 IN D3 AND CONTINUE WITH D4.

D3 What is the estimated current total value (in Pesos) of all the houses that you and/or your household own?

ALTERNATE QUESTION: If you were to sell your houses, how much (in Pesos) would you get from them?

-8 - DK
-9 - NA (Owns no house)

IF ANSWER TO D1 IS "YES" (CODE 1), ENTER -9 IN D4, D5 AND D6 AND CONTINUE WITH D7.

D4 Does your household rent this house or stay in it for free?

1 - Rents CONTINUE
2 - Stay for free CODE -9 IN D5, CONTINUE WITH D6
-8 - DK
-9 - NA

D5 How much (in Pesos) per month do you pay for renting this house?

-8 - DK
-9 - NA

CODE -9 IN D6, CONTINUE WITH D7

D6 If you were to rent this house, how much (in Pesos) do you think you would have to pay for this house every month?

-8 - DK
-9 - NA

D7 Excluding the bathroom and toilet, how many rooms do you have in this house?

CODE NUMBER OF ROOMS

D8 Do you or your household own any land, including the lot on which your house is built?

1 - Yes CONTINUE
0 - No CODE -9 IN D9, CONTINUE WITH D10
-8 - NR CODE -8 IN D9, CONTINUE WITH D10

D9 What is the estimated current total value (in Pesos) of all your landholdings?

ALTERNATE QUESTION: If you were to sell all your landholdings, how much (in Pesos) would you get for them?

-8 - DK
-9 - NA (owns no landholding)

D10 Do you or your household own any of the following vehicles?

1 - Yes
0 - No
-8 - DK

IF ALL ANSWERS ARE "NO" (CODE "0"), CODE -9 IN D11 AND CONTINUE WITH D12

Carabao cart

Push cart

Bicycle

Tricycle

Jeepney

Truck/Bus

Car

Other (SPECIFY) ____________

D11 What is the estimated current total value (in Pesos) of all your vehicles?

ALTERNATE QUESTION: If you were to sell all your vehicle(s), how much (in Pesos) would you get for them?

-8 - DK
-9 - NA (owns no vehicle)

D12 Do you or your household own any of the following livestock?

1 - Yes
0 - No
-8 - DK

IF ALL ANSWERS ARE "NO" (CODE "O"), CODE -9 IN D13 AND CONTINUE WITH D14

Poultry [ ]

Pigs [ ]

Goats [ ]

Cows [ ]

Carabaos [ ]

________________ Other (SPECIFY) [ ]

D13 What is the estimated total value (in Pesos) of all your livestock?

ALTERNATE QUESTION: If you were to sell all your livestock, how much (in Pesos) would you get for it?

-8 - DK
-9 - NA (owns no livestock)

[ ][ ][ ][ ][ ]

D14 Do you or your household own any of the following kinds of equipment?

1 - Yes
0 - No
-8 - DK

1. Agricultural Equipment

Plow [ ]

Grain Drier [ ]

Thresher [ ]

Rice/Corn Mill [ ]

________________ Other (SPECIFY) [ ]

1 - Yes
0 - No
-8 - DK

2. Fishing Equipment

Nets [ ]

Banca/Boat [ ]

Marine engine [ ]

Pressure lamp [ ]

________________ Other (SPECIFY) [ ]

1 - Yes
0 - No
-8 - DK

3. Business/Profession-related Equipment

Sewing machine [ ]

Beauticians kit [ ]

Grinder [ ]

Welding tools [ ]

Clerical equipment [ ]

________________ Other (SPECIFY) [ ]

D15 What is the estimated current total value (in Pesos) of all your agricultural and/or fishing and/or business/profession-related equipment?

ALTERNATE QUESTION: If you were to sell all your agricultural and/or fishing and/or business/profession-related equipment, how much (in Pesos) would you get for it?

-8 - DK
-9 - NA (owns no equipment)

[ ][ ][ ][ ][ ]

D16 Do you or your household own any of the following pieces of furniture?

1 - Yes
0 - No
-8 - DK

IF ALL ANSWERS ARE "NO" (CODE "0"), CODE -9 IN D17 AND CONTINUE WITH D18

Bench/Chairs [ ]

Sala Set [ ]

Table [ ]

Dining Set [ ]

Aparador(s) [ ]

Bed(s) [ ]

____________ Other (SPECIFY) [ ]

D17 What is the estimated current total value (in Pesos) of all your pieces of furniture?

ALTERNATE QUESTION: If you were to sell all your pieces of furniture, how much (in Pesos) would you get for them?

-8 - DK
-9 - NA (owns no furniture)

[ ][ ][ ][ ][ ]

D18 Do you or your household own any of the following household appliances?

1 - Yes
0 - No
-8 - DK

IF ALL ANSWERS ARE "NO" (CODE "0"), CODE -9 IN D19 AND CONTINUE WITH D20

Radio [ ]

Electric iron [ ]

Flat iron [ ]

Stereo/tape recorder [ ]

TV [ ]

Vacuum cleaner [ ]

Refrigerator [ ]

Air conditioner [ ]

Electric Fan [ ]

____________ Other (SPECIFY) [ ]

D19 What is the estimated current total value (in Pesos) of all your household appliances?

ALTERNATE QUESTION: If you were to sell your household appliances, how much (in Pesos) would you get for them?

-8 - DK
-9 - NA (owns no appliances)

D20 Do you or your household own any of the following kitchen equipment?

1 - Yes
0 - No
-8 - DK

IF ALL ANSWERS ARE "NO" (CODE "0"), CODE -9 IN D21 AND CONTINUE WITH D27

Clay pots and pans
Metal pots and pans
Clay stove
Kerosene stove
Electric/gas stove or range
Silverware (e.g. spoons, fork, etc.)
__________ Other (SPECIFY)

D21 What is the estimated current total value (in Pesos) of all your kitchen equipment?

ALTERNATE QUESTION: If you were to sell all your kitchen equipment, how much (in Pesos) would you get for it?

-8 - DK
-9 - NA (owns no kitchen equipment)

D22 Do you or your household own any of the following utensils which are related to baby food preparation and storage?

1 - Yes
0 - No
-8 - DK

IF ALL ANSWERS ARE "NO" (CODE "0"), CODE -9 IN D23 AND CONTINUE WITH NEXT BLOCK

Thermos bottle
Feeding bottles
Bottle brush
Measuring spoon/cup
Sterilizer
Grinder/Blender
__________ Other (SPECIFY)

D23 What is the estimated current total value (in Pesos) of all your utensils related to baby food preparation and storage?

ALTERNATE QUESTION: If you were to sell all your utensils related to baby food preparation and storage, how much (in Pesos) would you get for them?

-8 - DK
-9 - NA (owns no such utensils)

E1. MARKET ACTIVITIES OF RESIDENT HOUSEHOLD MEMBERS

BEFORE STARTING WITH BLOCK E, COPY ALL LINE NUMBERS AND NAMES OF RESIDENT HOUSEHOLD MEMBERS AGED 6 OR OLDER FROM TABLE 1 ENTER THESE IN THE COLUMNS PROVIDED FOR THIS PURPOSE IN TABLE 3A. ASK E1 AND E2 OF ALL PERSONS LISTED IN TABLE 3A. ENTE CODES ONLY IN TABLE 3A.

E1 Does he(she) currently do any work for pay?

WORK IS DEFINED AS ANY GAINFUL EMPLOYMENT WHICH INCLUDES ANY ACTIVITY FOR WHICH ONE RECEIVES REMUNERATION IN EITHER CASH OR KIND.

1 - Yes — GO TO E3
0 - No
-8 - NR } CONTINUE

E2 Did he(she) work at any time during the past four months?

1 - Yes — CONTINUE
0 - No
-8 - DK } ASK NEXT ELIGIBLE PERSON. BEGIN WITH E1.

E3 What is(was) his(her) main job?

MAIN JOB IS THAT JOB ON WHICH A PERSON SPENDS THE MOST TIME.

1 - Farming — CONTINUE
2 - Fishing — CODE -9 IN COL. E4, THEN GO TO E5
3 - Other — CODE -9 IN COL. E4, THEN GO TO E5

E4 In what capacity does(did) he(she) work?

1 - Farm owner	5 - Unpaid family worker
2 - Farm tenant	6 - Other
3 - Both owner & tenant	-8 - DK/NR
4 - Paid farm laborer	-9 - NA

IF THE FARM IS OWNED BY THE FAMILY/HOUSEHOLD, ONLY ONE MEMBER, USUALLY THE HOUSEHOLD HEAD, CAN WORK AS FARM OWNER. ALL OTHER HOUSEHOLD MEMBERS WORKING ON THE FAMILY FARM ARE EITHER PAID FARM LABORERS OR UNPAID FAMILY WORKERS. IF THE FAMILY/HOUSEHOLD OPERATES THE FARM AS TENANT, THEN ALL FAMILY HOUSEHOLD MEMBERS WORKING ON THAT FARM ARE TENANTS.

ENTER CODE IN COL. E4. CODE -9 IN COL. E5. GO TO E6.

E6 During the work week preceding this interview, how many hours did he(she) spend on this primary job?

ENTER NUMBER OF HOURS DURING WEEK IN COL. E6.
-8 - NR
-9 - NA (Did not work last week)

E7 Does he(she) currently perform a secondary job for additional pay in either cash or kind?

A SECONDARY JOB IS A JOB ON WHICH A PERSON SPENDS TIME EITHER (a) AFTER ATTENDING TO HIS(HER) MAIN JOB, OR (b) FOR A SHORTER DURATION, OR A COMBINATION OF BOTH.

1 - Yes — CONTINUE
0 - No
-8 - NR } ASK NEXT ELIGIBLE PERSON. BEGIN WITH E1.

E8 What is his(her) secondary job?

ENTER CODE IN COL. E8.

1 - Farming — CONTINUE
2 - Fishing — CODE -9 IN COL. E9, THEN GO TO E10
3 - Other — CODE -9 IN COL. E9, THEN GO TO E10

E9 In what capacity does(did) he(she) work on the secondary job?

1 - Farm owner	5 - Unpaid family worker
2 - Farm tenant	6 - Other
3 - Both owner & tenant	-8 - DK/NR
4 - Paid farm laborer	-9 - NA

ENTER CODE IN COL. E9. CODE -9 IN COL. E10, THEN ASK NEXT ELIGIBLE PERSON.

FOR THOSE PERSONS ENGAGED IN ACTIVITIES OTHER THAN FARMING

E5 What is(was) his(her) employment status?

ENTER CODE IN COL. E5, THEN CONTINUE

1 - Self-employed
2 - Wage/salary worker
3 - Paid worker in family-owned enterprise
4 - Unpaid worker in family owned enterprise
5 - Other
-8 - DK/NR
-9 - NA

IF THE FAMILY OWNS A PRIVATE BUSINESS OR DOES FISHING, ONLY ONE FAMILY MEMBER, USUALLY THE HEAD, CAN BE CLASSIFIED AS SELF-EMPLOYED. OTHER FAMILY MEMBERS WORKING IN THE FAMILY BUSINESS ARE EITHER PAID FAMILY WORKERS IF THEY RECEIVE WAGES, OR UNPAID FAMILY WORKERS. INDEPENDENTLY WORKING PROFESSIONALS (DOCTORS, LAWYERS, ENGINEERS) ARE SELF-EMPLOYED.

E10 What is his(her) employment status on the secondary

ENTER CODE IN COL. E10

1 - Self-employed
2 - Wage/salary worker
3 - Paid worker in family-owned enterprise
4 - Unpaid worker in owned enterprise
5 - Other
-8 - NR/DK
-9 - NA

E11 During the work <u>week</u> preceding this interview, how many hours did he(she) spend on this secondary job?

ENTER NUMBER OF HOURS DURING WEEK IN COL. E11. DK = -8
-9 - NA ~~(Did not work last week)~~

Table 3A. Record of Gainful Activities: Main and Secondary Jobs

LINE NO.	NAME	MAIN JOB						SECONDARY JOB				
		Has Job Now	Had Job Past 4 Months	Job Description	Farming Status	Status of Non-Farm Job	Hours Worked Past Week	Having Secondary Job Now	Job Description	Farming Status	Status of Non-Farm Job	Hours Worked Past Week
		(E1)	(E2)	(E3)	(E4)	(E5)	(E6)	(E7)	(E8)	(E9)	(E10)	(E11)

E2. OTHER ACTIVITIES OF ALL PERSONS CURRENTLY LIVING IN THE HOUSEHOLD

FOR ALL PERSONS LIVING IN THE HOUSEHOLD AGED 6 OR OLDER, INCLUDING NON-RESIDENTS, COPY NAMES AND LINE NUMBER FROM TABLE 1 INTO TABLE 3B. THEN ASK QUESTIONS E12-E31 FOR ALL OF THESE PERSONS AND ENTER RESPONSES IN TABLE 3B. CODE TIME IN MINUTES, USING THREE-DIGIT CODES, e.g., 090 FOR ONE HOUR AND A HALF.

E12 Who did the marketing for food in the household during the last week? MAKE CHECKMARK IN COL. E12 AFTER ALL PERSONS MENTIONED.

E13 How many minutes last week were spent on marketing for food by (ASK FOR ALL PERSONS MENTIONED, i.e., WITH CHECKMARK IN COL. E12)? RECORD MINUTES IN COL. E13.

E14 Who did the washing of dishes and/or other utensils in the household during the last week? CHECK PERSONS IN COL. E14.

E15 How many minutes last week were spent on washing dishes and/or other utensils by (PERSONS WITH CHECKMARK IN COL. E14)? RECORD MINUTES IN COL. E15.

E16 Who cleaned the house/backyard during the last week? CHECK PERSONS IN COL. E16.

E17 How many minutes last week were spent on cleaning the house/backyard by (PERSONS WITH CHECKMARK IN COL. E16)? RECORD MINUTES IN COL. E17.

E18 Who prepared and cooked food in the household during the last week? CHECK PERSONS IN COL. E18.

E19 How many minutes last week were spent on preparing and cooking food by (PERSONS WITH CHECKMARK IN COL. E18)? RECORD MINUTES IN COL. E19.

E20 Who washed and ironed clothes in the household during the last week? CHECK PERSONS IN COL. E20.

E21 How many minutes last week were spent on washing and ironing clothes by (PERSONS WITH CHECKMARK IN COL. E20) RECORD MINUTES IN COL. E21.

E22 Who fetched water and gathered/chopped firewood in the household during the last week? CHECK PERSONS IN COL. E22.

E23 How many minutes last week were spent on fetching water and gathering/chopping firewood by (PERSONS WITH CHECKMARK IN COL. E22)? RECORD MINUTES IN COL. E23.

E24 Who mended, sewed or repaired clothes in the household during the last week? CHECK PERSONS IN COL. E24.

E25 How many minutes last week were spent on mending, sewing and repairing clothes by (PERSONS WITH CHECKMARK IN COL. E24)? RECORD MINUTES IN COL. E25.

E26 Who cared for the children, aged 0-5 years old in the household during the last week? CHECK PERSONS IN COL. E26.

E27 How many minutes last week were spent by (PERSONS WITH CHECKMARK IN COL. E26) in caring for the children aged 0-5 years, i.e., by doing any or all of the following: Feeding, Bathing and Dressing, Cuddling/Watching.

E28 Who was involved on handicraft/home repair during the last week? CHECK PERSONS IN COL. E28.

E29 How many minutes last week were spent in handicraft/home repa[illegible] by (PERSONS WITH CHECKMARK IN COL. E28)? RECORD MINUTES IN COL. E29.

E30 Who was involved in other activities not mentioned? ASK WHAT ACTIVITIES THESE WERE AND CHECK PERSONS IN COL. E30.

E31 How many minutes last week were spent on this particular activity by (PERSONS WITH CHECKMARK IN COL. E31)? RECORD MINUTES IN COL. E33.

Table 3B. Time Allocation for Other Activities of Household Members During Work Week Immediately Preceeding Interview

LINE NO.	NAME	MARKETING (FOOD)		WASHING DISHES/ UTENSILS		CLEANING HOUSE & BACKYARD		COOKING & PREPARING FOOD		WASHING IRONING CLOTHES		GETTING WATER & FIREWOOD		MENDING SEWING CLOTHES		CARE OF CHILDREN UNDER 6		HANDICRAFT AT HOME		OTHER ACTIVITIES MENTIONED	
			TIME		TIME		TIME		TIME		TIME		TIME		TIME		TIME		TIME		TIME
		(E12)	(E13)	(E14)	(E15)	(E16)	(E17)	(E18)	(E19)	(E20)	(E21)	(E22)	(E23)	(E24)	(E25)	(E26)	(E27)	(E28)	(E29)	(E30)	(E31)

ASK OF EXPECTANT MOTHER ONLY

E32 Do you ever buy ready-cooked dishes?

1 - Yes CONTINUE
0 - No CODE -9 IN E33 AND E34, THEN GO TO SECTION F
-8 - NR/DK CODE -8 IN E33 AND E34, THEN GO TO SECTION F

E33 Where do you buy ready-cooked dishes?

Place (NAME OF STORE/MARKET): ____________________

E34 How often do you buy ready-cooked dishes?

1 - Once a day
2 - Once a week
3 - Twice a week
4 - Thrice or more per week
5 - Seldom, only on special occasions
6 - Other
-8 - DK/NR
-9 - NA

F. INCOME

INTERVIEWER: USE THE FOLLOWING GUIDELINES TO DETERMINE:

(A) WHETHER A PERSON HOLDING TWO JOBS HAS JOBS OF THE SAME OR OF DIFFERENT TYPES, AND

(B) THE INCOME TABLE INTO WHICH THE WORKING TIME AND THE INCOME HAVE TO BE ENTERED.

1. Copy into the scratch table on the following page the pertinent information from Table 3A.

2. For each person listed in the scratch table, determine whether he/she is holding one (main) or two (main and secondary) jobs.

3. FOR PERSONS HOLDING ONE (MAIN) JOB ONLY:

   (a) From the scratch table, obtain his/her job status, which is listed in col. E4 or E5. Then locate this same status in the table below. The last column of the row, in which the job status is found, indicates the income table that is to be used.

   (b) In case a person is SELF-EMPLOYED or an UNPAID FAMILY WORKER, check the code in col. E3 of the scratch table to determine the correct row in the table below.

4. FOR PERSONS HOLDING TWO (MAIN AND SECONDARY) JOBS:

   (a) Determine first whether both jobs are of the same or of different types. For this purpose, compare the job status in col. E4 (or E5) with that in col. E9 (or E10). In case of SELF-EMPLOYMENT and UNPAID FAMILY WORK, take into account also the codes in cols. E3 and E8. If the two jobs are found in the same row of the table below, then they are of the same type; if they are listed in two different rows, they are of different types.

   (b) If the two jobs are of the same type, add up the time spent on, and the income earned from, both jobs and enter the cumulative answers in that income table which is indicated in the table below in the last column of the row in which the two jobs are listed.

   (c) If the two jobs are of different types, treat each job separately. Determine, in the manner described under 3, first the income table into which information for the first (main) job has to be entered, and then the income table for the secondary job.

ROW NO.	CODES IN COLUMN OF TABLE 3A			ENTER IN TABLE
	E3/E8	E4/E9	E5/E10	
1	DOES NOT MATTER	4 - Paid Farm Laborer<br>6 - Other Farm Worker	2 - Wage/Salary Worker<br>3 - Paid Worker in Family-owned Enterprise<br>5 - Other Type of Worker	4 or 5
2	1 - Farming	1 - Farm Owner<br>2 - Farm Tenant<br>3 - Both Owner & Tenant<br>5 - Unpaid Family Worker		6A
3	2 - Fishing		1 - Self-employed<br>4 - Unpaid Worker in Family-owned Enterprise	7
4	3 - Other		1 - Self-employed<br>4 - Unpaid Worker in Family-owned Enterprise	8

COPY INTO THE SCRATCH TABLE BELOW ALL INFORMATION CONTAINED IN COLUMNS E3, E4, E5, E6, E9 AND E10 OF TABLE 3A. USE TABLE BELOW AS GUIDE FOR ALL OF YOUR INCOME QUESTIONS.

LINE NO.	NAME	MAIN JOB				SECONDARY JOB			
		Job Description (E3)	Farming Status (E4)	Status of Non-Farm Job (E5)	Job Type	Job Description (E8)	Farming Status (E9)	Status of Non-Farm Job (E10)	Job Type

I. INCOME DERIVED FROM WAGE LABOR

FROM THE TABLE ON THE PREVIOUS PAGE(SCRATCH TABLE), THE RECORD OF MARKET ACTIVITIES, COPY LINE NUMBERS AND NAMES OF ALL RESIDENT HOUSEHOLD MEMBERS WHO ARE OR WERE ENGAGED, OVER THE PAST FOUR MONTHS, IN MARKET ACTIVITIES FOR WHICH THEY RECEIVED A WAGE OR SALARY IN EITHER CASH OR KIND. PAID FARM LABORERS AND FISHERMEN HIRED BY OTHERS AND WORKING FOR PAY ARE WAGE LABORERS! WAGE LABORERS ARE ALL THOSE WITH A CODE OF "4" OR "6" IN COL. E4 AND/OR E9 OR CODES OF "2", "3" AND "5" IN COL. E5 AND/OR COL. E10 IN THE SCRATCH TABLE. IF A PERSON HAS TWO WAGE/SALARY JOBS, ENTER HOURS WORKED AND WAGES RECEIVED FOR BOTH JOBS COMBINED, PROVIDED BOTH JOBS ARE EITHER WAGE-FOR-TIME OR WAGE-FOR-PIECE JOBS. IF TYPES OF JOBS ARE DIFFERENT, ENTER INFORMATION FOR EACH JOB IN APPROPRIATE TABLE. BEFORE ENTERING THE NAMES IN EITHER TABLE 4 OR TABLE 5, ASK QUESTION F1.

F1 Is(was he(she) paid on a TIME basis or PER PIECE basis?

IF TIME BASIS, ENTER LINE NUMBER AND NAME IN TABLE 4 AND ASK F2 TO F4.
IF ON PER PIECE BASIS, ENTER LINE NUMBER AND NAME IN TABLE 5 AND ASK F5 TO F8.

F2 On the average, how many hours does(did) he(she) work during a usual work day?
(ENTER RESPONSE IN COL. F2 OF TABLE 4)

F3 How many days does(did) he(she) work in a usual week?
(ENTER RESPONSE IN COL. F3 OF TABLE 4)

F4 How much (IN PESOS) does(did) he(she) normally receive for a usual day's work, including allowances?

IF PERSON HAS A SECONDARY JOB WHICH IS DIFFERENT FROM THE MAIN JOB, GO TO APPROPRIATE QUESTIONS. IF PERSON HAS ONLY ONE JOB, GO TO NEXT PERSON OR, AFTER LAST PERSON, GO TO F21.

F5 How many pieces of (CLOTHES, SHELL NECKLACE, BRACELETS, LAUNDRY, ETC.) does he(she) finish during a usual day?
(ENTER RESPONSE IN COL. F5 OF TABLE 5)

F6 How much (IN PESOS) is(was) he(she) paid per finished item?
(ENTER RESPONSE IN F6 OF TABLE 5)

F7 On the average, how many hours per day does(did) he(she) engage in piece labor? (ENTER RESPONSE IN COL. F7 OF TABLE 5)

F8 On the average, how many days per week does(did) he(she) engage in piece labor? (ENTER RESPONSE IN COL. F8 OF TABLE 5)

IF PERSON HAS A SECONDARY JOB WHICH IS DIFFERENT FROM THE MAIN JOB, GO TO APPROPRIATE QUESTIONS. IF PERSON HAS ONLY ONE JOB, GO TO NEXT PERSON OR, AFTER LAST PERSON, TO F21.

Table 4. Income Derived from Wage Labor on a Time Basis

LINE NO.	NAME	AVE. NO. HOURS WORKED PER DAY (F2)	AVE. NO. DAYS PER WEEK OF WORK (F3)	PESOS RECEIVED PER DAY'S WORK (F4)

Table 5. Income Derived from Labor Paid per Piece

LINE NO.	NAME	AVE. NO. OF FINISHED ITEMS PER DAY (F5)	PESOS RECEIVED PER ITEM (F6)	AVE. NO. OF HOURS PER DAY (F7)	AVE. NO. OF DAYS WORKED PER WEEK (F8)

II. INCOME DERIVED FROM FARMING ACTIVITIES (EXCLUDING INCOME FROM LIVESTOCK RAISING)

FROM THE SCRATCH TABLE, THE RECORD OF MARKET ACTIVITIES, COPY INTO TABLE 6A THE LINE NUMBERS AND NAMES OF ALL HOUSEHOLD WHO HAVE OR HAD FARM JOBS. THESE ARE ONLY THOSE PERSONS WITH A CODE OF "1" IN COL. E3 AND/OR E8 AND WITH CODES "1", "2", "3" OR "5" IN COL. E4 AND/OR E9. FOR ALL OF THESE PERSONS, ASK QUESTIONS F9 AND F10.

F9 On an average working day, how many hours a day does he/she usually work on the farm?
(ENTER NUMBER OF HOURS PER DAY IN COL. F9 OF TABLE 6A.

-8 - NR/DK   -9 - NA

F10 In an average week, how many days does he/she usually work on the farm?
(ENTER NUMBER OF DAYS PER WEEK IN COL. F10 OF TABLE 6A)

IF THE FAMILY OPERATES THE FARM AS A FAMILY FARM OR AS TENANTS, ASK QUESTIONS F11 THRU F20 ONLY OF THE HEAD OR THE ELDEST MEMBER OF THAT FAMILY. IF A FAMILY OR HOUSEHOLD MEMBER CULTIVATES ANOTHER FARM INDEPENDENTLY OF THE FAMILY, ASK ALL QUESTIONS OF THIS MEMBER.

F11 How many parcels of land did he/she cultivate in 1982?
(ENTER RESPONSE IN COL. F11 OF TABLE 6A)

-8 - DK

F12 How large are all parcels taken together?

IF R ANSWERS IN TERMS OF SQUARE METERS, CONVERT TO HECTARES (1 HECTARE = 10,000 SQ. METERS), RECORD RESPONSE IN COL. F12 OF TABLE 6A. THEN GO TO F14. IF R DOES NOT KNOW THE ANSWER IN SQUARE METERS OR HECTARES, ASK F13.

F13 If he/she were to plant corn, how many chupas or gantas of seedlings would he/she need for planting each parcel?

Parcel No. 1 ________   Parcel No. 4 ________
Parcel No. 2 ________   -8 - NR/DK
Parcel No. 3 ________   -9 - NA

ADD UP TOTAL NUMBER OF CHUPAS OR GANTAS OF CORN SEEDLINGS USED. 1 GANTA IS EQUAL TO 6 CHUPAS. A HECTARE OF FLAT LAND CAN BE PLANTED WITH 4 GANTAS OF CORN SEEDLINGS, A HECTARE OF SLOPING LAND CAN BE PLANTED WITH 5 GANTAS OF CORN SEEDLINGS. USING THESE MEASURES AS GUIDE, CONVERT R'S RESPONSE INTO SQUARE METERS OR HECTARES AND ENTER RESULT IN TERMS OF SQUARE METERS '1 HA = 10,000 SQ. METERS' IN COL. F12/F13

F14 What were the major crops that he/she planted in 1982?
(ENTER RESPONSE IN COL. F14 OF TABLE 6A)

-8 - D[illegible]   (USE ONE LINE FOR EACH MAJOR CROP)

F15 How many times did he/she plant (NAME CROPS) in 1982?
(ENTER RESPONSE IN COL. F15 OF TABLE 6A)

-8 - DK

F16 On the average, how much did he/she spend each planting season for seedlings, fertilizer, tools, hired labor, insecticides, etc.? (IN PESOS. ENTER RESPONSE IN COL. F16 OF TABLE 6A)

-8 - DK

F17 How many times did he/she harvest (NAME CROPS) in 1982?

1 - Once a year   4 - Four times a year
2 - Twice a year   -8 - NR/DK
3 - Thrice a year   -9 - NA

ENTER RESPONSE IN COL. F17 OF TABLE 6A.

F18 What did he/she do with his/her share of the total produce per harvest in 1982?

1 - Sold all produce	GO TO F19
2 - Sold one portion, kept some	GO TO F19 & F20
3 - Shared the produce with owner	GO TO F20 + F19
4 - Sharing and giving away for free	GO TO F20
5 - All used for home consumption	GO TO F20
-8 - NR/DK	CODE -8 IN COLS. F19-F20
-9 - NA	

F19 On the average, how much did he/she receive for that portion of the produce that was sold? (ENTER RESPONSE IN COL. F19 OF TABLE 6A)

-8 - DK

F20 How much would he/she have received for that portion of the produce that was kept for home consumption if he/she had sold it? (ENTER RESPONSE IN PESOS IN COL. F20 OF TABLE 6A)

-8 - DK

Table 61. Income Derived from Farming (excl. livestock)

LINE NO.	NAME	WORKING TIME		PARCELS OF CULTIVATED LAND		MAJOR CROPS PLANTED IN 1982	NO. OF TIMES PLANTED IN 1982	AVERAGE EXPENSES FOR ALL CROPS PER PLANTING SEASON IN 1982	NO. OF TIMES MAJOR CROP WAS HARVESTED IN 1982	MONEY VALUE OF CROP PER HARVEST IN 1982	
		Hrs. Per Day	Days Per Week	Number	Size of all Parcels					Sold	Home Use
		(F9)	(F10)	(F11)	(F12/F13)	(F14)	(F15)	(F16)	(F17)	(F19)	(F20)

USE ONE LINE FOR EACH MAJOR CROP!

III. INCOME DERIVED FROM LIVESTOCK RAISING

QUESTION F21 AND, IF APPLICABLE, THE QUESTIONS RELATED TO LIVESTOCK RAISING, ARE TO BE ASKED OF ALL HOUSEHOLDS REGARDLESS WHETHER THEY OPERATE A FARM OR NOT!

F21 Is any member of your household presently engaged in the raising of livestock (cattle, pigs, goats, ducks, chicken, others)?

1 - Yes — ASK FOR NAME OF HOUSEHOLD MEMBER AND ENTER IT IN TABLE 6B TOGETHER WITH HIS/HER LINE NUMBER SHOWN IN TABLE 1

0 - No
-8 - NR/DK
-9 - NA
} GO TO F26

ASK QUESTIONS F22 AND F23 OF ALL PERSONS LISTED IN TABLE 6B.

F22 On an average working day, how many hours ~~a day~~ does he/she usually work with the livestock?
(ENTER NUMBER OF HOURS PER DAY IN COL. F22 OF TABLE 6B)

-8 - NR/DK -9 - NA

28

F23 In an average working week, how many days does he/she usually work with the livestock?
(ENTER NUMBER OF DAYS PER WEEK IN COL. F23 OF TABLE 6B)

-8 - NR/DK -9 - NA

IF THE LIVESTOCK RAISING IS A FAMILY OPERATION, ASK QUESTIONS F24 THRU F29 ONLY OF THE HEAD OR THE ELDEST MEMBER OF THE FAMILY. IF ANY FAMILY OR HOUSEHOLD MEMBER RAISES LIVESTOCK ELSEWHERE INDEPENDENTLY OF THE FAMILY OR HOUSEHOLD, ASK ALL QUESTIONS OF THIS HOUSEHOLD MEMBER.

F24 What kind of livestock does he/she presently raise?
(ENTER NAME AND CORRESPONDING CODE OF EACH TYPE OF LIVESTOCK; EACH ONE ON A SEPARATE LINE! IN COL. F24 OF TABLE 6B)

1 - Chicken
2 - Ducks
3 - Turkeys
4 - Pigs
5 - Goats
6 - Carabaos
7 - Cattle
8 - Other (SPECIFY) ____________
-8 - NR/DK
-9 - NA

ASK QUESTIONS F25 THRU F29 FOR EACH TYPE OF LIVESTOCK CURRENTLY OWNED BY RESPONDENT.

F25 How many pieces of (NAME EACH KIND OF LIVESTOCK MENTIONED IN COL. F24) does he/she presently have?
(ENTER RESPONSE IN COL. F25 OF TABLE 6b)

-8 - NR/DK
-9 - NA (started with livestock in 1983)

F26a Did he/she sell any livestock or livestock products (eggs, milk) in 1982? (ENTER RESPONSE IN COL. F26 OF TABLE 6B)

1 - Yes, all
2 - Yes, some
} CONTINUE

0 - No
-8 - NR/DK
} GO TO F29

-9 - NA (started with livestock in 1983)

F27 What was the approximate expense which he/she had for this particular type of livestock in 1982 (purchase, feeding, veterinarian, etc.)?
(ENTER ANSWER IN PESOS IN COL. F27 OF TABLE 6B)

F28 What was his/her total income in 1982 for the sale of this type of livestock or livestock products?
(ENTER RESPONSE (IN PESOS) IN COL. F28 OF TABLE 6B)

-8 - NR/DK
-9 - NA (started with livestock in 1983)

F29 How much do you think would he/she have earned in 1982 from that part of the livestock or the livestock products that was used for home consumption in case he/she had sold it?
(ENTER RESPONSE (IN PESOS) IN COL. F29 OF TABLE 6B)

-8 - NR/DK
-9 - NA (started with livestock in 1983)

Table 6B. Income Derived from Raising of Livestock

LINE NO.	NAME	WORKING TIME		KIND OF LIVESTOCK PRESENTLY RAISED		PIECE OF LIVESTOCK PRESENTLY OWNED	SELLING OF LIVESTOCK OR LIVESTOCK PRODUCTS IN 1982	TOTAL EXPENSES FOR LIVESTOCK IN 1982	TOTAL INCOME FROM SALE OF LIVESTOCK/ LIVESTOCK PRODUCTS IN 1982	APPROXIMATE VALUE OF HOME-CONSUMED LIVESTOCK/ LIVESTOCK PRODUCT IN 1982
		Hrs. Per Day	Days Per Week	Kind	Code					
		(F22)	(F23)	(F24)		(F25)	(F26)	(F27)	(F28)	(F29)

IV. INCOME DERIVED FROM FISHING ACTIVITIES

FROM THE SCRATCH TABLE, THE RECORD OF MARKET ACTIVITIES, COPY LINE NUMBERS AND NAMES OF ALL RESIDENT HOUSEHOLD MEMBERS WHO EITHER ARE OR WERE ENGAGED IN FISHING OVER THE PAST FOUR MONTHS. THESE ARE ONLY THOSE PERSONS WITH A CODE "2" IN COL. E3 AND/OR E8 AND A CODE OF "1" OR "4" IN COL. E5 AND/OR E10. FISHERS HIRED BY OTHERS AND WORKING FOR PAY ARE WAGE WORKERS. IF FISHING IS A FAMILY/HOUSEHOLD ACTIVITY, ASK F30 OF ALL MEMBERS LISTED IN TABLE 7, BUT ASK QUESTION F31 OF ONLY ONE MEMBER, THE ONE IN CHARGE OF THE FAMILY/HOUSEHOLD FISHING OPERATION.

F30 How many days per week does(did) he(she) usually go fishing?
-8 - NR/DK
-9 - NA

F31 How much is(was) his(her) usual net income out of a day's catch, including that portion of the catch which the household itself consumed?
-8 - NR/DK
-9 - NA

Table 7. Income Derived from Fishing Activities

LINE NO.	NAME	AVE.NO. DAYS OF FISHING PER WEEK (F30)	USUAL DAILY NET INCOME (IN PESOS) (F31)

V. INCOME DERIVED FROM SELF-EMPLOYMENT

FROM THE SCRATCH TABLE, THE RECORD OF MARKET ACTIVITIES, COPY LINE NUMBERS AND NAMES OF ALL HOUSEHOLD MEMBERS WHO ARE OR WERE SELF-EMPLOYED DURING THE PAST FOUR MONTHS. THESE ARE ONLY PERSONS WITH A CODE OF "2" OR "3" IN COL. E3 AND/OR E8 AND A CODE OF "1" OR "4" IN COL. E5 AND/OR E10. IF A FAMILY/HOUSEHOLD OPERATES A STORE IN WHICH SOME MEMBERS WORK AS UNPAID FAMILY WORKERS, ASK F32 AND F33 OF ALL MEMBERS WORKING IN THE STORE, BUT F34 ONLY OF THE MEMBER IN CHARGE OF THE STORE.

F32 How many hours does(did) he(she) work in a usual day?
-8 - NR/DK
-9 - NA

F33 How many days in a week does(did) he(she) usually work?
-8 - NR/DK
-9 - NA

F34 How much on the average is(was) his(her) daily net income?
-8 - NR/DK
-9 - NA

GO TO NEXT PERSON LISTED OR, IF LIST IS COMPLETE, TO F35.

Table 8. Income Derived from Self-Employment

LINE NO.	NAME	AVE. NO. HOURS WORKED/DAY (F32)	AVE. NO. DAYS WORKED/WEEK (F33)	AVERAGE DAILY NET INCOME (F34)

VI. HOUSEHOLD INCOME DERIVED FROM OTHER SOURCES

F35 Does(did) the household or any of its members have other sources of income?

1 - Yes — CONTINUE
0 - No — CODE -9 IN ALL BOXES OF F36 AND IN F37
-8 - NR/DK — CODE -8 IN ALL BOXES OF F36 AND IN F37

☐

F36 What are these source(s) of income? (CODE ALL!)

1 - Yes
0 - No
-8 - NR/DK
-9 - NA

Rent from agricultural/commercial land ☐

Income from boarders/lodgers ☐

Pensions, dividends, bonuses, savings' interest ☐

Cash - remittances from children, parents, other relatives, friends or anyone else ☐

Remittances from spouse abroad/sustento ☐

______________________ Other (SPECIFY) ☐

F37 How much additional income from all of these sources together did the household receive in 1982? (PESOS)

-8 - NR/DK
-9 - NA

☐☐☐☐☐

F38 Did you or your household receive any income in kind (food or clothing) in 1982 from children, parents, other relatives, friends or anybody else?

1 - Yes — CONTINUE
0 - No — CODE -9 IN F39, GO TO F40
-8 - NR/DK — CODE -8 IN F39, GO TO F40

☐

F39 What was the approximate value (IN PESOS) of this income in kind in 1982?

-8 - NR/DK
-9 - NA

☐☐☐☐

VII. INCOME DERIVED FROM HOME GARDENING

F40 Does your household engage in home gardening?

1 - Yes — CONTINUE
0 - No } GO TO G1
-8 - NR/DK }

☐

F41 Please tell me the names of all of your household members that usually help with home gardening.

LIST IN TABLE 9 BELOW THE NAMES OF ALL PERSONS MENTIONED. LIST THE NAMES, TOGETHER WITH THEIR LINE NUMBERS, IN THE ORDER IN WHICH THEY APPEAR IN COL. F41 OF TABLE 1 (HOUSEHOLD ROSTER). CHECK NAMES AGAINST TABLE 3B, COL. E30.

FOR EACH PERSON LISTED IN TABLE 9 ASK:

F42 In a usual week, how many hours does he/she usually help with home gardening?

ENTER RESPONSE (IN HOURS) IN COL. F42 OF TABLE 9.
-8 - NR/DK

Table 9. Household Members Engaged in Home Gardening

LINE NO.	NAME (F41)	HOURS OF HOME GARDENING PER WEEK (F42)

F43 What does your household normally plant in your home garden? (CODE ALL!)

1 - Yes
0 - No
-8 - NR/DK
-9 - NA

Fruits ☐
Vegetables ☐
Ornamental Plants ☐
Corn ☐
__________ Other (SPECIFY) ☐

F44 What does the household usually do with the produce from the garden?

1 - Sell all — GO TO F45
2 - Keep all for home use — GO TO F46
3 - Sell some, keep some for home use — GO TO F45 AND F46
-8 - NR/DK }
-9 - NA } CODE -8 IN F45 AND F46 ☐

F45 How much, on the average, does (did) the household get per month from the sale of fruits, vegetables or plants which were grown in the home garden?

0 - No income<br>-8 - NR/DK<br>-9 - NA	Total Pesos Received per Month from Sale of Produce ☐☐☐

F46 How much money (IN PESOS) do you save each month by growing your own vegetables or fruits which otherwise you would have to buy from the market?

0 - No savings<br>-8 - NR/DK<br>-9 - NA	Estimated Savings per Month through Home Gardening ☐☐☐

G. MATERNAL WORK AND PREGNANCY HISTORY

ALL QUESTIONS IN BLOCK G AND ALL THE FOLLOWING BLOCKS ARE TO BE ANSWERED BY THE PREGNANT WOMAN.

IF THE WOMAN IS CURRENTLY NOT ENGAGED IN ANY TYPE OF INCOME-GENERATING ACTIVITY (SEE QUESTION E1, TABLE 3A), START WITH G1, OTHERWISE, CODE "1" IN G1 AND START WITH G2.

G1 Have you ever worked in a job earning any income for yourself directly or for your family?

CONSIDER ONLY JOBS FOR WHICH THE WOMAN RECEIVED INCOME IN CASH OR KIND. UNPAID FAMILY WORK ON FAMILY FARM OR IN FAMILY BUSINESS IS EXCLUDED.

1 - Yes — CONTINUE
0 - No — GO TO G15
-8 - NR/DK — CONTINUE

☐

G2 Before you were married, how many years did you work?

0 - did not work before marriage
1 - 1 year
:
-8 - NR/DK
} CONTINUE

-9 - NA (woman is not married) — GO TO G4

☐☐ YEARS

G3 Since you were married, how many years have you worked?

00 - did not work since marriage — GO TO G15

01 - 1 year
02 - 2 years
:
-8 - NR/DK
} GO TO G5

-9 - NA (woman is not married) — GO TO G4

☐☐ YEARS

G4 What is the total number of years you have worked? (unmarried women only)

-8 - NR/DK

☐☐

G5 Did you do anything for pay last week?

PAY CAN BE EITHER IN CASH OR KIND

1 - Yes — CONTINUE

0 - No
-8 - NR/DK
-9 - NA
} GO TO G15

☐

G6 Where was this work of yours carried out?

1 - At home — GO TO G10

2 - Immediate neighborhood
3 - Same barangay
4 - Different barangay, same municipality/city
NAME OF BARANGAY ____________
5 - Different municipality/city, same province
NAME OF MUNICIPALITY ____________
6 - Different province
NAME OF PROVINCE ____________
} CONTINUE

-8 - NR/DK
-9 - NA
} GO TO G10

☐

G7 How long (in minutes) does it usually take you to travel to your place of work?

USE THREE-DIGIT CODE, E.G., 030 FOR 30 MINUTES

-8 - NR/DK
-9 - NA (does not travel to work each day)

☐☐☐

G8 How do you usually get to your place of work?

1 - walk — GO TO G10
2 - private car
3 - taxi, PU
4 - jeepney, bus, minibus
5 - truck
6 - motorcycle
7 - tricycle
8 - bicycle
9 - tartanilla
10 - other (SPECIFY): ______
11 - combination (SPECIFY CODES): ______
-8 - NR/DK
-9 - NA

(2 through -8: CONTINUE)

☐

G9 What is the usual daily travel cost (in pesos) to and from your place of work including cost for travel at lunch time?

USE FOUR DIGIT CODE, E.G., 0150 FOR ₱ 1.50.

-8 - NR/DK
-9 - NA

☐☐☐☐

G10 Do you have any children of pre-school age (under 6) who are living with you in this household?

1 - Yes — CHECK QUESTION G6. IF ANSWER IS 1 OR -8, CONTINUE; IF ANSWER IS 2, 3, 4, 5 OR 6, GO TO G13.

0 - No
-8 - NR/DK
-9 - NA

(0, -8, -9: GO TO G15)

☐

G11 Can you take care of your children of pre-school age while you work?

1 - Yes — GO TO G15
0 - No
-8 - NR/DK
-9 - NA

(0, -8, -9: CONTINUE)

☐

G12 Can you feed your children of pre-school age while you work?

1 - Yes — GO TO G15
0 - No
-8 - NR/DK
-9 - NA

(0, -8, -9: CONTINUE)

☐

G13 Can you take your children of pre-school age with you to work?

0 - No, not allowed
1 - Yes allowed, but I do not
2 - Usually take some of them
} CONTINUE

3 - Usually take all of them
-8 - NR/DK
-9 - NA
} GO TO G15

G14 Can you come home during working time to feed your children of pre-school age?

1 - Yes, always
2 - Yes, on some days
0 - No
-8 - NR/DK
-9 - NA

G15 At what age did you start menstruating (e.g., 10, 11, 12, etc.)?

CODE AGE OF FIRST MENSTRUATION.

G16 Is this current pregnancy your first one?

1 - Yes — GO TO G26
0 - No
-8 - NR/DK
} CONTINUE

G17 When did you have your last live birth? (MONTH, YEAR)

-8 - NR/DK
-9 - NA - (woman had no previous live birth)

MONTH YEAR

G18 Since your last live birth, did you have any stillbirth, miscarriage or abortion?

1 - No; last live birth was last pregnancy termination

CODE -9 IN G19 AND GO TO G20.

2 - Yes, had one stillbirth since
3 - Yes, had one abortion since
4 - Yes, had one miscarriage since
5 - Yes, had more than one termination (SPECIFY CODES): __________
} CONTINUE

-8 - NR/DK
-9 - NA

G19 When did you have your last pregnancy termination, i.e., stillbirth, miscarriage, abortion? (MONTH, YEAR)

-8 - NR/DK
-9 - NA

MONTH YEAR

G20 How many times have you been pregnant in the past, excluding this current pregnancy?

CODE ACTUAL NUMBER, INCLUDING PREGNANCIES THAT TERMINATED IN STILLBIRTHS, MISCARRIAGES, OR ABORTIONS.

-8 - NR/DK
-9 - NA (current pregnancy is the first one)

G21 How many of your pregnancies have resulted in live births? (INCLUDE CHILDREN WHO HAVE DIED AFTER BIRTH)

-8 - NR/DK
-9 - NA (current pregnancy is the first one)

G22 Out of the total number of live births that you have had, how many of the children are still alive today?

-8 - NR/DK
-9 - NA (woman had no previous live birth)

G23 How many of all your previous pregnancies have resulted in a stillbirth?

-8 - NR/DK
-9 - NA

G24 How many of all your previous pregnancies have resulted in a miscarriage and/or abortion?

-8 - NR/DK
-9 - NA

G25 Did you menstruate between this pregnancy and the termination of the previous one?

1 - Yes CONTINUE
0 - No }
-8 - NR/DK } GO TO G29
-9 - NA }

G26 When did you last menstruate? (Tell me the last day of your LAST REGULAR MENSTRUAL PERIOD)

Which calendar year?

Which month?

Which week within this month (first, second, third, fourth)?

CODE -8 IF ANSWER TO EITHER YEAR, MONTH OR WEEK IS NR/DK

G27 How long (in days) was this menstruation?

1 - one day
2 - two days
3 - three days
4 - four days
5 - five days
6 - six days
7 - seven days
-8 - NR/DK
-9 - NA

G28 How would you describe the intensity of flow of this menstruation compared to your usual menstruation?

1 - same as usual menstruation
2 - more intense than usual menstruation
3 - less intense than usual menstruation
-8 - NR/DK
-9 - NA

☐

G29 When did you first feel a quickening or movement of the fetus?

Which calendar year? ☐☐

Which month? ☐☐

Which week within this month (first, second, third, fourth)? ☐

CODE -8 IF ANSWER TO EITHER YEAR, MONTH, OR WEEK IS NR/DK.

G30 When do you expect to deliver your baby? (GIVE EXACT MONTH/DAY/YEAR IF POSSIBLE. CODE -8 IF NOT KNOWN)

Which calendar year? ☐☐

Which month? ☐☐

Which week within this month (first, second, third, fourth)? ☐

H. HEALTH SERVICES AND PRENATAL CARE

I. Health Service Information

REPLIES TO QUESTIONS H1 THRU H3 ARE TO BE ENTERED IN TABLE 10

WE WOULD LIKE TO ASK YOU NOW A FEW QUESTIONS ABOUT HEALTH SERVICES OF WHICH YOUR FAMILY CAN AVAIL AND ABOUT PRE-NATAL CARE.

H1 How many minutes would it take you to travel to (1) mananabang (2) mananambal (3) private midwife (4) private doctor (5) RHU/CHO (6) Pharmacy/Botica in barangay?

USE THREE-DIGIT CODE, e.g., 030 for 30 minutes.

H2 If you were to go to: mananabang, mananambal, private midwife, private doctor, RHU/CHO, pharmacy/botica sa barangay, would you walk?

1 - Yes, walk
2 - Yes, walk and take transportation
0 - No, would not walk
-8 - NR/DK
-9 - NA (Practitioner visits household)

H3 How much would the travel cost (in pesos) be for transportation if you would go to: mananabang, mananambal, private midwife, private doctor, RHU/CHO, pharmacy/botica sa barangay?

USE FOUR-DIGIT CODE, e.g., 0150 for ₱ 1.50.

-8 - NR/DK
-9 - NA

Table 10. Health Usage

HEALTH PERSON/ FACILITY	TRAVEL TIME (Minutes) (H1)	WALK? (H2)	TRAVEL COST (Pesos) (H3)
Mananabang			
Mananambal			
Private Doctor			
Private Midwife			
RHU/CHO			
Pharmacy/Botica ~~sa Barangay~~			

II. Health Insurance Coverage

H4 Are you or any other member of your family a member of the SSS or GSIS?

1 - Yes — CONTINUE
0 - No
-8 - NR/DK } CODE -9 IN H5 TO H8, CONTINUE WITH H9

☐

H5 Does the health insurance provided by SSS/GSIS cover part or all of your pregnancy-related health-care expenses?

1 - All
2 - Part only
3 - No Coverage
-8 - NR/DK
-9 - NA

☐

H6 Does this insurance cover your husband also?

1 - Yes
0 - No
-8 - NR/DK
-9 - NA

IF MOTHER HAS LIVING CHILDREN, ASK H7. IF SHE HAS NO LIVING CHILDREN, ASK H8.

H7 Does this insurance cover your school-aged (ages 5-14) children?

1 - Yes
0 - No
-8 - NR/DK
-9 - NA

H8 After your baby is born, will you include him/her also in this insurance?

1 - Yes
0 - No
-8 - NR/DK
-9 - NA

H9 Are you a holder of any <u>other</u> health insurance policy, i.e., not SSS or GSIS?

1 - Yes CONTINUE
0 - No
-8 - NR/DK } GO TO H14
-9 - NA

H10 Does this policy cover part or all of your pregnancy-related health-care expenses?

1 - All
2 - Part
3 - No Coverage
-8 - NR/DK
-9 - NA

H11 Does it cover health care for your husband?

1 - Yes
0 - No
-8 - NR/DK
-9 - NA

IF WOMAN HAS LIVING CHILDREN, ASK H12 & H13. IF SHE HAS NO LIVING CHILDREN, CODE -9 IN H12 AND H13 AND CONTINUE WITH H14.

H12 Does it cover health care for your school-aged (5-14) children?

1 - Yes
0 - No
-8 - NR/DK
-9 - NA

H13 Will you include your new baby in this policy?

1 - Yes
0 - No
-8 - NR/DK
-9 - NA

III. Health of Woman:

WE WOULD LIKE TO ASK A FEW QUESTIONS ABOUT YOUR HEALTH.

H14 Are you sick now or have you been sick in the last seven days from any illness?

1 - Yes — CONTINUE
0 - No — GO TO H19
-8 - NR/DK — GO TO H19

☐

H15 What illness(es) do you have now or did you have in the last seven days?

CHECK AS MANY ANSWERS AS THE MOTHER MENTIONS

1 - Yes
0 - No
-8 - NR/DK

Blood in sputum (tuberculosis) ☐

Watery stools ☐

Colds, nasal congestion, nasal discharge ☐

Coughs ☐

Fever ☐

________________ Other (SPECIFY) ☐

IF MOTHER HAS MENTIONED "blood in sputum" (tuberculosis), GO TO H19
IF MOTHER DID NOT MENTION tuberculosis, ASK

H16 During the last seven days, has there been anything wrong with your lungs?

1 - Yes — GO TO H19
0 - No — CONTINUE
-8 - NR/DK — CONTINUE
-9 - NA — GO TO H19

☐

H17 During the last seven days, did you notice any blood in your sputum?

1 - Yes — GO TO H19
0 - No — CONTINUE
-8 - NR/DK — CONTINUE
-9 - NA — GO TO H19

☐

H18 During the last seven days, did you take any medicine like ODINAH, TRISOVIT, or INH?

1 - Yes
0 - No
-8 - NR/DK
-9 - NA

☐

H19 Did you have any bleeding episode at any time during this pregnancy?

1 - Yes — CONTINUE
0 - No — GO TO H21
-8 - NR/DK — GO TO H21

☐

H20 When did the bleeding occur?

ENTER CODE FOR ALL 3 PREGNANCY TRIMESTERS

1 - Yes
0 - No
-8 - NR/DK
-9 - NA

First 3 months of pregnancy ☐

Month 4 to 6 of pregnancy ☐

After 6th month of pregnancy ☐

IV. Pre-Natal Care

H28 Have you consulted any health practitioner for pre-natal care during this pregnancy?

1 - Yes — CONTINUE
0 - No
-8 - NR/DK } GO TO H40

☐

ENTER ALL REPLIES TO QUESTIONS H29 THRU H39 IN APPROPRIATE LINE OF TABLE 11. ENTER CODES ONLY. ASK QUESTIONS H30 TO H39 FOR ALL PRACTITIONERS IDENTIFIED THROUGH QUESTION H29.

H29 Who have you visited?

Doctor in government facility
Nurse in government facility
Midwife in government facility
Other personnel in RHU/CHO/PC
Private or private hospital doctor
Private or private hospital nurse
Private or private hospital midwife
Mananabang
Mananambal

MAKE A CHECKMARK IN COL. H29 OF TABLE 11 AFTER EACH KIND OF PRACTITIONER MENTIONED. THEN ASK H30 TO H39 FOR EACH PRACTITIONER MENTIONED.

H30 During what month of pregnancy did you make your first consultation?

0 - As soon as suspected being pregnant
1 - 1st month of pregnancy
:
9 - 9th month of pregnancy
-8 - NR/DK
-9 - NA (did not visit any)

H33 What was the total cost (PESOS) of all visits to this practitioner?

-7 - Package deal, payable after delivery
-8 - NR/DK
-9 - NA

USE THREE-DIGIT CODE, e.g., 020 FOR ₱ 20.00

H34 Did this practitioner suggest that you take supplementary vitamins or minerals?

1 - Yes — CONTINUE
0 - No
-8 - NR/DK
-9 - NA } GO TO H37

H35 Were you given vitamins or minerals for free or did you buy any?

0 - No, were neither given nor bought any — GO TO H37
1 - Yes, was given for free
2 - Yes, bought them myself
3 - Yes, some were given for free, others were bought } CONTINUE
-8 - NR/DK
-9 - NA — GO TO H37

H36 Did you take them?

1 - Yes
0 - No
-8 - NR/DK
-9 - NA

H37 Did you get, from this practitioner, an anti-tetanus injection?

1 - Yes
0 - No
-8 - NR/DK
-9 - NA

H38 Did this health practitioner suggest how you should nurse your baby?

1 - Yes — CONTINUE
0 - No
-8 - NR/DK } GO TO H40
-9 - NA

H31 How many consultations in all for prenatal care to this practitioner did you make during this pregnancy?

-8 - NR/DK
-9 - NA (did not visit any)

H32 Where did most of these consultations take place?

1 - At home
2 - RHU/CHO/PC/Clinic
3 - Barangay Health Station
4 - Hospital, government/private
5 - Resident of practitioner
6 - Private clinic
-8 - NR/DK
-9 - NA

H39 What did he suggest?

1 - Breastfeeding
2 - Formula feeding
3 - Mixed feeding
4 - Others (SPECIFY IN COL. H39 OF TABLE 11)
-8 - NR/DK
-9 - NA

GO TO H30 FOR NEXT HEALTH PRACTITIONER MENTIONED OR, AFTER ANSWERS FOR ALL PRACTITIONERS MENTIONED HAVE BEEN COMPLETED, GO TO H40.

Table 11. Pre-Natal Care Chart

TYPE OF PRACTITIONER	CHECK	1st VISIT (Month of Pregnancy)	NO. OF CONSUL-TATIONS	PLACE OF CONSUL-TATIONS	COST OF CONSUL-TATIONS	VITAMIN SUGGES-TION	OBTAINING OF VITAMINS	TAKING OF VI-TAMINS	ANTI-TETANUS INJECTION	INFANT FEEDING RECOMMENDATION		
										Code	Particular Recommendation Code	Others (SPECIFY)
(H29)		(H30)	(H31)	(H32)	(H33)	(H34)	(H35)	(H36)	(H37)	(H38)	(H39)	
Doctor in government hospital or health unit												
Nurse in government hospital or health unit												
Midwife in government hospital or health unit												
Other personnel in RHU/CHO/PC												
Private Doctor incl. doctor in priv. hospital												
Private Nurse incl. nurse in priv. hospital												
Private Midwife incl. midwife in priv. hospital												
Mananabang												
Manananbal												

V. Infant Feeding Influences

H40 Have any other persons recommended or suggested to you how you should feed your baby?

1 - Yes — CONTINUE
0 - No } GO TO H43
-8 - NR/DK }

H41 Who were these persons?

ENTER REPLIES IN COL. H41 OF TABLE 12.

1 - Yes
0 - No
-8 - NR/DK
-9 - NA

H42 What did they recommend?

ENTER REPLIES IN COL. H42 OF TABLE 12. MATCH REPLY WITH TYPE OF PERSON WHO MADE THE SUGGESTION!

1 - Breastfeeding
2 - Formula feeding
3 - Mixed feeding
4 - Feed other foods
8 - Other (SPECIFY IN COL. H42 OF TABLE 12)
-8 - NR/DK
-9 - NA

Table 12. Infant Feeding Suggestions

RECOMMENDING PERSON	Code	RECOMMENDATION Code	RECOMMENDATION Other (SPECIFY)
(H41)		(H42)	
Infant food company representative			
Woman's mother			
Husband			
Other relative			
Neighbor/friend			
Other 1 (SPECIFY) ____________			
Other 2 (SPECIFY) ____________			

H43 Have you seen or heard any advertisement or read any pamphlets, newspapers and magazines, or heard radio/TV concerning foods to feed your baby?

1 - Yes — CONTINUE
0 - No } GO TO NEXT BLOCK
-8 - NR/DK }

H44 What was the source of that recommendation?

CODE AS MANY INFLUENCES AS MOTHER MENTIONS IN COL. H44.

1 - Yes
0 - No
-8 - NR/DK
-9 - NA

H45 What was recommended?

CODE THE RECOMMENDED KIND OF FEEDING IN COL. H45 BEHIND SOURCE OF RECOMMENDATION.

1 - Breastfeeding
2 - Formula feeding
3 - Mixed feeding
4 - Other (SPECIFY): ____________
5 - Combination (SPECIFY CODES): ___
-8 - NR/DK
-9 - NA

	H44	H45
Billboards	☐	☐
Newspapers	☐	☐
Magazines	☐	☐
Pamphlets	☐	☐
Radio	☐	☐
TV	☐	☐
Books	☐	☐
Nutribus	☐	☐
Others	☐	☐

I. INFANT NUTRITION KNOWLEDGE, ATTITUDE AND DECISION MAKING

INTERVIEWER: DO NOT READ PRECODED ANSWERS TO RESPONDENT OR PROMPT RESPONDENT IN ANY WAY TO GIVE ANY OF THE PRECODED ANSWERS. INSTEAD, CLASSIFY THE ANSWERS GIVEN BY THE RESPONDENTS IN TERMS OF THE PRECODED ANSWERS.

FOR ALL QUESTIONS IN THIS BLOCK CALLING FOR RELATIONSHIP OF PERSONS TO PREGNANT MOTHER, CHECK COL. B5 OF TABLE 1 FOR RELATIONSHIP OF PERSON BEING CONSULTED/MAKING FINAL DECISION. IF THE NAME OF THE PERSON BEING CONSULTED/MAKING FINAL DECISION DOES NOT APPEAR IN TABLE 1, ASK THE QUESTION: 'HOW IS THIS PERSON RELATED TO YOU?' IF THE NAME OF THE PERSONS MENTIONED APPEARS IN COL. B5 OF TABLE 1, ENTER CODE ONLY UNDER "DESIGNATION". YOU MAY ENTER UP TO TWO PERSONS THAT WILL BE CONSULTED.

I1 How do you intend to feed your baby in the first week after delivery? (CODE APPRORRIATE CATEGORY)

1 - Breastfeeding only	CONTINUE
2 - Formula feeding only	GO TO I4
3 - Mixed feeding	CONTINUE
4 - Other (SPECIFY): ________	} GO TO I4
-8 - NR/DK	

I2 What should be done with the first flow (colostrum) from your breast?

1 - Should be given to the newborn	GO TO I4
2 - Should not be given to the newborn but should be discarded	CONTINUE
-8 - NR/DK	GO TO I4
-9 - NA	

I3 Why should the first flow (colostrum) not be given to the newborn?

CODE AS MANY ANSWERS AS MOTHER GIVES.

1 - Yes
0 - No
-8 - NR/DK
-9 - NA

Too thin

Not clean

Toxic

Not good for baby

________ Other (SPECIFY)

Do not know exact reason

I4 Did you consult anyone or will you consult someone regarding how to feed your infant for the first few months, or do you make the decision all by yourself?

0 - Make decision myself	GO TO I6
1 - Will consult with someone	} CONTINUE
-8 - NR/DK	

15 Who is or will this person be who you did or will consult about how to feed your baby for the first few months? How is this person related to you?

PERSON WHO WAS/WILL BE CONSULTED	RELATIONSHIP TO PREGNANT MOTHER (Designation)
1. ______	______
2. ______	______

16 At what age should the infant be given semi-solid food supplements for the first time?

(SEMI-SOLIDS INCLUDE LUGAW, CERELAC, CHAMPORADO, ETC.)

1 - One month
2 - Two months
3 - Three months
4 - Four months
5 - Five months
6 - Six months
7 - Seven months
8 - Eight months
9 - Nine months
10 - Ten months
11 - Eleven months
12 - Twelve months
13 - Over 12 months
-8 - NR/DK
-9 - NA

I7 Do you think you will consult anyone when to give supplementary food to your infant, or will you make that decision all by yourself?

0 - Make decision myself — GO TO I9
1 - Will consult with someone } CONTINUE
-8 - NR/DK } CONTINUE

I8 Whom do you think you will consult regarding the giving of the first supplementary food to your infant? How is this person related to you?

PERSON WHO MAY BE CONSULTED	RELATIONSHIP TO PREGNANT MOTHER
1. ______	______
2. ______	______

I9 Do you think there are differences in growth between a breastfed infant and a bottle-fed one?

1 - Yes — CONTINUE
0 - No — CODE -9 IN I10, GO TO I11
-8 - NR/DK — CODE -8 IN I10, GO TO I11

I10 What are these differences?

CODE AS MANY ANSWERS AS THE MOTHER GIVES

1 - Yes
0 - No
-8 - NR/DK
-9 - NA

Breastfed infants are taller	☐
Breastfed infants are heavier/fuller	☐
Breastfed infants are healthier	☐
Bottlefed infants are taller	☐
Bottlefed infants are heavier/fuller	☐
Bottlefed infants are healthier	☐
____________ Others (SPECIFY)	☐

I11 Do you think that breastfeeding will affect the onset of the mother's next pregnancy?

1 - Yes	CONTINUE	
0 - No	CODE -9 IN I12, GO TO I13	☐
-8 - NR/DK	CODE -8 IN I12, GO TO I13	

I12 What are the effects of breastfeeding on the onset of the mother's next pregnancy?

CODE AS MANY ANSWERS AS THE MOTHER GIVES

1 - Yes
0 - No
-8 - NR/DK
-9 - NA

Delays onset of pregnancy	☐
Protects against pregnancy	☐
____________ Other (SPECIFY)_	☐

I13 Do you think that breastfeeding will affect the sexual relationships between a nursing mother and her husband?

1 - Yes	CONTINUE	
0 - No	CODE -9 IN I14, GO TO I15	☐
-8 - NR/DK	CODE -8 IN I14, GO TO I15	

I14 What are the effects of breastfeeding on the sexual relationships between the nursing mother and her husband?

1 - Yes
0 - No
-8 - NR/DK
-9 - NA

Decreases frequency of contact	☐
Increases frequency of contact	☐
____________ Other (SPECIFY)	☐

I15 Do you think that breastfeeding will affect the health of the mother?

1 - Yes	CONTINUE	
0 - No	CODE -9 IN I16, GO TO I17	☐
-8 - NR/DK	CODE -8 IN I16, GO TO I17	

I16 What are the effects of breastfeeding on the health of the mother?

CODE AS MANY ANSWERS AS THE MOTHER GIVES

1 - Yes
0 - No
-8 - NR/DK
-9 - NA

Mother will become thin	☐
Mother will tire easily	☐
Mother will have poor health	☐
______________ Other (SPECIFY)	☐

I17 Do you think that breastfeeding over a long period of time will affect the quality of the mother's breast milk?

1 - Yes	CONTINUE	
0 - No	CODE -9 IN I18, GO TO I19	☐
-8 - NR/DK	CODE -8 IN I18, GO TO I19	

I18 What are the effects of breastfeeding over a long period of time on the quality of the mother's breast milk?

CODE AS MANY ANSWERS AS THE MOTHER GIVES

1 - Yes
0 - No
-8 - NR/DK
-9 - NA

Milk becomes less nutritious	☐
Milk becomes watery	☐
Milk is no longer good for the baby	☐
______________ Others (SPECIFY)	☐

I19 Will breastfeeding affect the shape of the mother's breasts?

1 - Yes	CONTINUE	
0 - No	CODE -9 IN I20, GO TO I21	☐
-8 - NR/DK	CODE -9 IN I20, GO TO I21	

I20 What are the effects of breastfeeding on the shape of the mother's breasts?

CODE AS MANY ANSWERS AS THE MOTHER GIVES

1 - Yes
0 - No
-8 - NR/DK
-9 - NA

Breasts will become big	☐
Breasts will sag	☐
Breasts will shrink	☐
______________ Others (SPECIFY)	☐

I21 Should sungil be given to the infant before he or she is given supplementary food?

1 - Yes
0 - No
-8 - NR/DK

I22 In your opinion, what is the effect of adding oil to the usual diet/lugaw of an infant of less than one year of age?

1 - No effect
2 - More nutritious
3 - Changes flavor of food
4 - Causes diarrhea
5 - Causes constipation
6 - Other (SPECIFY): ______________________
-8 - NR/DK

I23 In your opinion, should a mother who is exposed to the sun breastfeed her baby?

1 - Yes
0 - No
-8 - NR/DK

I24 Do you think you will consult anyone concerning when to wean your infant, or will you make that decision all by yourself?

0 - Make decision myself — GO TO I26
1 - Will consult with someone } → CONTINUE
-8 - NR/DK } → CONTINUE

I25 Whom do you think you will consult regarding the weaning of your infant? How is this person related to you?

	PERSON WHO MAY BE CONSULTED	RELATIONSHIP TO PREGNANT MOTHER (Designation)
1.	______________________	______________________
2.	______________________	______________________

I26 If you wish to start or continue working for pay outside of your home,will you make that decision all by yourself or will you first consult with someone?

0 - Make decision myself — GO TO I28
1 - Will consult with someone } → CONTINUE
-8 - NR/DK } → CONTINUE

I27 Whom do you think you will consult regarding work outside of your home? How is this person related to you?

PERSON WHO MAY BE CONSULTED	RELATIONSHIP TO PREGNANT MOTHER (Designation)
1. ______	______
2. ______	______

I28 If you wish to start or continue using contraceptives after this pregnancy, will you make that decision all by yourself or will you first consult with someone?

0 - Make decision myself — GO TO NEXT BLOCK
1 - Will consult with someone } CONTINUE
-8 - NR/DK } CONTINUE

I29 Whom do you think you will consult regarding contraceptive practice? How is this person related to you?

PERSON WHO MAY BE CONSULTED	RELATIONSHIP TO PREGNANT MOTHER (Designation)
1. ______	______
2. ______	______

J. FOOD INTAKE RECALL: MOTHER

I would like you to tell me all about what you ate yesterday that is, from the time you woke up until you went to bed, including snacks.

J1 START WITH FIRST MEAL (OR SNACK) OF THE DAY.
CODE TYPE OF MEAL IN COL. J1 OF TABLE 13. IF RESPONDENT DOES NOT TAKE THREE REGULAR MEALS A DAY, ASK FOR THE TIME WHEN THE MEAL WAS TAKEN. ANY MEAL TAKEN BEFORE 10:00 A.M. IS BREAKFAST. LUNCH IS THE MEAL TAKEN BETWEEN 11:00 A.M. AND 2:00 P.M. SUPPER IS AFTER 4:00 P.M.

0 - before breakfast snack
1 - breakfast
2 - morning snack
3 - lunch
4 - afternoon snack
5 - supper
6 - evening snack
-8 - NR/DK

J2 What dishes did you have for (breakfast, lunch, supper, snacks)?

FILL UP COL. J2 OF TABLE 13. FOR EVERY DISH, USE A SEPARATE LINE(BOX).

FOR EVERY DISH EATEN ASK QUESTIONS J3 THRU J6

J3 What amount of the dish was eaten by you?

ENTER ANSWER IN COL. J3. USE THE FOLLOWING MEASURES: TABLESPOON (TBSP), TEASPOON (TSP), CUP (C), PIECES (PCS), MATCHBOX (MBX).

J4 CHECK WHETHER DISH IS INCLUDED IN THE FOOD TABLE.

IF YES, ENTER FCT CODE IN COL. J4 AND ASK J5 AND J6. IN CASES WHERE FOOD MAY BE WELL OR LOOSELY PACKED (RICE, CORN) OR WHERE FOOD MAY BE COMPOSED OF MUCH LIQUID AND LITTLE SOLID FOOD (OR VICE VERSA) LIKE UTAN BISAYA, ASK ALSO J8. THEN GO TO NEXT DISH.

IF NO, ENTER "O" IN COL. J4 AND THEN ASK J5 TO J9.

J5 How was the dish prepared?

1 - boiled
2 - fried
3 - sauteed
4 - broiled/roasted
5 - scrambled
6 - steamed
7 - baked
8 - processed
-8 - NR/DK
-9 - NA (raw)

ENTER CODE IN COL. J5 OF TABLE 13.

J6 Where was the dish prepared?

1 - home
2 - food store (carenderia, restaurant, bakery)
3 - feeding program
4 - ambulant food vendor
5 - other (SPECIFY) ____________
-8 - NR/DK
-9 - NA

ENTER CODE IN COL. J6 OF TABLE 13. FOR HOME-COOKED DISHES, ASK J7 TO J9. FOR CARENDERIA OR FEEDING PROGRAM OR AMBULANT FOOD VENDOR DISHES, GO TO CARENDERIA OR FEEDING PROGRAM OR AMBULANT FOOD VENDOR AFTER INTERVIEW AND ASK QUESTIONS J7 TO J9.

IF COL. J4 CONTAINS FOOD CODE, GO TO NEXT DISH OR, IF NECESSARY, ASK J8. IF CODE IN J4 IS "O", CONTINUE.

J7 What were the ingredients (food items) composing the dish?

ENTER ALL ITEMS IN COL. J7 OF TABLE 13. USE A SEPARATE LINE FOR EACH INDIVIDUAL FOOD ITEM.

IF THE SAME DISH WAS EATEN AT DIFFERENT MEALS, DO NOT WRITE "SAME" IN COLUMN J7 BUT SPELL OUT FOOD ITEMS EACH TIME THE DISH WAS EATEN.

J8 What were the specifics of these food items?

DESCRIBE THE FOOD ITEMS AS ACCURATELY AND CLEARLY AS TO FORM, KIND, COLOR, SIZE, CHARACTERISTICS THAT WILL IDENTIFY THE FOOD ITEM, i.e., WHAT KIND OF CEREAL? FRUIT? IF FISH, WHAT KIND? SIZE? PART? ETC. IF NECESSARY, USE FOOD PICTURES TO SPECIFY FOOD ITEM. ENTER ANSWER IN COL. J8 OF TABLE 13.

J9 What was the amount of the food item used when the dish was prepared? (IN TBSP, TSP, C, PCS, MBX)

ENTER ANSWER IN COL. J9 OF TABLE 13.

COLS. J10 AND J11 OF TABLE 13 WILL BE COMPLETED BY EDITORS.

Table 13. 24-Hour Food Intake Recall: Mother

MEAL CODE	NAME OF DISH	QUANTITY OF DISH EATEN	CODE OF DISH IN FCT	COOKING METHOD CODE	WHERE PREPARED	FOOD ITEMS DISH CONTAINS	FOOD ITEM DESCRIPTION	QUANTITY AMOUNT SIZE MEASURE	FOOD ITEM CODE	FOOD ITEM WEIGHT (GGRAMS)
(J1)	(J2)	(J3)	(J4)	(J5)	(J6)	(J7)	(J8)	(J9)	(J10)	(J11)

Table 13. 24-Hour Food Intake Recall: Mother (cont'd.)

MEAL CODE	NAME OF DISH	QUANTITY OF DISH EATEN	CODE OF DISH IN FCT.	COOKING METHOD CODE	WHERE PREPARED	FOOD ITEMS DISH CONTAINS	FOOD ITEM DESCRIPTION	QUANTITY AMOUNT SIZE MEASURE	FOOD ITEM CODE	FOOD ITEM WEIGHT (GRAMS)
(J1)	(J2)	(J3)	(J4)	(J5)	(J6)	(J7)	(J8)	(J9)	(J10)	(J11)

N. MOTHER'S ANTHROPOMETRY

N1 WEIGHT of mother (IN KILOGRAMS AND GRAMS): [ ][ ] KILOS [ ][ ][ ] GRAMS

N2 HEIGHT of mother (IN CENTIMETERS): [ ][ ][ ]

N3 ARM CIRCUMFERENCE of mother (IN CENTIMETERS): [ ][ ]

N4 SKINFOLD of mother:

INTERVIEWER: TAKE THREE MEASUREMENTS!

Measurement # 1 [ ][ ]

Measurement # 2 [ ][ ]

Measurement # 3 [ ][ ]

END OF INTERVIEW!

INTERVIEWER: THANK MOTHER FOR HER COOPERATION AND INFORM HER ABOUT THE APPROXIMATE TIME OF THE FOLLOWING INTERVIEW.

CEBU LONGITUDINAL HEALTH AND NUTRITION STUDY
2005 Follow-up Survey

IC's Questionnaire

ID1 Type of Survey: ☐☐

ID2 Current Barangay: ______________________ ☐☐☐
(See BARANGAY CODE LIST)

ID3 Current Stratum: 1 - Urban 2 - Rural ☐

ID4 Baseline ID Number: ☐☐☐ ☐☐☐☐ ☐☐☐
(Copy from 2002 Quest.) BRGY HHLD WMAN

ID5 2005 ID Number: ☐☐☐ ☐☐☐☐ ☐☐☐
(Office assigned) BRGY HHLD WMAN

ID6 Index Child's ID Number: ☐☐☐☐☐
(CPC/Computer assigned)

ID7 Name and Exact Address of Index Child in 2002 (or last visit) and 2005:

Name of Index Child: ______________________

2002 (or last visit) address: ______________________

2005 address: ______________________

____

ID8 Last Interview of Index Child: ☐ 2002 ☐ 1998

ID9 Whose household is interviewed in 2005? ☐

1 - Mom and Index Child GO TO ID11
3 - Index Child living separately from mom (Mom deceased/OM/Unlocated) CONTINUE
4 - Index Child deceased
5 - Index Child moved out of Metro Cebu >GO TO ID13
6 - Index Child refused/not located
11 - Index child mentally incapacitated
12 - Index child institutionalized
13 - Mother and Index child (but mom refused)
14 - Index child not contacted but did not refuse
15 - Index child cannot talk (mute; severe polio)

ID10 Who is Index Child living with? ☐

1 - Living alone (or with spouse as one nuclear family)
2 - Father
3 - Parent(s)-in-law
4 - Other relatives of IC
5 - Non-relatives
6 - Employer
-9 - IC deceased

ID11 Is Index Child living with partner/spouse? ☐

0 - No 1 - Yes

ID12 Is Index Child (or spouse of Index Child) the head of household? ☐

0 - No 1 - Index child 2 - Spouse of Index child

ID13 Sex of Index Child: 1 - Male 2 - Female

ID14 Age of Index Child: ___________

ID15 Name of Interviewer: ____

ID16 Date of Interview Completion:

MONTH DAY YEAR

CALL RECORD

SESSION. 1

CALL NO.	DATE	TIME		RESULTS (Use codes below) WRITE BLOCKS COMPLETED	APPOINTMENT MADE	
		Started	Finished		Date	Time

SESSION. 2

CALL NO.	DATE	TIME		RESULTS (Use codes below) WRITE BLOCKS COMPLETED	APPOINTMENT MADE	
		Started	Finished		Date	Time

RESULT CODES:
1 - Interview completed
2 - Interview partly completed, new appointment made
3 - Appointment made for interview later
4 - Refusal, no interview obtained
5 - No respondent at home
6 - Other (SPECIFY)

ID17 Total Number of Sessions Required to Complete Interview:

Other respondents during IC's interview

Line Number	Relationship to IC	Block(s) for which information was given	Block(s) for which other people were present during IC's interview

BLOCK X: SCREENING

IN 2002 (or last visit), INDEX CHILD LIVED: 1 - WITH MOTHER
2 - SEPARATED FROM MOTHER

X1 Is ___________________ (NAME OF INDEX CHILD) still living in this household? (the same household he/she had at last interview in 2002 or last visit)

0 -	No	CONTINUE
1 -	Yes	GO TO X9
-8 -	NR/DK	GO TO X5

X2 Why is __________________________ (NAME OF INDEX CHILD) no longer living in this household?

1 -	Died after last interview	CONTINUE
2 -	Moved elsewhere since 2002 (or last visit)	GO TO X5
-8 -	NR/DK	GO TO X5
-9 -	NA	

X3 When did _______________________ (NAME OF INDEX CHILD) die?
CODE RESPONSE IN MONTH AND YEAR

Month Year

-8 - NR/DK
-9 - NA

X4 What was the cause of death?
DESCRIBE: ____
-8 - NR/DK
-9 - NA

GO TO X8

X5 Where is __________________________ (NAME OF INDEX CHILD) currently living?

EXACT ADDRESS: ____

1 -	In Metro Cebu	5 -	Outside Cebu province, outside the country specify, ___________________________
2 -	Outside Metro Cebu, in Cebu province		
3 -	Outside Cebu province, not Manila/abroad	-8 -	NR/DK
4 -	Outside Cebu province, Manila	-9 -	NA

IF RESPONDENT DOES NOT KNOW, ASK FOR OTHER PERSON WHO MAY KNOW CURRENT ADDRESS OF INDEX CHILD

X6 What is __________________ (NAME OF INDEX CHILD) currently doing?

0 -	Not working or schooling	3 -	Working and schooling
1 -	Schooling	4 -	Other, specify ____________________________
2 -	Working	-8 -	NR/DK

X7 Marital status of index child

1 -	Never married	4 -	Widowed
2 -	Legally married	5 -	Separated
3-	Not legally married	-8 -	NR/DK

X8 TERMINATE INTERVIEW IF:

1 - Index Child dead
2 - Index Child moved out of Metro Cebu
3 - Index Child cannot be located in Metro Cebu
4 - Index Child cannot be located outside Metro Cebu (but in Cebu province)
5 - Index Child refused
6 - No available information about Index Child (incl. IC: institutionalized, not contacted, refused, incapacitated)

X9 OTHERWISE, DETERMINE IF:

MOM AND INDEX CHILD ARE LIVING TOGETHER, THEN GO TO A17;
INDEX CHILD IS LIVING SEPARATE FROM MOM, CONTINUE;
INDEX CHILD IS LIVING WITH EMPLOYER, CONTINUE AND ASK QUESTIONS A1 TO A14 PERTAINING ONLY TO INDEX CHILD AND TO ALL OF HIS/HER FAMILY MEMBERS LIVING WITH HIM/HER.

IN CASES WHERE IC IS NOT KNOWLEDGEABLE ENOUGH ABOUT HIS/HER NEW HOUSEHOLD, ASK FOR OTHER HOUSEHOLD MEMBERS WHO CAN RESPOND TO SPECIFIC SECTIONS IN THIS QUESTIONNAIRE.

BLOCK A: HOUSEHOLD COMPOSITION

A1 At present, how many persons are living with you in this household?

NO. OF PERSONS ☐☐

A2 How many of the people usually living here with you in this household are temporarily absent? IF NONE, CODE 00.

NO. OF PERSONS ☐☐

FOR ALL PERSONS IN THE HOUSEHOLD, INCLUDING THOSE TEMPORARILY ABSENT, ASK A3 THROUGH A14. RECORD RESPONSES IN TABLE A-1. BEGIN WITH HEAD OF HOUSEHOLD, WHO SHOULD HAVE LINE NUMBER 1, THEN CONTINUE WITH HIS/HER SPOUSE, UNMARRIED CHILDREN, MARRIED CHILDREN, HELPERS, ETC.

A3 What is his/her full name? ENTER NAME IN COL. A3

A4 How is he/she related to the household head? ENTER RESPONSE IN COL. A4 (CODES TO BE ASSIGNED BY EDITORS)

A5 How is he/she related to the index child? ENTER RESPONSE IN COL. A5 (CODES TO BE ASSIGNED BY EDITORS)

IF PERSON IS NOT A PART OF THE INDEX CHILD'S NUCLEAR FAMILY, CONTINUE, ELSE CODE -9 IN A6 AND GO TO A7.

A6 Is he/she paid in cash or in kind e.g., sent to school to do the household chores/family business?

0 - No
1 - Yes ENTER CODE IN COL. A6
-9 - NA

A7 IF NOT OBVIOUS TO INTERVIEWER, ASK: Is this person male or female?

1 - Male
2 - Female ENTER CODE IN COL. A7

A8 When was he/she born?
What month? ENTER MONTH IN FIRST COLUMN OF A8 AND
What year? FOUR DIGITS OF YEAR IN 2nd COLUMN OF A8

A9 How old was he/she on his/her last birthday? ENTER AGE IN COLUMN A9

A10 Has he/she resided in this household for the last six (6) months?
A RESIDENT IS DEFINED AS A PERSON WHO HAS LIVED IN THE HOUSEHOLD FOR THE LAST SIX MONTHS OR WHO HAS LIVED IN THE HOUSEHOLD FOR LESS THAN SIX MONTHS, BUT HAS NO OTHER PLACE OF RESIDENCE. INFANTS BORN INTO, OR PERSONS MARRIED INTO, THE HOUSEHOLD, ARE RESIDENTS REGARDLESS OF DATE THEY JOINED THE HOUSEHOLD.

IF RESPONSE IS YES, ENTER CODE 1 IN COL. A10 AND GO TO A11

IF RESPONSE IS NO OR NR/DK, ASK: Does he/she have a residence any place else?
IF NONE, ENTER CODE 3 (recent resident) IN COL A10
IF YES, ENTER CODE 2 (non- resident) IN COL. A10
-8 - NR/DK ENTER CODE -8 IN COL. A10

A11 What is the highest grade that he/she completed? ENTER RESPONSE IN COL. A11

-9 NA (For children under 6 years old)

A12 Is he/she currently studying in school (SY 2004-2005)? ENTER CODE IN COL. A12

0 - No -8 - NR/DK
1 - Yes -9 - NA (For children under 6)

A13 Is he/she currently working for pay (in cash or kind)? ENTER CODE IN COL. A13

0 - No
1 - Yes (GO TO BLOCK B IF THIS PERSON IS NOT THE IC, ELSE, GO TO A17)
-8 - NR/DK (GO TO BLOCK B IF THIS PERSON IS NOT THE IC, ELSE, GO TO A17)
-9 - NA (For HH members under 6)

A14 Did he/she work for pay in the last four months? ENTER CODE IN COL. A14

0 - No -8 - NR/DK
1 - Yes -9 - NA (For HH members under 6)

GO TO BLOCK B IF THIS PERSON IS NOT THE IC, ELSE, GO TO A17

FOR OFFICE EDITOR:
A15 CODE HOUSEHOLD TYPE ☐

A16 CODE LINE NUMBER OF INDEX CHILD ☐☐

TABLE A-1. HOUSEHOLD ROSTER (In case more than 19 persons are found in a household, staple additional HH Form to this one)

Line No.	Name	Relationship to Household Head	Relationship to Index Child	Paid	Sex	Date of Birth		Age	Resi-dent	Last Grade of School Completed	In School Now?	Work-ing Now?	Worked Past 4 Mos.?
		Description	Description			Mo.	Yr.						
	A3	A4	A5	A6	A7	A8		A9	A10	A11	A12	A13	A14

ASK A17 - A24 OF IC ONLY:

A17 What is your religion?

0 -	No religion	5 -	Moslem
1 -	Catholic	6 -	Buddhist
2 -	Protestant (Lutheran, Baptist, etc.)	7 -	Other (specify) ______________________________
3 -	Protestant (Born again)	-8 -	NR/DK
4 -	Iglesia ni Cristo		

A18 How often do you go to church?

0 -	Never	3 -	About once a week
1 -	Occasionally only	4 -	More often than once a week
2 -	About once a month	-8 -	NR/DK

A19 Do you consider yourself a religious person?

0 -	No	-8 -	NR/DK
1 -	Yes		

A20 Marital status of index child?

1 -	Never married	4 -	Widowed
2 -	Legally married	5 -	Separated
3 -	Not legally married	-8 -	NR/DK

A20a Were there any changes in your marriage since 2002 survey (or last visit)?

0 - Still never married
1 - Still living with same spouse in 2002/last visit
2 - Living with new spouse (legally married)
3 - Living with new spouse (not legally married)
4 - Widowed
5 - Separated
6 - Reunited with previous spouse
7 - Still widowed
8 - Still separated
9 - Others (Specify)______________________________________

A21 Are you a registered voter?

0 - No GO TO INSTRUCTION BEFORE A23
1 - Yes CONTINUE

A22 Did you vote in the last election (2004)?

0 - No ASK: Why not? ____
1 - Yes
-8 - NR/DK
-9 - NA

ASK QUESTIONS A23 AND A24 ONLY IF INDEX CHILD IS CURRENTLY MARRIED OR COHABITING. GO TO NEXT BLOCK IF INDEX CHILD IS NOT LIVING IN THE SAME HOUSEHOLD AS MOTHER, ELSE GO TO BLOCK F.

A23 What is the religion of your husband/partner?
SAME CODES AS IN A17

7 - Other (specify) ____
-8 - NR/DK
-9 - NA

A24 How often does he/she go to church?
SAME CODES AS IN A18

-9 - NA

IF INDEX CHILD IS LIVING IN THE SAME HOUSEHOLD AS MOTHER, GO TO BLOCK F

END OF BLOCK A

BLOCK B: ENVIRONMENTAL INFORMATION

B1 What is your usual source of drinking water? ☐

1 - MCWD piped supply (Metro Cebu Water District)
2 - Other municipal piped supply
3 - Tubewell, borehole, motorized pump with pipes
4 - Dug well fitted with pump
5 - Dug well without pump, bucket used
6 - Spring
7 - River
8 - Rainwater
9 - Mineral water/bottled water
10 - Other (specify) ____

B2 Where is this water source located? ☐

1 - Inside respondent's house
2 - In respondent's yard
3 - Not in house or yard, water delivered by vendor (someone paid to bring water to house)
4 - Not in house or yard, respondent or family member hauls water to house
-8 - NR/DK

B3 What type of toilet facility do you have? ☐

1 - Flush toilet ⎤
2 - Water-sealed toilet ⎥
3 - Latrine, antipolo ⎬ CONTINUE
4 - Open pit ⎦
5 - None (use field, canal, seashore) CODE -9 IN B4, GO TO B5
6 - Other (specify) GO TO B5
____
-8 - NR/DK

B4 Where is this located? ☐

1 - Inside respondent's house
2 - Neighbor's house
3 - Outside, private
4 - Outside, public
5 - Other (specify) ____
-8 - NR/DK

B5 What is your usual method of garbage disposal? ☐

1 - Collected by a garbage collector
2 - Burning
3 - Composting
4 - Dumped away from house
5 - Dumped around or near house
6 - Dumped in river/stream
7 - Other (specify) ____
-8 - NR/DK

B6 What type of lighting do you usually use? ☐

1 - Electricity
2 - Kerosene
3 - Oil
4 - LPG (e.g., Gasul, Shellane)
5 - Candle
6 - Other (specify) ____
-8 - NR/DK

B7 What fuel do you usually use for cooking? ☐

1 - Electricity
2 - Kerosene
3 - LPG (e.g., Gasul, Shellane)
4 - Wood/charcoal
5 - Other (specify) ____
-8 - NR/DK

B8 Where do you usually buy most of your food?

1 - Carbon/Taboan Market (main open markets)
2 - Other market in Metro Cebu, specify ____
3 - Supermarket (Name of supermarket: ____)
4 - Sari-sari store (neighborhood grocery stores)
-8 - NR/DK

☐

B9 How long (IN WALKING MINUTES) does it take you to walk to this place/store?

CODE NUMBER OF MINUTES — CONTINUE
-8 - NR/DK — CODE -8 IN B10-B11, GO TO B12
-9 - NA — CODE -9 IN B10-B11, GO TO B12

NUMBER OF MINUTES ☐ ☐ ☐

B10 If it's too far to walk, how long (IN MINUTES) does it take you to travel to the place/store?

CODE NUMBER OF MINUTES
-8 - NR/DK

NUMBER OF MINUTES ☐ ☐ ☐

B11 How much do you spend to travel to this place/store?

CODE IN PESOS
0 - Own vehicle, no pay
-8 - NR/DK

PESOS ☐ ☐ ☐

B12 Is house located along a busy road (where traffic is moderate to heavy)?

0 - No
1 - Yes

☐

B13 How would you describe the air quality in the neighborhood (street dust, fumes from cars/trucks, burning garbage, fumes from factories, etc.)?

1 - Fine, good
2 - Fresh
3 - Cool
4 - Polluted
5 - Smells bad/stinks
6 - No bad smell but dusty
7 - Warm
8 - Other description: ____

☐
☐

ENVIRONMENTAL ASSESSMENT

ALL QUESTIONS ARE TO BE ANSWERED BY INTERVIEWER BASED ON THE INTERVIEWER'S OBSERVATIONS OF THE RESPONDENT'S HOUSE OR THE AREA AROUND IT (TEN HOUSES CLOSEST TO THE RESPONDENT'S HOUSE).

B14 INTERVIEWER: OBSERVE, DO NOT ASK. Based on your judgment, how would you describe the air quality in the neighborhood (street dust, fumes from cars/trucks, burning garbage, fumes from factories, etc.)?

____ ☐

____ ☐

B15 What is the general condition of the area immediately around the house with respect to excreta removal? Is the house smelling?

1 - Heavy defecation in area
2 - Some defecation in area
3 - Very little excreta visible
4 - No excreta visible

☐

B16 What is the general condition of the neighborhood with respect to excreta removal?

1 - Heavy defecation in area
2 - Some defecation in area
3 - Very little excreta visible
4 - No excreta visible

☐

B17 What is the general condition of the neighborhood with respect to garbage disposal?

1 - Lots of uncollected garbage
2 - Some uncollected garbage
3 - Very little garbage
4 - No garbage visible

B18 Is the area in the house where the food is kept:

1 - Very clean
2 - Not so clean
3 - Filthy

B19 In what type of settlement does the respondent live?

INTERVIEWER: WHEN IN DOUBT, SPECIFY SITIO AND
DO NOT CODE: ____

1 - Urban squatter area
2 - Urban, congested and dirty
3 - Urban, less congested and dirty
4 - Urban, outskirts of city center (e.g., Camputhaw, Lahug, Guadalupe, Banilad)
5 - Rural town (poblacion)
6 - Rural barangay outside of poblacion
7 - Rural - remote (isolated sitio or single house)

B20 What is the area immediately around the respondent's house used for?

1 - Mostly residential houses
2 - Mostly commercial buildings
3 - Mostly open space, used for farming and/or livestock
4 - Mostly open space, not used
5 - Mostly factories/manufacturing/industrial buildings

B21 What is the general area around the respondent's house (within fifty meters) used for?

USE SAME CODES AS IN B20

B22 How many houses are very close (within fifty meters) to the respondent's house?

1 - One
2 - Two
:
20 - Twenty or more

B23 How many minutes does it take to walk to the house closest to the respondent's house?

ENTER RESPONSE IN MINUTES

0 - Less than one minute

B24 How long does it take to walk from the respondent's house to the nearest road?

ENTER RESPONSE IN MINUTES

-7 - Respondent lives on an island with no road GO TO B26

B25 What kind of road?

1 - National road
2 - Barangay road
3 - Feeder road

GO TO B27

B26 How long does it take for respondent to travel by boat from island (e.g. Caohagan) to the nearest road in next island (e.g. Mactan, Cebu)?

ENTER RESPONSE IN MINUTES GO TO B28

B27 How long does it take to walk to the nearest public transport (e.g. jeepney, bus, tricycle, boat)?

ENTER RESPONSE IN MINUTES

B28 Is the respondent's house connected to the electrical system (Visayan Electric Co. in Cebu City, Mandaue, Lapu-lapu City; some other public system) regardless as to whether it is used or not?

0 - No
1 - Yes

B29 Do any of the houses around the respondent's house have electrical service?

0 - No
1 - Yes

B30 Of what kind of material is the respondent's house constructed?

1 - Light - refers to house made of nipa or similar wood
2 - Mixed - refers to house made of cement and/or wood, but with nipa or similar materials for wall or roof
3 - Strong - refers to house made exclusively of cement and/or wood with galvanized iron roofing

B31 Overall, how would you rate the construction of the houses around the respondent's house?

1 - Mostly light (bamboo, nipa, cheap wood)
2 - Mostly mixed (wood with hollow blocks, cement)
3 - Mostly strong (hollow blocks, concrete, or good wood)

INTERVIEWER: ASSESS THE APPEARANCE OF THE HOUSE, THE CHILDREN, AND THE RESPONDENT (not too obviously!!)
USE TABLE BELOW AND ENTER RESPONSE CODES IN APPROPRIATE COLUMN

1 - Neat and tidy
2 - Not so neat and tidy
3 - Poorly kept, dirty, messy
-9 - NA (No children) For B33 only

B32	House/environs	
B33	Children	
B34	Index Child	

END OF BLOCK B

BLOCK C: HOUSEHOLD ASSETS

INTERVIEWER: ASK OF INDEX CHILD'S HOUSEHOLD. FOR INDEX CHILD WHO IS LIVING WITH EMPLOYER, ASK QUESTIONS C3, C11 THROUGH C23 PERTAINING TO INDEX CHILD'S AND HIS/HER FAMILY'S ASSETS IN THIS HOUSEHOLD.

READ TO RESPONDENT: **I will read a list of properties. Please tell me whether you have this or not.**

C1 Do you/does your household own this house you are living in?

0 - No
1 - Yes
-8 - NR/DK

C2 Do you/does your household own this land on which this house you're living in is built?

0 - No
1 - Yes
-8 - NR/DK

IF ANSWER TO BOTH C1 AND C2 IS "NO" (CODE 0), ASK C3 OF OTHER HOUSES/LANDS OWNED AND CONTINUE WITH C4. IF ANSWER TO EITHER C1 OR C2 IS "NR/DK" (CODE -8), ENTER -8 IN C3 AND CONTINUE WITH C4.

C3 At present, how much do you think is the value (in pesos) of all the houses and land that you own?

<u>ALTERNATIVE QUESTION</u>:

If you were to sell your houses and/or land, how much do you think their value would be (in pesos)?

-8 - NR/DK
-9 - NA (owns no house or land)

IF ANSWER IN C1 IS "YES", GO TO C7

C4 Are you renting this house or are you staying here for free?

1 - Rent CONTINUE
2 - Stay for free CODE -9 IN C5 AND GO TO C6
-8 - NR/DK
-9 - NA

C5 How much (in pesos) do you pay for rent for this house every month?

-8 - NR/DK CODE -9 IN C6 AND GO TO C7
-9 - NA

C6 If you were to rent this house, how much (in pesos) do you think you would have to pay for this house every month?

-8 - NR/DK
-9 - NA

C7 Is this the same house and location as in 2002 survey (or last visit)?

0 - No GO TO C9
1 - Yes CONTINUE
-8 - NR/DK
-9 - NA

C8 Have any additions or renovations been made since 2002 survey (or last visit)?

0 - No
1 - Yes
-8 - NR/DK
-9 - NA

C9 Excluding the bathroom or the toilet, how many rooms does your household occupy in this house?

CODE NUMBER OF ROOMS

C10 Is there a bathroom for your private use?

0 - No
1 - Yes, inside with tiled floor and/or walls
2 - Yes, inside with cement floor and/or walls
3 - Yes, inside, floor and/or walls not cemented/tiled
4 - Yes, outside with cement floor and/or walls
5 - Yes, outside with tiled floor and/or walls
6 - Yes, outside, floor and/or walls not cemented/tiled

C11 Does your household own any of the following vehicles? IF YES: How many?
ENTER NUMBER OF VEHICLES IN TABLE. CODE 0 IF NOT OWNED.

Bicycle		Truck/bus	
Bicycle with sidecar		Motorized boat	
Motorcycle/motorbike		Banca/raft	
Motorcycle with side car		Horse-drawn carriage	
Car		Farm vehicles (tractor)	
Jeep/jeepney/multicab		Other, specify ______________________	

C12 Does your household own any of the following animals? IF YES: How many?
ENTER NUMBER OF ANIMALS IN TABLE. CODE 0 IF NOT OWNED.

Chicken, ducks,etc.		Carabaos	
Goats		Horses	
Pigs		Other, specify ______________________	
Cows			

C13 Does your household own any of the following furniture? IF YES: How many?
ENTER NUMBER OF FURNITURES IN TABLE. CODE 0 IF NOT OWNED.

Living room set		Bed with mattress	
Dining room set		Bed without mattress	
Cabinet/bookshelf		Other, specify ______________________	

C14 Does your household own any of the following appliances? IF YES: How many?
ENTER NUMBER OF APPLIANCES IN TABLE. CODE 0 IF NOT OWNED.

Electric iron		VCR (Betamax, VHS, VCD/DVD)	
Electric fan		Cassette recorder	
Air conditioner		CD player	
Sewing machine		Stereo/Karaoke/Videoke	
Refrigerator		Computer, without internet access	
Gas (LPG)/electric stove		Computer, with internet access	
Gas (LPG)/electric range/oven		Washing machine	
Rice cooker		Vacuum cleaner/floor polisher	
Microwave oven		Kerosene stove	
Pressure cooker/turbo boiler/blender/other expensive specialized cooking gadgets		Digital camera/Video camera	
TV, without cable connection		Video games (playstation, X-box, etc.)	
TV, with cable connection		Other, specify ______________________	

C15 Does your household own equipments used in farming (e.g. threshers), fishing (e.g. fish nets) or for family business (e.g. photocopier)?

IF NONE, CODE 0. IF YES, ASK RESPONDENT TO IDENTIFY EQUIPMENT(S) AND ASK HOW MANY THEY OWN.

Type of equipment	Number
____________________	__________
____________________	__________
____________________	__________
____________________	__________
____________________	__________

C16 Did your household make any major purchase since 2002 survey (or last visit) (e.g. land, motorized vehicle, stereo, house, TV, refrigerator, cell phone, etc.)?

0 -	No	GO TO C18	☐
1 -	Yes	CONTINUE	
-8 -	NR/DK	GO TO C18	

C17 What did you purchase? (e.g. land, motorized vehicle, stereo, house, TV, refrigerator, cell phone, etc.)

1) ____________________ 4) ____________________ ☐

2) ____________________ 5) ____________________ ☐

3) ____________________ 6) ____________________

C18 Does your household own a business?

0 -	No	GO TO C21	☐
1 -	Yes	CONTINUE	

C19 What type of business?

INTERVIEWER: INQUIRE FROM RESPONDENT AND THEN DESCRIBE

BUSINESS MAY BE A STORE, A SHOP, A STALL IN A MARKET, SIDEWALK VENDING (e.g. cigarettes), ANYTHING INVOLVING AN EXCHANGE OF GOODS OR SERVICES FOR MONEY ON A REGULAR BASIS (NOT JUST ONCE).

DESCRIBE: ____

____

CODE WILL BE SUPPLIED BY OFFICE EDITORS ☐☐

-8 - NR/DK

-9 - NA (No business)

C20 How many employees are working in this business?

IF BUSINESS HAS EMPLOYEES (i.e. PERSONS WHOM THE HOUSEHOLD PAYS A WAGE OR SALARY IN EXCHANGE FOR LABOR) ENTER NUMBER OF EMPLOYEES IN BOX.

0 - No employees, unpaid family members only

-8 - NR/DK ☐☐

-9 - NA (No business)

C21 Does your household have a telephone (landline)?

0 -	None	☐
1 -	Yes, currently has a phone	
2 -	No, but has applied for phone connection	
-7 -	Yes, but currently disconnected	

C22 Does any member in your household have a cell phone?

IF NONE, CODE 0 AND GO TO C23. IF YES, ASK RESPONDENT WHO HAS CELL PHONE AND ENTER NAME AND LINE NUMBER OF HOUSEHOLD MEMBER.

Name	Line No.
____________________	________
____________________	________
____________________	________
____________________	________
____________________	________

C23 How would you compare your current economic condition to your economic condition in 2002 survey (or last visit)?

1 - Better off Why so? ____________________

2 - Worse off Why so? ____________________

3 - The same better condition
4 - The same worse condition
-8 - NR/DK

END OF BLOCK C

Table D-1. Record of Gainful Activities: Main and Secondary Jobs

LINE NO	NAME OF HOUSEHOLD MEMBER	STATUS	MAIN JOB								SECONDARY JOB								
			Job Description	Code	Status of farm job	Status of non-farm job	Hours worked past week	Bene-fits	TAXES	Go to	Has 2nd job now	Job Description	Code	Status of farm job	Status of non-farm job	Hours worked past week	Bene-fits	TAXES	Go to
D1		D2	D3		D4	D5	D6	D7	D8	D9	D10	D11		D12	D13	D14	D15	D16	D17

BLOCK D. MARKET ACTIVITIES OF RESIDENT HOUSEHOLD MEMBERS

SCREEN FOR INDEX CHILD WHO IS LIVING WITH EMPLOYER. IF SO, ASK BLOCKS D AND E PERTAINING TO IC AND HIS/HER FAMILY MEMBERS LIVING IN THE SAME HOUSEHOLD WITH HIM/HER.

FROM THE HOUSEHOLD ROSTER (TABLE A-1) COPY THE LINE NUMBERS AND NAMES OF RESIDENT HOUSEHOLD MEMBERS WHO ARE EITHER CURRENTLY WORKING (CODED 1 IN A13) OR WORKED IN THE PAST 4 MONTHS (CODED 1 IN A14) INTO TABLE D-1.

UNDER WORK STATUS COLUMN (COL. D2), WRITE "C" FOR THOSE CURRENTLY WORKING AND "P" FOR THOSE NOT CURRENTLY WORKING BUT WORKED IN THE PAST 4 MONTHS. FOR EACH NAME LISTED IN TABLE D-1, ASK D3 THROUGH D16. EXCEPT FOR COLUMNS D3 AND D11, ENTER ONLY CODES IN TABLE D-1.

IMMEDIATELY UPON COMPLETION OF TABLE D-1, STAPLE IT TO THE BLANK PAGE OF THIS QUESTIONNAIRE!!!

D3 What is/was his/her main job?

MAIN JOB IS THAT JOB ON WHICH A PERSON SPENDS THE MOST TIME.
WRITE DESCRIPTION OF JOB IN D3 BEFORE ENTERING ANY OF CODES SHOWN BELOW.
PLACE D3 CODES IN COLUMN PROVIDED.

1 -	Farming	CONTINUE
2 -	Fishing	CODE -9 IN COLUMN D4, THEN GO TO D5
3 -	Other	CODE -9 IN COLUMN D4, THEN GO TO D5
-8 -	NR/DK	
-9 -	NA	

INTERVIEWER: After having entered the codes for question D3 into Table D-1, enter into Col. D9 the **E number** indicated under Questions D4 and D5. This will help you in completing the E Block.

D4 What is/was the nature of his/her job?

1 -	Farm owner	GO TO D6 THROUGH D9 > THEN ASK E11 THROUGH E17 USING TABLE E-3A
2 -	Farm tenant	
3 -	Both owner and tenant	
4 -	Paid farm laborer	GO TO D6 THROUGH D9 > THEN ASK E1 ff., USING TABLE E-1 OR TABLE E-2
5 -	Unpaid family worker on family-owned farm (HAS NO INCOME!)	GO TO D6 THROUGH D9 > THEN ASK E11 ff., USING TABLE E-3A
-8 -	NR/DK	
-9 -	NA	

IF THE FARM IS OWNED BY THE FAMILY/HOUSEHOLD, ONLY ONE MEMBER, USUALLY THE HOUSEHOLD HEAD, CAN WORK AS FARM OWNER. ALL OTHER HOUSEHOLD MEMBERS WORKING ON THE FAMILY FARM ARE EITHER PAID FARM LABORERS OR UNPAID FAMILY WORKERS. IF THE FAMILY/HOUSEHOLD OPERATES THE FARM AS TENANT, THEN ALL FAMILY HOUSEHOLD MEMBERS WORKING ON THAT FARM ARE TENANTS.

ENTER CODE IN COLUMN D4. CODE -9 IN COLUMN D5. GO TO D6.

D5 What is the nature of his/her employment?

Code	Response	Instruction
1 -	Self-employed	CONTINUE THROUGH D9<br>IF D3 IS "2" (FISHING), GO TO E25, USE TABLE E-4<br>IF D3 IS "3" (OTHER), GO TO E29, USE TABLE E-5
2 -	Wage/salary worker	CONTINUE THROUGH D9 THEN GO TO E1 ff., USING TABLE E-1 OR TABLE E-2
3 -	Unpaid family worker in family-owned business (store, sewing) (HAS NO INCOME)	CONTINUE THROUGH D9<br>IF D3 IS "2" (FISHING), GO TO E25, USE TABLE E-4<br>IF D3 IS "3" (OTHER), GO TO E29 USING TABLE E-5
-8 -	NR/DK	
-9 -	NA	

ENTER CODE IN COLUMN D5. CODE IN D4 MUST BE -9. GO TO D6.

D6 During the workweek before this interview, how many hours did he/she spend on this job?

ENTER NUMBER OF HOURS DURING WEEK IN COLUMN D6.

-8 - NR/DK
-9 - NA (did not work last week)

D7 Do/Did you/he/she receive any employment benefits like SSS/GSIS, Philhealth (Medicare), PAG-IBIG in this job?

ENTER CODE IN COLUMN D7

0 - No
1 - Yes
-8 - NR/DK

D8 Do/Did you/he/she pay income or business tax for this job?

ENTER CODE IN COLUMN D8

0 - No
1 - Yes
-8 - NR/DK

D10 Does he/she currently hold a secondary job for which he/she is paid in cash or in kind to supplement his/her income?

A SECONDARY JOB IS A JOB ON WHICH A PERSON SPENDS TIME EITHER (a) AFTER ATTENDING TO HIS/HER MAIN JOB, OR (b) FOR A SHORTER DURATION THAN HIS/HER MAIN JOB, OR A COMBINATION OF BOTH.

Code	Response	Instruction
0 -	No	ASK NEXT ELIGIBLE PERSON, BEGIN WITH D3
1 -	Yes	CONTINUE
-8 -	NR/DK	ASK NEXT ELIGIBLE PERSON, BEGIN WITH D3
-9 -	NA	(Not currently working but worked during the last four (4) months)

ENTER CODE IN COLUMN D10 OF TABLE D-1.

D11 What is his/her secondary job?

WRITE DESCRIPTION OF JOB IN D11 BEFORE ENTERING ANY OF CODES SHOWN.
BE PRECISE IN JOB DESCRIPTION.
PLACE D11 CODE IN COLUMN PROVIDED.

1 -	Farming	CONTINUE
2 -	Fishing	CODE -9 IN COLUMN D12, THEN GO TO D13
3 -	Other	CODE -9 IN COLUMN D12, THEN GO TO D13
-8 -	NR/DK	
-9 -	NA	

INTERVIEWER: After having entered the codes for question D11 into Table D-1, enter into column D17 the **E number** indicated under Questions D12 and D13.This will help you in completing the E Block.

D12 What is the nature of his/her secondary job?

1 -	Farm owner	GO TO D14 THROUGH D17 THEN ASK E11 THROUGH E17 USING TABLE E3a
2 -	Farm tenant	
3 -	Both owner and tenant	
4 -	Paid farm laborer	GO TO D14 THROUGH D17 THEN ASK E1 ff., USING TABLE E-1 OR TABLE E-2
5 -	Unpaid family worker on family-owned farm (HAS NO INCOME!)	GO TO D14 THROUGH D17 THEN ASK E11 ff., USING TABLE E3a
-8 -	NR/DK	
-9 -	NA	

ENTER CODE IN COLUMN D12. CODE -9 IN COLUMN D13. GO TO D14

D13 What is the nature of his/her employment?

1 -	Self-employed	CONTINUE THROUGH D17, IF D11 IS "2" (FISHING), GO TO E25, USE TABLE E-4; IF D11 IS "3" (OTHER), GO TO E29, USE TABLE E-5
2 -	Wage/salary worker	CONTINUE THROUGH D17 THEN GO TO E1 ff., USING TABLE E-1 OR TABLE E-2
3 -	Unpaid family worker in family-owned business (store, sewing) (HAS NO INCOME)	CONTINUE THROUGH D17 IF D11 IS "2" (FISHING), GO TO E25, USE TABLE E-4; IF D11 IS "3" (OTHER), GO TO E29 USING TABLE E-5
-8 -	NR/DK	
-9 -	NA	

ENTER CODE IN COLUMN D13.

D14 During the workweek before this interview, how many hours did he/she spend on this secondary job?

ENTER NUMBER OF HOURS DURING WEEK IN COLUMN D14.

-8 - NR/DK
-9 - NA (Did not work on secondary job last week)

D15 Do/Did you/he/she receive any employment benefits like SSS/GSIS, Philhealth (Medicare), PAG-IBIG in this job?

ENTER CODE IN COLUMN D15

0 - No
1 - Yes
-8 - NR/DK

D16 Do/Did you/he/she pay income/business tax for this job?

ENTER CODE IN COLUMN D16

0 - No
1 - Yes
-8 - NR/DK

GO TO NEXT ELIGIBLE PERSON (D1) OR BLOCK E

END OF BLOCK D

BLOCK E. INCOME AND EXPENDITURES

DETERMINE FROM TABLE D-1 WHETHER A PERSON HOLDING TWO JOBS HAS JOBS OF THE SAME OR OF DIFFERENT TYPES.

For this purpose, compare the job status in column D4 (or D5) with that in column D12 (or D13). In case of SELF-EMPLOYMENT AND UNPAID FAMILY WORK, take into account also the codes in columns D3 and D11.

(a) If the two jobs are of the same type, add up the time spent on, and the income earned from, both jobs and enter the cumulative answers in that income table which is indicated.

(b) If the two jobs are of different types, treat each job separately. Determine first the income table into which information for the first (main) job has to be entered, and then the income table for the secondary job.

I. INCOME DERIVED FROM WAGE LABOR

FROM TABLE D-1 (GAINFUL ACTIVITIES), COPY LINE NUMBERS AND NAMES OF ALL RESIDENT HOUSEHOLD MEMBERS WHO ARE OR WERE ENGAGED, OVER THE LAST FOUR MONTHS, IN MARKET ACTIVITIES FOR WHICH THEY RECEIVED A WAGE OR SALARY IN EITHER CASH OR KIND. **WAGE LABORERS** ARE ALL THOSE WITH A CODE OF "4" IN COLUMN D4 AND/OR D12 OR A CODE OF "2" IN COLUMN D5 AND/OR COLUMN D13 IN TABLE D-1. FARM LABORERS AND FISHERMEN HIRED BY OTHERS AND WORKING FOR PAY ARE WAGE LABORERS! IF A PERSON HAS TWO WAGE/SALARY JOBS, ENTER HOURS WORKED AND WAGES RECEIVED FOR BOTH JOBS COMBINED, PROVIDED BOTH JOBS ARE EITHER WAGE-FOR-TIME OR WAGE-FOR-PIECE JOBS. IF TYPES OF JOBS ARE DIFFERENT, ENTER INFORMATION FOR EACH JOB IN APPROPRIATE TABLE. BEFORE ENTERING THE NAMES IN EITHER TABLE E-1 OR TABLE E-2, ASK E1.

E1 Is/Was he/she paid on a TIME basis or on a PER PIECE basis?

IF TIME BASIS, ENTER LINE NUMBER AND NAME IN TABLE E-1 AND ASK E2 TO E5a.
IF ON A PER PIECE BASIS, ENTER LINE NUMBER AND NAME IN TABLE E-2 AND ASK E6 TO E10a.
-8 - NR/DK
-9 - NA

E2 On the average, how many hours does/did he/she work during a usual work day?

ENTER RESPONSE IN COLUMN E2 OF TABLE E-1
-8 - NR/DK
-9 - NA

E3 On the average, how many days does/did he/she work in each week?

ENTER RESPONSE IN COLUMN E3 OF TABLE E-1
-8 - NR/DK
-9 - NA

E4 How much (IN PESOS) does/did he/she normally receive for a usual day's work, including allowances?

ENTER RESPONSE IN COLUMN E4 OF TABLE E-1
-8 - NR/DK
-9 - NA

E5 On the average, how many weeks does/did he/she work each year?

ENTER RESPONSE IN COLUMN E5 OF TABLE E-1
-8 - NR/DK
-9 - NA

E5a Is/Was the job in Table E-1 regular?

ENTER RESPONSE IN COLUMN E5a OF TABLE E-1
1 - Yes
2 - No, seasonal
3 - No, recently employed (last 3 months)
4 - No, not currently working but worked in past 4 months
-8 - NR/DK
-9 - NA

Table E-1. Income Derived from Wage Labor on a Time Basis

LINE NO.	NAME	AVE. NO. HRS. WORKED PER DAY	AVE. NO. DAYS PER WK. OF WORK	PESOS RECEIVED PER DAY'S WORK	AVE. NO. OF WKS. WORKED PER YEAR	STATUS OF JOB
	E1	E2	E3	E4	E5	E5a

IF PERSON HAS A SECONDARY JOB WHICH IS DIFFERENT FROM THE MAIN JOB, GO TO APPROPRIATE QUESTIONS. IF PERSON HAS ONLY ONE JOB, GO TO NEXT PERSON OR, AFTER LAST PERSON, GO TO E18.

E6 Usually, how many pieces of (CLOTHES, NECKLACES, BRACELETS, LAUNDRY, ETC.) does/did he/she finish in a day?

ENTER RESPONSE IN COLUMN E6 OF TABLE E-2
-8 - NR/DK -9 - NA

E7 How much (IN PESOS) is/was he/she paid per finished item?
ENTER RESPONSE IN COLUMN E7 OF TABLE E-2
-8 - NR/DK -9 - NA

E8 On the average, how many hours per day does/did he/she engage in piece labor?

ENTER RESPONSE IN COLUMN E8 OF TABLE E-2
-8 - NR/DK -9 - NA

E9 On the average, how many days per week does/did he/she engage in piece labor?

ENTER RESPONSE IN COLUMN E9 OF TABLE E-2
-8 - NR/DK -9 - NA

E10 On the average, how many weeks per year does/did he/she engage in piece labor?
ENTER RESPONSE IN COLUMN E10 OF TABLE E-2
-8 - NR/DK -9 - NA

E10a Is/Was the job in Table E-2 regular?
ENTER RESPONSE IN COLUMN E10a OF TABLE E-2
1 - Yes 4 - No, not currently working but worked in past 4 months
2 - No, seasonal -8 - NR/DK
3 - No, recently employed (last 3 months) -9 - NA

Table E-2. Income Derived from Labor Paid Per Piece

LINE NO.	NAME	AVE. NO. OF ITEMS FINISHED PER DAY	PESOS RECEIVED PER ITEM	AVE. NO. OF HRS. PER DAY	AVE. NO. OF DAYS WORKED PER WK.	AVE. NO. OF WKS WORKED PER YR.	STATUS OF JOB
		E6	E7	E8	E9	E10	E10a

IF PERSON HAS A SECONDARY JOB WHICH IS DIFFERENT FROM THE MAIN JOB, GO TO APPROPRIATE QUESTIONS. IF PERSON HAS ONLY ONE JOB, GO TO NEXT PERSON, OR, AFTER LAST PERSON, TO E18.

II. INCOME DERIVED FROM FARMING ACTIVITIES (EXCLUDING INCOME FROM LIVESTOCK)

FROM TABLE D-1, COPY INTO TABLE E-3A THE LINE NUMBERS AND NAMES OF ALL HOUSEHOLD MEMBERS WHO HAVE HAD FARM JOBS. THESE ARE ALL THOSE PERSONS WITH A CODE OF "1" IN COLUMN D3 AND/OR D11 AND WITH CODES "1" , "2" , "3" , OR "5" IN COLUMN D4 AND/OR D12. FOR ALL OF THESE PERSONS, ASK QUESTIONS E11 - E13a.

E11 On the average, how many hours per day does/did he/she work on the farm?
ENTER NUMBER OF HOURS PER DAY IN COLUMN E11 OF TABLE E-3A
-8 - NR/DK -9 - NA

E12 On the average, how many days per week does/did he/she work on the farm?
ENTER NUMBER OF DAYS PER WEEK IN COLUMN E12 OF TABLE E-3A
-8 - NR/DK -9 - NA

E13 On the average, how many weeks per year does/did he/she work on the farm?
ENTER NUMBER OF WEEKS PER YEAR IN COLUMN E13 OF TABLE E-3A

E13a Is/Was the job in Table E-3A regular?
ENTER RESPONSE IN COLUMN E13a OF TABLE E-3A

1 - Yes
2 - No, seasonal
3 - No, recently farming (last 3 months)
4 - No, not currently farming but worked in past 4 months
-8 - NR/DK
-9 - NA

IF THE FAMILY OPERATES THE FARM AS A FAMILY FARM, OR AS TENANTS, ASK QUESTIONS E14 THROUGH E17 ONLY OF THE HEAD OR THE ELDEST MEMBER OF THAT FAMILY. IF A FAMILY OR HOUSEHOLD MEMBER CULTIVATES ANOTHER FARM INDEPENDENTLY OF THE FAMILY, ASK ALL QUESTIONS OF THIS MEMBER.

E14 What were the major crops that he/she planted in the past 12 months?
USE ONE LINE FOR EACH MAJOR CROP
-8 - NR/DK -9 - NA

ENTER RESPONSE IN COLUMN E14 OF TABLE E-3A

E15 On the average, how much did he/she spend in the past 12 months for seedlings, fertilizer, tools, hired labor, insecticides, etc.?
ENTER RESPONSE, IN PESOS, IN COLUMN E15 OF TABLE E-3A
-8 - NR/DK -9 - NA

E16 How much did he/she receive for the portion of the produce that was sold?
ENTER RESPONSE, IN PESOS, IN COLUMN E16 OF TABLE E-3A
-8 - NR/DK -9 - NA

E17 If he/she sold that portion of the harvest that he/she set aside for home consumption, how much would he/she have received?
ENTER RESPONSE, IN PESOS, IN COLUMN E17 OF TABLE E-3A
-8 - NR/DK -9 - NA

Table E-3A. Income Derived from Farming, Excluding Livestock

LINE NO.	NAME	WORKING TIME				MAJOR CROPS	EXPEN-SES (PESOS)	MONEY VALUE OF CROP	
		HRS. PER DAY	DAYS PER WEEK	WKS. PER YEAR	STAT. OF JOB			SOLD	HOME USE
		E11	E12	E13	E13a	E14	E15	E16	E17

III. INCOME DERIVED FROM LIVESTOCK RAISING

QUESTION E18 AND, IF APPLICABLE, ALL OTHER QUESTIONS RELATED TO LIVESTOCK RAISING, ARE TO BE ASKED OF ALL HOUSEHOLDS REGARDLESS OF WHETHER THEY OPERATE A FARM OR NOT.

E18 Is there a member of your household who has been engaged in raising livestock (carabao, cow, goat, duck, chicken, and others) in the past 12 months?
ASK FOR NAME OF HOUSEHOLD MEMBER AND ENTER IT IN TABLE E-3B TOGETHER WITH HIS/HER LINE NUMBER SHOWN IN TABLE A-1. ☐

0 - No GO TO E25 -8 - NR/DK GO TO E25
1 - Yes CONTINUE -9 - NA GO TO E25

ASK QUESTIONS E19-E21 OF ALL PERSONS LISTED IN TABLE E-3B

E19 On an average working day, how many hours does/did he/she work with the livestock?
ENTER NUMBER OF HOURS PER DAY IN COLUMN E19 OF TABLE E-3B
-8 - NR/DK -9 - NA

E20 On an average work week, how many days does/did he/she work with the livestock?
ENTER NUMBER OF DAYS PER WEEK IN COLUMN E20 OF TABLE E-3B
-8 - NR/DK -9 - NA

E21 On the average, how many weeks in the past 12 months does/did he/she work with the livestock?
ENTER NUMBER OF WEEKS PER YEAR IN COLUMN E21 OF TABLE E-3B
-8 - NR/DK -9 - NA

E21a Is/Was the livestock raising regular?
ENTER RESPONSE IN COLUMN E21a OF TABLE E-3B
1 - Yes 4 - No, not currently raising but worked in past 4 months
2 - No, seasonal -8 - NR/DK
3 - No, recently raising (last 3 months) -9 - NA (Livestock raised by non-hhold member/hired)

IF THE LIVESTOCK RAISING IS A FAMILY OPERATION, ASK QUESTIONS E22 THROUGH E24 ONLY OF THE HEAD OR THE ELDEST MEMBER OF THE FAMILY. IF ANY FAMILY OR HOUSEHOLD MEMBER RAISES LIVESTOCK ELSEWHERE INDEPENDENTLY OF THE FAMILY OR HOUSEHOLD, ASK ALL QUESTIONS OF THIS HOUSEHOLD MEMBER.

E22 How much do you think did he/she spend in raising livestock for the past 12 months (for purchasing, feeding, treating, etc.)?
ENTER RESPONSE IN PESOS IN COLUMN E22 OF TABLE E-3B
-8 - NR/DK -9 - NA

E23 What was his/her total income from the sale of any livestock or livestock products in the past 12 months?
ENTER RESPONSE, IN PESOS, IN COLUMN E23 OF TABLE E-3B
-8 - NR/DK -9 - NA

E24 If he/she sold the livestock or livestock products which he/she set aside for consumption in the past 12 months, how much do you think he/she would have received?
ENTER RESPONSE, IN PESOS, IN COLUMN E24 OF TABLE E-3B
-8 - NR/DK -9 - NA

Table E-3B. Income Derived from Raising Livestock

LINE NO.	NAME	WORKING TIME				EXPENSES (PESOS)	MONEY VALUE OF LIVESTOCK	
		HRS. PER DAY	DAYS PER WEEK	WKS. PER YEAR	STATUS OF JOB		SOLD	HOME USE
		E19	E20	E21	E21a	E22	E23	E24

IV. INCOME DERIVED FROM FISHING ACTIVITIES

FROM TABLE D-1, COPY LINE NUMBERS AND NAMES OF ALL RESIDENT HOUSEHOLD MEMBERS WHO EITHER ARE OR WERE ENGAGED IN FISHING OVER THE PAST FOUR MONTHS. THESE ARE ALL THOSE PERSONS WITH A CODE OF "2" IN COLUMN D3 AND/OR COLUMN D11 AND A CODE OF "1" OR "3" IN COLUMN D5 AND/OR COLUMN D13. FISHERS HIRED BY OTHERS AND WORKING FOR PAY ARE WAGE WORKERS. IF FISHING IS A FAMILY/HOUSEHOLD ACTIVITY, ASK E25 THROUGH E27a OF ALL MEMBERS LISTED IN TABLE E-4, BUT ASK QUESTION E28 OF ONLY ONE MEMBER, THE ONE IN CHARGE OF THE FAMILY/HOUSEHOLD FISHING OPERATIONS.

E25 On an average working day, how many hours a day does/did he/she go fishing?

ENTER NUMBER OF HOURS PER DAY IN COLUMN E25 OF TABLE E-4
-8 - NR/DK
-9 - NA

E26 On the average, how many days per week does/did he/she usually go fishing?

ENTER RESPONSE IN COLUMN E26 OF TABLE E-4
-8 - NR/DK
-9 - NA

E27 On the average, how many weeks in a year does/did he/she usually go fishing?

ENTER RESPONSE IN COLUMN E27 OF TABLE E-4
-8 - NR/DK
-9 - NA

E27a Is/Was the job in Table E-4 regular?

ENTER RESPONSE IN COLUMN E27a OF TABLE E-4
1 - Yes
2 - No, seasonal
3 - No, recently fishing (last 3 months)
4 - No, not currently fishing but worked in past 4 months
-8 - NR/DK
-9 - NA

E28 How much is/was his/her usual net income out of a day's catch, including that portion of the catch which the household itself consumed? (EXCLUDING EXPENSES)

ENTER RESPONSE IN COLUMN E28 OF TABLE E-4
-7 - unpaid work
-8 - NR/DK
-9 - NA

Table E-4. Income Derived from Fishing Activities

LINE NO.	NAME	WORKING TIME				USUAL DAILY NET INCOME (IN PESOS)
		HRS. PER DAY	DAYS PER WK.	WEEKS PER YR.	STATUS OF JOB	
		E25	E26	E27	E27a	E28

V. INCOME DERIVED FROM SELF-EMPLOYMENT

FROM TABLE D-1, COPY LINE NUMBERS AND NAMES OF ALL HOUSEHOLD MEMBERS WHO ARE OR WERE SELF-EMPLOYED OR WORKING AS UNPAID FAMILY WORKERS IN A FAMILY-OWNED BUSINESS DURING THE PAST FOUR MONTHS. THESE ARE ALL PERSONS WITH A CODE OF “1” OR “3” IN D5 AND/OR COLUMN D13. E.G., IF A FAMILY/HOUSEHOLD OPERATES A STORE IN WHICH SOME MEMBERS WORK AS UNPAID FAMILY WORKERS, ASK E29 THROUGH E34a OF ALL MEMBERS WORKING IN THE STORE, BUT E35 ONLY OF THE MEMBER IN CHARGE OF THE STORE.

E29 Does he/she work in a family-owned business (e.g., sari-sari store, sewing business)?
ENTER RESPONSE IN COLUMN E29 OF TABLE E-5
0 - No GO TO E32 -8 - NR/DK
1 - Yes CONTINUE -9 - NA

E30 What is this business? (sari-sari store, sewing, barber, etc.)
SPECIFY (DO NOT CODE). ENTER RESPONSE IN COL. E30 OF TABLE E-5
-8 - NR/DK -9 - NA

E31 Is the enterprise located at home?
ENTER RESPONSE IN COLUMN E31 OF TABLE E-5
0 - No -8 - NR/DK
1 - Yes -9 - NA

E32 How many hours does/did he/she work in a usual day?
ENTER RESPONSE IN COLUMN E32 OF TABLE E-5
-8 - NR/DK -9 - NA

E33 How many days in a week does/did he/she usually work?
ENTER RESPONSE IN COLUMN E33 OF TABLE E-5
-8 - NR/DK -9 - NA

E34 How many weeks in a year does/did he/she usually work?
ENTER RESPONSE IN COLUMN E34 OF TABLE E-5
-8 - NR/DK -9 - NA

E34a Is/was the business regular?
ENTER RESPONSE IN COLUMN E34a OF TABLE E-5
1 - Yes 4 - No, not currently working but worked in past 4 months
2 - No, seasonal -8 - NR/DK
3 - No, recently employed (last 3 months) -9 - NA

E35 How much on the average is/was his/her daily net income? (EXCLUDE EXPENSES)
ENTER RESPONSE IN COLUMN E35 IN TABLE E-5
-7 - Unpaid work -9 - NA
-8 - NR/DK

GO TO NEXT PERSON LISTED OR, IF LIST IS COMPLETED, TO E36

Table E-5. Income Derived from Self-Employment

LINE NO	NAME	WORK IN FAM. BUS.	TYPE OF BUSINESS	BUS. AT HOME	AVE. NO. HRS/ DAY	AVE. NO. DAYS/ WEEK	AVE. WKS/ YEAR	STAT. OF JOB	AVE. DAILY NET INC. (IN PESOS)
		E29	E30	E31	E32	E33	E34	E34a	E35

VI. HOUSEHOLD INCOME DERIVED FROM OTHER SOURCES

E36 Does/did your household or any of its members have other sources of income?

0 -	No	CODE -9 IN E37 AND GO TO E38
1 -	Yes	CONTINUE
-8 -	NR/DK	CODE -8 IN E37 AND GO TO E38

E37 What are these sources of income? How much was received from each source in the past12 months?
IF NONE, CODE 00

Rent from agricultural/commercial land

Income from boarders/lodgers, house rental

Pensions, dividends, bonuses, savings interest

Cash remittances from children, parents, other relatives, friends or anyone else

Cash remittances from spouse abroad/sustento

Loans, donations

Winnings (masiao, lotto, sabong)

Income from home gardening

Others, specify ____

E38 Did you or your household receive any income in kind (food or clothing) from children, parents, relatives, friends or anyone in the past 12 months?

0 -	No	CODE -9 IN E39, GO TO E40
1 -	Yes	CONTINUE
-8 -	NR/DK	CODE -8 IN E39, GO TO E40

E39 What was the approximate value (IN PESOS) of this income in kind in the past 12 months?

-8 -	NR/DK	PESOS
-9 -	NA	

VII. HOUSEHOLD EXPENDITURES

==

INTERVIEWER: HOUSEHOLD EXPENDITURES ARE DIVIDED INTO WEEKLY (FOOD), MONTHLY (HOUSING, TRANSPORTATION, ETC.), AND ANNUAL (i.e. LESS THAN MONTHLY) OCCURRING EXPENSES (SCHOOLING, CLOTHING, TAXES, DURABLE GOODS, ETC.). ROUND AMOUNTS PAID TO THE CLOSEST FULL PESO.

IN CASE ITEMS LISTED UNDER WEEKLY OR MONTHLY OR ANNUAL ARE PAID MORE OR LESS REGULARLY IN OTHER TIME INTERVALS, MAKE A NOTE IN THE QUESTIONNAIRE AFTER THE ITEM, e.g. QUARTERLY.

==

1. WEEKLY EXPENSES:

Usually, how much is spent by your household each week for:

NO.	ITEM	PESOS
E40	**Food** (cereal, root crops, fish, meat, egg, milk and dairy products, vegetables, nuts and beans, fruits, oil, beverages, condiments/spices, bread, ready-cooked foods)	
E41	**Alcoholic beverages** (beer, palm wine, rum, gin, etc.)	
E42	**Tobacco**, cigarettes, 'abano', etc.	
E43	**Allowance for children/husband/wife**	

2. MONTHLY EXPENSES:

Each month, how much does your household spend for:

NO.	ITEM	PESOS
E44	**Household expenses** (rent, electricity, gas, water, wood, telephone, etc.)	
E45	**Cellular phone cards and accessories**	
E46	**TV cable access**	
E47	**Internet fee**	
E48	**Laundry detergent/bath soap,** toothpaste, toilet paper, cosmetics, etc.	
E49	**Household help**	
E50	**Transportation/fare** (public transport, gasoline or vehicle maintenance)	
E51	**Reading materials** (newspapers, magazines, etc.)	
E52	**Recreation** (movies, VHS/VCD/DVD or Betamax tapes rental/purchases, 'masiao', lotto, etc.)	
E53	**Loan(s)**	

3. EXPENSES FOR THE PAST YEAR (PAST TWELVE MONTHS):

NO.	ITEM	PESOS
E54	**House materials/land purchase**	
E55	**School expenses** (enrollment, matriculation/tuition fees, PTA/BOY/GIRL Scouts, Vocational course, school materials/books, uniforms, etc.)	
E56	**Medical expenses** (hospital, health center, doctor, traditional midwife, traditional healer, medicines, etc.)	
E57	**Clothing**, shoes and accessories (cloth, clothes, shoes, socks, hats, etc.)	
E58	**Durable goods** (vehicles, appliances, household furniture, kitchen equipment, jewelries, sports equipment, camera, watch, etc.)	
E59	**Cellular phones**	
E60	**Personal computer**	
E61	**Taxes and insurances** (income tax, property/realty tax, vehicle tax, accident insurance, life insurance, educational plan, pension plan, memorial plan, health insurance, etc.)	
E62	**Parties and other gatherings** (feasts, weddings, birthdays, baptisms, funerals, Christmas, All Soul's Day, etc.)	

E63 Are there other customary weekly, monthly or yearly household expenses?

0 - No GO TO NEXT BLOCK
1 - Yes

E64 If yes, what are these? INDICATE ALSO MODE OF PAYMENT: weekly, monthly, annually, etc.

ITEM # 1:____

ITEM # 2:____

-8 - NR/DK

-9 - NA

E65 How much? (ENTER PESOS IN BOXES TO THE RIGHT)

-8 - NR/DK ITEM # 1:

-9 - NA ITEM # 2:

END OF BLOCK E

BLOCK F: LIFE HISTORY, EDUCATION, EMPLOYMENT, FERTILITY

BLOCK F IS TO BE ASKED OF ALL INDEX CHILDREN REGARDLESS OF WHETHER THEY LIVE WITH THEIR MOTHERS OR NOT

I. The Life History Matrix

The Life History Matrix (LHM) is an instrument used for recording and sequencing various events in a person's life. Instead of the usual questionnaire format, a matrix is used as the interview schedule (Table F-1). Across the top of this matrix are the events (or behavioral categories) of interest and the first column on the left of the matrix represents the Index Child's single years of age from birth through current age. The interview is conducted by filling in the appropriate cells of the age-by-event matrix with information given by the Index Child.

We are recording 9 major aspects of the Index Child's life: 1) education, 2) occupation, 3) residence, 4) romantic relationship, 5) sexual experience, 6) pregnancy, 7) family planning, 8) marriage, and 9) major illnesses. We want to know how changes in one behavioral category relate to changes in other categories. The information recorded on the LHM should allow the researchers to determine the ages at which events occurred, the typical sequencing of events in relation to other events in the same behavioral category, and the typical sequencing of events in relation to events within other behavioral categories.

Administering the LHM

Review chronologically with the Index Child the events that occurred to him/her in the past, from age "0" (birth) up to the current age. Only changes within each behavioral category need to be recorded. For this reason, some of the matrix cells for a given interview will remain empty.

If it is clear that there were no events for several years within a category, **a line may be drawn vertically down a column from one event to the next one.**

The general instruction for the interview is to take one area (column) of the LHM as a "focus". Then, follow the sequence of events in this area and, for every change in it, relate those changes to changes in other areas. The area selected as a point of reference will vary according to the stage in the life cycle and also according to the IC's particular history. For example, begin by following IC's educational history; but at a later stage in the interview, occupation may be a better key to other events in the life history.

All events are linked to the age at which they occurred. In some instances it will be easier for IC to remember the year of an event than his/her age at the time. You should be able to reconcile these two temporal measures.

In some cases IC may have experienced more than one event during the same year or at a particular age, either in one column only or in different columns. In these situations, it will be necessary to **indicate the sequence of the events during the interview by writing and circling a number above the appropriate phrase.** If the sequence of events is unclear, proper ordering must be checked with IC.

By moving back and forth across the several substantive areas with the Index Child, events missed earlier in the interview as well as inconsistencies among earlier responses, can be discovered and corrected.

Specific procedure and ideal sequences of questions will vary from one Index Child to the next. The area of initial focus in the life history of a person, say, 18 years old who is in college, might be different from that of another person of the same age who had completed only six years of schooling but who had many changes of residence or jobs. Areas of focus will also differ for Index Child who best recall changes in terms of their family's history, versus others who will prefer to anchor their recall in relation to their own work or schooling histories.

What to record

A. Education

Entrances and exits from schooling, regardless of what type of schooling (preschool, vocational, etc.) should be recorded. Also record the name of school, the level of schooling, e.g. grade four, second year high school, first year college, etc. Ask about the type of course taken by IC when in college. More importantly, for each type of schooling, inquire whether IC is/was enrolled full time or part time and whether the school is public, private, sectarian, coed or otherwise.

If IC dropped out of school, specify the number of months IC attended before dropping out. The reason for dropping out/stopping school should also be recorded. Also, one should probe explicitly as to why IC did not go on to the next grade. If IC had stopped for a few weeks or more but returned and continued the same grade/year in the same term, no stop should be recorded. But if IC returned after some duration to re-start the same grade/year again, a stop and a start must be recorded.

In asking about IC's education, you should not assume that one schooling excludes another. There may be cases where IC attended both academic school and vocational school at the same time.

B. Occupation

Occupation or job refers to any activity for which IC receives remuneration either in cash or in kind. Distinguish between main job and secondary job. Main job is the job on which IC spends the most time compared with his/her other jobs. Secondary job is the job on which IC spends time after attending to his/her main job. IC may have one or more secondary jobs. All jobs should be described precisely.

IC's place of work should be asked, i.e. if it is at home, near home, away from home or ambulant. The name or type of firm or company and whether it is public or private should also be noted.

The beginning and stopping of jobs should also be recorded. If IC changed to a new job (a completely different activity) within the year, this should also be recorded. The reason for quitting a job should be recorded as well.

C. Residence

A change of residence is to be recorded when it happened. Also record the number of months IC stayed in one barangay especially for the short moves. Residence should be distinguished whether it is in the city, poblacion or barrio (to get urban-rural distinction) or abroad. The reason for the change of residence should also be recorded. It should also be specified with whom the respondent lived (e.g., parents, spouse, employer) for every change of residence.

D. Romantic relationship

Record all romantic relationships that IC had experienced and the age of his/her boyfriend or girlfriend. Ask for ages at first crush; first date and first courted someone/first courted by someone. The duration of the relationships should also be recorded. Don't fail to probe into relationships with the same sex and record this as such. Ask about main reason for a break-up.

E. Sexual experience

Inquire about any sexual experiences that IC may have had in a relationship and probe for any sexual experiences outside a relationship. Link this with pregnancy and family planning experiences.

F. Pregnancy/Fertility

Any pregnancy, including current pregnancy, is to be recorded (record as 1st pregnancy, 2nd pregnancy, etc.). Specify whether each pregnancy ended in a miscarriage (whether spontaneous or induced), stillbirth, or a live birth, and whether it is a single birth or a multiple birth (twins, triplets, etc.). The death of a child should also be noted.

G. Family planning

Ask about any family planning method used by IC (or the couple) regardless of the duration of use. When FP method was first tried, type of FP method used, switching to other methods, and discontinuation of use should all be noted. The reasons for the use of FP, switching to other methods, or discontinuation should also be specified. Be sure to probe for FP use even outside marriage or cohabitation.

H. Marriage or cohabitation

Marriage refers to any arrangement in which IC lived or is living with a man/woman as her husband/his wife. Thus, informal or consensual unions as well as legal marriages are included. This also includes cohabitation with same sex (specify if this is so). Also indicate whether it is IC's first marriage/cohabitation, the second, etc. Ask why they decided to get married or cohabit.

The marriage information to be recorded includes the point in time (age) at which entrance to marriage occurred and all periods of living apart from spouse/partner, whether short-term (less than six months) or long term (more than six months). Living apart refers to occasional absences of spouse due to work, travel, education, military service, etc.

Periods of separation, that is formal or informal due to, e.g., discord in the family where at least one spouse does not want to live with the other, and time spent between unions are also recorded. If separation, reunion to the same spouse, remarriage after a separation or widowhood occurred in IC's life, these should be recorded as well. Reasons for separation or termination of a marriage or union must be specified.

Probe and record if IC had experienced elopement and when this had happened.

I. Major illness

Record all major illnesses and disabilities that IC may have experienced in his/her life. Include details related with the illness such as whether the illness is chronic, hereditary, or if IC was hospitalized, underwent surgery, etc. For chronic illness or disability, ask when was the onset and the duration of such illness or disability.

One should never assume that he/she knows the order of events. The Index Child should be asked.

INTERVIEWER: IF IC WAS INTERVIEWED IN 2002, UPDATE THE NINE EVENTS (EDUCATION, OCCUPATION, RESIDENCE, ROMANTIC RELATIONSHIP, SEXUAL EXPERIENCE, PREGNANCY/FERTILITY, FAMILY PLANNING, MARRIAGE OR COHABITATION, AND MAJOR ILLNESS).

II. SCHOOLING OF INDEX CHILD

AS A GENERAL RULE, INFORMATION ALREADY OBTAINED IN THE LHM NEED NOT BE ASKED AGAIN UNLESS IT IS INADEQUATE OR AMBIGUOUS. JUST COPY OR RECORD PERTINENT RESPONSES IN THE SPACES PROVIDED IN THE QUESTIONS BELOW. THESE QUESTIONS ARE MARKED WITH AN @ SIGN BEFORE THE QUESTION NUMBER. IF PREFERRED, YOU MAY VERIFY THE ANSWERS WITH IC. ALSO, THERE MAY BE QUESTIONS THAT HAVE ALREADY BEEN ANSWERED IN 2002 AND NEED NOT BE ASKED AGAIN. THESE QUESTIONS ARE MARKED WITH 3 ASTERISKS (***) AND ARE PRE-CODED IF APPLICABLE.

F2.0 Highest grade completed: ____________________ ☐☐

@F2.1 Are you currently in school (the point of reference is school year 2004-2005)? ☐

0 -	No	CONTINUE
1 -	Yes	GO TO F2.15
2 -	No, enrolled but dropped out	CONTINUE
3 -	No, graduated from college	GO TO F2.16
4 -	No, but enrolled in first semester	CONTINUE
5 -	Yes, but not enrolled in first semester	GO TO F2.15
-8 -	NR/DK (Not sure)	

@F2.2 Why are you not currently in school/Why did you drop out of school?

FOR THIS INTERVIEW "DROPPING OUT" IS DIFFERENTIATED FROM "STOPPED SCHOOLING". "DROPPING OUT" MEANS LEAVING SCHOOL BEFORE A GRADE/YEAR IS COMPLETED, "STOPPED SCHOOLING" MEANS NOT CONTINUING TO THE NEXT GRADE LEVEL AFTER FINISHING A GRADE LEVEL.

REASON: ____________________ ☐☐

____

-8 - NR/DK
-9 - NA

@F2.3 What was the last grade/year you were enrolled in? ☐☐

1 -	Grade 1	10 -	Third Year High School
2 -	Grade 2	11 -	Fourth Year High School
3 -	Grade 3	12 -	First Year College
4 -	Grade 4	13 -	Second Year College
5 -	Grade 5	14 -	Third Year College
6 -	Grade 6	15 -	Fourth Year College
7 -	Grade 7	16 -	Fifth Year College
8 -	First Year High School	-8 -	NR/DK
9 -	Second Year High School	-9 -	NA

@F2.4 Did you complete the last grade/year you were enrolled in? ☐

0 - No
1 - Yes
-8 - NR/DK
-9 - NA

@F2.5 In what school/college/university were you enrolled before?

RECORD NAME OF SCHOOL: ☐☐☐

____________________ ☐

1 - Private, not coed, Catholic (e.g., STC, SHS, USC-HS)
2 - Private, not coed, not Catholic (e.g., Buddhist)
3 - Private, coed, Catholic
4 - Private, coed, not Catholic (lay) (e.g., UV)
5 - Public school
-8 - NR/DK
-9 - NA

F2.6 Was there a conscious decision by you or your family for you to drop out or stop schooling? ☐

0 -	No	GO TO F2.9
1 -	Yes	CONTINUE
-8 -	NR/DK	
-9 -	NA	

F2.7 Who were the people contributing to this decision? MULTIPLE RESPONSES ALLOWED

1 -	IC himself/herself	7 -	Other male relative
2 -	Spouse/Partner of IC	8 -	Other female relative
3 -	Father	9 -	Non-household member, specify relationship ____________
4 -	Mother		
5 -	Father-in-law	-8 -	NR
6 -	Mother-in-law	-9 -	NA

GO TO F2.9 IF ONLY ONE (1) PERSON DECIDED

F2.8 (IF MORE THAN ONE PERSON IN F2.7): Whose decision prevailed?

0 - Joint (specify) ____________ IN ADDITION TO THE CODES IN F2.7

F2.9 Who paid for your tuition and other school-related expenses when you were last in school?

SAME CODES AS IN F2.7 MULTIPLE RESPONSES ALLOWED

GO TO F2.11 IF ONLY ONE (1) PERSON PAID

F2.10 (IF MULTIPLE RESPONSE GIVEN IN F2.9): Who paid the most?

SAME CODES AS IN F2.8

F2.11 Do you want to go back to school?

0 - No CONTINUE
1 - Yes, when ____________ GO TO F2.13

F2.12 Why do you not want to go back to school?

VERBATIM: ____________

____

-9 - NA

GO TO F2.14

F2.13 Why do you want to go back to school?

VERBATIM: ____________

____

-9 - NA

F2.14 Whose decision would matter the most if you were to return (or not return) to school?

SAME CODES AS IN F2.7

GO TO INSTRUCTIONS BEFORE F2.17

@F2.15 What grade/year are you currently enrolled in?

1 -	Grade 1	10 -	Third Year High School
2 -	Grade 2	11 -	Fourth Year High School
3 -	Grade 3	12 -	First Year College
4 -	Grade 4	13 -	Second Year College
5 -	Grade 5	14 -	Third Year College
6 -	Grade 6	15 -	Fourth Year College
7 -	Grade 7	16 -	Fifth Year College
8 -	First Year High School	-8 -	NR/DK
9 -	Second Year High School	-9 -	NA

@F2.16 What school/college/university are you currently enrolled in (did you graduate from)?

RECORD NAME OF SCHOOL: ____

1 - Private, not coed, Catholic (e.g., STC, SHS, USC-HS)
2 - Private, not coed, not Catholic (e.g., Buddhist)
3 - Private, coed, Catholic
4 - Private, coed, not Catholic (lay) (e.g., UV)
5 - Public school
-8 - NR/DK
-9 - NA

IF IC IS IN COLLEGE, GRADUATED FROM COLLEGE OR WAS IN COLLEGE WHEN HE/SHE DROPPED OUT OF SCHOOL, CONTINUE

IF IC IS CURRENTLY IN HIGH SCHOOL, GRADUATED FROM HIGH SCHOOL, WAS IN HIGH SCHOOL, OR WAS IN ELEMENTARY WHEN HE/SHE DROPPED OUT OF SCHOOL, GO TO F2.30

F2.17 Was there a conscious decision by you or your family for you to proceed to college?

0 - No, it was assumed GO TO F2.21
1 - Yes
-8 - NR/DK
-9 - NA

F2.18 Who were the people contributing to this decision? MULTIPLE RESPONSES ALLOWED

1 - IC himself/herself
2 - Spouse/Partner of IC
3 - Father
4 - Mother
5 - Father-in-law
6 - Mother-in-law
7 - Other male relative
8 - Other female relative
9 - Non-household member, specify relationship ________________________
-8 - NR
-9 - NA

GO TO F2.20 IF ONLY ONE (1) PERSON DECIDED

F2.19 (IF MORE THAN ONE PERSON IN F2.18): Whose decision prevailed?

0 - Joint (specify ________________________)
1 - IC himself/herself
2 - Spouse/Partner of IC
3 - Father
4 - Mother
5 - Father-in-law
6 - Mother-in-law
7 - Other male relative
8 - Other female relative
9 - Non-household member, specify relationship ________________________
-8 - NR
-9 - NA

F2.20 What factors were considered behind the decision for you to go to college?

VERBATIM: ____

____

-9 - NA

F2.21. Who decided where (what school) you would study in college? MULTIPLE RESPONSES ALLOWED

0 - Joint (specify) ____________
1 - IC himself/herself
2 - Spouse/Partner of IC
3 - Father
4 - Mother
5 - Father-in-law
6 - Mother-in-law
7 - Other male relative
8 - Other female relative
9 - Non-household member, specify relationship ____________________
-8 - NR
-9 - NA

@F2.22 What course are (were) you taking in college?

____

-9 - NA

F2.23 What is/was your major in college?

____

-9 - NA

F2.24 How many years is/was the course you are/were taking in college?
ENTER NUMBER OF YEARS

-9 - NA

F2.25 Who decided what course you would take? MULTIPLE RESPONSES ALLOWED

SAME CODES AS IN F2.21

F2.26 Why did you/they choose this course?

VERBATIM: ____

____

F2.27 Who paid for your tuition and other school-related expenses when you were in college?

SAME CODES AS IN F2.21 MULTIPLE RESPONSES ALLOWED

GO TO F2.29 IF ONLY ONE (1) PERSON PAID

F2.28 (IF MULTIPLE RESPONSE GIVEN IN F2.27): Who paid the most?

SAME CODES AS IN F2.21

F2.29 Any academic honors/awards received?

0 - None
1 - Dean's list
2 - Graduated with honors, specify ______________________
3 - Board examination topnotcher, specify rank ______________________
4 - Other award, specify ______________________
-8 - NR/DK
-9 - NA

F2.29a Did you transfer school when you were in college?

0 - No
1 - Yes

F2.29b Did you change course(s) when you were in college?

0 - No
1 - Yes

F2.29c Did you shift major when you were in college?

0 - No
1 - Yes

@F2.30 Have you ever repeated a grade/year since June 2002 (or last visit)?

0 -	No	GO TO F2.33
1 -	Yes	CONTINUE
-8 -	NR/DK	GO TO F2.33
-9 -	NA	

@F2.31 What grade/year did you repeat?

____

-8 - NR/DK
-9 - NA

@F2.32 What was the reason for repeating the grade/year?

REASON: ____

____

-8 - NR/DK
-9 - NA

@F2.33 Did you ever skip a grade/year since June 2002 (or last visit)?

0 -	No	GO TO F2.35
1 -	Yes	CONTINUE
-8 -	NR/DK	GO TO F2.35
-9 -	NA	

@F2.34 What grade/year did you skip?

____

-8 - NR/DK
-9 - NA

@F2.35 Since June 2002 (or last visit), were there school years during which you did not enroll in school?

0 -	No	GO TO F2.38
1 -	Yes	CONTINUE
-8 -	NR/DK	GO TO F2.38

@F2.36 Which school year(s)?

ENTER FOUR DIGITS FOR YEAR(S)
-8 - NR/DK
-9 - NA

FIRST

SECOND

THIRD

F2.37 Why did you not enroll/attend school during that (those) year(s)?

VERBATIM: ____

____

-8 - NR/DK
-9 - NA

F2.38 I will mention a number of different reading materials. Please tell me whether or not you usually read them, what language they are in, the title or general theme of material, and whether you own any of these.

RECORD RESPONSES IN TABLE F2-1

Table F2-1

READING MATERIAL	IC READS		LANGUAGE Specify:	TITLE/ THEME Specify:	OWN MATERIAL	
	yes	no			yes	no
Books						
Magazines						
Comic Books						
Newspapers						

F2.39 What level of education do you wish to complete?
(FOR DROPOUTS OR THOSE WHO STOPPED SCHOOLING): What level of education would you have wanted to reach?

1 -	Some elementary	5 -	Some college
2 -	Elementary graduate	6 -	College graduate
3 -	Some high school	7 -	More than college
4 -	High school graduate	-8 -	NR/DK

F2.40 Do you think you can achieve this aspiration/Have you achieved this aspiration?

0 -	No	CONTINUE
1 -	Yes	GO TO INSTRUCTIONS BEFORE F2.42
2 -	Have achieved aspiration	GO TO INSTRUCTIONS BEFORE F2.42
-8 -	NR/DK	CONTINUE

F2.41 Why not?

REASON: ____

____

-8 - NR/DK
-9 - NR

IF INDEX CHILD IS NOT CURRENTLY IN SCHOOL (SY 2004-2005), GO TO F2.49;

IF INDEX CHILD IS CURRENTLY IN SCHOOL OR HAS GRADUATED FROM COLLEGE IN 2005, CONTINUE

F2.42 In the past month (or past 30 days), how many days have you missed school when school was in session?

(IF DURING SUMMER OR IF GRADUATED IN 2005, REFER TO LAST 30 SCHOOL DAYS)
ENTER NUMBER OF DAYS IN BOX
-8 - NR/DK
-9 - NA

IF 0, GO TO F2.44

F2.43 Why did you miss school?

VERBATIM: ____

____

____

-8 - NR/DK
-9 - NA

F2.44 I will mention a number of school-related expenses. Please tell me how much is paid for each and who pays for it:

ENTER FULL PESOS. RECORD RELATIONSHIP TO IC OF THE PERSON PAYING (TO BE CODED BY EDITORS).

Table F2-2. School Expenses

EXPENSE	PESOS	WHO PAYS
Transportation to and from school each day		
Food purchased in or around school each day		
Tuition/PTA/other school fees in past 12 months		
Uniforms (school, PE, scouting, CAT, etc.) in past 12 months		
Textbooks in the past 12 months		
Notebooks/paper/pens/other school supplies in past 12 months		
Board and lodging in the past 12 months		
Tutorial fees, extra-curricular activities in the past 12 months		
Other school expenses, in the past 12 months, specify ____________________		

-8 - NR/DK
-9 - NA

F2.45 Does/Did somebody usually help you with schoolwork?

0 - No GO TO F2.47
1 - Yes CONTINUE
-8 - NR/DK GO TO F2.47
-9 - NA

F2.46 Who usually helps/helped you with schoolwork? (MULTIPLE ANSWERS ALLOWED)

1 -	Mother	8 -	Tutor
2 -	Father	9 -	Friend
3 -	Sibling	10 -	Spouse/Partner of IC
4 -	Grandparent	11 -	Other non-relative (specify):
5 -	Aunt/uncle		____________________________
6 -	Cousin	-8 -	NR/DK
7 -	Other relative	-9 -	NA

F2.47 Do/Did you study with friends/peers/classmates?

0-	No	GO TO F2.49
1 -	Yes	CONTINUE
-8 -	NR/DK	GO TO F2.49
-9 -	NA	

F2.48 Where do/did you usually study?

1 - In own home
2 - At friend's home
3 - In school
4 - Elsewhere, specify: ____________________________

F2.49 I would now like to ask you about other types of schooling such as vocational, technical, or on-the-job training that you may have had in addition to or in combination with your formal schooling.

a) Have you attended any vocational, technical or on-the-job training program since 2002/last visit?

0 -	No	GO TO F3.1
1 -	Yes	CONTINUE
-8 -	NR/DK	

b) How many such training have you attended?
ENTER NUMBER OF TRAINING
-9 - NA

FOR EACH VOCATIONAL/TECHNICAL/ON-THE-JOB TRAINING PROGRAM ATTENDED, ASK F2.49c to F2.49l AND RECORD RESPONSES IN TABLE F2-3; INCLUDE ONGOING TRAINING PROGRAM THAT IC MAY BE ATTENDING.

c) What kind of training? ENTER TITLE/DESCRIPTION IN COLUMN F2.49c
-9 - NA

d) Is/Was this a vocational, technical or on-the-job-training program?

1 - Vocational training
2 - Technical training
3 - On–the-job-training
-9 - NA

e) Where did you receive this training?
ENTER NAME OF TRAINING CENTER IN COLUMNF2.49e
-9 - NA

f) When did you start this training? ENTER MONTH AND YEAR IN COL. F2.49f
-9 - NA

g) How long did the training last?
ENTER RESPONSE AS GIVEN. EDITOR WILL CODE AS NUMBER OF WEEKS
-99 - Training still ongoing
-9 - NA

h) Did you complete this training?
0 - No GO TO F2.49k
1 - Yes CONTINUE
-99 - Training still ongoing
-9 - NA

i) Did you get a certificate, license or diploma for this training?

0 - No GO TO F2.49k
1 - Yes CONTINUE
-9 - NA

j) What kind of certificate/license/diploma did you get?
ENTER TITLE/DESCRIPTION OF CERTICATE IN COLUMN F2.49j
-9 - NA

k) What made you decide to undergo this training?
ENTER VERBATIM RESPONSE IN COLUMN F2.49k
-9 - NA

l) Who paid for this training?
ENTER VERBATIM RESPONSE IN COLUMN F2.49l
-9 - NA

Table F2-3. Training Programs Attended

KIND OF TRAINING (F2.49c)	TYPE (F2.49d)	PLACE OF TRAINING (F2.49e)	DATE OF TRAINING (MONTH & YEAR) (F2.49f)	DURATION OF TRAINING (F2.49g)	COMPLE-TED? (F2.49h)	CERTI-FICATE (F2.49i)	TYPE OF CERTIFICATE (F2.49j)	REASON FOR TRAINING (F2.49k)	WHO PAID TRAINING (F2.49l)

III: EMPLOYMENT OF INDEX CHILD

AS IN "SCHOOLING OF INDEX CHILD", SOME INFORMATION ON EMPLOYMENT CAN ALSO BE FOUND IN THE LHM. INFORMATION WILL BE COPIED INTO THIS SECTION IN THE QUESTIONS MARKED BY AN @ SIGN.

SKIP F3.1 TO F3.7 IF INFORMATION IS ALREADY OBTAINED IN 2002 LHM, OTHERWISE, CONTINUE

@F3.1 Have you ever worked? I do not mean household chores but work for which you are paid in cash or in kind, or if your family operates a farm or owns a family business, have you ever been asked to work in them?***

0 - No GO TO F3.4
1 - Yes CONTINUE

☐

@F3.2 At what age did you start working?

ENTER AGE IN YEARS
-8 - NR/DK
-9 - NA

☐☐

F3.3 Whose idea was it that you started working?

0 - Own idea
1 - Spouse/partner
2 - Father
3 - Mother
4 - Both parents
5 - Other relatives, specify ____________________________
6 - Other persons, specify _____________________________
-8 - NR/DK
-9 - NA

☐

F3.4 Do you like the idea of working?

0 - No
1 - Yes
-8 - NR/DK
-9 - NA

☐

F3.5 Why?

VERBATIM: ____

____

☐

-8 - NR/DK
-9 - NA

F3.6 What do you think are the advantages of your working?
IF IC HAS NOT STARTED WORKING, ASK QUESTION HYPOTHETICALLY

VERBATIM:
____

____

☐

-8 - NR/DK
-9 - NA

F3.7 What do you think are the disadvantages of your working?
VERBATIM:

____

____

☐

-8 - NR/DK
-9 - NA

IF IC HAS NEVER WORKED (CODED "0" IN F3.1), GO TO F3.30

F3.7a Are you currently working?

0 - No
1 - Yes
-9 - NA

@F3.8 What is/was/were your present and past occupation?

IF IC WAS INTERVIEWED IN 2002 AND EVER HAD A JOB, ASK FOR ALL JOBS HE/SHE HAS HAD AFTER 2002. BEGIN FROM THE FIRST JOB AFTER 2002 SURVEY TO THE LATEST/CURRENT JOB. RECORD MAIN JOBS AND SECONDARY JOBS ACCORDING TO THE HISTORICAL SEQUENCE THAT THEY ARE REPORTED. THAT IS, FIRST JOBS ARE RECORDED TO THE LEFT OF THE MORE RECENT JOBS. THE CURRENT JOB SHOULD APPEAR IN THE RIGHT-MOST COLUMN.

HOWEVER, IF IC WAS NOT INTERVIEWED IN 2002, FROM THE LHM, COPY INTO THE FIRST ROW OF TABLE F3-1 ALL OCCUPATIONS THAT IC HAS HAD. BEGIN FROM THE EARLIEST TO THE LATEST/ CURRENT JOB. RECORD MAIN JOBS AND SECONDARY JOBS ACCORDING TO THE HISTORICAL SEQUENCE THAT THEY ARE REPORTED IN THE LHM, THAT IS, EARLIER JOBS ARE RECORDED TO THE LEFT OF THE MORE RECENT JOBS. THE CURRENT JOB SHOULD APPEAR IN THE RIGHT-MOST COLUMN.

FOR EACH JOB, ASK F3.9 TO F3.29 AND RECORD RESPONSES IN THE SPACES PROVIDED IN TABLE F3-1.

F3.9 Who is/was your employer in this job?

(Are/Were you employed by government, a private company, private individual/family, non-profit organization, your own family, or self-employed?)

1 - Self employed (operated by IC himself/herself
2 - Own family (operated by another member of IC's family)
3 - Private individual/family
4 - Private company
5 - Non-profit organization
6 - Government
7 - Others (specify)
-9 - NA

F3.10 **What type of business or industry are you is/was your employer engaged in?**

1 - Agriculture, fishery and forestry
2 - Mining, quarrying
3 - Manufacturing
4 - Electricity, gas and water
5 - Construction
6 - Wholesale trade
7 - Retail trade
8 - Transportation and communication
9 - Financing, insurance, real estate and business services
10 - Community and social services
11 - Personal services
12 - Others, not adequately defined
-9 - NA

F3.11 **What are/were your usual activities at this job?** (e.g., typing, accounting, selling, etc.) ENTER DESCRIPTION

-9 - NA

F3.12 **In what physical position do/did you usually carry out this job?**

1 - Sitting most of the time
2 - Standing most of the time
3 - Squatting on the ground most of the time (e.g. doing laundry , weeding the farm)
4 - Standing and bending over most of the time (e.g. planting rice)
5 - Moving around most of the time
6 - Combination of any codes above (specify codes)
-9 - NA

F3.13 **Does/Did your job require physical exertion (e.g., lifting, pushing objects, etc.)? IF YES, what kind?**

0 - No
1 - Yes (specify)
-9 - NA

F3.14 **Does your job require the use of equipment? IF YES, what kind? (e.g., calculator, computer, etc.)**

0 - No
1 - Yes (specify)
-9 - NA

F3.15 **What is/was the minimum educational level required (by your employer) for this job?**

0 - None
1 - Some elementary education
2 - Elementary graduate
3 - Some high school education
4 - High school graduate
5 - Some college education
6 - College graduate
7 - More than college
8 - Vocational/technical training
9 - Others (specify)
-9 - NA

F3.16 **Does/Did this job require prior work experience, apprenticeship or on-the-job training?**

0 - No
1 - Yes
-8 - NR/DK
-9 - NA

F3.17 **Do/Did you supervise people in this job? IF YES, how many?**

0 - No
1 - Yes, 1 or 2 people
2 - Yes, 3 to 5 people
3 - Yes, 6 to 10 people
4 - Yes, more than 10 people
-9 - NA

F3.18 **How many days in a week do/did you usually work?**

WRITE NUMBER OF DAYS

-8 - NR/DK
-9 - NA

F3.19 **How many hours do/did you usually work during a week?**
WRITE NUMBER OF HOURS

-8 - NR/DK -9 - NA

F3.20 **Do/Did you usually work the same number of hours each week?**

0 - No -8 - NR/DK
1 - Yes -9 - NA

F3.21 **How are/were you paid in this job?**

1 - In kind
2 - In cash, time basis
3 - In cash, piece basis
4 - In cash, commission/profit basis
5 - In cash and in kind
6 - Unpaid family worker GO TO F3.26
-9 - NA

F3.22 **Are/Were you paid on an hourly, daily, weekly or monthly basis?**

1 - Per hour 4 - Per month
2 - Per day 5 - Other (specify)
3 - Per week -9 - NA

F3.23 **How much do/did you usually earn per day?**
CODE AMOUNT IN PESOS
IF PAID PER WEEK OR MONTH, CALCULATE DAILY AVERAGE

-8 - NR/DK -9 - NA

@F3.24 **Do/Did you receive any employment benefits at this job? (e.g., SSS, PhilHealth, PAG-IBIG)**

0 - No 1 - Yes

F3.25 **Do/Did you receive overtime pay for this job? If so, how much per month on average?**

0 - No -8 - NR/DK
1 - Yes (AMOUNT) -9 - NA

F3.26 **Who decided that you take this job?**
MULTIPLE RESPONSES ALLOWED

0 - Own idea
1 - Spouse/Partner
2 - Father
3 - Mother
4 - Both parents
5 - Other relatives, specify
6 - Other persons, specify
-8 - NR/DK
-9 - NA

F3.27 **Why did you/he/she/they choose this job?**
VERBATIM

9 - NA

F3.28 **How did you get this job?**
VERBATIM

-9 - NA

F3.29 **How do/did you feel about this job?**

1 - Like it very much
2 - Like it fairly well
3 - Indifferent
4 - Dislike it somewhat
5 - Dislike it very much
-9 - NA

Table F3-1. Employment History of IC

	First job		Second job		Third job		Fourth job	
	Main job	Secondary job	Main job	Secondary job	Main job	Secondary job	Main job	Secondary job
F3.8 Occupation								
F3.9 Employer								
F3.10 Business/ Industry								
F3.11 Usual activity								
F3.12 Physical position								
F3.13 Physical exertion? What?								
F3.14 Equipment? What?								
F3.15 Educ. level								
F3.16 Prior experience								
F3.17 Supervise people								
F3.18 Usual days-work/wk.								
F3.19 Usual hrs./wk.								
F3.20 Same hrs./wk.								
F3.21 How paid								
F3.22 Paid by hr./ day/week								
F3.23 Earning/day								
F3.24 With benefits								
F3.25 With overtime pay								
F3.26 Who decided for job								
F3.27 Why this job chosen								
F3.28 How you got the job								
F3.29 How you feel about the job								

AFTER ASKING ABOUT LAST/CURRENT JOB:

F3.30 What kind of job would you like to have in the future, say, 10 years from now?
VERBATIM:

____

____

-8 - NR/DK

F3.31 Do you think it is possible for you to achieve this aspiration?

0 -	No	CONTINUE
1 -	Yes	GO TO F3.33
-8 -	NR/DK	GO TO F3.34
-9 -	NA	

F3.32 Why is it not possible for you to achieve this aspiration?
VERBATIM:

____

____

-8 - NR/DK
-9 - NA

GO TO F3.34

F3.33 What would you need to do to achieve this aspiration?
PROBE FOR SOMETHING CONCRETE

VERBATIM:

____

____

-8 - NR/DK
-9 - NA

F3.34 Aside from your regular job (or: If you do not have a regular job), do you earn some money by other means?

EXAMPLES SUCH AS RUNNING ERRANDS, SELLING THINGS, OR OTHERS THAT ARE NOT CONSIDERED AS REGULAR JOBS

If yes, by what means? (VERBATIM) ____

____

0 - No GO TO F3.36
-9 - NA

IF YES IN F3.34, CONTINUE; OTHERWISE, GO TO F3.36

F3.35 In the past 12 months, how much did you earn by this means?

AMOUNT IN PESOS: _______________
-9 - NA

IF IC IS CURRENTLY WORKING, ASK:

F3.36 Who decides how your earnings are spent?

IF IC IS NOT CURRENTLY WORKING, ASK:
Who do you think will decide how your earnings will be spent?

0 - IC alone
1 - Spouse/Partner
2 - Father
3 - Mother
4 - Both parents
5 - Other relatives, specify ___________________________
6 - Other persons, specify ___________________________
-8 - NR/DK
-9 - NA

F3.37 What do/will you do with your earnings?

1 -	Keep all	CONTINUE
2 -	Keep part	CONTINUE
3 -	Turn all over to spouse/partner	GO TO F4.1
4 -	Turn all over to mother/father	GO TO F4.1
5 -	Turn all over to other relatives. Specify relative ______________	GO TO F4.1
-8 -	NR/DK	
-9 -	NA	

F3.38 If you keep all or part of your earnings, what do/will you usually spend/be spending this money on?

VERBATIM:

____

____

-8 - NR/DK
-9 - NA

IF IC KEEPS/WILL KEEP ALL OF HIS/HER EARNINGS, GO TO F4.1

IF IC KEEPS/WILL KEEP PART OF HIS/HER EARNINGS, CONTINUE

F3.39 How much in proportion to your total earnings do/will you keep?

______ %

F3.40 Who do/will you share your earnings with and how large a proportion do/will you give to each of them?

NAME:

NAME	%
____________________	____ %
____________________	____ %
____________________	____ %
____________________	____ %

F3.41 Is/Will this manner of sharing done/be done regularly or not?

0 - Not regular
1 - Regular
-8 - NR/DK
-9 - NA

IV. REPRODUCTION

PHYSICAL CHARACTERISTICS:

F4.1 How do you describe your body in terms of your weight?

1 - Slender
2 - Average
3 - Chubby
-8 - NR/DK

F4.2 With your present weight, do you want to:

1 - Be thinner
2 - Be heavier
3 - Maintain present weight
4 - Don't care
-8 - NR/DK

F4.3 What did you do/are you doing to achieve the weight you wish to have?

VERBATIM: ____

____

GO TO F4.5 IF IC IS MALE

F4.4 BODY IMAGE (FOR FEMALE IC)

INTERVIEWER: SHUFFLE THE NINE (9) DRAWINGS OF A WOMAN'S BODY FIGURE SO THAT THEY ARE IN RANDOM ORDER. SHOW THEM TO IC AND ASK HER TO IDENTIFY WHICH DRAWING SHE THINKS SHE RESEMBLES THE MOST. WRITE IN THE BOX THE NUMBER OF THE DRAWING CHOSEN.

a) I would like to show you some drawings. These drawings show the different body figures of a woman. In your opinion, which drawing closely resembles your body figure?

SHUFFLE THE DRAWINGS AGAIN AND SHOW THEM TO IC. ASK HER TO IDENTIFY WHICH DRAWING SHE WOULD WANT TO LOOK LIKE. WRITE IN THE BOX THE NUMBER OF THE DRAWING SHE CHOOSES.

b) I am going to show you the drawings again. Please tell me which drawing you would want to look like.

c) What are you doing to achieve your desired body figure?

VERBATIM____

____

d) INTERVIEWER ONLY! How do you assess IC's body figure based on the nine drawings?

INTERVIEWER: SHUFFLE THE NINE (9) DRAWINGS OF A MAN'S BODY FIGURE SO THAT THEY ARE IN RANDOM ORDER. SHOW THEM TO IC AND ASK HER TO IDENTIFY WHICH DRAWING SHE WOULD WANT A MAN TO LOOK LIKE. WRITE IN THE BOX THE NUMBER OF THE DRAWING CHOSEN.

e) I am going to show you another set of drawings. These drawings show the different body figures of a man. Please tell me which drawing you would want a man to look like.

GO TO F4.6

F4.5 BODY IMAGE (FOR MALE IC)

INTERVIEWER: SHUFFLE THE NINE (9) DRAWINGS OF A MAN'S BODY FIGURE SO THAT THEY ARE IN RANDOM ORDER. SHOW THEM TO IC AND ASK HIM TO IDENTIFY WHICH DRAWING HE THINKS HE RESEMBLES THE MOST. WRITE IN THE BOX THE NUMBER OF THE DRAWING CHOSEN.

a) I would like to show you some drawings. These drawings show the different body figures of a man. In your opinion, which drawing closely resembles your body figure? ☐

SHUFFLE THE DRAWINGS AGAIN AND SHOW THEM TO IC. ASK HIM TO IDENTIFY WHICH DRAWING HE WOULD WANT TO LOOK LIKE. WRITE IN THE BOX THE NUMBER OF THE DRAWING HE CHOOSES.

b) I am going to show you the drawings again. Please tell me which drawing you would want to look like. ☐

c) What are you doing to achieve your desired body figure? ☐

VERBATIM____

____

d) INTERVIEWER ONLY! How do you assess IC's body figure based on the nine drawings? ☐

INTERVIEWER: SHUFFLE THE NINE (9) DRAWINGS OF A WOMAN'S BODY FIGURE SO THAT THEY ARE IN RANDOM ORDER. SHOW THEM TO IC AND ASK HIM TO IDENTIFY WHICH DRAWING HE WOULD WANT A WOMAN TO LOOK LIKE. WRITE IN THE BOX THE NUMBER OF THE DRAWING CHOSEN.

e) I am going to show you another set of drawings. These drawings show the different body figures of a woman. Please tell me which drawing you would want a woman to look like. ☐

FAMILY PLANNING

IF THE LHM SHOWS THAT IC HAS USED FAMILY PLANNING, CODE "1" (YES) IN F4.6 BELOW, IF NOT, ASK F4.6

@F4.6 Have you heard of family planning or using certain means to prevent pregnancy? ☐

0 -	No	GO TO F4.14
1 -	Yes	CONTINUE
-8-	NR	GO TO F4.14

F4.7 From where did you get your information on family planning? ☐

VERBATIM:

____

____

-8 - NR/DK
-9 - NA

F4.8 Nowadays, there are many family planning methods. What method have you heard of?

LET IC ENUMERATE ALL METHODS HE/SHE KNOWS WITHOUT PROMPTING HIM/HER. WRITE “1" IN THE SPONTANEOUS COLUMN FOR EACH METHOD MENTIONED SPONTANEOUSLY; THEN PROCEED DOWN THE COLUMN, READ THE NAME OF EACH METHOD NOT MENTIONED SPONTANEOUSLY, AND WRITE “2" UNDER PROMPTED ANSWER COLUMN IF IC HAS HEARD OF METHOD, AND “0" IF HE/SHE HAS NOT.

METHOD		HEARD OF	
		SPONTANEOUS	PROMPTED ANSWER
1	Pill		
2	IUD		
3	Injection (DEPO PROVERA)		
4	Implant (NORPLANT)		
5	Diaphragm		
6	Foam, Jelly (SAMPOON)		
7	Condom		
8	Ligation		
9	Vasectomy		
10	Rhythm, Calendar Method		
11	Rhythm, Temperature, Symptoms		
12	Withdrawal		
13	Breastfeeding		
14	Abstinence		
15	Other (specify)		
-9	NA		

F4.9 Do you know where to get family planning supplies/services?

0 - No GO TO F4.11
1 - Yes CONTINUE
-8 - NR/DK GO TO F4.11
-9 - NA

F4.10 Where can you get family planning supplies/services?
VERBATIM:

____

____

-8 - NR/DK
-9 - NA

F4.11 In your opinion, do you need to know about family planning at your age?

0 - No
1 - Yes
-8 - NR/DK
-9 - NA

F4.12 In your opinion, what is the right age for a person to know about family planning?

IF EXACT AGE IS GIVEN: _________

IF ANSWER IS CONDITIONAL: VERBATIM: ______________________________

____

-9 - NA

F4.13 From whom should a person first learn about family planning?

VERBATIM: ____

-9 - NA

SEXUAL RELATIONS

INTERVIEWER: IF NO PRECODED RESPONSE, ASK:

F4.14 Have you ever had a crush on a girl/boy (opposite sex of IC)?***

0 - No
1 - Yes
-8 - NR/DK

F4.15 Have you ever had a crush on a boy/girl (same sex as IC)?***

0 - No
1 - Yes
-8 - NR/DK

IF ANSWER IN F4.14 OR F4.15 IS "1" (YES), ASK:

@F4.16 How old were you when you had your first crush?***

AGE: ____________

-9 - Did not have a crush

F4.17 Have you ever gone on a date?***

0 - No
1 - Yes
3 - Unsure

IF ANSWER IN F4.17 IS "1" (YES), ASK:

@F4.18 At what age did you have your first date?***

AGE: ___________

-9 - NA (Never gone on a date) GO TO F4.21

F4.19 Was your first date:

1- As a group
2- You and your date with a chaperone
3 - You and your date without a chaperone
-8 - NR
-9 - NA (Never gone on a date)

F4.20 What did you do on your first date?

VERBATIM: ____

____

-8 - NR
-9 - NA (Never gone on a date)

F4.21 Have you ever courted a girl/boy (opposite sex as IC)?***

0 - No
1 - Yes
-8 - NR/DK

F4.22 Have you ever courted a boy/girl (same sex as IC)?***

0 - No
1 - Yes
-8 - NR/DK

IF ANSWER IN F4.21 OR F4.22 IS "1" (YES), ASK:

@F4.23 At what age did your first court someone?

AGE: __________

-9 - NA (Did not court someone)

F4.24 Have you ever been courted by a girl/boy (opposite sex as IC)?***

0 - No
1 - Yes
-8 - NR/DK

F4.25 Have you ever been courted by a boy/girl (same sex as IC)?***

0 - No
1 - Yes
-8 - NR/DK

IF ANSWER IN F4.24 OR F4.25 IS "1" (YES), ASK:

@F4.26 At what age were you first courted by someone?

AGE: __________

-9 - NA (Not courted by anyone)

IF IC IS CURRENTLY MARRIED OR COHABITING, DO NOT ASK QUESTIONS F4.27, F4.30 AND F4.31; JUST RECORD A "YES" RESPONSE TO THESE QUESTIONS

@F4.27 Have you ever been in a romantic relationship (opposite sex as IC)?***

0 - No GO TO F4.29
1 - Yes CONTINUE
3 - Unsure GO TO F4.29

@F4.28 At what age did you have your first romantic relationship?

AGE: ___________

-9 - NA

F4.29 Have you had a romantic relationship with someone of the same sex?***

0 - No
1 - Yes
-8 - NR/DK

F4.29a Are you in a romantic relationship at present?

0 - No
1 - Yes
-8 - NR/DK

F4.29b What do you understand by romantic relationship?

VERBATIM: ____

____

F4.30 Please allow me to ask you questions about sexual experiences. Regardless of whether you have had a romantic relationship or not; with the opposite sex or with the same sex. Tell me if you have ever done the following:

a)	Holding hands	0 -	No	1 -	Yes	-8 -	NR
b)	Kissing	0 -	No	1 -	Yes	-8 -	NR
c)	Petting	0 -	No	1 -	Yes	-8 -	NR

@F4.31 Have you ever had sexual intercourse?

0 - No GO TO F4.39a
1 - Yes CONTINUE
-8 - NR GO TO F4.39a

@F4.32 At what age did you first have sexual intercourse?

AGE: ___________

-9 - NA

F4.33 Where did the first sexual intercourse take place?

WRITE PLACE MENTIONED BY IC:____

-9 - NA

F4.33a Was the first intercourse. . .

1 - Something you wanted to happen
2 - Something you did not want to happen but you went along with
3 - Something you did not plan to happen but happened anyway
4 - Something that happened against your will
-9 - NA

F4.33b People engage in sexual activities for a variety of reasons. What were your reasons for having sex the first time?

VERBATIM: ____
-8 - NR
-9 - NA

F4.33c How many people have you had sex with since you began having sex?

-8 - NR
-9 - NA

F4.33d Have you ever paid anyone to have sex with him/her?

0 - No
1 - Yes
-9 - NA

F4.33e Have you ever been paid to have sex with someone else?

0 - No
1 - Yes
-9 - NA

F4.33f Have you had any sexual contact with someone of the same sex?

0 - No
1 - Yes
-9 - NA

F4.33g How often in the past month have you had sex?

0 - Never GO TO F4.33i
1 - Once or twice
2 - Once a week
3 - 2 or more times a week
-9 - NA

F4.33h Did you or your partner use any contraceptive method in the past month?

0 - No ASK: Why not? ____
1 - Yes
-9 - NA

F4.33i Have you ever had sex against your will?

0 - No
1 - Yes
-9 - NA

F4.33j Have you ever forced somebody to have sex?

0 - No
1 - Yes
-9 - NA

F4.33k Have you ever experienced group sex?

0 - No
1 - Yes
-9 - NA

F4.33l Have you ever experienced sex with any member of your family?

0 - No
1 - Yes
-9 - NA

@F4.34 Have you ever used family planning?

0 - No CONTINUE
1 - Yes GO TO F4.36
-8 - NR
-9 - NA

F4.35 Why have you not used family planning?

VERBATIM: ____

____

-9 - NA

GO TO F4.39a

@F4.36 What family planning methods have you used?

NAME ALL METHODS EVER USED: ______________________________

____

-9 - NA

F4.37 Where do/did you get your supply or services for family planning?

SOURCES: ____

____

-9 - NA

F4.38 What method are you currently using?

0 - None GO TO F4.39

METHOD(S): ____

____

-9 - NA

GO TO F4.39a

F4.39 Why did you stop using family planning?

0 - Currently pregnant

VERBATIM: ____

____

-9 - NA

F4.39a In your opinion, should a government health center provide family planning as one of its services?

0 - No
1 - Yes

F4.39b In your opinion, should a school provide family planning in high school as one of its services?

0 - No
1 - Yes

F4.39c Is your religion for or against family planning?

1 - In favor
2 - Against
3 - Neutral
4 - Don't know

F4.39d Had your mother/father ever talked to you about family planning?

0 - No
1 - Yes ASK: What about family planning did you discuss?
VERBATIM:____

____

F4.39e Do you think you will use family planning in the future?

0 - No
1 - Yes

F4.39f What do you think will influence your future use of family planning?

1 - Self
2 - Family/relative
3 - Other people
4 - Religion GO TO F4.39h
5 - Media (specify type of media ______________________________)
6 - Other, specify ____

F4.39g Do you think your future use of family planning will be affected by your religion's stand on family planning?

0 - No
1 - Yes

F4.39h Do you think your future use of family planning will be affected by the government's stand on family planning?

0 - No
1 - Yes

IF IC IS NOT SEXUALLY ACTIVE, GO TO BLOCK G, OTHERWISE CONTINUE

PREGNANCY

@ F4.40 IF IC IS FEMALE: Have you ever been pregnant?***
IF IC IS MALE: Have you ever gotten a woman pregnant?***

0 - No GO TO F4.45 IF ANSWER IN F4.31 IS YES, OTHERWISE , GO TO F4.47
1 - Yes CONTINUE
2 - Woman unsure about current first pregnancy (female IC only) GO TO F4.42
3 - Man unsure about pregnancy (male IC only) GO TO F4.42
-8 - NR/DK

GO TO F4.41 IF IC IS MALE

F4.40a Are you currently pregnant?

0 - No
1 - Yes; no. of months: ____________________
-9 - NA

@ F4.41 IF FEMALE: How many pregnancies have you had?
IF MALE: How many times have you gotten a woman pregnant?

NO. OF PREGNANCIES: ____________
-9 - NA

F4.42 Was this pregnancy/Were all of these pregnancies intended or planned?

0 - No CONTINUE
1 - Yes GO TO F4.44 IF IC IS FEMALE, OTHERWISE, GO TO F4.45
-9 - NA

F4.43 How did you react to the unintended/unplanned pregnancy?
PROBE FOR INTENTION TO ABORT

VERBATIM: ____

____

-9 - NA

IF IC IS MALE, GO TO F4.45. IF IC IS FEMALE AND HAS HAD A PREGNANCY (INCLUDING CURRENT PREGNANCY), CONTINUE

F4.44 PREGNANCY HISTORY

THE PREGNANCY HISTORY IS A COMPLETE RECORD OF ALL THE PREGNANCIES IC HAS HAD, INCLUDING HER CURRENT PREGNANCY. THIS INCLUDES:

A. ALL LIVE BIRTHS, REGARDLESS OF WHETHER THEY HAVE DIED SINCE OR ARE LIVING IN THE SAME HOUSEHOLD AS IC OR ELSEWHERE,
B. ALL STILLBIRTHS
C. ALL OTHER PREGNANCY TERMINATIONS NOT ENDING IN LIVE BIRTH OR STILLBIRTH (e.g. MISCARRIAGE)

RECORD ALL PERTINENT INFORMATION /ANSWERS IN TABLE F4-1.

(1) Now I would like to ask you about each of your pregnancies beginning from the first to the last.

LIST ALL PREGNANCIES IC EVER HAD OR HAD SINCE 2002 IN TABLE F4-1. FOR CHILDREN OF IC WITH NAMES, LIST NAMES IN ROW (2).

FOR EACH PREGNANCY, ASK:

@(2) What was the result of this pregnancy?

1 - Single live birth still living
2 - Single live birth now dead
3 - Stillbirth
4 - Miscarriage
5 - Multiple (indicate twins, triplets, etc., and if all alive or some died)
6 - Currently pregnant
-7 - Not sure if currently pregnant

(3) What is the date of birth or pregnancy termination? (Month, Day, Year)

-7 - Not sure if currently pregnant
-9 - Currently pregnant

(4) How many months or weeks were you pregnant?

IF CURRENTLY PREGNANT, GIVE NUMBER OF MONTHS OR WEEKS PREGNANT.

IF NOT SURE IF PREGNANT, GIVE NUMBER OF WEEKS OR DAYS SINCE LAST MENTRUATION (START FROM THE FIRST DAY OF LAST CYCLE) THEN GO TO F4.45.

(5) Did you get prenatal care during this pregnancy?

0 - No GO TO INSTRUCTION BEFORE (12)
1 - Yes CONTINUE

(6) When (in what month of your pregnancy) did you first get prenatal care? (Month, Day, Year or in what month of pregnancy)

-9 - NA

(7) Where did you first get prenatal care for this pregnancy?

1 - Traditional birth attendant
2 - Government personnel/facility
3 - Private personnel/facility
-9 - NA

(8) How many times during this pregnancy did you get prenatal care from _______(CATEGORIES IN (7)?

0 - None (for this category)
1 - Once
:
-9 - NA (Did not get any prenatal care)

(9) Were you given any supplements/vitamins during this pregnancy? If yes, what kind?

MULTIPLE ANSWERS ALLOWED

0 - No
1 - Yes, (specify)

(10) Were you given an injection during this pregnancy? If yes, what kind?

MULTIPLE ANSWERS ALLOWED

0 - No injection GO TO INSTRUCTION BEFORE (12)
1 - 1st injection (specify)
2 - 2nd injection (specify)
-9 - NA

(11) Who gave you this injection?

1st injection __________
2nd injection __________
-9 - NA

ASK QUESTIONS (12) TO (17) OF LIVE BIRTHS AND STILLBIRTHS ONLY. IF PREGNANCY RESULTED IN MISCARRIAGE, GO TO (35). IF CURRENTLY PREGNANT, GO TO (38)

@(12) Is the child male or female?

1 - Male
2 - Female
-9 - NA

(13) Where was the child born?

1 - At home
2 - Public hospital/health center (specify)
3 - Private hospital/private clinic (specify)
4 - Others (specify)
-9 - NA

(14) Who assisted you in delivering the child?

1 - Doctor
2 - Midwife
3 - Traditional birth attendant
4 - Others (specify)
5 - Combination (specify)
-9 - NA

(15) What type of delivery?

1 - Normal
2 - "Caesarian"
-9 - NA

(16) Were there any complications during labor or delivery?

0 - None
1 - Yes (describe)
-9 - NA

(17) Were you given anesthesia during delivery?

0 - No
1 - Yes
-9 - NA

IF STILLBIRTH, GO TO (38)

(18) Was child weighed at birth?

0 - No GO TO (23)
1 - Yes
-9 - NA

(19) Where was child weighed?

1 - At home
2 - Public hospital/health center (specify)
3 - Private hospital/private clinic (specify)
4 - Others (specify)
-9 - NA

(20) Who weighed the child?

1 - Doctor
2 - Nurse
3 - Midwife
4 - Traditional birth attendant
5 - Others (specify)
-9 - NA

(21) What was the child's weight at birth?

RECORD WEIGHT IN WHATEVER UNIT THE IC REPORTS IN APPROPRIATE ROW, I.E., FROM MOM'S RECALL OR FROM RECORD).

-9 - NA

(22) When was the child's weight taken?

RECORD MONTH, DAY AND YEAR

-9 - NA

(23) IF CHILD WAS NOT WEIGHED AT BIRTH: What do you think was the child's weight at birth?
-9 - NA

(24) Do you think the child was born early, late or on time?

1 - Early
2 - On time
3 - Late
-8 - NR/DK
-9 - NA

IF CHILD DIED, CONTINUE; IF STILL ALIVE GO TO (27)

(25) When did child die?

RECORD MONTH, DAY AND YEAR

-9 - NA

@(26) What was the cause of death?

ASK OF ALL LIVE BORN CHILDREN ONLY

(27) Did you ever breastfeed the child? If not, why?

0 - No (give reason) GO TO (33)
1 - Yes CONTINUE
-9 - NA

Table F4-1. Pregnancy History of IC

QUEST. NO.	PREG. ORDER	FIRST PREGNANCY	SECOND PREGNANCY	THIRD PREGNANCY	FOURTH PREGNANCY
	NAME				
2) Type of pregnancy termination					
3) Date of pregnancy termination					
4) Pregnancy duration					
5) With prenatal care?					
6) Date of first prenatal care					
7) Source of first prenatal care					
8) Frequency of prenatal care					
9) Supplements or vitamins taken and type					
10) Injections received and type					
11) Source of injection					
12) Sex of child					
13) Place of delivery					
14) Type of personnel who assisted delivery					
15) Type of delivery					
16) Any complications during labor or delivery?					
17) Received anesthesia?					
18) Was child weighed at birth?					
19) Place where child was weighed					
20) Type of personnel who weighed the child					
21) Weight of child at birth (Mom's recall)					
(From record)					
22) Date weight of child was taken					
23) IC's perception of child's weight					
24) Child born early, on time or late					
25) Date child died					
26) Cause of child's death					
27) Was child ever breastfed?					

(28) When did you first breastfeed the child?

RECORD IN NUMBER OF HOURS AFTER BIRTH
-9 - NA

(29) Are you still breastfeeding the child? If not, why?

0 - No (give reason) CONTINUE
1 - Yes GO TO (31)
-9 - NA

(30) How long was the child breastfed?

RECORD RESPONSE OF IC
-9 - NA

(31) Is/Was child given any breast milk supplement (including infant formula)? If yes, what kind(s)?

0 - No GO TO (33)
1 - Yes (GIVE TYPE/NAME OF SUPPLEMENT(S): __________
-9 - NA

(32) How old was child when first given breast milk supplement on a regular basis?

RECORD RESPONSE OF IC
-9 - NA

(33) Is/Was child given semi-solid and solid foods?

0 - No GO TO (38)
1 - Yes
-9 - NA

(34) How old was child when first given semi-solid/solid foods on a regular basis?

RECORD RESPONSE OF IC
-9 - NA

GO TO (38)

ASK (35) TO (37) ONLY FOR MISCARRIAGE.

(35) Did you or anyone do something to end this pregnancy?

0 - No GO TO (38)
1 - Yes
-9 - NA

(36) Who did something to end this pregnancy?

1 - IC herself
2 - Doctor
3 - Nurse
4 - Midwife
5 - Traditional birth attendant/ mananabang
6 - Others (specify)
-9 - NA

(37) What did you/he/she do?

VERBATIM:

(38) Who is the father of this child/pregnancy?

WRITE NUMBER OF FATHER (FATHER #1, FATHER #2, etc.)
-9 - NA

(39) Were you cohabiting with or married to the father at the time of this pregnancy?

0 - No
1 - Yes, cohabiting
2 - Yes, legally married
-9 - NA

(40) Before this pregnancy (or between this pregnancy and the previous one), what FP method were you using for the longest duration?

0 - None
-9 - NA

@(41) Did you work during this pregnancy? If yes, what kind of work?

0 - No
1 - Yes (specify kind of work/occupation)
-9 - NA

@(42) Did you work after this pregnancy? If yes, what kind of work?

0 - No
1 - Yes (specify kind of work/occupation)
-7 - Still pregnant
-9 - NA

(43) Who performed household chores:

a) During this pregnancy

1 - IC herself
2 - Mother/Mother-in-law of IC
3 - Paid help
4 - Other (specify)
-9 - NA

b) One month after delivery

1 - IC herself
2 - Mother/Mother-in-law of IC
3 - Paid help
4 - Other (specify)
-7 - Still pregnant
-9 - NA

Table F4-1 Continued

QUEST. NO.	PREG. ORDER	FIRST PREGNANCY	SECOND PREGNANCY	THIRD PREGNANCY	FOURTH PREGNANCY
	NAME				
28) No. of hours after birth when child was first breastfed					
29) Breastfeeding until now? Reason for stopping breastfeeding					
30) Duration of breastfeeding					
31) Was breast milk supplement given? Type of supplement					
32) Age of child when given first breast milk supplement					
33) Were semi-solid and solid foods given?					
34) Age of child when semi-solid food and solid food first given					
35) Did something to end pregnancy?					
36) Who performed?					
37) What was done to end pregnancy?					
38) Who is the father of this pregnancy?					
39) Cohabiting at the time of pregnancy?					
40) FP method used for longest duration before or between the pregnancy					
41) Work during this pregnancy?					
42) Work after this pregnancy?					
43) Performed household chores: a) During pregnancy b) One month after delivery					

Index Child's Child (ICC'S) ANTHROPOMETRY:

(44) Line Number of ICC
-9 - NA

(45) Date of birth of ICC
ENTER IN MONTH DAY AND YEAR
-9 - NA

(46) Weight of ICC
TAKE THREE MEASUREMENTS IN KILOGRAMS
-9 - NA

(47) Height of ICC
TAKE THREE MEASUREMENTS IN CENTIMETERS
-9 - NA

(48) Date of measurement
RECORD IN MONTH/DAY/YEAR
-9 - NA

Table F4-1 Continued

QUEST. NO.	PREG. ORDER	FIRST PREGNANCY	SECOND PREGNANCY	THIRD PREGNANCY	FOURTH PREGNANCY
	NAME				
44) Line no. of ICC					
45) Date of birth of ICC (MO/DAY/YR)					
46) Weight of ICC (IN KGS.)	1)				
	2)				
	3)				
47) Height of ICC (IN CMS.)	1)				
	2)				
	3)				
48) Date of measurement (MO/DAY/YR)					

AFTER LAST PREGNANCY, ASK:

F4.45 Did you have sexual relations in the past week?

0 - No GO TO F4.47
1 - Yes CONTINUE
-8 - NR/DK GO TO F4.47
-9 - NA

F4.46 How many days last week did you have sexual relations?

1 - One day
:
-8 - NR/DK
-9 - NA (None during last week)

MARRIAGE/COHABITATION

@F4.47 How many times have you been married (including live-in or cohabitation)?

0 - Never GO TO NEXT BLOCK
1 - Once
:
-8 - NR/DK
-9 - NA

FOR EACH MARRIAGE/COHABITATION, ASK QUESTIONS F4.48 TO F4.53. ENTER RESPONSES IN THE TABLE BELOW

F4.48 When was this marriage/cohabitation happened? ENTER RESPONSE IN MONTH AND YEAR

-8 - NR/DK
-9- NA

F4.49 Was the marriage done in church, by a judge or consensual union? Or, were you married in church, civil or agreed to cohabit?

1 - Church
2 - Civil
3 - Consensual union
-9 - NA

F4.50 Did you elope before you get married/cohabited?

0 - No
1 - Yes
-9 - NA

F4.51 How long was the marriage/cohabitation? ENTER RESPONSE IN NUMBER OF MONTHS

-8 - NR/DK
-9- NA

F4.52 What was the reason for the termination of marriage/cohabitation?

1 - Death of partner
2 - Separation
3 - Desertion by respondent
4 - Desertion by partner
-8- NR/DK
-9 - NA

F4.53 How many pregnancies did you have during this marriage/cohabitation (including current pregnancy)?

-8 - NR/DK
-9- NA

Marriage/Cohabitation	F4.48. Date of marriage	F4.49. Type of marriage	F4.50. Eloped?	F4.51. Duration of marriage	F4.52. Reason for termination of marriage	F4.53. Total number of pregnancies
First marriage						
Second marriage						
Third marriage						
Fourth marriage						
Fifth marriage						

END OF BLOCK F

BLOCK G: DECISION MAKING, FINANCIAL MANAGEMENT AND PARENTAL STATUS

I. DECISION MAKING

DETERMINE IF INDEX CHILD IS MARRIED/COHABITING OR NOT. IF IC HAS A PARTNER OR SPOUSE, ASK QUESTIONS G1a TO G1f; IF NOT, USE THE ALTERNATE SET OF QUESTIONS, G1g TO G1j. ALL RESPONSES ARE TO BE RECORDED IN THE APPROPRIATE COLUMNS OF TABLE G-1.

FOR INDEX CHILDREN IN A COHABITING RELATIONSHIP:

G1 I would like to know how some decisions in your current household are made. I will name some of these decisions. If you have never made this type of decision in your household, think about what would happen should this decision come up. For each decision listed, please tell me:
RECORD ALL RESPONSES IN TABLE G-1.

a) Have you/your household ever made this decision?

0 - No DO NOT USE THIS FOR SKIP PATTERN. INSTEAD ASK REMAINING QUESTIONS OF ALL RESPONDENTS AS HYPOTHETICAL.
1 - Yes

b) Who contributes (would contribute) to this decision?

1 -	IC himself/herself	7 -	Other male relative
2 -	Partner/Spouse	8 -	Other female relative
3 -	Father	9 -	Non-household member,
4 -	Mother		specify relationship ______________________________
5 -	Father-in-law	-8 -	NR
6 -	Mother-in-law		

c) If a decision required a total of, say, 6 votes, how many votes would each person contributing to the decision have? (e.g. husband and wife with a completely joint decision would have 3 votes each; or husband, wife and mother-in-law would have 2 votes each).

d) Whose decision prevails/prevailed on this decision?

0 - Joint (specify) IN ADDITION TO THE CODES IN G1b

e) IF ICS DECISION DID NOT PREVAIL, ASK: Are you usually satisfied with the decision being made?

0 - No
1 - Yes
-9 - IC's decision prevailed

f) On a scale of 1 to 3, with 1 not important, 2 important and 3 very important, how important is this decision to you?

FOR INDEX CHILDREN WHO ARE NOT IN A COHABITING RELATIONSHIP:
(CROSS OUT COLUMNS G1a AND G1e IN TABLE G-1)

G1 I would like to know how you think decisions will be made in your household once you are in a cohabiting relationship. I realize that this is hypothetical, but I want you to imagine what it will be like. I will name some decisions that you may have to make. Think about what would happen should this decision come up in the next 6 months. For each decision, please tell me:

RECORD ALL RESPONSES IN TABLE G-1.

g) Who will contribute to this decision?
SAME CODES AS IN G1b

h) If a decision required a total of, say, 6 votes, how many votes would each person contributing to the decision have? (e.g. husband and wife with a completely joint decision would have 3 votes each; or husband, wife and mother-in-law would have 2 votes each).

i) Whose decision will prevail on this decision?
SAME CODES AS IN G1d

j) On a scale of 1 to 3, with 1 not important, 2 important and 3 very important, how important is this decision to you?

Table G-1. Decision-making in Household

Situation	Ever made this decision? (G1a)	Who contributes/will contribute to this decision? (G1b or G1g)	How many votes would this person get? (G1c or G1h)	Whose decision prevails? (G1d or G1i)	Usually satisfied with the decision? (G1e)	How important is decision to you? (G1f or G1j)
Buying your shoes?						
Buying clothes for your children?						
Bringing your child to the doctor?						
Buying major appliances e.g. TV?						
Buying or selling land?						
Where to send your children for schooling?						
Where you can go with your friends?						
When you should seek health care?						
If you were to study?						
If you were to work outside the home?						
If you were to visit your parents, relatives, friends outside Cebu?						
What gift to give your own relatives?						
Hiring of household help?						
If you were to transfer residence?						
Whether to use family planning or not?						
What FP method to use?						

FOR ALL INDEX CHILDREN WHETHER COHABITING OR NOT:

G2 Who is recognized as the head of your household?

CODE IN RELATIONSHIP TO RESPONDENT ☐

1 - IC
2 - Spouse/Partner
3 - Mother/Stepmother
4 - Father/Stepfather
5 - Mother-in-law
6 - Father-in-law
10 - Other adult male, specify ________________________
11 - Other adult female, specify ______________________
12 - Other (specify) _______________________________

II FINANCIAL MANAGEMENT

G3 Is it important to you to have some money that you earned on your own? ☐

0 - No
1 - Yes
2 - Unsure
-9 - NA

G4 If you had a real worry about money, who would you primarily talk it over with? ☐

0 - No one
1 - Spouse/Partner
2 - Parents
3 - Parents-in-law
4 - Other relative
5 - Friend
6 - Lending individual/institution
7 - Other (specify) ___________________________
-9 - NA

G5 Do you usually find that you can cope with major household bills and expenses?

1 - Can usually cope
2 - Sometimes difficult to cope
3 - Always difficult to cope
-7- IC/Couple not responsible for major household bills/expenses
-9 - NA

G6 In the last 12 months, have you skipped a meal due to lack of money or to save money?

0 - No GO TO G8
1 - Yes

G7 How often did you skip meals due to lack of money or to save money in the last 12 months?

1 - Once a month or less
2 - More than once a month
3 - Almost everyday
-8 - NR
-9 - NA

ASK G8-G20 ONLY OF COHABITING IC. IF IC IS NOT COHABITING, GO TO INSTRUCTIONS BEFORE G28.
FOR IC EARNING AN INCOME, ASK G8-G9; OTHERWISE SKIP TO G10

G8 How do you feel about your income; is it yours alone or do you regard it as your spouse's/partner's as well?

1 - Own
2 - Both
3 - Unsure
-9 - NA

G9 Would your household have enough to live on without your income?

0 - No
1 - Yes
2 - Unsure
-9 - NA

G10 If your spouse/partner earns enough to pay for household expenses, would you want to do or go on doing paid work?

0 - No
1 - Yes
2 - Unsure
-9 - NA

G11 If your spouse/partner is working, does he give you the money he earns?

0 - No, nothing
1 - Yes, all
2 - Yes, part How large a part? _________ (in %)
3 - Spouse/Partner has not worked since 2002 survey
-9 - NA

G12 How do you feel about what your spouse/partner earns; do you feel it is your spouse's/partner's income or do you regard it as yours as well?

1 - Spouse's/Partner's
2 - Both
3 - Spouse has not worked since 2002 survey
-9 - NA

G13 Who in your household keeps track of your and your spouse's/partner's (the couple's) money and how it is spent?

1 - IC
2 - Spouse/Partner
3 - Both
4 - No one
5 - Other (specify) ____________________________
-9 - NA

G14 Who would you say really controls the money that you as a couple have?

1 - IC
2 - Spouse/Partner
3 - Both
4 - No one
5 - Other (specify) ______________________________
-9 - NA

G15 Does your spouse/partner have an idea of how much it costs to run the household?

0 - No
1 - Yes, very well
2 - Yes, somewhat
-9 - NA

G16 How often do you and your spouse/partner argue about money?

0 - Never
1 - Rarely (a few times a year)
2 - Sometimes (once a month)
3 - Frequently (more than once a month)
-9 - NA

G17 Does your spouse/partner know how much money you spend on yourself?

0 - No
1 - Yes
-9 - NA

G18 Do you feel you need to justify to your spouse/partner spending money on some of the things you buy?

0 - No
1 - Yes
-9 - NA

G19 Are there things you would like to buy and feel you can afford but don't buy because you feel your spouse/partner may not approve?

0 - No
1 - Yes
-9 - NA

G20 Would you say that in general you and your spouse/partner have separate areas of responsibility when it comes to buying things or paying bills?

1 - Separate areas
2 - Shared responsibility (does not matter)
3 - DK
4 - Other, specify ____
-9 - NA

ASK G21 - G27 OF IC THEN OF THE SPOUSE/PARTNER AND ENTER CODED RESPONSES IN TABLE G-2

G21 For extras, things like a drink out with friends, a present for your spouse/partner, getting your hair cut or done, do you (does your spouse/partner):

1 - Take it out of your own (his own) earnings?
2 - Take it out of housekeeping allowance?
3 - Have separate money set aside for this?
4 - Take it out of a common pool?
5 - Other (specify) ____
-9 - NA

G22 Do you (your spouse/partner) have any money that is put aside for personal spending?

0 - No GO TO G27
1 - Yes
-9 - NA

G23 Is this money separate from the housekeeping money?

0 - No
1 - Yes
-9 - NA

G24 Do you (your spouse/partner) have a set amount for personal spending?

0 - No GO TO G27
1 - Yes
-9 - NA

G25 How much on average do you (does your spouse/partner) have a week?

-9 - NA

G26 Does your spouse/partner (Do you) know how this money is spent?

0 - No
1 - Yes
-9 - NA

G27 If you (your spouse/partner) made a special effort to earn extra money, do you feel the money is somehow more yours (your spouse's/partner's) to spend as you like (your spouse/partner likes)?

0 - No
1 - Yes
2 - DK
-9 - NA

Table G-2. Couple's Personal Expenses

	IC	SPOUSE/PARTNER
G21 Source for extras?		
G22 Have money for personal spending?		
G23 Separate from housekeeping money?		
G24 Have set amount?		
G25 How much a week? (in Pesos)		
G26 Know how this is spent?		
G27 Feel extra money is yours (your spouse) to spend?		

III. PARENTAL STATUS

FOR NEVER MARRIED RESPONDENTS, GO TO G31

ASK G28-G30 IF IC WAS NOT INTERVIEWED IN 2002 OR IF CURRENT OR MOST RECENT RELATIONSHIP IS DIFFERENT FROM 2002. OTHERWISE, GO TO G32.

G28 Referring back to your current or most recent relationship, at the time that you were married or entered into your last cohabiting relationship was:

Your father alive? 0 - No 1 - Yes -8 - DK -9 - Never married ☐

Your spouse's/partner's father alive? 0 - No 1 - Yes -8 - DK -9 - Never married ☐

Your mother alive? 0 - No 1 - Yes -8 - DK -9 - Never married ☐

Your spouse's/partner's mother alive? 0 - No 1 - Yes -8 - DK -9 - Never married ☐

G29 Referring back to your current or most recent relationship, at the time that you were married or entered into your last cohabiting relationship was your mother's educational level:

1 - Higher than your spouse's/partner's mother's educational level
2 - Lower than your spouse's/partner's mother's educational level
3 - The same as your spouse's/partner's mother's educational level
-8 - NR/DK
-9 - Never married

G30 Referring back to your current or most recent relationship, at the time that you were married or entered into your last cohabiting relationship was your mother's economic situation:

1 - Higher than your spouse's/partner's mother's economic situation
2 - Lower than your spouse's/partner's mother's economic situation
3 - The same as your spouse's/partner's mother's economic situation
-8 - NR/DK
-9 - Never married

G31 IF IC WAS NOT INTERVIEWED IN 2002, ASK QUESTIONS G31a - G31c OF IC'S FATHER, MOTHER, MATERNAL AND PATERNAL GRANDFATHER ANDGRANDMOTHER. RECORD ALL RESPONSES IN TABLE G-3, OTHERWISE, GO TO G32

a) What is his/her highest grade completed?
-8 - DK

b) What is his/her main occupation for most of his/her adult life?
BE PRECISE IN JOB DESCRIPTION.
-8 - DK

c) Is your father/mother taller than you are?

0 - No
1 - Yes
2 - Same height
-8 - DK

Is your mother's father/mother taller than she is?

0 - No
1 - Yes
2 - Same height
-8 - DK

Is your father's father/mother taller than he is?

0 - No
1 - Yes
2 - Same height
-8 - DK

Table G-3

	Highest Grade<br><br>G31a	Main Occupation For Most Adult Life<br>G31b	Relative Height<br><br>G31c
IC's father			
IC's mother			
Father of IC's mother			
Mother of IC's mother			
Father of IC's father			
Mother of IC's father			

G32 How many siblings do you have (including those who are now dead)? ☐☐

G33 What is your birth order? ☐☐

G34 For each of your living siblings, starting from the oldest to the youngest, tell me their:

a) Name
b) Sex
c) Age as of last birthday
d) Highest grade completed
e) Current job (main)

ENTER RESPONSES IN TABLE G-4 BELOW

Table G-4. IC's Siblings

NAME OF SIBLING<br><br>G34a	SEX<br><br>G34b	AGE<br><br>G34c	HIGHEST GRADE COMPLETED<br>G34d	CURRENT MAIN JOB<br><br>G34e

END OF BLOCK G

BLOCK H: AGGRESSION/INTIMATE PARTNER VIOLENCE MODULE

H1 No matter how well people gets along, there are times when they disagree on major decisions, get annoyed about something the person does, or just have spats or fights. I am going to read a list of things or behavior that you might have done or experienced when you had a dispute. I would like you to tell me if this ever happened with a close friend or family member, ever happened with a partner, happened in the past year or happened during the last pregnancy, and how frequent it was:

ENTER RESPONSES IN THE APPROPRIATE COLUMNS IN TABLE H-1.

a) Ever done by respondent to:
ENCIRCLE CODE

0 - No one
1 - Current partner
2 - Family member
3 - Close friend
4 - Other person, specify _____________

b) Ever done to respondent by:
ENCIRCLE CODE

0 - No one
1 - Current partner
2 - Family member
3 - Close friend
4 - Other person, specify _____________

c) Done by respondent in the past year to?
ENCIRCLE CODE

0 - No one
1 - Current partner
2 - Family member
3 - Close friend
4 - Other person, specify _____________
-9 - NA

FOR CODES 1 - 4, ASK: How often?

1 - Rarely (a few times a year)
2 - Sometimes (once a month)
3 - Frequently (more than once a month)
-9 - NA

ENTER CODED RESPONSE

d) Done to respondent in the past year by?
ENCIRCLE CODE

0 - No one
1 - Current partner
2 - Family member
3 - Close friend
4 - Other person, specify _____________
-9 - NA

FOR CODES 1 - 4, ASK: How often?

1 - Rarely (a few times a year)
2 - Sometimes (once a month)
3 - Frequently (more than once a month)
-9 - NA

ENTER CODED RESPONSE

GO TO INSTRUCTION BEFORE H2 IF IC IS MALE, OTHERWISE, CONTINUE

e) Done by respondent during last pregnancy to?
USE SAME CODES AS IN H1c

f) Done to respondent during last pregnancy by?
USE SAME CODE AS IN H1d

Table H-1

	a) Ever done by respondent to:	b) Ever done to respondent by:	c) Done by respondent in past year to:<br><br>FOR CODES 1 - 4, ASK: How often?	d) Done to respondent in past year by:<br><br>FOR CODES 1 - 4, ASK: How often?	e) Done by respondent during last pregnancy to:<br><br>FOR CODES 1 - 4, ASK: How often?	f) Done to respondent during last pregnancy by:<br><br>FOR CODES 1 - 4, ASK: How often?
Discussed the issue calmly	0 1 2 3 4	0 1 2 3 4	0 - _________<br>1 - _________<br>2 - _________<br>3 - _________<br>4 - _________	0 - _________<br>1 - _________<br>2 - _________<br>3 - _________<br>4 - _________	0 - _________<br>1 - _________<br>2 - _________<br>3 - _________<br>4 - _________	0 - _________<br>1 - _________<br>2 - _________<br>3 - _________<br>4 - _________
Got information to back up side of the argument	0 1 2 3 4	0 1 2 3 4	0 - _________<br>1 - _________<br>2 - _________<br>3 - _________<br>4 - _________	0 - _________<br>1 - _________<br>2 - _________<br>3 - _________<br>4 - _________	0 - _________<br>1 - _________<br>2 - _________<br>3 - _________<br>4 - _________	0 - _________<br>1 - _________<br>2 - _________<br>3 - _________<br>4 - _________
Brought in or tried to bring in someone to help settle things	0 1 2 3 4	0 1 2 3 4	0 - _________<br>1 - _________<br>2 - _________<br>3 - _________<br>4 - _________	0 - _________<br>1 - _________<br>2 - _________<br>3 - _________<br>4 - _________	0 - _________<br>1 - _________<br>2 - _________<br>3 - _________<br>4 - _________	0 - _________<br>1 - _________<br>2 - _________<br>3 - _________<br>4 - _________
Excessively nagged	0 1 2 3 4	0 1 2 3 4	0 - _________<br>1 - _________<br>2 - _________<br>3 - _________<br>4 - _________	0 - _________<br>1 - _________<br>2 - _________<br>3 - _________<br>4 - _________	0 - _________<br>1 - _________<br>2 - _________<br>3 - _________<br>4 - _________	0 - _________<br>1 - _________<br>2 - _________<br>3 - _________<br>4 - _________
Yelled or insulted one	0 1 2 3 4	0 1 2 3 4	0 - _________<br>1 - _________<br>2 - _________<br>3 - _________<br>4 - _________	0 - _________<br>1 - _________<br>2 - _________<br>3 - _________<br>4 - _________	0 - _________<br>1 - _________<br>2 - _________<br>3 - _________<br>4 - _________	0 - _________<br>1 - _________<br>2 - _________<br>3 - _________<br>4 - _________
Swore at the other one	0 1 2 3 4	0 1 2 3 4	0 - _________<br>1 - _________<br>2 - _________<br>3 - _________<br>4 - _________	0 - _________<br>1 - _________<br>2 - _________<br>3 - _________<br>4 - _________	0 - _________<br>1 - _________<br>2 - _________<br>3 - _________<br>4 - _________	0 - _________<br>1 - _________<br>2 - _________<br>3 - _________<br>4 - _________
Sulked or refused to talk about argument	0 1 2 3 4	0 1 2 3 4	0 - _________<br>1 - _________<br>2 - _________<br>3 - _________<br>4 - _________	0 - _________<br>1 - _________<br>2 - _________<br>3 - _________<br>4 - _________	0 - _________<br>1 - _________<br>2 - _________<br>3 - _________<br>4 - _________	0 - _________<br>1 - _________<br>2 - _________<br>3 - _________<br>4 - _________
Stomped out of the room	0 1 2 3 4	0 1 2 3 4	0 - _________<br>1 - _________<br>2 - _________<br>3 - _________<br>4 - _________	0 - _________<br>1 - _________<br>2 - _________<br>3 - _________<br>4 - _________	0 - _________<br>1 - _________<br>2 - _________<br>3 - _________<br>4 - _________	0 - _________<br>1 - _________<br>2 - _________<br>3 - _________<br>4 - _________
Threw or smashed at something (but not at anyone)	0 1 2 3 4	0 1 2 3 4	0 - _________<br>1 - _________<br>2 - _________<br>3 - _________<br>4 - _________	0 - _________<br>1 - _________<br>2 - _________<br>3 - _________<br>4 - _________	0 - _________<br>1 - _________<br>2 - _________<br>3 - _________<br>4 - _________	0 - _________<br>1 - _________<br>2 - _________<br>3 - _________<br>4 - _________
Had something in your hand to throw at anyone, but didn't throw it	0 1 2 3 4	0 1 2 3 4	0 - _________<br>1 - _________<br>2 - _________<br>3 - _________<br>4 - _________	0 - _________<br>1 - _________<br>2 - _________<br>3 - _________<br>4 - _________	0 - _________<br>1 - _________<br>2 - _________<br>3 - _________<br>4 - _________	0 - _________<br>1 - _________<br>2 - _________<br>3 - _________<br>4 - _________
Threw something at anyone	0 1 2 3 4	0 1 2 3 4	0 - _________<br>1 - _________<br>2 - _________<br>3 - _________<br>4 - _________	0 - _________<br>1 - _________<br>2 - _________<br>3 - _________<br>4 - _________	0 - _________<br>1 - _________<br>2 - _________<br>3 - _________<br>4 - _________	0 - _________<br>1 - _________<br>2 - _________<br>3 - _________<br>4 - _________
Pushed, grabbed, or shoved one	0 1 2 3 4	0 1 2 3 4	0 - _________<br>1 - _________<br>2 - _________<br>3 - _________<br>4 - _________	0 - _________<br>1 - _________<br>2 - _________<br>3 - _________<br>4 - _________	0 - _________<br>1 - _________<br>2 - _________<br>3 - _________<br>4 - _________	0 - _________<br>1 - _________<br>2 - _________<br>3 - _________<br>4 - _________
Hit one (not with anything)	0 1 2 3 4	0 1 2 3 4	0 - _________<br>1 - _________<br>2 - _________<br>3 - _________<br>4 - _________	0 - _________<br>1 - _________<br>2 - _________<br>3 - _________<br>4 - _________	0 - _________<br>1 - _________<br>2 - _________<br>3 - _________<br>4 - _________	0 - _________<br>1 - _________<br>2 - _________<br>3 - _________<br>4 - _________
Hit one with something hard	0 1 2 3 4	0 1 2 3 4	0 - _________<br>1 - _________<br>2 - _________<br>3 - _________<br>4 - _________	0 - _________<br>1 - _________<br>2 - _________<br>3 - _________<br>4 - _________	0 - _________<br>1 - _________<br>2 - _________<br>3 - _________<br>4 - _________	0 - _________<br>1 - _________<br>2 - _________<br>3 - _________<br>4 - _________
Harmed one enough to need medical attention	0 1 2 3 4	0 1 2 3 4	0 - _________<br>1 - _________<br>2 - _________<br>3 - _________<br>4 - _________	0 - _________<br>1 - _________<br>2 - _________<br>3 - _________<br>4 - _________	0 - _________<br>1 - _________<br>2 - _________<br>3 - _________<br>4 - _________	0 - _________<br>1 - _________<br>2 - _________<br>3 - _________<br>4 - _________

IF IC IS CURRENTLY MARRIED/COHABITING OR CURRENTLY IN A ROMANTIC RELATIONSHIP (WITH BOYFRIEND/GIRLFRIEND, CONTINUE; OTHERWISE GO TO INSTRUCTION BEFORE H10.

H2 I am going to ask you some questions about your relationship with your husband/wife/ partner/ boyfriend/ girlfriend. Please tell me if you agree or disagree.
(SOME QUESTIONS MAY BE HYPOTHETICAL)

Table H-2.

	0 - No<br>1 - Yes<br>-8 - NR
Your husband/wife/partner/boyfriend/girlfriend always wants to know where you are.	
Your husband/wife/partner/boyfriend/girlfriend tells you who you can spend time with.	
You feel trapped or stuck in your relationship.	
Your husband/wife/partner/boyfriend/girlfriend does what he/she wants, even if you don't want him/her to.	
When you and your husband/wife/partner/boyfriend/girlfriend disagree, he/she gets his/her way most of the time.	
Your husband/wife/partner/boyfriend/girlfriend won't let you wear certain things.	
Because your husband/wife/partner/boyfriend/girlfriend buys you things, you want to please him/her.	
**For girls:** If you ask your husband/partner/boyfriend to use a condom, he would get angry.<br>**For boys:** Your wife/partner/girlfriend would get angry if you use condom.	
Your husband/wife/partner/boyfriend/girlfriend is having sex or has had sex with someone else (within marriage).	

H3 What often causes your husband/wife/partner/boyfriend/girlfriend to hurt you?

VERBATIM: ____

____

-9 - NA (No one hurts the other)

H4 What often causes you to hurt your husband/wife/partner/boyfriend/girlfriend?

VERBATIM: ____

____

-9 - NA (No one hurts the other)

H5 Has your husband/wife/partner/boyfriend/girlfriend ever threatened to use or actually used a gun, knife or other weapon against you?

0 - No
1 - Yes
-9 - NA (Does not experience any quarrel)

H6 The last time you and your husband/wife/partner/boyfriend/girlfriend had a fight, who started it?

1 - Myself
2 - Partner
-9 - NA (Does not experience any quarrel)

H7 The last time a fight between you and your husband/wife/partner/boyfriend/girlfriend turned violent, who started it?

1 - Myself
2 - Partner
-9 - NA (No one hurts the other)

H8 Have you ever had sex with your husband/wife/partner/boyfriend/girlfriend when you did not want to because you were afraid of what he/she might do?

0 - No
1 - Yes
-8 - NR/DK

H9 Usually, when you and your husband/wife/partner/boyfriend/girlfriend disagree, how do you resolve the disagreement?

VERBATIM: ____

____

-8 - NR/DK

GO TO H13 IF IC WAS IN TERVIEWED IN 2002; OTHERWISE CONTINUE.

H10 Do you remember if either of your parents/caretakers ever hit, slapped, kicked, or used other means like pushing or shoving to try to hurt the other physically when you were growing up?

0 - No GO TO H12
1 - Yes
-8 - NR/DK

H11 Who hurt the other physically?

1 - Mother
2 - Father
3 - Both
4 - Other, specify ____________________________
-9 - NA

H12 Do you ever recall one of your parents/caretakers needing medical attention as a result of being physically hurt by the other parent/caretaker?

0 - No
1 - Yes
-8 - NR/DK

H13 I would like to ask you some questions that will tell us how you might assess certain situations.

INTERVIEWER: READ THE SITUATIONS AND RECORD RESPONSES IN TABLE H-3.

Table H-3

SITUATION	RESPONSE		
What happens to you is usually your own doing.	0 - No	1 - Yes	-8 - Don't know
Do you often feel that you don't have enough control over the direction your life is taking?	0 - No	1 - Yes	-8 - Don't know
When you plan to do something in your life, is it usually up to you for such a plan to work out?	0 - No	1 - Yes	-8 - Don't know
It is not always wise to plan too far ahead, because many things turn out to be a matter of good or bad fortune anyhow.	0 - No	1 - Yes	-8 - Don't know
Usually, does getting what you want have nothing to do with luck?	0 - No	1 - Yes	-8 - Don't know
Do you often feel that your choices could be made just as well by flipping a coin?	0 - No	1 - Yes	-8 - Don't know
Do you often find that you have little influence over the things that happen to you?	0 - No	1 - Yes	-8 - Don't know
Chance and luck do not play an important part in your life.	0 - No	1 - Yes	-8 - Don't know
When a poor person becomes rich, do you think it is above all due to his/her destiny?	0 - No	1 - Yes	-8 - Don't know
Do you usually have the courage to let someone of the opposite sex know that you fancy him/her?	0 - No	1 - Yes	-8 - Don't know
If it is possible, would you like to change something about your body (e.g., height, skin color, nose, hair, etc.)?	0 - No	1 - Yes	-8 - Don't know
Your sister, who is not married, gets pregnant. Do you think that this is God's will?	0 - No	1 - Yes	-8 - Don't know
You get up one day and no one in your family speaks to you. Do you feel that you have done something wrong?	0 - No	1 - Yes	-8 - Don't know
Do you think that your health sometimes depends upon your behavior?	0 - No	1 - Yes	-8 - Don't know
Do you think that you have to have sex with someone for them to be able to love you?	0 - No	1 - Yes	-8 - Don't know
**For girls**: You ask your partner to wear a condom when you are just about to have sex but he refuses. Would you call off the sexual encounter? **For boys**: You want to wear a condom when having sex but your partner refuses. Would you call off the sexual encounter?	0 - No	1 - Yes	-8 - Don't know

H14 Sometimes a husband is annoyed or angered by things that his wife does. In your opinion, is a husband justified in hitting or beating his wife in the following situations:

SITUATION	RESPONSE		
If she goes out without telling him?	0 - No	1 - Yes	-8 - Don't know
If she neglects the children?	0 - No	1 - Yes	-8 - Don't know
If she argues with him?	0 - No	1 - Yes	-8 - Don't know
If she refuses to have sex with him?	0 - No	1 - Yes	-8 - Don't know
If she burns the food?	0 - No	1 - Yes	-8 - Don't know
If she hits or throws at him first?	0 - No	1 - Yes	-8 - Don't know
If she is suspected for being unfaithful?	0 - No	1 - Yes	-8 - Don't know

H15 Husbands and wives do not always agree on everything. Please tell me if you think a wife is justified in refusing to have sex with her husband if…

SITUATION	RESPONSE
She is tired and not in the mood?	0 - No 1 - Yes -8 - Don't know
She has recently given birth?	0 - No 1 - Yes -8 - Don't know
She knows her husband has sex with other women?	0 - No 1 - Yes -8 - Don't know
She knows her husband has a sexually transmitted disease?	0 - No 1 - Yes -8 - Don't know

H16 Do you think that if a woman refuses to have sex with her husband when he wants her to, he has the right to…

SITUATION	RESPONSE
Get angry and reprimand her?	0 - No 1 - Yes -8 - Don't know
Refuse to give her money or other means of financial support?	0 - No 1 - Yes -8 - Don't know
Use force and have sex with her even if she doesn't want to?	0 - No 1 - Yes -8 - Don't know
Hit or beat and have sex with her even if she doesn't want to?	0 - No 1 - Yes -8 - Don't know
Go and have sex with other woman?	0 - No 1 - Yes -8 - Don't know

END OF BLOCK H

BLOCK I. INDEX CHILD'S PHYSICAL ACTIVITY

IF IC IS WORKING AND NOT IN SCHOOL, GO TO I7;

IF IC IS NOT WORKING AND NOT IN SCHOOL, GO TO I11 IF IC IS NOT LIVING WITH MOTHER OR EMPLOYER OR I16 IF IC IS LIVING WITH MOTHER

IF IC IS IN SCHOOL, CONTINUE

I1 CHECK F2.15 IF IC IS IN ELEMENTARY, HIGH SCHOOL, OR IN COLLEGE

1 - Elementary
2 - High school
3 - College

I2 How many days in a week do you attend classes?

ENTER NUMBER OF DAYS

-9 - NA

I3 How many hours do you usually attend classes in a week?

ENTER NUMBER OF HOURS

-9 - NA

I4 How do you travel to school (bus, jeepney, walk, private car, etc)?

1 - Walk
2 - Ride bicycle/'trisikad'
3 - Ride motorized vehicle (car, jeepney, bus, tricycle, etc.)
4 - Combination of 1 and 3
5 - Others, specify ____
-9 - NA

I5 How long does it take you to travel to school?

ENTER NUMBER OF MINUTES
-8 - NR/DK
-9 - NA

I6 During school days, do you have any physical activities like sports, games, gardening, etc.?

0 - No
1 - Yes
-9 - NA

GO TO INSTRUCTION BEFORE I11 IF IC IS NOT WORKING

I7 How many days in a week do you work?

ENTER NUMBER OF DAYS

-9 - NA

I8 How many hours do you usually work in a week?

ENTER NUMBER OF HOURS

-9 - NA

I9 How do you travel to your work place (bus, jeepney, walk, private car, etc)?

1 - Walk
2 - Ride bicycle/'trisikad'
3 - Ride motorized vehicle (car, jeepney, bus, tricycle, etc.)
4 - Combination of 1 and 3
5 - Others, specify ____
-9 - NA

I10 How long does it take you to travel to your work place?
ENTER NUMBER OF MINUTES

-8 - NR/DK
-9 - NA

I11 Do you have household help?

0 - No GO TO I13
1 - Yes CONTINUE

I12 How many? CODE NUMBER

-9 - NA

I13 Who performs these various household tasks?

MARK ALL HOUSEHOLD MEMBERS DOING THESE CHORES WITH X.
THE INDICATED HOUSEHOLD-MEMBER CODES IN THE TABLE MEAN:

0 - Task not done in household
1 - Respondent herself
2 - Spouse
3 - Mother or mother-in-law
4 - Father or father-in-law
5 - Other female relatives
6 - Other male relatives
7 - Hired household helper
8 - Others, specify ___________________________
-8 - NR/DK

HOUSEHOLD MEMBER CODES	0	1	2	3	4	5	6	7	8
Goes to market to buy food									
Prepares food									
Cleans up after meals									
Cleans the house									
Buys clothes									
Washes clothes									
Takes care of the children									
Fetches water									
Gathers firewood									
Tends the animals									
Tends the plants									
Does repairs at home									

FOR TASKS DONE BY MORE THAN ONE HOUSEHOLD MEMBER, ASK:

I14 Who is mainly responsible for this task?

FOR EACH TASK, ENCIRCLE X OF PERSON IN ABOVE TABLE

IF IC IS NOT WORKING OR STUDYING, GO TO I16

DAILY ACTIVITIES ON A WORKING/SCHOOL DAY

I15 Now I would like to ask about your usual activities on a working day or school day. Let's please break down a day from the time you wake to the time you sleep.

ENTER ANSWERS TO THE FOLLOWING 10 QUESTIONS IN TABLE I-1 ON THE OPPOSITE PAGE

a) What time do you usually wake up?

b) What time do you usually eat breakfast (Or, first eating time after waking up)?

c) What time do you usually eat lunch (Or, second eating time after waking up)?

d) What time do you usually eat supper (Or, third eating time after waking up)?

e) What time do you usually go to bed?

f) From the time you wake up until your breakfast/1st eating time, what are your usual activities?

g) From breakfast/1st eating time to lunchtime/2nd eating time, what are your usual activities?

h) From lunchtime/2nd eating time to suppertime/3rd eating time, what are your usual activities?

i) From suppertime/3rd eating time until the time you sleep, what are your usual activities?

j) Please tell me how long each activity you've mentioned would take (in minutes)?

INTERVIEWER: BE SURE TO HAVE THE RESPONDENT INCLUDE RESTING, TALKING WITHFRIENDS OR NEIGHBORS, TRAVEL TIME TO SCHOOL/WORK AS WELL AS USUAL HOUSEHOLD TASKS AND USUAL WORK OUTSIDE OF THE HOME. BE SURE TO INCLUDE PHYSICAL ACTIVITIES LIKE SPORTS, ETC. NOTE ACTIVITIES THAT ARE DONE SIMULTANEOUSLY, SUCH AS IRONING AND WATCHING THE CHILDREN. AFTER THE ACTIVITY ITSELF, ASK ABOUT THE APPROXIMATE TIME (TO BE RECORDED IN MINUTES) THAT RESPONDENT SPENDS ON A GIVEN TASK. IF THIS IS MORE THAN THE MORNING OR AFTERNOON OR EVENING, PROBE. THE REPORTING OF RESTING AND SLEEPING AS ACTIVITIES IS O.K.! WE DO NOT WANT IC TO REPORT LOTS OF ACTIVITIES IN ORDER NOT TO APPEAR LAZY. WHENEVER YOU FEEL THAT IC HAS REPORTED TOO MANY ACTIVITIES BUT NO PERIODS OF REST, PROBE.

Table I-1. Daily Activities on a Working/School Day

PART OF DAY	ACTIVITY (I15f, g, h, i)	TIME SPENT I15j)
I15a)<br><br>Waking-up Time:<br><br>-------------------	1	
	2	
	3	
	4	
	5	
	6	
I15b)<br><br>Breakfast Time: (1st eating time)<br><br>-------------------	1	
	2	
	3	
	4	
	5	
	6	
I15c)<br><br>Lunch Time: (2nd eating time)<br><br>-------------------	1	
	2	
	3	
	4	
	5	
	6	
	7	
	8	
	9	
	10	
I15d)<br><br>Supper Time: (3rd eating time)<br><br>-------------------	1	
	2	
	3	
	4	
	5	
	6	
	7	
	8	
	9	
	10	
I15e)<br>Bed Time:<br><br>-------------------		

I16 Now I would like to ask about your usual activities on a non-working/non-school day (e.g., weekend for those who are working/studying). Let's please break down the day from the time you wake up to the time you sleep.

ASK SAME QUESTIONS AS I15a) to I15j) AND ENTER ANSWERS IN TABLE I-2 BELOW

Table I-2. Daily Activities on a Non-working/Non-school Day

PART OF DAY	ACTIVITY (I16f, g, h, i)	TIME SPENT I16j)
I16a) Waking-up Time: -------------------	1	
	2	
	3	
	4	
	5	
	6	
I16b) Breakfast Time: (1st eating time) -------------------	1	
	2	
	3	
	4	
	5	
	6	
I16c) Lunch Time: (2nd eating time) -------------------	1	
	2	
	3	
	4	
	5	
	6	
	7	
	8	
	9	
	10	
I16d) Supper Time: (3rd eating time) -------------------	1	
	2	
	3	
	4	
	5	
	6	
	7	
	8	
	9	
	10	
I16e) Bed Time: -------------------		

I17 Are there other physical activities that you regularly participate in at least once a month that have not been mentioned above (e.g., basketball, tennis, gardening, etc.)?

0-	No	GO TO I21
1 -	Yes	CONTINUE

I18 What kind of activities do you participate in?

VERBATIM:

1______________________________

2______________________________

3______________________________

4______________________________

5______________________________

6______________________________

-9 - NA

I19 How many times per month do you participate in these activities?

ASK FOR EACH ACTIVITY MENTIONED

1 -	Once a month	ACTIVITY 1
2 -	Two times a month	ACTIVITY 2
3 -	Three times a month	ACTIVITY 3
4 -	Four times a month	ACTIVITY 4
5 -	More than four times a month	ACTIVITY 5
-8 -	NR/DK	ACTIVITY 6
-9 -	NA	

I20 How much time do you usually spend on each activity?

ASK FOR EACH ACTIVITY MENTIONED AND ENTER RESPONSE IN MINUTES

______________________ ACTIVITY 1

______________________ ACTIVITY 2

______________________ ACTIVITY 3

______________________ ACTIVITY 4

______________________ ACTIVITY 5

______________________ ACTIVITY 6

-8 - NR/DK
-9 - NA

I21 Do you work out regularly?

0-	No	GO TO I25
1 -	Yes, at home	CONTINUE
2 -	Yes, at the gym	CONTINUE

I22 What kind of activities do you do when you work out?

VERBATIM:

1____

2____

3____

4____

5____

6____

-9 - NA

I23 How many times per month do you participate in these activities when you work out?

ASK FOR EACH ACTIVITY MENTIONED

1 -	Once a month	ACTIVITY 1
2 -	Two times a month	ACTIVITY 2
3 -	Three times a month	ACTIVITY 3
4 -	Four times a month	ACTIVITY 4
5 -	More than four times a month	ACTIVITY 5
-8 -	NR/DK	ACTIVITY 6
-9 -	NA	

I24 How much time do you usually spend on each activity when you work out?

ASK FOR EACH ACTIVITY MENTIONED AND ENTER RESPONSE IN MINUTES

_______________________ ACTIVITY 1

_______________________ ACTIVITY 2

_______________________ ACTIVITY 3

_______________________ ACTIVITY 4

_______________________ ACTIVITY 5

_______________________ ACTIVITY 6

-8 - NR/DK
-9 - NA

I25 Do you do other sedentary activities such as watching video tapes, playing video or computer games, chess, etc. at least once a month? (probe for activities not yet mentioned above)

0 - No GO TO I29
1 - Yes CONTINUE

I26 What kind of activities do you participate in?

VERBATIM:

1______________________________

2______________________________

3______________________________

4______________________________

5______________________________

6______________________________

-9 - NA

I27 How many times per month do you participate in these activities?

ASK FOR EACH ACTIVITY MENTIONED

1 - Once a month	ACTIVITY 1
2 - Two times a month	ACTIVITY 2
3 - Three times a month	ACTIVITY 3
4 - Four times a month	ACTIVITY 4
5 - More than four times a month	ACTIVITY 5
-8 - NR/DK	ACTIVITY 6
-9 - NA	

I28 How much time do you usually spend on each activity?

ASK FOR EACH ACTIVITY MENTIONED AND ENTER RESPONSE IN MINUTES

______________________ ACTIVITY 1

______________________ ACTIVITY 2

______________________ ACTIVITY 3

______________________ ACTIVITY 4

______________________ ACTIVITY 5

______________________ ACTIVITY 6

-8 - NR/DK
-9 - NA

GO TO I30 IF IC IS NOT WORKING (PAID OR UNPAID)

I29 In your day-to-day life, you face different demands. Would you say your work (paid, unpaid) is less demanding, average, or more demanding in the following domains:

ENCIRCLE APPROPRIATE CODES IN TABLE I-3 BELOW

Table I-3. Demands of Work

DEMANDS	CODES	
a) Physical strength	1 - Less demanding<br>2 - Average<br>3 - More demanding	-7 - No physical strength involved<br>-9 - NA
b) Dexterity/coordination	1 - Less demanding<br>2 - Average<br>3 - More demanding	-7 - No dexterity needed<br>-9 - NA
c) Doing multiple tasks at once	1 - Less demanding<br>2 - Average<br>3 - More demanding	-7 - No multiple tasks done at once<br>-9 - NA
d) Mathematical skills	1 - Less demanding<br>2 - Average<br>3 - More demanding	-7 - No mathematical skills required<br>-9 - NA
e) Reading skills	1 - Less demanding<br>2 - Average<br>3 - More demanding	-7 - No reading skills required<br>-9 - NA
f) Getting along well with others	1 - Less demanding<br>2 - Average<br>3 - More demanding	-7 - No need to get along well with others<br>-9 - NA
g) Stress	1 - Less stressful<br>2 - Average<br>3 - More stressful	-7 - No stress involved<br>-9 - NA

I30 How about in your home life? How demanding are your household activities in terms of:

ENCIRCLE APPROPRIATE CODES IN TABLE I-4 BELOW

Table I-4. Demands of Household Activities

DEMANDS	CODES	
a) Physical strength	1 - Less demanding<br>2 - Average<br>3 - More demanding	-7 - No physical strength involved<br>-9 - NA
b) Dexterity/coordination	1 - Less demanding<br>2 - Average<br>3 - More demanding	-7 - No dexterity needed<br>-9 - NA
c) Doing multiple tasks at once	1 - Less demanding<br>2 - Average<br>3 - More demanding	-7 - No multiple tasks done at once<br>-9 - NA
d) Mathematical skills	1 - Less demanding<br>2 - Average<br>3 - More demanding	-7 - No mathematical skills required<br>-9 - NA
e) Reading skills	1 - Less demanding<br>2 - Average<br>3 - More demanding	-7 - No reading skills required<br>-9 - NA
f) Getting along well with others	1 - Less demanding<br>2 - Average<br>3 - More demanding	-7 - No need to get along well with others<br>-9 - NA
g) Stress	1 - Less stressful<br>2 - Average<br>3 - More stressful	-7 - No stress involved<br>-9 - NA

I31 During the past four (4) weeks, how often have you encountered any of the following problems with your work or other regular daily activities as a result of your physical health?

ASK THE FOLLOWING AND ENTER CODED RESPONSE IN APPROPRIATE COLUMN IN TABLE I-5

Table I-5. Problems Due to Physical Health

SITUATION	1 - None of the time<br>2 - Occasionally or some of the time<br>3 - Most or all of the time
a) Was absent/ late or requested for under-time, or, was not able to do regular daily activities	
b) Did not complete the required task	
c) Had difficulty performing the work or other activities (e.g. it took extra effort)	

I32 During the past four (4) weeks, how often have you encountered any of the following problems with your work, or other regular daily activities as a result of your emotional health (e.g. happiness, frustration, loneliness, boredom and others)?

ASK THE FOLLOWING AND ENTER CODED RESPONSE IN APPROPRIATE COLUMN IN TABLE I-6

Table I-6. Problems Due To Emotional Health

SITUATION	1 - None of the time<br>2 - Occasionally or some of the time<br>3 - Most or all of the time
a) Was absent/ late or requested for under-time, or, was not able to do regular daily activities	
b) Did not complete the required task	
c) Had difficulty performing the work or other activities (e.g. it took extra effort)	

END OF BLOCK I

BLOCK J: IC'S DIET - DAY ONE

INTERVIEWER: (a) "Usual" means at least 3 to 4 times weekly.
(b) Food preparation or eating "at home" includes any home setting or an extension of home, e.g., relative's home.
(c) Food provided at place of work is considered bought.
(d) For IC who sells food and eats what they sell, the food is bought.

J1 How many meals, excluding 'painit'/snacks, do you usually eat in a day?

CODE NUMBER OF USUAL DAILY MEALS ☐☐

J2 How many times do you usually eat 'painit'/snacks in a day?

CODE NUMBER OF USUAL DAILY SNACKS ☐☐

J3 How many times do you eat at home, excluding 'painit'/snacks in a week?

CODE NUMBER OF WEEKLY MEALS PREPARED AT HOME AND EATEN AT HOME ☐☐

J4 Excluding 'painit'/snacks, how many times do you buy ready-cooked food in a week?

CODE NUMBER OF WEEKLY MEALS PURCHASED OUTSIDE AND EATEN BY IC ☐☐

IF ONLY COOKED VIANDS ARE BOUGHT OUTSIDE BUT NOT OTHER ITEMS OF THE MEAL, CONSIDER THE ENTIRE MEAL AS BOUGHT OUTSIDE!

J5 How many times do you or your household buy 'painit'/snacks in a week? ☐☐

CODE NUMBER OF TIMES

J6 Where do you usually eat snacks and lunch?

1 - In school CONTINUE
2 - At workplace CONTINUE
3 - At home GO TO J8
4 - Elsewhere GO TO J8
Specify: ____________________________
-8 - NR/DK

'Painit'/snacks ☐☐

Lunch ☐☐

J7 If you eat in school or at workplace, where is the food prepared?

1 - Bought in or around school/at workplace
2 - Brought from home
3 - Provided free by school/workplace
-8 - NR/DK
-9 - NA

'Painit'/Snacks ☐☐

Lunch ☐☐

J8 In a month, how many times do you eat outside the home: RECORD NUMBER OF TIMES

1 - In a formal/expensive restaurant ☐☐

2 - In a fastfood place (food courts, Jollibee, etc.) ☐☐

IF RESPONSE IS NOT EQUAL TO "0", ASK:
What is/are the name(s) of the fastfood place(s) where you usually go to eat? ☐☐ ☐☐

____

____

____

3 - In a cafeteria/canteen (incl. workplace canteens) ☐☐

4 - In a carenderia/turo-turo (cheaper eating place) ☐☐

INTERVIEWER: DO NOT CODE QUESTIONS J9 THROUGH J12

J9 What do you usually eat and drink for breakfast? FOOD/DRINK 1 2 3 4 5

LIST GENERAL CATEGORIES OF FOOD SUCH AS
dried fish, pork, rice, noodles, camote, banana, etc.

________, ________, ________, ________, ________

J10 What do you usually eat and drink for lunch? FOOD/DRINK 1 2 3 4 5

LIST GENERAL CATEGORIES OF FOOD SUCH AS
dried fish, pork, rice, noodles, camote, banana, etc.

________, ________, ________, ________, ________

J11 What do you usually eat and drink for supper? FOOD/DRINK 1 2 3 4 5

LIST GENERAL CATEGORIES OF FOOD SUCH AS
dried fish, pork, rice, noodles, camote, banana, etc.

________, ________, ________, ________, ________

J12 What do you usually eat and drink for painit/snacks? FOOD/DRINK 1 2 3 4 5

LIST GENERAL TYPES OF SNACKS SUCH AS
bread, puto, biko, bodbod, etc.

________, ________, ________, ________, ________

J13 Do you regularly take vitamin or mineral supplements?

0 -	No	GO TO J15	EXCLUDE PRE- AND
1 -	Yes	CONTINUE	POSTNATAL VITAMINS
-8 -	NR/DK	GO TOJ15	AND MINERALS!!

J14 What kind? SPECIFY: ________________________

-8 - NR/DK
-9 - NA

J15 What kind of oil do you usually use for cooking?

0 - No cooking oil used in household
1 - Coconut oil ('edible', 'tinakus')
2 - Lard
3 - Others (corn oil, etc.)
4 - Combination of 1 to 3
5 - All meals bought
-8 - NR/DK

J16 How much oil do you usually use in a week?

SPECIFY QUANTITY ________________________

IN TERMS OF: lapad, tumbok, longneck, gallon, etc.
-8 - NR/DK (For households preparing food for business and consumption)
-9 - NA (No cooking oil used)

INTERVIEWER: DO NOT CODE! OFFICE: CONVERT INTO MILLILITER

J16a-e We are interested in things you might add to foods to make them salty. I am going to mention some of these items (J16a), and would like you to tell me if you use them (J16b), and if so, how often you use them (J16c), how much you would usually use (J16d), and how do you consume these food items (J16e).

J16a) Food Item	J16b) Consume?<br>0 - No<br>1 - Yes	J16c) How often?<br>1 - at every meal<br>2 - daily<br>3 - 5-6 days per wk<br>4 - 2-4 days per wk<br>5 - 1 day per wk<br>6 - seldom (less than once a week)<br>-9 - NA	J16d) Usual amount (in tsp. or record in any appropriate amount)<br><br>-9 - NA	J16e) How consumed?<br>1 - as ingredient in cooking/ added when cooked<br>2 - added when consumed /dish accompaniment<br>3 - dipping sauce<br>4 - other (specify)<br>-9 - NA<br>MULTIPLE RESPONSES ALLOWED
1 - Table salt/'asin'				
2 - Soy sauce/'toyo'				
3 - Shrimp paste/'hipon'				
4 - Salted fish/'ginamos'/ 'amahong'/ 'sisi'/'tihitihi'				
5 - Dried fish/'bulad'				
6 - Patis (e.g. Rufina)				
7 - 'Vetsin'				
8 - Others, specify:<br>a) ________________<br>b) ________________<br>c) ________________				

IC'S 24-HR FOOD RECALL
Date of interview: MONTH/DAY/YEAR ____________________________________

J17 Food Recall:

1 - Day 1
2 - Day 2

J18 Day of the week recalled:

1 - Monday
2 - Tuesday
3 - Wednesday
4 - Thursday
5 - Friday
6 - Saturday
7 - Sunday

IC'S 24-HOUR FOOD RECALL (DAY ONE)

J19 I would like you to tell me all about what you ate yesterday that is, from the time you woke up until you went to bed, including snacks.

START WITH FIRST MEAL OR SNACK OF THE DAY

0 -	Before-breakfast snack	4 -	Afternoon snack
1 -	Breakfast	5 -	Supper
2 -	Morning snack	6 -	Evening snack
3 -	Lunch		

ENTER CODE IN COL. J19 OF TABLE J-1

J20 What dishes did you have for breakfast (lunch, supper, snacks)?
WRITE NAME OF DISH IN COL. J20 OF TABLE J-1

J21 How was the dish prepared?

1 -	Boiled	6 -	Steamed
2 -	Fried	7 -	Baked
3 -	Sauteed	8 -	Processed
4 -	Broiled/roasted	9 -	Raw
5 -	Scrambled	-8 -	NR/DK

ENTER CODE IN COL. J21 OF TABLE J-1

J22 Where was the dish prepared?

1 - Home (includes any home setting or home extension like a relative's home, office)
2 - Restaurant (carenderia, cafeteria)
3 - Feeding program
4 - Ambulant food vendor, street foods
5 - Store (sari-sari, grocery, bakery, etc.)
-8 - NR/DK
-9 - NA

ENTER CODE IN COL. J22 OF TABLE J-1

J23 Where was the dish eaten?

1 -	At home	-8 -	NR/DK
2 -	Away from home	-9 -	NA

ENTER CODE IN COL. J23 OF TABLE J-1

J24 What were the ingredients (food items) composing the dish that you have eaten?

ENTER ALL FOOD ITEMS EATEN IN COL. J24 OF TABLE J-1. USE A SEPARATE LINE FOR EACH INDIVIDUAL FOOD ITEM. IF THE SAME DISH WAS EATEN AT DIFFERENT MEALS, DO NOT WRITE "SAME" BUT SPELL OUT ITEM EACH TIME!

J25 Dish Number
DISHES FORMING PART OF A MEAL ARE NUMBERED CONSECUTIVELY, STARTING WITH # 1. IF A DISH CONSISTS OF MORE THAN ONE ITEM, EACH ITEM BELONGING TO THE DISH MUST HAVE THE SAME DISH NUMBER

J26 What were the specifics of these food items?
DESCRIBE FOOD ITEM CLEARLY AS TO FORM, KIND, COLOR, SIZE, e.g., WHAT KIND OF MEAT, FISH, CEREAL, FRUIT, OR PART OF IT.

ENTER ANSWER IN COL. J26 OF TABLE J-1

J27 Food Code
TO BE ACCOMPLISHED BY DIETARY EDITORS

J28 Amount consumed
ENTER AMOUNT IN COL. J28 OF TABLE J-1

J29 Unit of Measurement

1 -	Cup	4 -	Piece
2 -	Tbsp	5 -	Pack, bottle
3 -	Matchbox (mbx)	6 -	Tsp

SPELL OUT IN COL. J29 OF TABLE J-1

J30 Was the food you ate yesterday your usual food intake? ☐

0 -	No	CONTINUE
1 -	Yes	GO TO NEXT BLOCK

J31 If not, why?

VERBATIM:____ ☐

____

-8 - NR/DK -9 - NA

Table J-1. IC's Food Recall (Day One)

MEAL CODE	NAME OF DISH	COOKING METHOD	WHERE PREPARED	WHERE EATEN	FOOD ITEMS DISH CONTAINS	DISH NUM	FOOD ITEM DESCRIPTION	FOOD CODE	AMOUNT CONSUMED	UNIT OF MEASURE	
J19	J20	J21	J22	J23	J24	J25	J26	J27	J28	J29	

IC'S 24-HR FOOD RECALL

Date of interview: MONTH/DAY/YEAR

J32 Food Recall: ☐

1 - Day 1
2 - Day 2

J33 Day of the week recalled: ☐

1 - Monday
2 - Tuesday
3 - Wednesday
4 - Thursday
5 - Friday
6 - Saturday
7 - Sunday

IC'S 24-HOUR FOOD RECALL (DAY TWO)

J34 I would like you to tell me all about what you ate yesterday that is, from the time you woke up until you went to bed, including snacks.

START WITH FIRST MEAL OR SNACK OF THE DAY

0 -	Before-breakfast snack	4 -	Afternoon snack
1 -	Breakfast	5 -	Supper
2 -	Morning snack	6 -	Evening snack
3 -	Lunch		

ENTER CODE IN COL. J34 OF TABLE J-2

J35 What dishes did you have for breakfast (lunch, supper, snacks)?

WRITE NAME OF DISH IN COL. J35 OF TABLE J-2

J36 How was the dish prepared?

1 -	Boiled	6 -	Steamed
2 -	Fried	7 -	Baked
3 -	Sauteed	8 -	Processed
4 -	Broiled/roasted	9 -	Raw
5 -	Scrambled	-8 -	NR/DK

ENTER CODE IN COL. J36 OF TABLE J-2

J37 Where was the dish prepared?

1 - Home (includes any home setting or home extension like a relative's home, office)
2 - Restaurant (carenderia, cafeteria)
3 - Feeding program
4 - Ambulant food vendor, street foods
5 - Store (sari-sari, grocery, bakery, etc.)
-8 - NR/DK
-9 - NA

ENTER CODE IN COL. J37 OF TABLE J-2

J38 Where was the dish eaten?

1 -	At home	-8 -	NR/DK
2 -	Away from home	-9 -	NA

ENTER CODE IN COL. J38 OF TABLE J-2

J39 What were the ingredients (food items) composing the dish that you have eaten?

ENTER ALL FOOD ITEMS EATEN IN COL. J39 OF TABLE J-2. USE A SEPARATE LINE FOR EACH INDIVIDUAL FOOD ITEM. IF THE SAME DISH WAS EATEN AT DIFFERENT MEALS, DO NOT WRITE "SAME" BUT SPELL OUT ITEM EACH TIME!

J40 Dish Number
DISHES FORMING PART OF A MEAL ARE NUMBERED CONSECUTIVELY, STARTING WITH # 1. IF A DISH CONSISTS OF MORE THAN ONE ITEM, EACH ITEM BELONGING TO THE DISH MUST HAVE THE SAME DISH NUMBER

J41 What were the specifics of these food items?

DESCRIBE FOOD ITEM CLEARLY AS TO FORM, KIND, COLOR, SIZE, e.g., WHAT KIND OF MEAT, FISH, CEREAL, FRUIT, OR PART OF IT.

ENTER ANSWER IN COL. J41 OF TABLE J-2

J42 Food Code

TO BE ACCOMPLISHED BY DIETARY EDITORS

J43 Amount consumed

ENTER AMOUNT IN COL. J43 OF TABLE J-2

J44 Unit of Measurement

1 -	Cup	4 -	Piece
2 -	Tbsp	5 -	Pack, bottle
3 -	Matchbox (mbx)	6 -	Tsp

SPELL OUT IN COL. J44 OF TABLE J-2

J45 Was the food you ate yesterday your usual food intake? ☐

0 -	No	CONTINUE
1 -	Yes	GO TO NEXT BLOCK

J46 If not, why?

VERBATIM:____ ☐

____

-8 -	NR/DK	-9 -	NA

Table J-2. IC's Food Recall (Day Two)

MEAL CODE	NAME OF DISH	COOKING METHOD	WHERE PREPARED	WHERE EATEN	FOOD ITEMS DISH CONTAINS	DISH NUM	FOOD ITEM DESCRIPTION	FOOD CODE	AMOUNT CONSUMED	UNIT OF MEASURE	
J34	J35	J36	J37	J38	J39	J40	J41	J42	J43	J44	

BLOCK K: MORBIDITY AND REPRODUCTIVE HEALTH OF IC

SOME INFORMATION IN THIS SECTION MAY BE AVAILABLE IN THE LHM. COPY PERTINENT INFORMATION IN THE APPROPRIATE SPACES.

I. MORBIDITY

K1 Have you ever been sick since our last visit in 2002 (or last visit)?

0 - No GO TO K4
1 - Yes CONTINUE

K2 What were you sick of?

1____

2____

3____

4____

5____

6____

-8 - NR/DK
-9 - NA

K3 Were you hospitalized because of this illness/any of these illnesses?

0 - No
1 - Yes Specify which illness(es) :____________________________
-9 - NA

@K4 Do you have a chronic illness/disability?***

0 - No GO TO K8
1 - Yes CONTINUE
-8 - NR/DK

@K5 What is your chronic illness/disability?
VERBATIM:____

____

-8 - NR/DK
-9 - NA

@K6 When was the onset of this chronic illness/disability?
RECORD IC'S RESPONSE:
____

-8 - NR/DK
-9 - NA

K7 Does this illness/disability limit your ability to attend school or to work?

0 - No
1 - Yes
-7 - IC not studying nor working
-8 - NR/DK
-9 - NA

K8 Since 2002 (or last visit), have you received any immunization?

0 - No GO TO K10
1 - Yes CONTINUE
-8 - NR/DK

K9 What type of immunization was it?
LIST VACCINATIONS, DO NOT CODE

1 ____

2 ____

-8 - NR/DK
-9 - NA

ENTER RESPONSES TO QUESTIONS K10 – K13 IN TABLE K-1

K10 Have you ever...
- smoked?
- drank alcoholic beverages?
- taken drugs?

K11 How old were you when you first tried...
- smoking?
- drinking alcoholic beverages?
- taking drugs?

K12 Who initiated you into...
- smoking?
- drinking alcoholic beverages?
- taking drugs?
VERBATIM:

K13 How often do you...
- smoke?
- drink alcoholic beverages?
- take drugs?

Table K-1

ACTIVITY	EVER TRIED	AGE WHEN 1st TRIED	PERSON WHO INITIATED	STILL DOING NOW, HOW OFTEN?
	K10	K11	K12	K13
Smoking	0 - No GO TO DRINKING<br>1 - Yes			1 - One stick/day<br>2 - Two sticks/day<br>3 - Three sticks/day<br>:<br>-6 - Smokes but not daily<br>-7 - Stopped smoking<br>-9 - NA
Drinking alcoholic beverages	0 - No GO TO DRUGS<br>1 - Yes			1 - Only occasionally<br>2 - Every week<br>3 - Every day<br>-7 - Stopped drinking<br>-9 - NA
Taking drugs	0 - No GO TO K14a<br>1 - Yes<br>(If yes, specify drug(s) tried)<br>____________<br>____________<br>____________			1 - Only occasionally<br>2 - Every week<br>3 - Every day<br>-7 - Stopped taking drugs<br>-9 - NA<br><br>Specify drug ______________<br>__________________________

K14a Do other members of your household smoke?

0 - No GO TO INSTRUCTION BEFORE K15
1 - Yes

K14b How many members of your household usually smoke inside the house?

1 - One
:

IF IC DRINKS (CODE 1, 2 OR 3 IN K13) CONTINUE; OTHERWISE GO TO K17

K15 What alcoholic drink do you usually consume?

1 - Tuba
2 - Beer
3 - Gin
4 - Other, specify ___________________________
:
-9 - NA

K16 How much do you usually consume?

SPECIFY QUANTITY IN TERMS OF BOTTLE OR GLASS ________________________________

INTERVIEWER: CONVERT INTO CUPS _____________________
-9 - NA

K17 We would like to know how your health has been in general over the past four weeks. Please answer how frequently in the past four weeks did you experience these common feelings or problems.

ENTER CODES IN TABLE K-2

1 - None of the time
2 - Occasionally
3 - Most of the time

TABLE K-2. Feelings/Problems in the Past 4 Weeks

FEELINGS/PROBLEMS	CODES
You were happy	
You had headaches	
You had poor digestion	
You had difficulty falling asleep	
You felt lonely	
You were hopeful about the future	
People were unfriendly	
You were worried	
You felt you couldn’t overcome difficulties	
You were able to face problems	
You felt people disliked you	
You enjoyed normal daily activities	
You thought of yourself as worthless	
You felt life isn’t worth living	
You wished you were dead	
You had the idea of taking your own life	

K18 How would you rate your general health?

1 - Poor
2 - Good
3 - Excellent

K19 Are you wearing eyeglasses now?

0 - No
1 - Yes GO TO K20b

K20a Do you think you need one?

0 - No
1 - Yes
-9 - NA

GO TO K21

K20b How old were you when you started wearing eyeglasses?

-8 - NR/DK
-9 - NA

K20c Are you wearing higher grade of lens (eyeglasses) than the last survey/last visit?
Nitaas ba ang grado sa imong anteyohos sukad sa katapusan namong pagbisita?

0 - No
1 - Yes
-9 - NA/Started wearing eyeglass since last survey

K21 Do you/did you have any cavities or decayed teeth?

0 - No
1 - Yes

K22a Are your teeth still complete?

0 - No
1 - Yes GO TO K23a

K22b How many teeth are lost or missing?

1 - One
:
-9 - NA

K23a Have you had your teeth filled?

0 - No GO TO K24
1 - Yes

K23b How many teeth have been filled?

1 - One
:
-9 - NA

K24 How many times do you usually take a bath in one week?

CODE NUMBER OF TIMES
-8 - NR

II. REPRODUCTIVE HEALTH

K25 I will mention a list of problems women/men may experience. Please tell me if you have experienced any of these since 2002/last visit.

0 - No
1 - Yes

ENTER ANSWER IN COL. K25 OF TABLE K-3

Table K-3. Reproductive Health Problems

PROBLEM	Experienced? K25
1. Painful urination	
2. Itchiness in vaginal area/genital area	
3. Vaginal discharges/genital discharges	
4. Other related problems, specify ______________________	
ASK 5, 6, 7 AND 8 OF INDEX GIRLS, CODE -9 OF INDEX BOYS	
5. Painful menstruation (dysmenorrhea)	
6. Irregular menstruation	
7. Unusually excessive/too little flow	
8. Other related problems, specify ______________________	

IF IC HAS NOT EXPERIENCED ANY PROBLEM, GO TO K35, OTHERWISE ASK K26

K26 Did you consult someone for any of these problems?

0 - No CONTINUE
1 - Yes GO TO K28
-9 - NA

K27 Why not?

VERBATIM: ____

____

-8 - NR/DK
-9 - NA

GO TO INSTRUCTION BEFORE K38

FILL OUT TABLE K-4 FOR DETAILS REGARDING EACH PRACTITIONER MENTIONED

K28 Who did you consult? (GET NAME OF PRACTITIONER AND ADDRESS OF CLINIC)
RECORD ANSWER IN COL. K28 OF TABLE K-4

IF IC DOESN'T KNOW NAME AND/OR ADDRESS AND HIS/HER RESPONSE TO K31 IS 0, **DO NOT GET ADDRESS FROM MOTHER/SPOUSE**, VERIFY AT OPS OR FROM SUPERVISOR.

EDITORS: ASSIGN A UNIQUE CLINIC CODE FOR EACH CLINIC MENTIONED
0 - No clinic, practices at home
1 - No clinic, does home visit
:
8 - Holy Family Clinic (example)
9 - Camputhaw Health Center (example)
-8 - NR/DK
-9 - NA

K29 Is this person a:

1 - Private doctor
2 - Private nurse
3 - Private midwife
4 - Government doctor
5 - Government nurse
6 - Government midwife
7 - School doctor
8 - School nurse
9 - Traditional practitioner
10 - Mother
11 - Father
12 - Caretaker
13 - Others, specify ___________________________________
-8 - NR/DK
-9 - NA

ENTER ANSWER IN COL. K29 OF TABLE K-4

K30 What was the diagnosis?
VERBATIM- ENTER KEY PHRASES IN COL. K30 OF TABLE K-4

K31 Were your parents/Was your spouse informed of this consultation?

0 - No
1 - Yes, specify which one
-8 - NR/DK
-9 - NA

ENTER CODE IN COL. K31 OF TABLE K-4

K32 How many times have you visited this person?

1 - Once
:
9 - Nine
-8 - NR/DK
-9 - NA

ENTER ANSWER IN COL. K32 OF TABLE K-4

K33 Would you consult this person again should you have similar problems in the future?

0 - No
1 - Yes
-8 - NR/DK
-9 - NA

ENTER CODE IN COL. K33 OF TABLE K-4

K34 How much do you usually pay this person per visit? (IN PESOS)

-8 - NR/DK
-9 - NA

ENTER ANSWER IN COLK34 OF TABLE K-4
AFTER LAST PRACTITIONER, GO TO INSTRUCTION BEFORE K38

Table K4. Practitioners consulted for reproductive health problems

Name of Practitioner/Clinic Address	Clinic Code (K28)	Type (K29)	Diagnosis (K30)	Parents/Spouse informed (K31)	Number of visits made (K32)	Will consult again? (K33)	Cost per visit (K34)

K35 Imagine you were having any of the problems I just mentioned, would you consult someone?

0 -	No	CONTINUE
1 -	Yes	GO TO K37
-8 -	NR/DK	GO TO INSTRUCTION BEFORE K38
-9 -	NA	

K36 Why not?

VERBATIM:

____

____

-8 - NR/DK
-9 - NA

GO TO INSTRUCTION BEFORE K38

K37 Who would you consult? Do you have a specific person or clinic in mind?

VERBATIM:
RECORD NAME, ADDRESS AND TYPE OF PRACTITIONER FOR EACH PERSON MENTIONED

____

____

____

-8 - NR/DK
-9 - NA

IF IC CANNOT NAME A SPECIFIC PERSON, ASK TYPE OF PRACTITIONER OR CLINIC (e.g., private doctor, government midwife, etc.)

EDITORS: ASSIGN TWO CODES FOR EACH PERSON MENTIONED

1) CLINIC CODE - To link clinic with index children-patients

0 -	No clinic, practices at home
1 -	No clinic, does home visits
2 -	Any private clinic
3 -	Any government clinic
:	
-8 -	NR/DK
-9 -	NA

2) TYPE OF PRACTITIONER

1 -	Private doctor
2 -	Private nurse
3 -	Private midwife
4 -	Government doctor
5 -	Government nurse
6 -	Government midwife
7 -	School doctor
8 -	School nurse
9 -	Traditional practitioner
10 -	Mother
11 -	Father
12 -	Caretaker
13 -	Others, specify ______________________
-8 -	NR/DK
-9 -	NA

IF IC IS SEXUALLY ACTIVE (CHECK LHM OR F4.31), CONTINUE, OTHERWISE, GO TO K51

I will read to you a list of problems women/men who are having sex may experience. INTERVIEWER: GO THROUGH EACH SYMPTOM IN TABLE K-5. FOR EACH SYMPTOM, ASK K38.

K38 Have you experienced since 2002/last visit
- 0 - No (IF NO OR NR/DK, MOVE TO NEXT SYMPTOM. IF RESPONSE IS 0 AND/OR -8 TO ALL SYMPTOMS, GO TO K48)
- 1 - Yes
- -8 - NR/DK

ENTER CODE IN COL. K38 OF TABLE K-5

K39 Did you consult someone about this problem?

- 0 - No CONTINUE
- 1 - Yes GO TO K41

ENTER CODE IN COL. K39 OF TABLE K-5

K40 Why not?
VERBATIM- ENTER KEY PHRASES IN COL. K40 OF TABLE K-5
- -8 - NR/DK
- -9 - NA

GO TO K51

K41 Who did you consult? (GET NAME OF PRACTITIONER AND ADDRESS OF CLINIC)
- -8 - NR/DK
- -9 - NA

IF IC DOESN'T KNOW NAME AND ADDRESS AND HIS/HER RESPONSE TO K44 IS 0, **DO NOT GET SUCH INFORMATION FROM MOTHER/SPOUSE**. VERIFY AT OPS OR FROM SUPERVISOR.

EDITORS: ASSIGN CLINIC CODE FOR EACH CLINIC MENTIONED
- 0 - No clinic, practices at home
- 1 - No clinic, does home visit
- :
- 8 - Holy Family Clinic (example)
- 9 - Camputhaw Health Center (example)

K42 Is this person a:

1 -	Private doctor	9 -	Traditional practitioner
2 -	Private nurse	10 -	Mother
3 -	Private midwife	11 -	Father
4 -	Government doctor	12 -	Caretaker
5 -	Government nurse	13 -	Others, specify ____________
6 -	Government midwife	-8 -	NR/DK
7 -	School doctor	-9 -	NA
8 -	School nurse		

ENTER CODE IN COL. K42 OF TABLE K-5

K43 What was the diagnosis?
VERBATIM- ENTER KEY PHRASES IN COL. K43 OF TABLE K-5
- -8 - NR/DK
- -9 - NA

K44 Were your parents/Was your spouse informed of this consultation?
- 0 - No
- 1 - Yes, specify which one
- -8 - NR/DK
- -9 - NA

ENTER ANSWER IN COL. K44 OF TABLE K-5

K45 How many times have you visited this person?
ENTER NUMBER IN COL. K45 OF TABLE K-5
- -8 - NR/DK
- -9 - NA

K46 Would you consult this person again should you have similar problems in the future?
- 0 - No
- 1 - Yes
- -8 - NR/DK
- -9 - NA

ENTER CODE IN COL. 46 OF TABLE K-5

K47 How much do you usually pay this person per visit? (IN PESOS)
ENTER ANSWER IN COL. K47 OF TABLE K-5
- -8 - NR/DK
- -9 - NA

AFTER LAST PRACTITIONER, GO TO K63

Table K-5. Practitioners Consulted by Sexually -Active IC

SYMPTOM	Expe-rienced (K38)	Consult someone (K39)	Reason for not consulting (K40)	Name of Practitioner/Address (K41)	Type (K42)	Diagnosis (K43)	Parents/ Spouse know (K44)	Number of visits made (K45)	Will consult again (K46)	Cost per visit (K47)
Vaginal discharges/ irritation; Penile discharges/ irritation										
Painful urination										
Pain during intercourse										
IF MALE: Genital warts/ulcers										
IF FEMALE: Signs of pregnancy										
Other related problems, specify ______________ ____________________										

K48 Imagine you were having any of these problems, would you consult someone?

0 -	No	CONTINUE
1 -	Yes	GO TO K50
-8 -	NR/DK	GO TO K51
-9 -	NA	

K49 Why not?
VERBATIM:

____

____

-8 - NR/DK
-9 - NA

GO TO K51

K50 Who would you consult? Do you have specific person or clinic in mind?

RECORD NAME, ADDRESS AND TYPE OF PRACTITIONER FOR EACH PERSON MENTIONED
VERBATIM:

____

____

____

-8 - NR/DK
-9 - NA

IF IC CANNOT NAME A SPECIFIC PERSON, ASK TYPE OF PRACTITIONER OR CLINIC (e.g., private doctor, government midwife, etc.)

EDITORS: ASSIGN TWO CODES FOR EACH PERSON MENTIONED

1) CLINIC CODE - To link clinic with index children-patients

0 - No clinic, practices at home
1 - No clinic, does home visit
:
8 - Holy Family Clinic (example)
9 - Camputhaw Health Center (example)
-8 - NR/DK
-9 - NA

2) TYPE OF PRACTITIONER

1 - Private doctor
2 - Private nurse
3 - Private midwife
4 - Government doctor
5 - Government nurse
6 - Government midwife
7 - School doctor
8 - School nurse
9 - Traditional practitioner
10 - Mother
11 - Father
12 - Caretaker
13 - Others, specify ______________________________
-8 - NR/DK
-9 - NA

K51 Do you know that there are diseases one can get by having sex?

0 -	No	GO TO K57
1 -	Yes	CONTINUE
-8 -	NR/DK	GO TO K57

K52 From where did you learn about this?

VERBATIM:

___

___

-8 - NR/DK
-9 - NA

K53 How are such diseases called?

VERBATIM:

___

___

-8 - NR/DK
-9 - NA

K54 What are the symptoms?

VERBATIM:

___

___

-8 - NR/DK
-9 - NA

K55 What should one do if he or she has these symptoms?

VERBATIM:

___

___

-8 - NR/DK
-9 - NA

K56 What should one do to avoid getting such disease?

VERBATIM:

___

___

-8 - NR/DK
-9 - NA

K57 PERCEIVED STRESS SCALE

The questions in this scale ask you about your feelings and thoughts during the last four weeks. In each case, you will be asked to indicate how often you felt or thought a certain way. Although some of the questions are similar, there are differences between them and you should treat each one as a separate question. The best approach is to answer each question fairly quickly. That is, don't try to count up the number of times you felt a particular way, but rather indicate the alternative that seems like a reasonable estimate.

For each question, choose from the following alternatives:

0 - Never
1 - Almost never
2 - Sometimes
3 - Fairly often
4 - Very often

	0 - Never	1 - Almost never	2 - Sometimes	3 - Fairly often	4 - Very often
1. In the last 4 weeks, how often have you been upset because of something that happened unexpectedly?					
2. In the last 4 weeks, how often have you felt that you were unable to control the important things in your life?					
3. In the last 4 weeks, how often have you felt nervous and stressed?					
4. In the last 4 weeks, how often have you dealt successfully with irritating life hassles?					
5. In the last 4 weeks, how often have you felt that you were effectively coping with important changes that were occurring in your life?					
6. In the last 4 weeks, how often have you felt confident about your ability to handle your personal problems?					
7. In the last 4 weeks, how often have you felt that things were going your way?					
8. In the last 4 weeks, how often have you found that you could not cope with all the things that you had to do?					
9. In the last 4 weeks, how often have you been able to control irritations in your life?					
10. In the last 4 weeks, how often have you felt that you were on top of things?					

K58 Usually, about how many days per week do you awake feeling rested?

ENCIRCLE NUMBER

1 2 3 4 5 6 7

END OF BLOCK K

BLOCK L. FAMILY, PEER AND MEDIA INFLUENCE

FAMILY INFLUENCE

L1 Do you confide in any one in your household when you have problems?

0 - No GO TO L3
1 - Yes CONTINUE
-8 - NR/DK

L2 Whom do you usually confide in?

1 - Spouse/Partner
2 - Mother
3 - Father
4 - Brother
5 - Sister
6 - Other relatives, specify ____________________
-8 - NR/DK
-9 - NA

IF IC IS MARRIED OR COHABITING, ASK L3-L4, OTHERWISE GO TO L5

L3 Do you ever quarrel with your spouse/partner?

0 - No GO TO L5
1 - Yes CONTINUE
-8 - NR/DK GO TO L5

L4 How often have you quarreled with your spouse/partner in the past 12 months?

0 - No quarrel in the past 12 months
1 - Once a month or less
2 - More than once a month
3 - Almost every day
-8 - NR/DK
-9 - NA

L5 Do you ever quarrel with other household members?

0 - No GO TO L7
1 - Yes CONTINUE
-8 - NR/DK GO TO L7

L6 How often have you quarreled with other household members in the past 12 months?

0 - No quarrel in the past 12 months
1 - Once a month or less
2 - More than once a month
3 - Almost every day
-8 - NR/DK
-9 - NA

L7 Do your parents ever quarrel?

0 - No GO TO L9
1 - Yes CONTINUE
-8 - NR/DK GO TO L9
-9 - NA (For single, widowed, separated parent or if IC is living separately from parents) GO TO L9

L8 How often have your parents quarreled with each other in the past 12 months?

0 - No quarrel in the past 12 months
1 - Once a month or less
2 - More than once a month
3 - Almost every day
-7 - Both parents not in household the past 12 months
-8 - NR/DK
-9 - NA

L9 Are you allowed to go to discos or dances, entertainment centers (bar, videoke, internet café)?

0 - No
1 - Yes
-8 - NR/DK

L10 What time are you expected to be home in the evening?

On schooldays: ________________________

On workdays: __________________________

On school and workdays: _________________

On weekends: ___________________________

FOR THOSE WHO ARE NOT WORKING OR STUDYING, ASK:
On non-work days/non-school days: ______________________

-8 - NR/DK

IF IC IS MARRIED/COHABITING, ASK L11, OTHERWISE GO TO L12

L11 Do you think your spouse/partner is strict?

0 - No
1 - Yes
-8 - NR/DK
-9 - NA (no spouse/partner)

L12 Do you think your mother is strict? (Refer to biological mother)

0 - No
1 - Yes
-8 - NR/DK
-9 - NA

L13 Do you think your father is strict? (Refer to biological father)

0 - No
1 - Yes
-8 - NR/DK
-9 - NA

L14 I will read to you a list of activities. Please tell me if this activity is done in your household?

READ EACH ACTIVITY AND CHECK APPROPRIATE RESPONSE IN TABLE L-1

Table L-1.

ACTIVITY	0- Not done in household	1-Yes, allowed to do alone	2 - Yes, allowed to do with friends	3 - Yes, must be done with spouse/ family
Go to church				
Go to the movies				
Go to the mall (e.g., Ayala, SM, etc.)				
Go to picnic				
Visit relatives				
Other activities, specify__________________				

L15 How close do you think you are to your mother? (Refer to biological mother)

1 - Close
2 - Not close
-8 - NR/DK
-9 - NA

L16 How close do you think you are to your father? (Refer to biological father)

1 -	Close	-8 -	NR/DK
2 -	Not close	-9 -	NA

L17 Who do you consider as the person responsible for your upbringing?

1 -	Mother	4 -	Others, specify ___________________________
2 -	Father	-8 -	NR/DK
3 -	Both parents		

L18 Do you discuss the following with your mother/father (preferably the person responsible for upbringing)?

0 - No, referring to biological mother/father
1 - No, referring to stepmother/stepfather/caretaker (or foster parent)
2 - Yes, referring to biological mother/father
3 - Yes, referring to stepmother/stepfather/caretaker (or foster parent)
-8 - NR/DK
-9 - NA

READ TOPICS LISTED IN TABLE L-2. RECORD RESPONSES IN COLUMN L18 OF TABLE L-2. IF NO TOPIC IS EVER DISCUSSED, GO TO L21

FOR EACH TOPIC DISCUSSED, ASK QUESTIONS L19 TO L21 AND ENTER RESPONSE IN THE APPROPRIATE COLUMNS IN THE TABLE BELOW

L19 Who usually initiates the discussion about this topic?

0 -	IC	4 -	Step or foster parent/Caretaker initiates discussion (Coded 3 in L18)
1 -	Mother		
2 -	Father	-8 -	NR/DK
3 -	Can't remember	-9 -	NA

L20 How is this topic discussed?

1 -	Serious one-on-one talk	4 -	Others, specify _________________________
2 -	Casual conversation	-8 -	NR/DK
3 -	Indirectly (in jest, through a third person)	-9 -	NA

L21 Whose opinion do you value most on these topics?

1 -	Your own	7 -	Friends
2 -	Mother	8 -	Medical practitioner
3 -	Father	9 -	School/Teacher
4 -	Both parents	10 -	Others, specify _________________________
5 -	Caretaker	-8 -	NR/DK
6 -	Others, relatives __________________	-9 -	NA

Table L-2. Communication with IC

Topic No.	TOPIC	Is the topic discussed?	Who initiates the discussion?	How is the topic discussed?	Whose opinion you value most?
		L18	L19	L20	L21
a.	Your friends				
b.	Having crushes				
c.	Having boyfriends/girlfriends				
d.	Going out on dates				
e.	Breaking up with boyfriend/girlfriend				
f.	Marriage				
g.	Sexuality				
h.	Sexual relations				
i.	Family planning				

L21a IF FAMILY PLANNING IS DISCUSSED, ASK: What particular topic in family planning do you discuss?

____

-9 - NA

PEER INFLUENCE:

L22 How many close girl friends do you have?

ENTER NUMBER IN BOXES

0 - None GO TO L24
:
10 - Ten CONTINUE

L23 Are they:

1 - Younger than you
2 - Your age
3 - Older than you
-8 - NR/DK
-9 - NA

L24 How many close boy friends do you have?

ENTER NUMBER IN BOXES

0 - None IF L22 IS ALSO 0, GO TO L30; IF NOT, GO TO L26
:
10 - Ten CONTINUE

L25 Are they:

1 - Younger than you
2 - Your age
3 - Older than you
-8 - NR/DK
-9 - NA

L26 How often do you usually see your close friends in a week?

VERBATIM: ____

____

-8 - NR/DK
-9 - NA

L27 Do any of your close friends (both sexes) have boyfriends/girlfriends/spouses?

0 - No
1 - Yes
-8 - NR/DK
-9 - NA

L28 Do any of your close friends

smoke?	0 - No	1 -Yes	-8 -NR/DK
drink alcoholic beverages?	0 - No	1- Yes	-8 -NR/DK
take drugs?	0 - No	1- Yes	-8 -NR/DK

-9 - NA

L29 Do you think they have done the following?

1 -	Dating	0 - No	1 - Yes	-8 - NR/DK
2 -	Holding hands	0 - No	1 - Yes	-8 - NR/DK
3 -	Kissing	0 - No	1 - Yes	-8 - NR/DK
4 -	Have sex	0 - No	1 - Yes	-8 - NR/DK

-9 - NA

IF IC IS MARRIED OR COHABITING ASK L30, OTHERWISE GO TO L31.

L30 Does your spouse/partner

smoke?	0 - No	1 - Yes	-8 -NR/DK
drink alcoholic beverages?	0 - No	1 - Yes	-8 -NR/DK
take drugs?	0 - No	1 - Yes	-8 -NR/DK

-9 - NA

L31 Do you belong to any club or organization?

0 - No GO TO L33
1 - Yes CONTINUE

L32 Of what club or organization are you a member?

WRITE NAME OF CLUB OR ORGANIZATION

____

____

-8 - NR/DK
-9 - NA

EDITORS: CODE WHETHER RELIGIOUS, ACADEMIC, SPORTS-ORIENTED, ETC.

MEDIA/INFORMATION TECHNOLOGY INFLUENCE:

L33 Do you watch TV every week?

0 - No GO TO L37
1 - Yes CONTINUE

L34 How many hours of TV do you usually watch on a weekday?
ENTER RESPONSE IN NUMBER OF HOURS

-9 - NA

L35 How many hours of TV do you usually watch on a weekend?
ENTER RESPONSE IN NUMBER OF HOURS

-9 - NA

L36 What are your favorite TV shows?

WRITE NAME OF SHOW, SCHEDULE AND TV CHANNEL

____

____

-8 - NR/DK
-9 - NA

L37 Do you listen to radio every week?

0 - No GO TO L39
1 - Yes CONTINUE

L38 What kind of radio programs do you usually listen to?
WRITE NAME OF PROGRAM, SCHEDULE AND RADIO STATION

____

____

-8 - NR/DK
-9 - NA

L39 Do you read magazines/pocketbooks/comics/newspapers?

0 -	Never	GO TO L41
1 -	Yes	CONTINUE
2 -	Very seldom	GO TO L41

L40 What magazine/pocketbook/comics/newspaper do you usually read?
WRITE NAME OF READING MATERIALS

____

____

-8 - NR/DK
-9 - NA

L41 Do you surf the web?

0 -	Never	GO TO L43
1 -	Yes	CONTINUE
2 -	Very seldom	GO TO L43

L42 What web sites do you usually visit?
WRITE NAME OF WEB SITES

____

____

-9 - NA

L43 Do you send/receive text messages or emails?

0 - No
1 - Yes
-8 - NR/DK

L44 Do you read/watch/listen to pornographic shows/reading materials?

0 - No
1 - Yes
-8 - NR/DK

END OF BLOCK L

BLOCK M: IC'S ANTHROPOMETRY

Date of measurement: MONTH/DAY/YEAR ________________________________

Number of months pregnant: ________________________
-9 - Not currently pregnant

M1 WEIGHT (IN KILOGRAMS)

e.g., 30.7 [ ][ ][ ][ ]

M2 HEIGHT (IN CENTIMETERS)

e.g., 130.8 [ ][ ][ ][ ][ ]

M3 ARM CIRCUMFERENCE (IN CENTIMETERS)

e.g., 20.3 [ ][ ][ ][ ]

M4 TRICEPS SKIN-FOLD THICKNESS
INTERVIEWER: TAKE THREE MEASUREMENTS

Measurement #1 [ ][ ][ ][ ]

Measurement #2 [ ][ ][ ][ ]

Measurement #3 [ ][ ][ ][ ]

M5 SUBSCAPULAR SKIN-FOLD THICKNESS
INTERVIEWER: TAKE THREE MEASUREMENTS

Measurement #1 [ ][ ][ ][ ]

Measurement #2 [ ][ ][ ][ ]

Measurement #3 [ ][ ][ ][ ]

M6 SUPRA-ILIAC THICKNESS
INTERVIEWER: TAKE THREE MEASUREMENTS

Measurement #1 [ ][ ][ ][ ]

Measurement #2 [ ][ ][ ][ ]

Measurement #3 [ ][ ][ ][ ]

M7 WAIST CIRCUMFERENCE (cm) [ ][ ][ ]
-9- Currently pregnant

M8 HIP CIRCUMFERENCE (cm) [ ][ ][ ]
-9 - Currently pregnant

M9 BLOOD PRESSURE (mm Hg)
INTERVIEWER: TAKE THREE MEASUREMENTS

Measurement #1 [ ][ ][ ] / [ ][ ][ ]
Systolic Diastolic

Measurement #2 [ ][ ][ ] / [ ][ ][ ]
Systolic Diastolic

Measurement #3 [ ][ ][ ] / [ ][ ][ ]
Systolic Diastolic

END OF INTERVIEW

CEBU LONGITUDINAL HEALTH AND NUTRITION STUDY
NSF Pregnancy Survey

ID1 Type of Survey: [2] [0] [0] [9] [2]

ID2 Current Barangay: ____________________ [ ] [ ] [ ]
(See BARANGAY CODE LIST)

ID3 Current Stratum: 1 - Urban 2 - Rural [ ]

ID4 Baseline ID Number: [ ] [ ] [ ] BRGY [ ] [ ] [ ] [ ] HHLD [ ] [ ] [ ] WMAN

ID5 Index Child's ID Number: [ ] [ ] [ ] [ ] [ ]

ID6 Mother's ID Number: [ ] [ ] [ ] [ ] [ ]

ID7 Name of Index Child: ____________________

2009 address (as of tracking visit): ____________________

____

New address (if different from tracking address):

____

____

Contact Number: ____________________

ID8 INTERVIEWER: Is the pregnancy visit address same as the tracking address? [ ]

0 - No
1 - Yes

ID9 Name of Interviewer: ____________________ [ ] [ ]

ID10 Date of Interview Completion: [ ] [ ] MONTH [ ] [ ] DAY [ ] [ ] [ ] [ ] YEAR

ID11 PREGNANCY INTERVIEW STATUS:
Is Index Child available for this interview? [ ]

0 No (CONTINUE)
1 Yes (GO TO CALL RECORD AND PROCEED WITH MAIN INTERVIEW)

ID12 Reason why not interviewed: [ ]

1 - Index Child deceased

X1 DATE OF DEATH: [ ] [ ] Month [ ] [ ] [ ] [ ] Year

-8 - NR/DK
-9 - NA

X2 CAUSE OF DEATH [ ] [ ]

DESCRIBE: ____________________
-8 - NR/DK
-9 - NA

END INTERVIEW

2 - Index Child moved out of last known address to another area in Metro Cebu; and unlocated

3 - Index Child moved out of last known address to an area outside Metro Cebu

X3 WHEREABOUTS OF IC BASED ON HOUSEHOLD INFORMANT:

1 - Outside Metro Cebu, in Cebu province
2 - Outside Cebu province, specify: ____

-7 DK/No available information
-8 - NR
-9 - NA

4 - Index Child refused

5 - Index child institutionalized

6 - Index child incapacitated

-9 - NA

FOR INDEX CHILDREN WHO COULD NOT BE INTERVIEWED:

OBTAIN THE FF. INFORMATION FROM A RELIABLE INFORMANT (COULD BE A HOUSEHOLD MEMBER OR ANY OTHER PERSON WHO MAY HAVE THESE INFORMATION ON THE INDEX CHILD

X4 What is __________________ (NAME OF INDEX CHILD) currently doing?

0 - Not working nor in school
1 - In school
2 - Working
3 - Working and in school
4 - Other, specify ____
-8 - NR/DK

IF IC IS NOT CURRENTLY WORKING, ASK X5

X5 Has __________________ (NAME OF INDEX CHILD) ever worked?

0 - No GO TO X8
1 - Yes
-8 - NR/DK

X6 Has ___________________ (NAME OF INDEX CHILD) worked abroad since tracking survey (for example, as an "Overseas Filipino Worker" or OFW)?

0- No GO TO X8
1- Yes CONTINUE

X7 Where did she work abroad? What kind of jobs did she hold? When did she work abroad?
FILL OUT TABLE X-1 WITH INFORMATION ON JOBS HELD ABROAD. ENTER JOBS IN CHRONOLOGICAL ORDER (STARTING WITH FIRST JOB HELD ABROAD). DATES MAY BE ESTIMATED.

Table X-1. Overseas work experience

Place of job	Type of job	Date started	Date ended (enter -99 if job currently held)
1.			
2.			
3.			
4.			

X8 Marital status of index child

1 - Never married
2 - Legally married
3- Not legally married/cohabiting
4 - Widowed
5 - Separated
-8 - NR/DK

CALL RECORD

Day 1

CALL NO.	DATE	TIME		RESULTS (Use codes below) WRITE BLOCKS COMPLETED	APPOINTMENT MADE	
		Started	Finished		Date	Time

Day 2

CALL NO.	DATE	TIME		RESULTS (Use codes below) WRITE BLOCKS COMPLETED	APPOINTMENT MADE	
		Started	Finished		Date	Time

RESULT CODES:
1 - Interview completed
2 - Interview partly completed, new appointment made
3 - Appointment made for interview later
4 - Refusal, no interview obtained
5 - No respondent at home
6 - Other (SPECIFY)

ID15 Total Number of Sessions Required to Complete Interview:

FOR INDEX CHILDREN WHO WERE INTERVIEWED:

REMIND IC THAT, AS INDICATED IN THE CONSENT FORM, WE WILL BE CALLING HER IN THE FUTURE TO CHECK IF SHE HAS DELIVERED.

ASK IC IF THERE ARE OTHER CONTACT PERSONS WE CAN CALL TO GET IN TOUCH WITH HER IN CASE WE COULD NOT GET HOLD OF HER. CLEARLY EXPLAIN TO THE IC THAT NO QUESTIONS WILL BE ASKED OF THESE CONTACT PERSONS OTHER THAN HOW TO REACH HER.

PERMISSION TO CALL ALTERNATE CONTACT PERSONS GRANTED? ☐

0 - No
1 - Yes

ALTERNATE CONTACT INFORMATION (For questionnaire controller use only; DO NOT ENCODE!!)

1. Name of contact person#1:____

Relationship to IC: ____

Phone numbers: ____

2. Name of contact person#2:____

Relationship to IC: ____

Phone numbers: ____

3. Name of contact person#3:____

Relationship to IC: ____

Phone numbers: ____

BLOCK A: HOUSEHOLD COMPOSITION

A0 Who is the head of this household? ☐

1 - Index child 3- Other household member
2 - Spouse of Index child SPECIFY: ________________________

IF IC IS NOT KNOWLEDGEABLE ENOUGH ABOUT THE HOUSEHOLD, ASK FOR OTHER HOUSEHOLD MEMBERS WHO CAN RESPOND TO SPECIFIC SECTIONS IN THIS BLOCK.

A1 At present, how many persons are living with you in this household?

NO. OF PERSONS ☐☐

A2 How many of the people usually living here with you in this household are temporarily absent? IF NONE, CODE 00.

NO. OF PERSONS ☐☐

FOR ALL PERSONS IN THE HOUSEHOLD, INCLUDING THOSE TEMPORARILY ABSENT, ASK A3 THROUGH A8. RECORD RESPONSES IN TABLE A-1. BEGIN WITH HEAD OF HOUSEHOLD, WHO SHOULD HAVE LINE NUMBER 1, THEN CONTINUE WITH HIS/HER SPOUSE, UNMARRIED CHILDREN, MARRIED CHILDREN, HELPERS, ETC.

A3 What is his/her full name? ENTER NAME IN COL. A3

A4 How is he/she related to the household head? ENTER RESPONSE IN COL. A4
(CODES TO BE ASSIGNED BY EDITORS)

A5 How is he/she related to the index child? ENTER RESPONSE IN COL. A5
(CODES TO BE ASSIGNED BY EDITORS)

A6 IF NOT OBVIOUS TO INTERVIEWER, ASK: Is this person male or female?

1 - Male
2 - Female ENTER CODE IN COL. A6

A7 When was he/she born?

What month? ENTER MONTH IN FIRST COLUMN OF A7 AND

What year? FOUR DIGITS OF YEAR IN 2nd COLUMN OF A7

A8 How old was he/she on his/her last birthday? ENTER AGE IN COLUMN A8

ASK A9 AND A10 OF IC ONLY

A9 What is the highest grade that she completed?
ENTER RESPONSE IN COL. A9

A10 Is she currently studying in school? ENTER CODE IN COL. A10

0 - No
1 - Yes IF YES: What degree/program are you currently enrolled in?

FOR OFFICE EDITOR:
A11 CODE HOUSEHOLD TYPE ☐

TABLE A-1. HOUSEHOLD ROSTER (In case more than 19 persons are found in a household, staple additional HH Form to this one)

Line No.	Name	Relationship to Household Head	Relationship to Index Child	Sex	Date of Birth		Age	ASK OF IC ONLY: Last Grade of School Completed	ASK OF IC ONLY: In School Now? In School Now? What degree/Program?
		Description	Description		Mo.	Yr.			
	A3	A4	A5	A6	A7		A8	A9	A10

A12 Marital status of index child?

1 - Never married
2 - Legally married
3 - Not legally married/cohabiting
4 - Widowed
5 - Separated
-8 - NR/DK

A13 Were there any changes in your marriage since our last visit?

0 - Still never married
1 - Still living with same spouse in last visit
2 - Living with new spouse (legally married)
3 - Living with new spouse (not legally married)
4 - Widowed
5 - Separated
6 - Reunited with previous spouse
7 - Still widowed
8 - Still separated
9 - Others (Specify)____

END OF BLOCK A

BLOCK B: ENVIRONMENTAL INFORMATION

B1 What is your usual source of drinking water? ☐

1 - MCWD piped supply (Metro Cebu Water District)
2 - Other municipal piped supply
3 - Tubewell, borehole, motorized pump with pipes
4 - Dug well fitted with pump
5 - Dug well without pump, bucket used
6 - Spring
7 - River
8 - Rainwater
9 - Mineral water/bottled water
10 - Other (specify) ______________________________

B2 Where is this water source located? ☐

1 - Inside respondent's house
2 - In respondent's yard
3 - Not in house or yard, water delivered by vendor (someone paid to bring water to house)
4 - Not in house or yard, respondent or family member hauls water to house
-8 - NR/DK

B3 What type of toilet facility do you have? ☐

1 - Flush toilet
2 - Water-sealed toilet
3 - Latrine, antipolo
4 - Open pit
(1–4) >CONTINUE
5 - None (use field, canal, seashore) CODE -9 IN B4, GO TO B5
6 - Other (specify) GO TO B5
____
-8 - NR/DK

B4 Where is this located? ☐

1 - Inside respondent's house
2 - Neighbor's house
3 - Outside, private
4 - Outside, public
5 - Other (specify) ______________________________
-8 - NR/DK

B5 What is your usual method of garbage disposal? ☐

1 - Collected by a garbage collector
2 - Burning
3 - Composting
4 - Dumped away from house
5 - Dumped around or near house
6 - Dumped in river/stream
7 - Other (specify) ______________________________
-8 - NR/DK

B6 What type of lighting do you usually use? ☐

1 - Electricity
2 - Kerosene
3 - Oil
4 - LPG (e.g., Gasul, Shellane)
5 - Candle
6 - Other (specify) ______________________________
-8 - NR/DK

B7 What fuel do you usually use for cooking? ☐

1 - Electricity
2 - Kerosene
3 - LPG (e.g., Gasul, Shellane)
4 - Wood/charcoal
5 - Other (specify) ______________________________
-8 - NR/DK

B8 Where do you usually buy most of your food?

1 - Carbon/Taboan Market (main open markets)
2 - Other market in Metro Cebu, specify ____
3 - Supermarket (Name of supermarket: ____)
4 - Sari-sari store (neighborhood grocery stores)
5- Other, specify: ____

-8 - NR/DK

B9 How would you describe the air quality in the neighborhood (street dust, fumes from cars/trucks, burning garbage, fumes from factories, etc.)?

1 - Good (fine, fresh, cool)
2 - Bad smell from human/animal waste
3 - Bad smell from canal/garbage
4 - Presence of street dust/fumes from trucks & other vehicles
5 - Fumes from factories
-8 - NR/DK

ENVIRONMENTAL ASSESSMENT

ALL QUESTIONS FROM B10 TO B24 ARE TO BE ANSWERED BY INTERVIEWER BASED ON THE INTERVIEWER'S OBSERVATIONS OF THE RESPONDENT'S HOUSE OR THE AREA AROUND IT (TEN HOUSES CLOSEST TO THE RESPONDENT'S HOUSE).

B10 INTERVIEWER: OBSERVE, DO NOT ASK. Based on your judgment, how would you describe the air quality in the neighborhood (street dust, fumes from cars/trucks, burning garbage, fumes from factories, etc.)?

1 - Good (fine, fresh, cool)
2 - Bad smell from human/animal waste
3 - Bad smell from canal/garbage
4 - Presence of street dust/fumes from trucks & other vehicles
5 - Fumes from factories
-8 - NR/DK

B11 What is the general condition of the area immediately around the house with respect to excreta removal? Is the house smelling?

1 - Heavy defecation in area
2 - Some defecation in area
3 - Very little excreta visible
4 - No excreta visible

B12 What is the general condition of the neighborhood with respect to excreta removal?

1 - Heavy defecation in area
2 - Some defecation in area
3 - Very little excreta visible
4 - No excreta visible

B13 What is the general condition of the neighborhood with respect to garbage disposal?

1 - Lots of uncollected garbage
2 - Some uncollected garbage
3 - Very little garbage
4 - No garbage visible

B14 Is the area in the house where the food is kept:

1 - Very clean
2 - Not so clean
3 - Filthy

B15 In what type of settlement does the respondent live?

INTERVIEWER: WHEN IN DOUBT, SPECIFY SITIO AND
DO NOT CODE: ____

1 - Urban squatter area
2 - Urban, congested and dirty
3 - Urban, less congested and dirty
4 - Urban, outskirts of city center (e.g., Camputhaw, Lahug, Guadalupe, Banilad)
5 - Rural town (poblacion)
6 - Rural barangay outside of poblacion
7 - Rural - remote (isolated sitio or single house)

B16 What is the area immediately around the respondent's house used for?

1 - Mostly residential houses
2 - Mostly commercial buildings
3 - Mostly open space, used for farming and/or livestock
4 - Mostly open space, not used
5 - Mostly factories/manufacturing/industrial buildings

B17 What is the general area around the respondent's house (within fifty meters) used for?

USE SAME CODES AS IN B16

B18 How many houses are very close (within fifty meters) to the respondent's house?

1 - One
2 - Two
:
20 - Twenty or more

B19 Is the respondent's house connected to the electrical system (Visayan Electric Co. in Cebu City, Mandaue, Lapu-lapu City; some other public system) regardless as to whether it is used or not?

0 - No
1 - Yes

B20 Of what kind of material is the respondent's house constructed?

1 - Light - refers to house made of nipa or similar wood
2 - Mixed - refers to house made of cement and/or wood, but with nipa or similar materials for wall or roof
3 - Strong - refers to house made exclusively of cement and/or wood with galvanized iron roofing

B21 Overall, how would you rate the construction of the houses around the respondent's house?

1 - Mostly light (bamboo, nipa, cheap wood)
2 - Mostly mixed (wood with hollow blocks, cement)
3 - Mostly strong (hollow blocks, concrete, or good wood)

INTERVIEWER: ASSESS THE APPEARANCE OF THE HOUSE, THE CHILDREN, AND THE RESPONDENT (not too obviously!!)

USE TABLE BELOW AND ENTER RESPONSE CODES IN APPROPRIATE COLUMN

1 - Neat and tidy
2 - Not so neat and tidy
3 - Poorly kept, dirty, messy
-9 - NA (No children) For B23 only

B22	House/environs	
B23	Children	
B24	Index Child	

END OF BLOCK B

BLOCK C: HOUSEHOLD ASSETS

INTERVIEWER: IF INDEX CHILD IS LIVING WITH EMPLOYER, QUESTIONS IN THIS BLOCK PERTAIN ONLY TO INDEX CHILD'S AND HER FAMILY'S ASSETS.

C1 Do you/does your household own this house you are living in?

0 - No
1 - Yes
-8 - NR/DK

C2 Do you/does your household own this land on which this house you're living in is built?

0 - No
1 - Yes
-8 - NR/DK

C3 Does your household own any of the following vehicles and appliances?

0 - No
1 - Yes

Bicycle		Rice cooker	
Bicycle with side car		Microwave oven	
Motorcycle/motorbike		Pressure cooker/turbo boiler/blender/other expensive specialized cooking gadgets	
Motorcycle with side car		TV, without cable connection	
Car		TV, with cable connection	
Jeep/jeepney/multicab		VCR (Betamax, VHS, VCD/DVD)	
Truck/bus		Cassette recorder	
Motorized boat		CD player	
Banca/raft		Stereo/Karaoke/Videoke	
Other vehicle, specify ___________________		Computer, without internet access	
Electric iron		Computer, with internet access	
Electric fan		Washing machine	
Air conditioner		Vacuum cleaner/floor polisher	
Sewing machine		Kerosene stove	
Refrigerator		Digital camera/Video camera	
Gas (LPG)/electric stove		Video games (playstation, X-box, etc.)	
Gas (LPG)/electric range/oven		Other appliances, specify ________________	

C4 Does your household have a telephone (landline)?

0 - None
1 - Yes, currently has a phone (PLS MAKE SURE PHONE NUMBER IS RECORDED ON COVER PAGE (ID9)).
2 - No, but has applied for phone connection
-7 - Yes, but currently disconnected

C5 Does any member in your household have a cell phone? If yes, how many members have cell phones (referring to functioning cell phones)?

IF NONE, CODE 0.

END OF BLOCK C

Table D-1. Record of Gainful Activities: Main and Secondary Jobs

LINE NO	NAME OF HOUSEHOLD MEMBER	STATUS	MAIN JOB								SECONDARY JOB								
			Job Description	Code	Status of farm job	Status of non-farm job	Hours worked past week	Bene-fits	TAXES	Go to	Has 2nd job now	Job Description	Code	Status of farm job	Status of non-farm job	Hours worked past week	Bene-fits	TAXES	Go to
D1		D2	D3		D4	D5	D6	D7	D8	D9	D10	D11		D12	D13	D14	D15	D16	D17

BLOCK D. MARKET ACTIVITIES OF RESIDENT HOUSEHOLD MEMBERS

SCREEN FOR INDEX CHILD WHO IS LIVING WITH EMPLOYER. IF SO, ASK BLOCKS D AND E PERTAINING TO IC AND HIS/HER FAMILY MEMBERS LIVING IN THE SAME HOUSEHOLD WITH HIM/HER.

FROM THE HOUSEHOLD ROSTER (TABLE A-1) COPY THE LINE NUMBERS AND NAMES OF RESIDENT HOUSEHOLD MEMBERS WHO ARE 6 YEARS OLD AND ABOVE ON TO TABLE D-1.

UNDER WORK STATUS COLUMN (COL. D2), WRITE "C" FOR THOSE CURRENTLY WORKING AND "P" FOR THOSE NOT CURRENTLY WORKING BUT WORKED IN THE PAST 4 MONTHS. FOR EACH NAME LISTED IN TABLE D-1, ASK D3 THROUGH D16. EXCEPT FOR COLUMNS D3 AND D11, ENTER ONLY CODES IN TABLE D-1. CODE 0 IF NOT CURRENTLY WORKING AND DID NOT WORK IN THE PAST 4 MONTHS

IMMEDIATELY UPON COMPLETION OF TABLE D-1, STAPLE IT TO THE BLANK PAGE OF THIS QUESTIONNAIRE!!!

D3 What is/was his/her main job?

MAIN JOB IS THAT JOB ON WHICH A PERSON SPENDS THE MOST TIME. WRITE DESCRIPTION OF JOB IN D3 BEFORE ENTERING ANY OF CODES SHOWN BELOW. PLACE D3 CODES IN COLUMN PROVIDED.

1 -	Farming	CONTINUE
2 -	Fishing	CODE -9 IN COLUMN D4, THEN GO TO D5
3 -	Other	CODE -9 IN COLUMN D4, THEN GO TO D5
-8 -	NR/DK	
-9 -	NA	

INTERVIEWER: After having entered the codes for question D3 into Table D-1, enter into Col. D9 the **E number** indicated under Questions D4 and D5. This will help you in completing the E Block.

D4 What is/was the nature of his/her job?

1 -	Farm owner	GO TO D6 THROUGH D9 > THEN ASK E11 THROUGH E17 USING <u>TABLE E-3A</u>
2 -	Farm tenant	
3 -	Both owner and tenant	
4 -	Paid farm laborer	GO TO D6 THROUGH D9 > THEN ASK E1 ff., USING <u>TABLE E-1 OR TABLE E-2</u>
5 -	Unpaid family worker on family-owned farm (HAS NO INCOME!)	GO TO D6 THROUGH D9 > THEN ASK E11 ff., USING <u>TABLE E-3A</u>
-8 -	NR/DK	
-9 -	NA	

IF THE FARM IS OWNED BY THE FAMILY/HOUSEHOLD, ONLY ONE MEMBER, USUALLY THE HOUSEHOLD HEAD, CAN WORK AS FARM OWNER. ALL OTHER HOUSEHOLD MEMBERS WORKING ON THE FAMILY FARM ARE EITHER PAID FARM LABORERS OR UNPAID FAMILY WORKERS. IF THE FAMILY/HOUSEHOLD OPERATES THE FARM AS TENANT, THEN ALL FAMILY HOUSEHOLD MEMBERS WORKING ON THAT FARM ARE TENANTS.

ENTER CODE IN COLUMN D4. CODE -9 IN COLUMN D5. GO TO D6.

D5 What is the nature of his/her employment?

1 -	Self-employed	CONTINUE THROUGH D9 IF D3 IS "2" (FISHING), GO TO E25, USE <u>TABLE E-4</u> > IF D3 IS "3" (OTHER), GO TO E29, USE <u>TABLE E-5</u>
2 -	Wage/salary worker	CONTINUE THROUGH D9 > THEN GO TO E1 ff., USING <u>TABLE E-1 OR TABLE E-2</u>
3 -	Unpaid family worker in family-owned business (store, sewing) (HAS NO INCOME)	CONTINUE THROUGH D9 IF D3 IS "2" (FISHING), GO TO E25, USE <u>TABLE E-4</u> > IF D3 IS "3" (OTHER), GO TO E29 USING TABLE E-5
-8 -	NR/DK	
-9 -	NA	

ENTER CODE IN COLUMN D5. CODE IN D4 MUST BE -9. GO TO D6.

D6 During the workweek before this interview, how many hours did he/she spend on this job?

ENTER NUMBER OF HOURS DURING WEEK IN COLUMN D6.

-8 - NR/DK
-9 - NA (did not work last week)

D7 Do/Did you/he/she receive any employment benefits like SSS/GSIS, Philhealth (Medicare), PAG-IBIG in this job?

ENTER CODE IN COLUMN D7

0 - No
1 - Yes
-8 - NR/DK

D8 Do/Did you/he/she pay income or business tax for this job?

ENTER CODE IN COLUMN D8

0 - No
1 - Yes
-8 - NR/DK

D10 Does he/she currently hold a secondary job for which he/she is paid in cash or in kind to supplement his/her income?

A SECONDARY JOB IS A JOB ON WHICH A PERSON SPENDS TIME EITHER (a) AFTER ATTENDING TO HIS/HER MAIN JOB, OR (b) FOR A SHORTER DURATION THAN HIS/HER MAIN JOB, OR A COMBINATION OF BOTH.

0 - No ASK NEXT ELIGIBLE PERSON, BEGIN WITH D3
1 - Yes CONTINUE
-8 - NR/DK ASK NEXT ELIGIBLE PERSON, BEGIN WITH D3
-9 - NA (Not currently working but worked during the last four (4) months)

ENTER CODE IN COLUMN D10 OF TABLE D-1.

D11 What is his/her secondary job?

WRITE DESCRIPTION OF JOB IN D11 BEFORE ENTERING ANY OF CODES SHOWN.
BE PRECISE IN JOB DESCRIPTION. PLACE D11 CODE IN COLUMN PROVIDED.

1 - Farming CONTINUE
2 - Fishing CODE -9 IN COLUMN D12, THEN GO TO D13
3 - Other CODE -9 IN COLUMN D12, THEN GO TO D13
-8 - NR/DK
-9 - NA

INTERVIEWER: After having entered the codes for question D11 into Table D-1, enter into column D17 the **E number** indicated under Questions D12 and D13. This will help you in completing the E Block.

D12 What is the nature of his/her secondary job?

1 - Farm owner
2 - Farm tenant
3 - Both owner and tenant
] GO TO D14 THROUGH D17 THEN ASK E11 THROUGH E17 USING TABLE E3a

4 - Paid farm laborer
] GO TO D14 THROUGH D17 > THEN ASK E1 ff., USING TABLE E-1 OR TABLE E-2

5 - Unpaid family worker on family-owned farm (HAS NO INCOME!)
] GO TO D14 THROUGH D17 > THEN ASK E11 ff., USING TABLE E3a

-8 - NR/DK
-9 - NA

ENTER CODE IN COLUMN D12. CODE -9 IN COLUMN D13. GO TO D14

D13 What is the nature of his/her employment?

1 -	Self-employed	CONTINUE THROUGH D17,<br>IF D11 IS "2" (FISHING),<br>GO TO E25, USE TABLE E-4<br>><br>IF D11 IS "3" (OTHER),<br>GO TO E29, USE TABLE E-5
2 -	Wage/salary worker	CONTINUE THROUGH D17<br>>THEN GO TO E1 ff., USING<br>TABLE E-1 OR TABLE E-2
3 -	Unpaid family worker<br>in family-owned business<br>(store, sewing)<br>(HAS NO INCOME)	CONTINUE THROUGH D17<br>IF D11 IS "2" (FISHING),<br>GO TO E25, USE TABLE E-4<br>><br>IF D11 IS "3" (OTHER),<br>GO TO E29 USING<br>TABLE E-5
-8 -	NR/DK	
-9 -	NA	

ENTER CODE IN COLUMN D13.

D14 During the workweek before this interview, how many hours did he/she spend on this secondary job?

ENTER NUMBER OF HOURS DURING WEEK IN COLUMN D14.

-8 - NR/DK
-9 - NA (Did not work on secondary job last week)

D15 Do/Did you/he/she receive any employment benefits like SSS/GSIS, Philhealth (Medicare), PAG-IBIG in this job?

ENTER CODE IN COLUMN D15

0 - No
1 - Yes
-8 - NR/DK

D16 Do/Did you/he/she pay income/business tax for this job?

ENTER CODE IN COLUMN D16

0 - No
1 - Yes
-8 - NR/DK

GO TO NEXT ELIGIBLE PERSON (D1) OR BLOCK E

END OF BLOCK D

BLOCK E. HOUSEHOLD INCOME

DETERMINE FROM TABLE D-1 WHETHER A PERSON HOLDING TWO JOBS HAS JOBS OF THE SAME OR OF DIFFERENT TYPES.

For this purpose, compare the job status in column D4 (or D5) with that in column D12 (or D13). In case of SELF-EMPLOYMENT AND UNPAID FAMILY WORK, take into account also the codes in columns D3 and D11.

(a) If the two jobs are of the same type, add up the time spent on, and the income earned from, both jobs and enter the cumulative answers in that income table which is indicated.

(b) If the two jobs are of different types, treat each job separately. Determine first the income table into which information for the first (main) job has to be entered, and then the income table for the secondary job.

I. INCOME DERIVED FROM WAGE LABOR

FROM TABLE D-1 (GAINFUL ACTIVITIES), COPY LINE NUMBERS AND NAMES OF ALL RESIDENT HOUSEHOLD MEMBERS WHO ARE OR WERE ENGAGED, OVER THE LAST FOUR MONTHS, IN MARKET ACTIVITIES FOR WHICH THEY RECEIVED A WAGE OR SALARY IN EITHER CASH OR KIND. **WAGE LABORERS** ARE ALL THOSE WITH A CODE OF "4" IN COLUMN D4 AND/OR D12 OR A CODE OF "2" IN COLUMN D5 AND/OR COLUMN D13 IN TABLE D-1. FARM LABORERS AND FISHERMEN HIRED BY OTHERS AND WORKING FOR PAY ARE WAGE LABORERS! IF A PERSON HAS TWO WAGE/SALARY JOBS, ENTER HOURS WORKED AND WAGES RECEIVED FOR BOTH JOBS COMBINED, PROVIDED BOTH JOBS ARE EITHER WAGE-FOR-TIME OR WAGE-FOR-PIECE JOBS. IF TYPES OF JOBS ARE DIFFERENT, ENTER INFORMATION FOR EACH JOB IN APPROPRIATE TABLE. BEFORE ENTERING THE NAMES IN EITHER TABLE E-1 OR TABLE E-2, ASK E1.

E1 Is/Was he/she paid on a TIME basis or on a PER PIECE basis?

IF TIME BASIS, ENTER LINE NUMBER AND NAME IN TABLE E-1 AND ASK E2 TO E5a.
IF ON A PER PIECE BASIS, ENTER LINE NUMBER AND NAME IN TABLE E-2 AND ASK E6 TO E10a.
-8 - NR/DK
-9 - NA

E2 On the average, how many hours does/did he/she work during a usual work day?

ENTER RESPONSE IN COLUMN E2 OF TABLE E-1
-8 - NR/DK
-9 - NA

E3 On the average, how many days does/did he/she work in each week?

ENTER RESPONSE IN COLUMN E3 OF TABLE E-1
-8 - NR/DK
-9 - NA

E4 How much (IN PESOS) does/did he/she normally receive for a usual day's work, including allowances?

ENTER RESPONSE IN COLUMN E4 OF TABLE E-1
-8 - NR/DK
-9 - NA

E5 On the average, how many weeks does/did he/she work each year?

ENTER RESPONSE IN COLUMN E5 OF TABLE E-1
-8 - NR/DK
-9 - NA

E5a Is/Was the job in Table E-1 regular?

ENTER RESPONSE IN COLUMN E5a OF TABLE E-1
1 - Yes
2 - No, seasonal
3 - No, recently employed (last 3 months)
4 - No, not currently working but worked in past 4 months
-8 - NR/DK
-9 - NA

Table E-1. Income Derived from Wage Labor on a Time Basis

LINE NO.	NAME	AVE. NO. HRS. WORKED PER DAY	AVE. NO. DAYS PER WK. OF WORK	PESOS RECEIVED PER DAY'S WORK	AVE. NO. OF WKS. WORKED PER YEAR	STATUS OF JOB
	E1	E2	E3	E4	E5	E5a

IF PERSON HAS A SECONDARY JOB WHICH IS DIFFERENT FROM THE MAIN JOB, GO TO APPROPRIATE QUESTIONS. IF PERSON HAS ONLY ONE JOB, GO TO NEXT PERSON OR, AFTER LAST PERSON, GO TO E18.

E6 Usually, how many pieces of (CLOTHES, NECKLACES, BRACELETS, LAUNDRY, ETC.) does/did he/she finish in a day?

ENTER RESPONSE IN COLUMN E6 OF TABLE E-2
-8 - NR/DK -9 - NA

E7 How much (IN PESOS) is/was he/she paid per finished item?
ENTER RESPONSE IN COLUMN E7 OF TABLE E-2
-8 - NR/DK -9 - NA

E8 On the average, how many hours per day does/did he/she engage in piece labor?

ENTER RESPONSE IN COLUMN E8 OF TABLE E-2
-8 - NR/DK -9 - NA

E9 On the average, how many days per week does/did he/she engage in piece labor?

ENTER RESPONSE IN COLUMN E9 OF TABLE E-2
-8 - NR/DK -9 - NA

E10 On the average, how many weeks per year does/did he/she engage in piece labor?
ENTER RESPONSE IN COLUMN E10 OF TABLE E-2
-8 - NR/DK -9 - NA

E10a Is/Was the job in Table E-2 regular?
ENTER RESPONSE IN COLUMN E10a OF TABLE E-2
1 - Yes 4 - No, not currently working but worked in past 4 months
2 - No, seasonal -8 - NR/DK
3 - No, recently employed (last 3 months) -9 - NA

Table E-2. Income Derived from Labor Paid Per Piece

LINE NO.	NAME	AVE. NO. OF ITEMS FINISHED PER DAY	PESOS RECEIVED PER ITEM	AVE. NO. OF HRS. PER DAY	AVE. NO. OF DAYS WORKED PER WK.	AVE. NO. OF WKS WORKED PER YR.	STATUS OF JOB
		E6	E7	E8	E9	E10	E10a

IF PERSON HAS A SECONDARY JOB WHICH IS DIFFERENT FROM THE MAIN JOB, GO TO APPROPRIATE QUESTIONS. IF PERSON HAS ONLY ONE JOB, GO TO NEXT PERSON, OR, AFTER LAST PERSON, TO E18.

II. INCOME DERIVED FROM FARMING ACTIVITIES (EXCLUDING INCOME FROM LIVESTOCK)

FROM TABLE D-1, COPY INTO TABLE E-3A THE LINE NUMBERS AND NAMES OF ALL HOUSEHOLD MEMBERS WHO HAVE HAD FARM JOBS. THESE ARE ALL THOSE PERSONS WITH A CODE OF "1" IN COLUMN D3 AND/OR D11 AND WITH CODES "1" , "2" , "3" , OR "5" IN COLUMN D4 AND/OR D12. FOR ALL OF THESE PERSONS, ASK QUESTIONS E11 - E13a.

E11 On the average, how many hours per day does/did he/she work on the farm?
ENTER NUMBER OF HOURS PER DAY IN COLUMN E11 OF TABLE E-3A
-8 - NR/DK -9 - NA

E12 On the average, how many days per week does/did he/she work on the farm?
ENTER NUMBER OF DAYS PER WEEK IN COLUMN E12 OF TABLE E-3A
-8 - NR/DK -9 - NA

E13 On the average, how many weeks per year does/did he/she work on the farm?
ENTER NUMBER OF WEEKS PER YEAR IN COLUMN E13 OF TABLE E-3A

E13a Is/Was the job in Table E-3A regular?
ENTER RESPONSE IN COLUMN E13a OF TABLE E-3A

1 - Yes
2 - No, seasonal
3 - No, recently farming (last 3 months)
4 - No, not currently farming but worked in past 4 months
-8 - NR/DK
-9 - NA

IF THE FAMILY OPERATES THE FARM AS A FAMILY FARM, OR AS TENANTS, ASK QUESTIONS E14 THROUGH E17 ONLY OF THE HEAD OR THE ELDEST MEMBER OF THAT FAMILY. IF A FAMILY OR HOUSEHOLD MEMBER CULTIVATES ANOTHER FARM INDEPENDENTLY OF THE FAMILY, ASK ALL QUESTIONS OF THIS MEMBER.

E14 What were the major crops that he/she planted in the past 12 months?
USE ONE LINE FOR EACH MAJOR CROP
-8 - NR/DK -9 - NA

ENTER RESPONSE IN COLUMN E14 OF TABLE E-3A

E15 On the average, how much did he/she spend in the past 12 months for seedlings, fertilizer, tools, hired labor, insecticides, etc.?
ENTER RESPONSE, IN PESOS, IN COLUMN E15 OF TABLE E-3A
-8 - NR/DK -9 - NA

E16 How much did he/she receive for the portion of the produce that was sold?
ENTER RESPONSE, IN PESOS, IN COLUMN E16 OF TABLE E-3A
-8 - NR/DK -9 - NA

E17 If he/she sold that portion of the harvest that he/she set aside for home consumption, how much would he/she have received?
ENTER RESPONSE, IN PESOS, IN COLUMN E17 OF TABLE E-3A
-8 - NR/DK -9 - NA

Table E-3A. Income Derived from Farming, Excluding Livestock

LINE NO.	NAME	WORKING TIME				MAJOR CROPS	EXPEN-SES (PESOS)	MONEY VALUE OF CROP	
		HRS. PER DAY	DAYS PER WEEK	WKS. PER YEAR	STAT. OF JOB			SOLD	HOME USE
		E11	E12	E13	E13a	E14	E15	E16	E17

III. INCOME DERIVED FROM LIVESTOCK RAISING

QUESTION E18 AND, IF APPLICABLE, ALL OTHER QUESTIONS RELATED TO LIVESTOCK RAISING, ARE TO BE ASKED OF ALL HOUSEHOLDS REGARDLESS OF WHETHER THEY OPERATE A FARM OR NOT.

E18 Is there a member of your household who has been engaged in raising livestock (carabao, cow, goat, duck, chicken, and others) in the past 12 months?
ASK FOR NAME OF HOUSEHOLD MEMBER AND ENTER IT IN TABLE E-3B TOGETHER WITH HER LINE NUMBER SHOWN IN TABLE A-1. ☐

0 -	No	GO TO E25	-8 -	NR/DK	GO TO E25
1 -	Yes	CONTINUE	-9 -	NA	GO TO E25

ASK QUESTIONS E19-E21 OF ALL PERSONS LISTED IN TABLE E-3B

E19 On an average working day, how many hours does/did he/she work with the livestock?
ENTER NUMBER OF HOURS PER DAY IN COLUMN E19 OF TABLE E-3B
-8 - NR/DK -9 - NA

E20 On an average work week, how many days does/did he/she work with the livestock?
ENTER NUMBER OF DAYS PER WEEK IN COLUMN E20 OF TABLE E-3B
-8 - NR/DK -9 - NA

E21 On the average, how many weeks in the past 12 months does/did he/she work with the livestock?
ENTER NUMBER OF WEEKS PER YEAR IN COLUMN E21 OF TABLE E-3B
-8 - NR/DK -9 - NA

E21a Is/Was the livestock raising regular?
ENTER RESPONSE IN COLUMN E21a OF TABLE E-3B

1 -	Yes	4 -	No, not currently raising but worked in past 4 months
2 -	No, seasonal	-8 -	NR/DK
3 -	No, recently raising (last 3 months)	-9 -	NA (Livestock raised by non-hhold member/hired)

IF THE LIVESTOCK RAISING IS A FAMILY OPERATION, ASK QUESTIONS E22 THROUGH E24 ONLY OF THE HEAD OR THE ELDEST MEMBER OF THE FAMILY. IF ANY FAMILY OR HOUSEHOLD MEMBER RAISES LIVESTOCK ELSEWHERE INDEPENDENTLY OF THE FAMILY OR HOUSEHOLD, ASK ALL QUESTIONS OF THIS HOUSEHOLD MEMBER.

E22 How much do you think did he/she spend in raising livestock for the past 12 months (for purchasing, feeding, treating, etc.)?
ENTER RESPONSE IN PESOS IN COLUMN E22 OF TABLE E-3B
-8 - NR/DK -9 - NA

E23 What was his/her total income from the sale of any livestock or livestock products in the past 12 months?
ENTER RESPONSE, IN PESOS, IN COLUMN E23 OF TABLE E-3B
-8 - NR/DK -9 - NA

E24 If he/she sold the livestock or livestock products which he/she set aside for consumption in the past 12 months, how much do you think he/she would have received?
ENTER RESPONSE, IN PESOS, IN COLUMN E24 OF TABLE E-3B
-8 - NR/DK -9 - NA

Table E-3B. Income Derived from Raising Livestock

LINE NO.	NAME	WORKING TIME				EXPENSES (PESOS)	MONEY VALUE OF LIVESTOCK	
		HRS. PER DAY	DAYS PER WEEK	WKS. PER YEAR	STATUS OF JOB		SOLD	HOME USE
		E19	E20	E21	E21a	E22	E23	E24

IV. INCOME DERIVED FROM FISHING ACTIVITIES

FROM TABLE D-1, COPY LINE NUMBERS AND NAMES OF ALL RESIDENT HOUSEHOLD MEMBERS WHO EITHER ARE OR WERE ENGAGED IN FISHING OVER THE PAST FOUR MONTHS. THESE ARE ALL THOSE PERSONS WITH A CODE OF "2" IN COLUMN D3 AND/OR COLUMN D11 AND A CODE OF "1" OR "3" IN COLUMN D5 AND/OR COLUMN D13. FISHERS HIRED BY OTHERS AND WORKING FOR PAY ARE WAGE WORKERS. IF FISHING IS A FAMILY/HOUSEHOLD ACTIVITY, ASK E25 THROUGH E27a OF ALL MEMBERS LISTED IN TABLE E-4, BUT ASK QUESTION E28 OF ONLY ONE MEMBER, THE ONE IN CHARGE OF THE FAMILY/HOUSEHOLD FISHING OPERATIONS.

E25 On an average working day, how many hours a day does/did he/she go fishing?

ENTER NUMBER OF HOURS PER DAY IN COLUMN E25 OF TABLE E-4
-8 - NR/DK
-9 - NA

E26 On the average, how many days per week does/did he/she usually go fishing?

ENTER RESPONSE IN COLUMN E26 OF TABLE E-4
-8 - NR/DK
-9 - NA

E27 On the average, how many weeks in a year does/did he/she usually go fishing?

ENTER RESPONSE IN COLUMN E27 OF TABLE E-4
-8 - NR/DK
-9 - NA

E27a Is/Was the job in Table E-4 regular?

ENTER RESPONSE IN COLUMN E27a OF TABLE E-4
1 - Yes
2 - No, seasonal
3 - No, recently fishing (last 3 months)
4 - No, not currently fishing but worked in past 4 months
-8 - NR/DK
-9 - NA

E28 How much is/was his/her usual net income out of a day's catch, including that portion of the catch which the household itself consumed? (EXCLUDING EXPENSES)

ENTER RESPONSE IN COLUMN E28 OF TABLE E-4
-7 - unpaid work
-8 - NR/DK
-9 - NA

Table E-4. Income Derived from Fishing Activities

LINE NO.	NAME	WORKING TIME				USUAL DAILY NET INCOME (IN PESOS)
		HRS. PER DAY	DAYS PER WK.	WEEKS PER YR.	STATUS OF JOB	
		E25	E26	E27	E27a	E28

V. INCOME DERIVED FROM SELF-EMPLOYMENT

FROM TABLE D-1, COPY LINE NUMBERS AND NAMES OF ALL HOUSEHOLD MEMBERS WHO ARE OR WERE SELF-EMPLOYED OR WORKING AS UNPAID FAMILY WORKERS IN A FAMILY-OWNED BUSINESS DURING THE PAST FOUR MONTHS. THESE ARE ALL PERSONS WITH A CODE OF "1" OR "3" IN D5 AND/OR COLUMN D13. E.G., IF A FAMILY/HOUSEHOLD OPERATES A STORE IN WHICH SOME MEMBERS WORK AS UNPAID FAMILY WORKERS, ASK E29 THROUGH E34a OF ALL MEMBERS WORKING IN THE STORE, BUT E35 ONLY OF THE MEMBER IN CHARGE OF THE STORE.

E29 Does he/she work in a family-owned business (e.g., sari-sari store, sewing business)?
ENTER RESPONSE IN COLUMN E29 OF TABLE E-5
0 - No GO TO E32 -8 - NR/DK
1 - Yes CONTINUE -9 - NA

E30 What is this business? (sari-sari store, sewing, barber, etc.)
SPECIFY (DO NOT CODE). ENTER RESPONSE IN COL. E30 OF TABLE E-5
-8 - NR/DK -9 - NA

E31 Is the enterprise located at home?
ENTER RESPONSE IN COLUMN E31 OF TABLE E-5
0 - No -8 - NR/DK
1 - Yes -9 - NA

E32 How many hours does/did he/she work in a usual day?
ENTER RESPONSE IN COLUMN E32 OF TABLE E-5
-8 - NR/DK -9 - NA

E33 How many days in a week does/did he/she usually work?
ENTER RESPONSE IN COLUMN E33 OF TABLE E-5
-8 - NR/DK -9 - NA

E34 How many weeks in a year does/did he/she usually work?
ENTER RESPONSE IN COLUMN E34 OF TABLE E-5
-8 - NR/DK -9 - NA

E34a Is/was the business regular?
ENTER RESPONSE IN COLUMN E34a OF TABLE E-5
1 - Yes 4 - No, not currently working but worked in past 4 months
2 - No, seasonal -8 - NR/DK
3 - No, recently employed (last 3 months) -9 - NA

E35 How much on the average is/was his/her daily net income? (EXCLUDE EXPENSES)
ENTER RESPONSE IN COLUMN E35 IN TABLE E-5
-7 - Unpaid work -9 - NA
-8 - NR/DK

GO TO NEXT PERSON LISTED OR, IF LIST IS COMPLETED, TO E36

Table E-5. Income Derived from Self-Employment

LINE NO	NAME	WORK IN FAM. BUS.	TYPE OF BUSINESS	BUS. AT HOME	AVE. NO. HRS/ DAY	AVE. NO. DAYS/ WEEK	AVE. WKS/ YEAR	STAT. OF JOB	AVE. DAILY NET INC. (IN PESOS)
		E29	E30	E31	E32	E33	E34	E34a	E35

VI. HOUSEHOLD INCOME DERIVED FROM OTHER SOURCES

E36 Does/did your household or any of its members have other sources of income? ☐

0 -	No	CODE -9 IN E37 AND GO TO E38
1 -	Yes	CONTINUE
-8 -	NR/DK	CODE -8 IN E37 AND GO TO E38

E37 What are these sources of income? How much was received from each source in the past12 months?
IF NONE, CODE 00

Rent from agricultural/commercial land ☐☐☐☐☐

Income from boarders/lodgers, house rental ☐☐☐☐☐

Pensions, dividends, bonuses, savings interest ☐☐☐☐☐

Cash remittances from children, parents, other relatives, friends or anyone else ☐☐☐☐☐

Cash remittances from spouse abroad/sustento ☐☐☐☐☐

Loans, donations ☐☐☐☐☐

Winnings (masiao, lotto, sabong) ☐☐☐☐☐

Income from home gardening ☐☐☐☐☐

Others, specify ____ ☐☐☐☐☐

E38 Did you or your household receive any income in kind (food or clothing) from children, parents, relatives, friends or anyone in the past 12 months? ☐

0 -	No	CODE -9 IN E39, GO TO BLOCK F
1 -	Yes	CONTINUE
-8 -	NR/DK	CODE -8 IN E39, GO TO BLOCK F

E39 What was the approximate value (IN PESOS) of this income in kind in the past 12 months?

-8 -	NR/DK	PESOS ☐☐☐☐
-9 -	NA	

END OF BLOCK E

BLOCK F. FERTILITY, MARRIAGE, AND COHABITATION

CURRENT PREGNANCY

RECORD ALL PERTINENT INFORMATION /ANSWERS IN TABLE F-1.

F1 What was the result of this pregnancy?

6 - Currently pregnant
-7 - Not sure if currently pregnant

F3 How many months or weeks were you pregnant?

GIVE NUMBER OF MONTHS OR WEEKS PREGNANT.

IF NOT SURE IF PREGNANT, GIVE NUMBER OF WEEKS OR DAYS SINCE LAST MENTRUATION (START FROM THE FIRST DAY OF LAST CYCLE) THEN GO TO F50.

F4 Did you get prenatal care during this pregnancy?

0 - No GO TO INSTRUCTION BEFORE F12
1 - Yes CONTINUE

F5 When (in what month of your pregnancy) did you first get prenatal care? (Month, Day, Year or in what month of pregnancy)

-9 - NA

F6 Where did you first get prenatal care for this pregnancy?

1 - Traditional birth attendant
2 - Government personnel/facility
3 - Private personnel/facility
-9 - NA

F7 How many times during this pregnancy did you get prenatal care from _______(CATEGORIES IN F6)?

0 - None (for this category)
1 - Once
-9 - NA (Did not get any prenatal care)

F8 During your prenatal care, have you been told you had any problems with this pregnancy?

0 - No problem
1 - Gestational Diabetes
2 - High Blood Pressure
3 - Placenta Previa
4 - Small pelvis
5 - Other (specify): __________

F9 Did you take any supplements/vitamins during this pregnancy? If yes, what kind?

MULTIPLE ANSWERS ALLOWED

0 - No
1 - Yes, (specify)
IF YES: Probe on source of vitamins

F10 Were you given an injection during this pregnancy? If yes, what kind?
MULTIPLE ANSWERS ALLOWED

0 - No injection GO TO INSTRUCTION BEFORE F12
1 - 1st injection (specify)
2 - 2nd injection (specify)
-9 - NA

F11 Who gave you this injection?

1st injection __________
2nd injection __________
-9 - NA

F12 Where are you planning on giving birth?

1 - At home
2 - Public hospital/health center (specify)
3 - Private hospital/private clinic (specify)
4 - Others (specify)
-9 - NA

F13 Who will assist you in delivering the child?

1 - Doctor
2 - Midwife
3 - Traditional birth attendant
4 - Others (specify)
5 - Combination (specify)
-9 - NA

Table F-1. Current Pregnancy

QUEST. NO.	PREG. ORDER	FIRST PREGNANCY	SECOND PREGNANCY	THIRD PREGNANCY	FOURTH PREGNANCY	FIFTH PREGNANCY	SIXTH PREGNANCY
	NAME						
**F1** Type of pregnancy termination							
**F3** Pregnancy duration							
**F4** With prenatal care? IF NO, GO TO INSTRUCTION BEFORE F12							
**F5** Date of first prenatal care							
**F6** Source of first prenatal care							
**F7** Frequency of prenatal care from: 1. TBA 2. Govt. 3. Pvt.							
**F8** During your prenatal care, have you been told you had any problems with this pregnancy							
**F9** Supplements or vitamins taken and type							
**F10** Injections received and type							
**F11** Type of personnel who gave injection<br>1st injection<br>2nd injection							
**F12** Place where to give birth							
**F13** Type of personnel who will assist in delivery							

F37 Are you planning on breastfeeding?

0 - No, why not planning breastfeeding
1 - Yes, why planning on breastfeeding
-9 - NA

F41 Who is the father of this child/pregnancy?

WRITE NUMBER OF FATHER (FATHER #1, FATHER #2, etc.)
-9 - NA

F42 Were you cohabiting with or married to the father at the time of this pregnancy?

0 - No
1 - Yes, cohabiting
2 - Yes, legally married
-9 - NA

F43 Before this pregnancy (or between this pregnancy and the previous one), what FP method were you using for the longest duration?

0 - None GO TO F44
1 - Pill
2 - IUD
3 - Injection (DEPO PROVERA)
4 - Implant (NORPLANT)
5 - Diaphragm
6 - Foam, Jelly (SAMPOON)
7 - Condom
8 - Ligation
9 - Vasectomy
10 - Rhythm, Calendar Method
11 - Rhythm, Temperature, Symptoms
12 - Withdrawal
13 - Breastfeeding
14 - Abstinence
15 - Other, specify ___________________
-9 - NA

IF FP WAS USED SKIP TO F45

F44 Can you tell us the reason for not using family planning at that time?

0- Wanted to get pregnant
1- Partner disapproved
2- Concern re: side effects
2- Health concerns
3- Difficult to obtain
4- Costs too much
5- Inconvenient to use
6- Other, specify ___________________
-9 - NA

F45 Did you work during this pregnancy? If yes, what kind of work?

0 - No
1 - Yes (specify kind of work/occupation)
-9 - NA

F47 At the time you were pregnant, did you want to become pregnant then, did you want to wait until later, or did you want no (no more) children at all?

1 - Then
2 - Later
3 - No/No more

F48 How did you react to the pregnancy?
PROBE FOR INTENTION TO ABORT

(VERBATIM)

F49 How did your spouse/partner react to the pregnancy?
PROBE FOR INTENTION TO ABORT

(VERBATIM)

Table F-1. Current Pregnancy (continued)

QUEST. NO.	PREG. ORDER	FIRST PREGNANCY	SECOND PREGNANCY	THIRD PREGNANCY	FOURTH PREGNANCY	FIFTH PREGNANCY	SIXTH PREGNANCY
	NAME						
**F37** Are you planning on breastfeeding? Why?							
**F41** Who is the father of this child/pregnancy? WRITE NUMBER OF FATHER (FATHER #1, FATHER #2, etc.)							
**F42** Cohabiting at the time of pregnancy?							
**F43** FP method used for longest duration before or between the pregnancy							
**F44** IF NO FP used, ask reason for not using							
**F45** Work during this pregnancy? If yes, what kind of work?							
**F47** At the time you were pregnant, did you want to become pregnant then, did you want to wait until later, or did you want no (no more) children at all?<br>1 - Then<br>2 - Later<br>3 - No/No more							
**F48** How did you react to the pregnancy? PROBE FOR INTENTION TO ABORT (VERBATIM)							
**F49** How did your spouse/partner react to the pregnancy? PROBE FOR INTENTION TO ABORT (VERBATIM)							

F50 Did you have sexual relations in the past week?

0 - No GO TO F52
1 - Yes CONTINUE
-8 - NR/DK GO TO F52
-9 - NA

F51 How many days last week did you have sexual relations?

1 - One day
:
-8 - NR/DK
-9 - NA (None during last week)

MARRIAGE/COHABITATION

INTERVIEWER: MARITAL HISTORY IS AN UPDATE OF ALL MARRIAGES IC HAD SINCE TRACKING SURVEY.

F52 How many times have you been married/cohabited/lived-in?

0 - Never GO TO BLOCK I
1 - Once
:
-8 - NR/DK
-9 - NA

FOR EACH MARRIAGE/COHABITATION, ASK QUESTIONS F53 TO F59. ENTER RESPONSES IN APPROPRIATE COLUMNS IN TABLE F-2

F53 When did this marriage/cohabitation happen?
ENTER RESPONSE IN MONTH AND YEAR

-8 - NR/DK
-9- NA

F54 Was the marriage done in church, by a judge or consensual union? Or, were you married in church, civil or agreed to cohabit?

1 - Church
2 - Civil
3 - Consensual union
4 - Civil first then church
5 - Consensual union, church
6 - Consensual union, civil
7 - Consensual union, civil, church
-9 - NA

F55 Did you elope before you got married/cohabited?

0 - No
1 - Yes
-9 - NA

F56 How long was the marriage/cohabitation? ENTER RESPONSE IN NUMBER OF MONTHS

-8 - NR/DK
-9- NA

F57 What was the reason for the termination of marriage/cohabitation?

0 - Still living together
1 - Death of partner
2 - Separation
3 - Desertion by respondent
4 - Desertion by partner
-8- NR/DK
-9 - NA

F58 How many pregnancies did you have during this marriage/cohabitation (including current pregnancy)?

-8 - NR/DK
-9- NA

F59 What family planning method(s) did you use during this marriage/cohabitation?

Table F-2. Marital History of IC

Marriage/ Cohabitation	F53. Date	F54. Type	F55. Eloped?	F56. Duration	F57. Reason for termination	F58. Total no. of pregnancies	F59. FP method (s) used
First							
Second							
Third							
Fourth							
Fifth							
Sixth							
Seventh							

END OF BLOCK F

BLOCK I. PHYSICAL ACTIVITY

IF IC IS CURRENTLY WORKING, GO TO I1
OTHERWISE GO TO I5

I1 In what physical position do you usually carry out your main job?

1 - Sitting most of the time
2 - Standing most of the time
3 - Squatting on the ground most of the time (e.g. doing laundry, weeding the farm)
4 - Standing and bending over most of the time (e.g. planting rice)
5 - Moving around most of the time
6 - Combination of any codes above (specify codes) ____________________________
-9 - NA

I2 Does your main job require physical exertion (e.g., lifting, pushing objects, etc.)? IF YES, what kind?

0 - No
1 - Yes (specify) ____
-9 - NA

IF IC HAS A SECONDARY JOB, GO TO I3
OTHERWISE GO TO I5

I3 In what physical position do you usually carry out your secondary job?

1 - Sitting most of the time
2 - Standing most of the time
3 - Squatting on the ground most of the time (e.g. doing laundry, weeding the farm)
4 - Standing and bending over most of the time (e.g. planting rice)
5 - Moving around most of the time
6 - Combination of any codes above (specify codes) ____________________________
-9 - NA

I4 Does your secondary job require physical exertion (e.g., lifting, pushing objects, etc.)? IF YES, what kind?

0 - No
1 - Yes (specify) ____
-9 - NA

I5 ENTER ANSWERS TO THE FOLLOWING 10 QUESTIONS IN TABLE I-1 ON THE OPPOSITE PAGE

a) What time do you usually wake up?

b) What time do you usually eat breakfast (Or, first eating time after waking up)?

c) What time do you usually eat lunch (Or, second eating time after waking up)?

d) What time do you usually eat supper (Or, third eating time after waking up)?

e) What time do you usually go to bed?

f) From the time you wake up until your breakfast/1st eating time, what are your usual activities?

g) From breakfast/1st eating time to lunchtime/2nd eating time, what are your usual activities?

h) From lunchtime/2nd eating time to suppertime/3rd eating time, what are your usual activities?

i) From suppertime/3rd eating time until the time you sleep, what are your usual activities?

j) Please tell me how long each activity you've mentioned would take (in minutes)?

INTERVIEWER: BE SURE TO HAVE THE IC INCLUDE RESTING, TALKING WITH FRIENDS OR NEIGHBORS, TRAVEL TIME TO SCHOOL/WORK AS WELL AS USUAL HOUSEHOLD TASKS AND USUAL WORK OUTSIDE OF THE HOME. BE SURE TO INCLUDE PHYSICAL ACTIVITIES LIKE SPORTS, ETC. NOTE ACTIVITIES THAT ARE DONE SIMULTANEOUSLY, SUCH AS IRONING AND WATCHING THE CHILDREN. AFTER THE ACTIVITY ITSELF, ASK ABOUT THE APPROXIMATE TIME (TO BE RECORDED IN MINUTES) THAT IC SPENDS ON A GIVEN TASK. IF THIS IS MORE THAN THE MORNING OR AFTERNOON OR EVENING, PROBE. THE REPORTING OF RESTING AND SLEEPING AS ACTIVITIES IS O.K.! WE DO NOT WANT IC TO REPORT LOTS OF ACTIVITIES IN ORDER NOT TO APPEAR LAZY. WHENEVER YOU FEEL THAT IC HAS REPORTED TOO MANY ACTIVITIES BUT NO PERIODS OF REST, PROBE.

Table I-1. Daily Activities on a Working Day

PART OF DAY	ACTIVITY (I5f, g, h, i)	TIME SPENT I5j)
I5a)<br><br>Waking-up Time:<br><br>-------------------	1	
	2	
	3	
	4	
	5	
	6	
I5b)<br><br>Breakfast Time: (1st eating time)<br><br>-------------------	1	
	2	
	3	
	4	
	5	
	6	
I5c)<br><br>Lunch Time: (2nd eating time)<br><br>-------------------	1	
	2	
	3	
	4	
	5	
	6	
	7	
	8	
	9	
	10	
I5d)<br><br>Supper Time: (3rd eating time)<br><br>-------------------	1	
	2	
	3	
	4	
	5	
	6	
	7	
	8	
	9	
	10	
I5e)<br>Bed Time:<br><br>-------------------		

I6 Now I would like to ask about your usual activities on a non-working/non-school day (e.g., weekend for those who are working/studying). Let's please break down the day from the time you wake up to the time you sleep.

ASK SAME QUESTIONS AS I5a) to I5j) AND ENTER ANSWERS IN TABLE I-2 BELOW

Table I-2. Daily Activities on a Non-working/Non-school Day

PART OF DAY	ACTIVITY (I6f, g, h, i)	TIME SPENT I6j)
I6a) Waking-up Time: -------------------	1	
	2	
	3	
	4	
	5	
	6	
I6b) Breakfast Time: (1st eating time) -------------------	1	
	2	
	3	
	4	
	5	
	6	
I6c) Lunch Time: (2nd eating time) -------------------	1	
	2	
	3	
	4	
	5	
	6	
	7	
	8	
	9	
	10	
I6d) Supper Time: (3rd eating time) -------------------	1	
	2	
	3	
	4	
	5	
	6	
	7	
	8	
	9	
	10	
I6e) Bed Time: -------------------		

END OF BLOCK I

BLOCK J: IC'S DIET - DAY ONE

INTERVIEWER: (a) "Usual" means at least 3 to 4 times weekly.
(b) Food preparation or eating "at home" includes any home setting or an extension of home, e.g., relative's home.
(c) Food provided at place of work is considered bought.
(d) For IC who sells food and eats what they sell, the food is bought.

J1 How many meals, excluding 'painit'/snacks, do you usually eat in a day?

CODE NUMBER OF USUAL DAILY MEALS ☐☐

J2 How many times do you usually eat 'painit'/snacks in a day?

CODE NUMBER OF USUAL DAILY SNACKS ☐☐

J3 How many times do you eat at home, excluding 'painit'/snacks in a week?

CODE NUMBER OF WEEKLY MEALS PREPARED AT HOME AND EATEN AT HOME ☐☐

J4 Excluding 'painit'/snacks, how many times do you buy ready-cooked food in a week?

CODE NUMBER OF WEEKLY MEALS PURCHASED OUTSIDE AND EATEN BY IC ☐☐

IF ONLY COOKED VIANDS ARE BOUGHT OUTSIDE BUT NOT OTHER ITEMS OF THE MEAL, CONSIDER THE ENTIRE MEAL AS BOUGHT OUTSIDE!

J5 How many times do you or your household buy 'painit'/snacks in a week? ☐☐

CODE NUMBER OF TIMES

J6 Where do you usually eat snacks and lunch?

1 -	In school	CONTINUE	'Painit'/snacks	☐☐
2 -	At workplace	CONTINUE		
3 -	At home	GO TO J8		
4 -	Elsewhere	GO TO J8		
	Specify: ____________________		Lunch	☐☐
-8 -	NR/DK			

J7 If you eat in school or at workplace, where is the food prepared?

1 -	Bought in or around school/at workplace	'Painit'/Snacks	☐☐
2 -	Brought from home	Lunch	☐☐
3 -	Provided free by school/workplace		
-8 -	NR/DK		
-9 -	NA		

J8 In a month, how many times do you eat outside the home: RECORD NUMBER OF TIMES

1 - In a formal/expensive restaurant ☐☐

2 - In a fastfood place (food courts, Jollibee, etc.) ☐☐

IF RESPONSE IS NOT EQUAL TO "0", ASK:
What is/are the name(s) of the fastfood place(s) where you usually go to eat? ☐☐

____ ☐☐

____

____

3 - In a cafeteria/canteen (incl. workplace canteens) ☐☐

4 - In a carenderia/turo-turo (cheaper eating place) ☐☐

INTERVIEWER: DO NOT CODE QUESTIONS J9 THROUGH J12

J9 What do you usually eat and drink for breakfast?

LIST GENERAL CATEGORIES OF FOOD SUCH AS dried fish, pork, rice, noodles, camote, banana, etc.

FOOD/DRINK 1	2	3	4	5
☐	☐	☐	☐	☐

__________, __________, __________, __________, __________

J10 What do you usually eat and drink for lunch?

FOOD/DRINK 1 2 3 4 5

LIST GENERAL CATEGORIES OF FOOD SUCH AS dried fish, pork, rice, noodles, camote, banana, etc.

__________, __________, __________, __________, __________

J11 What do you usually eat and drink for supper?

FOOD/DRINK 1 2 3 4 5

LIST GENERAL CATEGORIES OF FOOD SUCH AS dried fish, pork, rice, noodles, camote, banana, etc.

__________, __________, __________, __________, __________

J12 What do you usually eat and drink for painit/snacks?

FOOD/DRINK 1 2 3 4 5

LIST GENERAL TYPES OF SNACKS SUCH AS bread, puto, biko, bodbod, etc.

__________, __________, __________, __________, __________

J13 Do you regularly take vitamin or mineral supplements?

0 -	No	GO TO J15	EXCLUDE PRE- AND
1 -	Yes	CONTINUE	POSTNATAL VITAMINS
-8 -	NR/DK	GO TOJ15	AND MINERALS!!

J14 What kind? SPECIFY: __________

-8 - NR/DK
-9 - NA

J15 What kind of oil do you usually use for cooking?

0 - No cooking oil used in household
1 - Coconut oil ('edible', 'tinakus')
2 - Lard
3 - Others (corn oil, etc.)
4 - Combination of 1 to 3
5 - All meals bought
-8 - NR/DK

J16 How much oil do you usually use in a week?

SPECIFY QUANTITY __________

IN TERMS OF: lapad, tumbok, longneck, gallon, etc.
-8 - NR/DK (For households preparing food for business and consumption)
-9 - NA (No cooking oil used)

INTERVIEWER: DO NOT CODE! OFFICE: CONVERT INTO MILLILITER

IC'S 24-HR FOOD RECALL
Date of interview: MONTH/DAY/YEAR __________

J17 Food Recall:

1 - Day 1
2 - Day 2

[1]

J18 Day of the week recalled:

1 - Monday
2 - Tuesday
3 - Wednesday
4 - Thursday
5 - Friday
6 - Saturday
7 - Sunday

IC'S 24-HOUR FOOD RECALL (DAY ONE)

J19 I would like you to tell me all about what you ate yesterday that is, from the time you woke up until you went to bed, including snacks.

START WITH FIRST MEAL OR SNACK OF THE DAY

0 -	Before-breakfast snack	4 -	Afternoon snack
1 -	Breakfast	5 -	Supper
2 -	Morning snack	6 -	Evening snack
3 -	Lunch		

ENTER CODE IN COL. J19 OF TABLE J-1

J20 What dishes did you have for breakfast (lunch, supper, snacks)?
WRITE NAME OF DISH IN COL. J20 OF TABLE J-1

J21 How was the dish prepared?

1 -	Boiled	6 -	Steamed
2 -	Fried	7 -	Baked
3 -	Sauteed	8 -	Processed
4 -	Broiled/roasted	9 -	Raw
5 -	Scrambled	-8 -	NR/DK

ENTER CODE IN COL. J21 OF TABLE J-1

J22 Where was the dish prepared?

1 - Home (includes any home setting or home extension like a relative's home, office)
2 - Restaurant (carenderia, cafeteria)
3 - Feeding program
4 - Ambulant food vendor, street foods
5 - Store (sari-sari, grocery, bakery, etc.)
-8 - NR/DK
-9 - NA

ENTER CODE IN COL. J22 OF TABLE J-1

J23 Where was the dish eaten?

1 -	At home	-8 -	NR/DK
2 -	Away from home	-9 -	NA

ENTER CODE IN COL. J23 OF TABLE J-1

J24 What were the ingredients (food items) composing the dish that you have eaten?

ENTER ALL FOOD ITEMS EATEN IN COL. J24 OF TABLE J-1. USE A SEPARATE LINE FOR EACH INDIVIDUAL FOOD ITEM. IF THE SAME DISH WAS EATEN AT DIFFERENT MEALS, DO NOT WRITE "SAME" BUT SPELL OUT ITEM EACH TIME!

J25 Dish Number
DISHES FORMING PART OF A MEAL ARE NUMBERED CONSECUTIVELY, STARTING WITH # 1. IF A DISH CONSISTS OF MORE THAN ONE ITEM, EACH ITEM BELONGING TO THE DISH MUST HAVE THE SAME DISH NUMBER

J26 What were the specifics of these food items?
DESCRIBE FOOD ITEM CLEARLY AS TO FORM, KIND, COLOR, SIZE, e.g., WHAT KIND OF MEAT, FISH, CEREAL, FRUIT, OR PART OF IT.

ENTER ANSWER IN COL. J26 OF TABLE J-1

J27 Food Code
TO BE ACCOMPLISHED BY DIETARY EDITORS

J28 Amount consumed
ENTER AMOUNT IN COL. J28 OF TABLE J-1

J29 Unit of Measurement

1 -	Cup	4 -	Piece
2 -	Tbsp	5 -	Pack, bottle
3 -	Matchbox (mbx)	6 -	Tsp

SPELL OUT IN COL. J29 OF TABLE J-1

J30 Was the food you ate yesterday your usual food intake? ☐

0 -	No	CONTINUE
1 -	Yes	GO TO NEXT BLOCK

J31 If not, why?

VERBATIM:____ ☐

____

-8 - NR/DK -9 - NA

Table J-1. IC's Food Recall (Day One)

MEAL CODE	NAME OF DISH	COOKING METHOD	WHERE PREPARED	WHERE EATEN	FOOD ITEMS DISH CONTAINS	DISH NUM	FOOD ITEM DESCRIPTION	FOOD CODE	AMOUNT CONSUMED	UNIT OF MEASURE	
J19	J20	J21	J22	J23	J24	J25	J26	J27	J28	J29	

IC'S 24-HR FOOD RECALL
Date of interview: MONTH/DAY/YEAR ______________________________

J32 Food Recall: [2]

1 - Day 1
2 - Day 2

J33 Day of the week recalled: [ ]

1 - Monday
2 - Tuesday
3 - Wednesday
4 - Thursday
5 - Friday
6 - Saturday
7 - Sunday

IC'S 24-HOUR FOOD RECALL (DAY TWO)

J34 I would like you to tell me all about what you ate yesterday that is, from the time you woke up until you went to bed, including snacks.

START WITH FIRST MEAL OR SNACK OF THE DAY

0 -	Before-breakfast snack	4 -	Afternoon snack
1 -	Breakfast	5 -	Supper
2 -	Morning snack	6 -	Evening snack
3 -	Lunch		

ENTER CODE IN COL. J34 OF TABLE J-2

J35 What dishes did you have for breakfast (lunch, supper, snacks)?
WRITE NAME OF DISH IN COL. J35 OF TABLE J-2

J36 How was the dish prepared?

1 -	Boiled	6 -	Steamed
2 -	Fried	7 -	Baked
3 -	Sauteed	8 -	Processed
4 -	Broiled/roasted	9 -	Raw
5 -	Scrambled	-8 -	NR/DK

ENTER CODE IN COL. J36 OF TABLE J-2

J37 Where was the dish prepared?

1 - Home (includes any home setting or home extension like a relative's home, office)
2 - Restaurant (carenderia, cafeteria)
3 - Feeding program
4 - Ambulant food vendor, street foods
5 - Store (sari-sari, grocery, bakery, etc.)
-8 - NR/DK
-9 - NA

ENTER CODE IN COL. J37 OF TABLE J-2

J38 Where was the dish eaten?

1 -	At home	-8 -	NR/DK
2 -	Away from home	-9 -	NA

ENTER CODE IN COL. J38 OF TABLE J-2

J39 What were the ingredients (food items) composing the dish that you have eaten?

ENTER ALL FOOD ITEMS EATEN IN COL. J39 OF TABLE J-2. USE A SEPARATE LINE FOR EACH INDIVIDUAL FOOD ITEM. IF THE SAME DISH WAS EATEN AT DIFFERENT MEALS, DO NOT WRITE "SAME" BUT SPELL OUT ITEM EACH TIME!

J40 Dish Number
DISHES FORMING PART OF A MEAL ARE NUMBERED CONSECUTIVELY, STARTING WITH # 1. IF A DISH CONSISTS OF MORE THAN ONE ITEM, EACH ITEM BELONGING TO THE DISH MUST HAVE THE SAME DISH NUMBER

J41 What were the specifics of these food items?

DESCRIBE FOOD ITEM CLEARLY AS TO FORM, KIND, COLOR, SIZE, e.g., WHAT KIND OF MEAT, FISH, CEREAL, FRUIT, OR PART OF IT.

ENTER ANSWER IN COL. J41 OF TABLE J-2

J42 Food Code
TO BE ACCOMPLISHED BY DIETARY EDITORS

J43 Amount consumed

ENTER AMOUNT IN COL. J43 OF TABLE J-2

J44 Unit of Measurement

1 -	Cup	4 -	Piece
2 -	Tbsp	5 -	Pack, bottle
3 -	Matchbox (mbx)	6 -	Tsp

SPELL OUT IN COL. J44 OF TABLE J-2

J45 Was the food you ate yesterday your usual food intake? ☐

0 -	No	CONTINUE
1 -	Yes	GO TO NEXT BLOCK

J46 If not, why?

VERBATIM:______________________________ ☐

____

-8 -	NR/DK	-9 -	NA

Table J-2. IC's Food Recall (Day Two)

MEAL CODE	NAME OF DISH	COOKING METHOD	WHERE PREPARED	WHERE EATEN	FOOD ITEMS DISH CONTAINS	DISH NUM	FOOD ITEM DESCRIPTION	FOOD CODE	AMOUNT CONSUMED	UNIT OF MEASURE	
J34	J35	J36	J37	J38	J39	J40	J41	J42	J43	J44	

BLOCK K: MORBIDITY, RISK-TAKING BEHAVIORS, AND REPRODUCTIVE HEALTH OF IC

MORBIDITY

K1 Have you been sick since our last visit?

0 - No GO TO K4
1 - Yes CONTINUE

K2 What were/are you sick of?
1________________________________

2________________________________

3________________________________

4________________________________

5________________________________

6________________________________

-8 - NR/DK
-9 - NA

IF ANY OF THE ILLNESSES IN TABLE K-1 IS MENTIONED, ASK K2A AND K2B FOR EACH ILLNESS AND ENTER RESPONSES IN TABLE K-1

K2A When did you start having (NAME OF ILLNESS)?

ENTER YEAR OF ONSET OF ILLNESS IN COLUMN K2A OF TABLE K-1
-8 - NR/DK
-9 - NA

K2B Are you currently taking medication for this illness?

0 - No
1 - Yes (SPECIFY MEDICATION)

TABLE K-1. ILLNESS RECORD

ILLNESS	YEAR OF ONSET	MEDICATION (What kind?)
	K2A	K2B
Diabetes		
Heart disease		
Hypertension		

K3 Were you hospitalized because of this illness/any of these illnesses?

0 - No
1 - Yes Specify which illness(es) :________________
-9 - NA

K4 Since our last visit, have you received any immunization?

0 - No GO TO K6
1 - Yes CONTINUE
-8 - NR/DK

K5 What type of immunization was it?
LIST VACCINATIONS, DO NOT CODE
1 ________________________

2 ________________________

-8 - NR/DK
-9 - NA

RISK-TAKING BEHAVIORS

K6 Do you smoke?

0 - No GO TO K9
1 - Yes CONTINUE
-8 - NR/DK

K7 How many cigarettes do you smoke each day? ___________ no. of sticks/day

0 - Less than 1 stick/day
-8 - NR/DK
-9 - NA

K8 If less than 1 stick per day, how many cigarettes do you smoke each week? _______ no. of sticks/week

-7 - Smokes at least 1 stick/day
-8 - NR/DK
-9 - NA

K9 Do other members of your household smoke?

0 - No GO TO K11
1 - Yes

K10 How many members of your household usually smoke inside the house?

1 - One
:
-8 - NR/DK
-9 - NA

RECORD RESPONSES TO QUESTIONS K11 TO K14 IN THE APPROPRIATE COLUMNS IN TABLE K-2

K11 Have you ever...
- drank alcoholic beverages?
- taken drugs?

K12 How old were you when you first tried...
- drinking alcoholic beverages?
- taking drugs?

K13 Who initiated you into...
- drinking alcoholic beverages?
- taking drugs?
VERBATIM:

K14 How often do you...
- drink alcoholic beverages?
- take drugs?

Table K-2. Risk-taking behaviors

ACTIVITY	EVER TRIED	AGE WHEN 1st TRIED	PERSON WHO INITIATED	STILL DOING NOW, HOW OFTEN?
	K11	K12	K13	K14
Drinking alcoholic beverages	0 - No GO TO DRUGS<br>1 - Yes			1 - Only occasionally<br>2 - Every week<br>3 - Every day<br>-7 - Stopped drinking<br>-9 - NA
Taking drugs	0 - No GO TO K15<br>1 - Yes<br>(If yes, specify drug(s) tried)<br>___________<br>___________<br>___________			1 - Only occasionally<br>2 - Every week<br>3 - Every day<br>-7 - Stopped taking drugs<br>-9 - NA<br><br>Specify drug ______________<br>____________________

K15 We would like to know how your health has been in general during your pregnancy. Please answer how frequently during your pregnancy did you experience these common feelings or problems.

ENTER CODES IN TABLE K-3

1 - None of the time
2 - Occasionally
3 - Most of the time

TABLE K-3. Feelings/Problems in the Past 4 Weeks

FEELINGS/PROBLEMS	CODES
You were happy	
You had headaches	
You had poor digestion	
You had difficulty falling asleep	
You felt lonely	
You were hopeful about the future	
People were unfriendly	
You were worried	
You felt you couldn't overcome difficulties	
You were able to face problems	
You felt people disliked you	
You enjoyed normal daily activities	
You thought of yourself as worthless	
You felt life isn't worth living	
You wished you were dead	
You had the idea of taking your own life	

K16 How would you rate your general health?

1 - Poor
2 - Good
3 - Excellent

K17 Are you wearing eyeglasses now?

0 - No
1 - Yes

K18 Do you/did you have any cavities or decayed teeth?

0 - No
1 - Yes

K19 Are your teeth still complete?

0 - No
1 - Yes GO TO K21

K20 How many teeth are lost or missing?

1 - One
:
-9 - NA

K21 Have you had your teeth filled?

0 - No GO TO K23
1 - Yes

K22 How many teeth have been filled?

1 - One
:
-9 - NA

K23 How many times do you usually take a bath in one week?

CODE NUMBER OF TIMES
-8 - NR

☐☐

REPRODUCTIVE HEALTH

K24 I will mention a list of problems women may experience. Please tell me if you have experienced any of these our last visit.

0 - No
1 - Yes

ENTER ANSWER IN COL. K24 OF TABLE K-4

Table K-4. Reproductive Health Problems

PROBLEM	Experienced? K24
1. Painful urination	
2. Itchiness in vaginal area	
3. Vaginal discharges/irritation	
4. Pain during intercourse	
5. Signs of pregnancy	
6. Other related problems, specify ____________________	
7. Painful menstruation (dysmenorrhea)	
8. Irregular menstruation	
9. Unusually excessive/too little flow	
10. Other related problems, specify ____________________	

K25 PERCEIVED STRESS SCALE

The questions in this scale ask you about your feelings and thoughts during the last four weeks. In each case, you will be asked to indicate how often you felt or thought a certain way. Although some of the questions are similar, there are differences between them and you should treat each one as a separate question. The best approach is to answer each question fairly quickly. That is, don't try to count up the number of times you felt a particular way, but rather indicate the alternative that seems like a reasonable estimate.

For each question, choose from the following alternatives:

0 - Never
1 - Almost never
2 - Sometimes
3 - Fairly often
4 - Very often

	0 - Never	1 - Almost never	2 - Sometimes	3 - Fairly often	4 - Very often
1. In the last 4 weeks, how often have you been upset because of something that happened unexpectedly?					
2. In the last 4 weeks, how often have you felt that you were unable to control the important things in your life?					
3. In the last 4 weeks, how often have you felt nervous and stressed?					
4. In the last 4 weeks, how often have you dealt successfully with irritating life hassles?					
5. In the last 4 weeks, how often have you felt that you were effectively coping with important changes that were occurring in your life?					
6. In the last 4 weeks, how often have you felt confident about your ability to handle your personal problems?					
7. In the last 4 weeks, how often have you felt that things were going your way?					
8. In the last 4 weeks, how often have you found that you could not cope with all the things that you had to do?					
9. In the last 4 weeks, how often have you been able to control irritations in your life?					
10. In the last 4 weeks, how often have you felt that you were on top of things?					

K26 Usually, about how many days per week do you wake up feeling rested? ENCIRCLE NUMBER ☐

0 1 2 3 4 5 6 7

END OF BLOCK K

BLOCK M: ASSESSMENTS

Date of measurement: MONTH/DAY/YEAR________________ [ ][ ] [ ][ ] [ ][ ][ ][ ]
MONTH DAY YEAR

M1 WEIGHT (IN KILOGRAMS)
UNIT NO.: _______________

INTERVIEWER: TAKE THREE MEASUREMENTS
e.g., 30.7

MEAS #1 [ ][ ][ ][.][ ]

MEAS #2 [ ][ ][ ][.][ ]

MEAS #3 [ ][ ][ ][.][ ]

M2 ARM CIRCUMFERENCE (IN CENTIMETERS)
INTERVIEWER: TAKE THREE MEASUREMENTS
e.g., 20.3

MEAS #1 [ ][ ][.][ ]

MEAS #2 [ ][ ][.][ ]

MEAS #3 [ ][ ][.][ ]

M3 TRICEPS SKIN-FOLD THICKNESS
UNIT NO.: _______________

INTERVIEWER: TAKE THREE MEASUREMENTS

MEAS #1 [ ][ ][.][ ]

MEAS #2 [ ][ ][.][ ]

MEAS #3 [ ][ ][.][ ]

M4 BICEP THICKNESS
INTERVIEWER: TAKE THREE MEASUREMENTS
e.g., 20.3

MEAS #1 [ ][ ][.][ ]

MEAS #2 [ ][ ][.][ ]

MEAS #3 [ ][ ][.][ ]

M5 SUBSCAPULAR SKIN-FOLD THICKNESS
INTERVIEWER: TAKE THREE MEASUREMENTS
e.g., 20.3

MEAS #1 [ ][ ][.][ ]

MEAS #2 [ ][ ][.][ ]

MEAS #3 [ ][ ][.][ ]

M6 SUPRA-ILIAC THICKNESS
INTERVIEWER: TAKE THREE MEASUREMENTS
e.g., 20.3

MEAS #1 [ ][ ][.][ ]

MEAS #2 [ ][ ][.][ ]

MEAS #3 [ ][ ][.][ ]

M7 BLOOD PRESSURE (mm Hg)
UNIT NO.: ________________

INTERVIEWER: TAKE THREE MEASUREMENTS

Measurement #1 ☐☐☐/☐☐☐
Systolic Diastolic

Measurement #2 ☐☐☐/☐☐☐
Systolic Diastolic

Measurement #3 ☐☐☐/☐☐☐
Systolic Diastolic

M8 BIILIAC DIAMETER (CM)
UNIT NO.: ________________

INTERVIEWER: TAKE THREE MEASUREMENTS

Measurement #1 ☐☐☐.☐

Measurement #2 ☐☐☐.☐

Measurement #3 ☐☐☐.☐

M9 Hemoglobin/Fingerprick (g/dL) ☐☐.☐
UNIT NO.: ________________
LOT NO.: ________________

M10 A1C Now/Diabetes Test ☐.☐
UNIT NO.: ________________
LOT NO.: ________________

M11 Blood spot card collected? ☐

0 No (If NO, please indicate (below) the reason why not collected)
1 Yes

Remarks: ____

END OF BLOCK M

END OF INTERVIEW

CEBU LONGITUDINAL HEALTH AND NUTRITION STUDY
NSF BIRTH QUESTIONNAIRE

ID1 Type of Survey: [2] [0] [0] [9] [3]

ID2 Current Barangay: ______________________ [ ] [ ] [ ]
(See BARANGAY CODE LIST)

ID3 Current Stratum: 1 - Urban 2 - Rural [ ]

ID4 Baseline ID Number: [ ] [ ] [ ] [ ] [ ] [ ] [ ] [ ] [ ] [ ]
BRGY HHLD WMAN

ID5 Index Child's ID Number: [ ] [ ] [ ] [ ] [ ]

ID6 Mother's ID Number: [ ] [ ] [ ] [ ] [ ]

ID7 Sex of Index Child:

2 - Female [2]

ID8 Last Interview of Index Child:

1 - 2007
2 - 2005 [ ]

ID9 Name of Index Child: ______________________

Address at last visit: ______________________

____

Current address:
____

____

Contact Number: ______________________

ID10 INTERVIEWER: Is the birth interview address same as the address at pregnancy?

0 - No
1 - Yes [ ]

ID11 Name of Interviewer: ______________________ [ ] [ ]

ID12 Date of Interview Completion/Household Visit: [ ][ ] [ ][ ] [ ][ ][ ][ ]

MONTH DAY YEAR

ID13 INTERVIEW STATUS:
Is Index Child available for this interview? [ ]

0 - No (CONTINUE)
1 - Yes (GO TO CALL RECORD AND PROCEED WITH MAIN INTERVIEW)

ID14 Reason why not interviewed: [ ]

1 - Index Child deceased

X1 DATE OF DEATH: [ ][ ] [ ][ ][ ][ ]

-8 - NR/DK Month Year
-9 - NA

X2 CAUSE OF DEATH [ ][ ]

DESCRIBE: ____
-8 - NR/DK
-9 - NA

END INTERVIEW

2 - Index Child moved out of last known address to another area in Metro Cebu; and unlocated

Address: ____

3 - Index Child moved out of last known address outside Metro Cebu

X3 WHEREABOUTS OF IC BASED ON HOUSEHOLD INFORMANT:

1 - Outside Metro Cebu, in Cebu province [ ]
2 - Outside Cebu province, specify: ______________________________
-7- DK/No available information
-8 - NR
-9 - NA

4 - Index Child refused
5 - Index child institutionalized
6 - Index child incapacitated
-9 - NA

CALL RECORD

CALL NO.	DATE	TIME		RESULTS (Use codes below)	APPOINTMENT MADE	
		Started	Finished		Date	Time

RESULT CODES:
1 - Interview completed
2 - Interview partly completed, new appointment made
3 - Appointment made for interview later
4 - Refusal, no interview obtained
5 - No respondent at home
6 - Other (SPECIFY)

ID15 Total Number of Sessions Required to Complete Interview: ☐

BLOCK L: BIRTH OUTCOMES/DELIVERY INFORMATION

L1 What was the outcome of the pregnancy? ☐

1 - Single live birth
2 - Single stillbirth
3 - Miscarriage/Abortion
4 - Twin birth, both born alive
5 - Twin birth both stillbirth
6 - Twin birth one born alive and one dead
7 - Other multiple births: specify details _
____
__

IF SINGLE BIRTH GO TO ID16

OTHERWISE READ THE FOLLOWING INSTRUCTIONS FOR MULTIPLE BIRTHS:

a) USE A SEPARATE QUESTIONNAIRE TO FILL OUT ITEMS ID16-ID18, L3-L14, L33-L37, AND ICC ANTHROPOMETRY (WHATEVER APPLIES) FOR EACH BIRTH (WHETHER BORN ALIVE OR BORN DEAD).

b) RECORD THE FF: L2 Number of females (born alive or born dead) ☐

Number of males (born alive or born dead) ☐

ID16 INDEX CHILD'S CHILD (ICC) NUMBER ☐

1- singleton or child #1(for multiple births)
2- child #2
3- child #3

ID17 ICC NAME ____

LEAVE BLANK IF NO NAME GIVEN (FOR CHILDREN BORN DEAD OR WHO DIED)

ID18 ICC UNC ID (OFFICE ASSIGNED) ☐☐☐☐☐☐☐

L3 Sex of child, born dead or alive. ☐

1 - Male
2 - Female

L4 Date of pregnancy termination

☐☐ ☐☐ ☐☐☐☐

MONTH DAY YEAR

L5 TIME OF DAY:

CODE 12-HOUR CLOCK

HOUR MINUTE

HOUR (range: 1-12) MINUTE (range: 1-59)

AM/PM

1 - AM
2 - PM

IF PREGNANCY TERMINATED IN A MISCARRIAGE, END INTERVIEW AT THIS POINT

L6 Was the infant, born dead or alive, ever weighed by anyone (including hospital) at any time after delivery?

0 -	No, baby has not been weighed yet	GO TO L9
1 -	Yes, first hour of life	CONTINUE
2 -	Yes, later on first day of life	CONTINUE
3 -	Yes, second day of life	CONTINUE
4 -	Yes, third day of life	CONTINUE
5 -	Yes, fourth day of life	CONTINUE
6 -	Yes, fifth day of life	CONTINUE
7 -	Yes, after fifth day of life	CONTINUE
-8 -	NR/DK	GO TO L9

L7 What was the weight when the infant, born dead or alive, was weighed for the first time?

INTERVIEWER: ENTER WEIGHT _____________ KGS OR ________________LBS

EDITOR: CODE BIRTH WEIGHT IN KG

-8 - NR/DK
-9 - NA

L8 INTERVIEWER: INDICATE SOURCE OF DATA FOR BIRTH WEIGHT

1 from birth record (or any official birth document)
2 mother's recall

IF STILLBIRTH, END INTERVIEW AT THIS POINT

L9 What is the current status of the infant?

1 - Still living GO TO L15
2 - Infant died CONTINUE

L10 On what day (date) did the infant die? ________________________

-8 - NR/DK
-9 - NA

MONTH DAY YEAR

L11 How many hours or days did the infant live? _____________

ENTER RESPONSE IN HOURS. IF ANSWER IS GIVEN IN TERMS OF DAYS, CONVERT DAYS INTO HOURS. INQUIRE ABOUT EXACT NUMBER OF HOURS OF FIRST AND LAST DAY.

0 - Less than one hour
-8 - NR/DK
-9 - NA

L12 What was the reason for the infant's death?
WRITE DOWN ANSWER VERBATIM. DO NOT CODE!

____

____

-8 - NR/DK
-9 - NA

L13 Was the infant fed at any time before death?

1 - Yes CONTINUE
0 - No
-8 - NR/DK } GO TO L15
-9 - NA

L14 What day of his life was the infant fed for the last time?

1 - First day of life
2 - Second day of life
3 - After second day of life
-8 - NR/DK
-9 - NA

L15 Who delivered your infant?

0 - Woman delivered by herself
1 - No practitioner, (only relatives helped)
2 - Public doctor
3 - Public nurse
4 - Public midwife
5 - Private doctor
6 - Private nurse
7 - Private midwife
8 - Mananabang
9 - Others (SPECIFY) ______________________
-8 - NR/DK
-9 - NA

L16 Where did the delivery take place?

1 - At home
2 - Barangay health station
3 - Public hospital
4 - Private hospital
5 - Public clinic/office (RHU/CHO/Puericulture center)
6 - Private clinic/office
7 - Others (SPECIFY) ______________________
-8 - NR/DK
-9 - NA

L17 How did you deliver?

1 - Caesarian
2 - Vaginal/natural
-8 - NR/DK
-9 - NA

L18 How long did your entire labor last, from the very beginning of labor* until the baby was born/delivered?

1 - <5 hours
2 - 5-10 hours
3 - 11-15 hours
4 - >15 hours
-7 - C-section
-8 - NR/DK
-9 - NA

*As soon as mother is aware or is told she is in labor

L19 How long was the time from when your water broke until the baby was born/delivered?

1 - <5 hours
2 - 5-10 hours
3 - 11-15 hours
4 - >15 hours
-7 - C-section
-8 - NR/DK
-9 - NA

L20 How long was the time from when you started pushing hard until the baby was born/delivered?

1 - <15 minutes
2 - 15-30 minutes
3 - 31-45 minutes
4 - >45 minutes
-7 - C-section
-8 - NR/DK
-9 - NA

L21 How much time did it take for the placenta to be delivered after the baby was born/delivered?

1 - <15 minutes
2 - 15-30 minutes
3 - 31-45 minutes
4 - >45 minutes
-4 - Placenta had to be removed by doctor/nurse/midwife
-7 - C-section
-8 - NR/DK
-9 - NA

L22 Was anesthesia used in the delivery?

0 - No
1 - Yes
-8 - NR/DK
-9 - NA

L23 Were there any complications in the delivery?

0 - No complication
1 - Yes, heavy bleeding
2 - Yes, infection
3 - Yes, prolonged labor (more than 24 hours)
4 - Yes, breech
5 - Yes, forceps had to be used
6 - Yes, cord coil
7 - Yes, dry labor
8 - Others (SPECIFY):_______________________________
-8 - NR/DK
-9 - NA

L24 Was your mother near you (or present in the room) when you delivered?
0 - No
1 - Yes
-8 - NR/DK
-9 - NA

L25 Was your husband near you (or present in the room) when you delivered?

0 - No
1 - Yes
-8 - NR/DK
-9 - NA

IF DELIVERY WAS AT HOME ASK L26 OTHERWISE GO TO L27

L26 When delivery took place, how long (IN HOURS) was the practitioner with you in your house? (INDICATE EXACT NUMBER OF HOURS) _______________

0 - Less than one hour
1 - One hour
:
-8 - NR/DK
-9 - NA

GO TO L29

L27 How many days before delivery did you stay in the hospital/clinic?

0 - Less than one full day
1 - One day
2 - Two days
:
-8 - NR/DK
-9 - NA

L28 How many days after delivery did you stay in the hospital/clinic?

0 - Less than one full day
1 - One day
2 - Two days
:
-8 - NR/DK
-9 - NA

L29 What was the total delivery cost (IN PESOS)?

ADD UP ALL COSTS (INCLUDING HOSPITALIZATION, DOCTOR'S FEES, MEDICINES, NURSERY FEES, AND OTHER DELIVERY-RELATED COSTS.

ROUND OFF VALUE TO NEAREST PESO.

0 - No costs
-8 - NR/DK
-9 - NA
} GO TO L33

L30 Does your family have any insurance like GSIS, SSS, Philhealth or any other private health insurance?

0 -	No	GO TO L33
1 -	Yes	CONTINUE
-8 -	NR/DK	GO TO L33
-9 -	NA	

L31 Did the insurance pay for all or part of the costs of delivery and confinement?

0 -	No	GO TO L33
1 -	Yes, all costs	CONTINUE
2 -	Yes, part of the costs	CONTINUE
-8 -	NR/DK	GO TO L33
-9 -	NA	

L32 What amount of the total cost for the delivery and confinement was paid by the insurance? ___________

ENTER AMOUNT TO NEAREST PESO
-8 - NR/DK
-9 - NA

INFANT FEEDING/BREASTFEEDING

L33 Have you initiated breastfeeding?

0 -	No	GO TO ICC ANTHROPOMETRY
1 -	Yes	CONTINUE

L34 Did you give the first flow from your breast to the baby?

0 - No
1 - Yes
-8 - NR/DK

L35 How many times during the first 24 hours of the infant's life did you breastfeed the infant?

1 - Once
2 - Twice
:
-8 - NR/DK
-9 - NA (Infant was not breastfed on first day)

L36 Approximately how long (IN MINUTES) was the infant on the breast(s) every time he/she was breastfed?
____

-8 - NR/DK
-9 - NA

L37 Approximately how long (IN MINUTES) was the infant actively sucking every time he/she was breastfed?
____

-8 - NR/DK
-9 - NA

ICC ANTHROPOMETRY

Date of measurement: [ ][ ] [ ][ ] [ ][ ][ ][ ]
MONTH DAY YEAR

Measurement	1	2	3
L38 Length (cm) UNIT NO._______			
L39 Weight (kg) UNIT NO._______			
L40 Tricep (mm) UNIT NO._______			
L41 Bicep (mm)			
L42Subscapular (mm)			
L43 Suprailiac (mm)			
L44 Calf (mm)			
L45 Thigh (mm)			
L46 Arm Circumference (cm)			
L47 Head Circumference (cm)			
L48 Abdominal Circumference (cm)			

BLOCK M: MATERNAL ANTHROPOMETRY (IC)

Date of measurement: [ ][ ] [ ][ ] [ ][ ][ ][ ]
MONTH DAY YEAR

M1 WEIGHT (IN KILOGRAMS)
UNIT NO.: ________________

INTERVIEWER: TAKE THREE MEASUREMENTS
e.g., 30.7

Measurement #1 [ ][ ][ ].[ ]

Measurement #2 [ ][ ][ ].[ ]

Measurement #3 [ ][ ][ ].[ ]

M2 ARM CIRCUMFERENCE (IN CENTIMETERS)
INTERVIEWER: TAKE THREE MEASUREMENTS
e.g., 20.3

Measurement #1 [ ][ ][ ][ ]

Measurement #2 [ ][ ][ ][ ]

Measurement #3 [ ][ ][ ][ ]

M3 TRICEPS SKIN-FOLD THICKNESS
UNIT NO.: ________________

INTERVIEWER: TAKE THREE MEASUREMENTS

Measurement #1 [ ][ ][ ][ ]

Measurement #2 [ ][ ][ ][ ]

Measurement #3 [ ][ ][ ][ ]

M4 BICEP THICKNESS
INTERVIEWER: TAKE THREE MEASUREMENTS

Measurement #1 [ ][ ][ ][ ]

Measurement #2 [ ][ ][ ][ ]

Measurement #3 [ ][ ][ ][ ]

M5 SUBSCAPULAR SKIN-FOLD THICKNESS
INTERVIEWER: TAKE THREE MEASUREMENTS

Measurement #1 ☐☐☐☐

Measurement #2 ☐☐☐☐

Measurement #3 ☐☐☐☐

M6 SUPRA-ILIAC THICKNESS
INTERVIEWER: TAKE THREE MEASUREMENTS

Measurement #1 ☐☐☐☐

Measurement #2 ☐☐☐☐

Measurement #3 ☐☐☐☐

M7 BLOOD PRESSURE (mm Hg)
UNIT NO.: ________________

INTERVIEWER: TAKE THREE MEASUREMENTS

Measurement #1 ☐☐☐/☐☐☐
Systolic Diastolic

Measurement #2 ☐☐☐/☐☐☐
Systolic Diastolic

Measurement #3 ☐☐☐/☐☐☐
Systolic Diastolic

M8 Hemoglobin/Fingerprick (g/dL) ☐☐.☐
UNIT NO.: ________________
LOT NO.: ________________

M9 Blood spot card collected? ☐

0 No (If NO, please indicate (below) the reason why not collected)
1 Yes

Remarks: ____

END OF BLOCK M

END OF INTERVIEW
